# *Akkermansia muciniphila* Reduces Peritonitis and Improves Intestinal Tissue Wound Healing after a Colonic Transmural Defect by a MyD88-Dependent Mechanism

**DOI:** 10.3390/cells11172666

**Published:** 2022-08-27

**Authors:** Radu Bachmann, Matthias Van Hul, Pamela Baldin, Daniel Léonard, Nathalie M. Delzenne, Clara Belzer, Janneke P. Ouwerkerk, Dirk Repsilber, Ignacio Rangel, Alex Kartheuser, Robert Jan Brummer, Willem M. De Vos, Patrice D. Cani

**Affiliations:** 1 Metabolism and Nutrition Research Group, Louvain Drug Research Institute, Walloon Excellence in Life Sciences and BIOtechnology (WELBIO), UCLouvain, Université Catholique de Louvain, 1200 Brussels, Belgium; 2 Colorectal Surgery Unit, Cliniques Universitaires Saint-Luc, 1200 Brussels, Belgium; 3 Pathology Department, Cliniques Universitaires Saint-Luc, UCLouvain, 1200 Brussels, Belgium; 4 Laboratory of Microbiology, Wageningen University, 6700 EH Wageningen, The Netherlands; 5 Nutrition-Gut-Brain Interactions Research Centre, School of Medical Sciences, Faculty of Medicine and Health, Örebro University, S-701 82 Örebro, Sweden; 6 Human Microbiome Research Program, Faculty of Medicine, University of Helsinki, 00014 Helsinki, Finland

**Keywords:** *Akkermansia muciniphila*, wound healing, colonic leakage, peritonitis, Myd88

## Abstract

Anastomotic leakage is a major complication following colorectal surgery leading to peritonitis, complications, and mortality. *Akkermansia muciniphila* has shown beneficial effects on the gut barrier function. Whether *A. muciniphila* reduces peritonitis and mortality during colonic leakage is unknown. Whether *A. muciniphila* can directly modulate the expression of genes in the colonic mucosa in humans has never been studied. We investigated the effects of a pretreatment (14 days) with live *A. muciniphila* prior to surgical colonic perforation on peritonitis, mortality, and wound healing. We used mice with an inducible intestinal-epithelial-cell-specific deletion of MyD88 (IEC-MyD88 KO) to investigate the role of the innate immune system in this context. In a proof-of-concept pilot study, healthy humans were exposed to *A. muciniphila* for 2 h and colonic biopsies taken before and after colonic instillation for transcriptomic analysis. Seven days after colonic perforation, *A.-muciniphila*-treated mice had significantly lower mortality and severity of peritonitis. This effect was associated with significant improvements of wound histological healing scores, higher production of IL22, but no changes in the mucus layer thickness or genes involved in cell renewal, proliferation, or differentiation. All these effects were abolished in IEC-MyD88 KO mice. Finally, human subjects exposed to *A. muciniphila* exhibited an increased level of the bacterium at the mucus level 2 h after instillation and significant changes in the expression of different genes involved in the regulation of cell cycling, gene transcription, immunity, and inflammation in their colonic mucosa. *A. muciniphila* improves wound healing during transmural colonic wall defect through mechanisms possibly involving IL22 signaling and requiring MyD88 in the intestinal cells. In healthy humans, colonic administration of *A. muciniphila* is well tolerated and changes the expression of genes involved in the immune pathways.

## 1. Introduction

Anastomotic leakage remains a major and potentially life-threatening complication following colorectal surgery. Despite numerous technical surgical advancements and the development of perioperative enhanced recovery methods in the last decades [1,2], the percentage of colonic anastomotic leakages did not improve significantly. The literature shows that the prevalence of anastomotic leakage ranges from 1 to 19%, depending on the anatomical site, and that, in addition to the economic implications, this has direct impact on survival, quality of life, and cancer recurrence [3,4]. The mortality rates in case of an anastomotic leakage vary in the literature, typically from 15 to 33% [5].

Despite growing knowledge in this field, it is still difficult to accurately predict anastomotic leakage occurrence and, so far, there is no adequate preoperative bowel preparation that significantly helps to reduce its incidence [6]. Different preventive strategies have been proposed aimed at influencing factors that have been identified as impairing anastomotic healing [7,8,9]. Kirchhoff [10], for example, classified these factors into two categories: factors that can be influenced (e.g., obesity, nutritional status, preoperative bowel cleaning, experience of the surgeon) and predisposing or risk factors that are not under our control (e.g., age and gender). Strikingly, many of these risk factors have also been shown to affect the composition of the microbiota [11,12,13,14,15,16,17], while at the same time they can themselves be deeply impacted by the microbiota, suggesting that the gut microbiota has a role in intestinal wound healing [3].

In this study, we investigated in an in vivo mouse model whether administration of a next-generation beneficial bacteria prior to surgery could modulate the intestinal environment so that healing is promoted, reducing the risk for anastomotic leakage, and enhancing post-surgery recovery in general. We have recently developed a novel, fast-track, innovative intestinal wound healing model in mice called the ‘colonoscopic leakage model’, in which we create a controlled transmural perforation in the colon by using a biopsy grasper during a colonoscopy. This model was validated and showed its important advantages over the classical ‘anastomotic leakage model’ by decreasing the invasiveness [18]. *Akkermansia muciniphila* is a commensal bacterium that was isolated from the human gut microbiota in 2004 [19]. This bacterium represents between 0.5 and 5% of the microbial community in healthy human subjects and is specialized in colonizing the mucus layer, being one of the few bacteria living close to the intestinal epithelial cells [19,20]. This anaerobic, Gram-negative bacterium of the phylum Verrucomicrobia has been shown to digest mucus using mucolytic enzymes encoded in its genome [21,22]. *A. muciniphila* has been previously associated with beneficial metabolic effects in different pathological situations linked with an altered gut barrier function such as during obesity, type 2 diabetes, and aging [23,24,25,26], and is considered a next-generation beneficial bacterium. In a mechanistic point of view, we and others have previously discovered that *A. muciniphila* activates different receptors such as Toll-like receptor 2 (TLR2) and TLR4 [27,28,29] and modulates the immune system, thereby reinforcing the gut barrier [28,29,30,31,32]. It has also been shown that IL22 was upregulated by a treatment with *A. muciniphila* [31]. A disruption of the intestinal barrier upon injury to intestinal epithelial cells is known to lead to the exposure of numerous TLR ligands produced by commensals and eventually may induce intestinal inflammation but also peritonitis, and mice lacking TLR2, TLR4, or the myeloid differentiation primary response 88 (MyD88) are more sensitive to these diseases [32,33,34,35]. MyD88 is a central adaptor molecule for most TLRs, but more importantly MyD88 is involved in the control of IL22 expression and regulation. Given that *A. muciniphila* has been shown to activate different receptors and to modulate the Immune system and reinforce the gut barrier [27,28,29,30], and that IL22 was strongly upregulated by the treatment with *A. muciniphila*, we investigated whether the beneficial effects of *A. muciniphila* on the wound healing were mechanistically dependent on the expression of MyD88 on the intestinal epithelial cells. 

Here, we investigate the putative impact of *A. muciniphila* on host recovery and wound healing in the context of colorectal surgery in wild-type mice and mice with an inducible intestinal-epithelial-cell-specific deletion of MyD88. We also explored the direct impact of 2 h exposure to *A. muciniphila* on the expression of genes in the colon of human volunteers.

## 2. Materials and Methods

### 2.1. Ethics

All mouse experiments were approved by the Ethical Committee for Animal Care of the Health Sector of the Université Catholique de Louvain (UCLouvain), under number 2018/UCL/MD/013, and were performed in accordance with the guidelines of the Local Ethics Committee and in accordance with the Belgian Law of 29 May 2013, regarding the protection of laboratory animals (agreement number LA1230314). 

### 2.2. Animals

Experiments 1 and 2 were designed to determine the effects of pretreating mice with *A. muciniphila* Muc^T^ (ATTC BAA-835) on body weight, organ weight, mortality, peritonitis severity, wound healing score, and different intestinal markers in the colonoscopic leakage model. For experiments 1 and 2 (replicate experiments performed independently), 9-week-old male C57BL/6J mice were purchased from Janvier (Le Genest-Saint-Isle, France). The mice were housed in pairs under strict specific and opportunistic pathogen-free (SOPF) conditions and in a controlled environment (room temperature of 22 ± 2 °C, 12-h daylight cycle, lights off at 6 pm) with free access to food and water. After an acclimatization period of one week, mice were randomly assigned to one of two conditions: the control group, gavaged with vehicle (anaerobic PBS with 25% (vol/vol) glycerol) (CT, *n* = 20 per experiment), or the test group, gavaged with 200 µL of a solution containing 1 × 10^9^ live cells of *A. muciniphila* Muc^T^ (ATTC BAA-835) per ml (Akk, *n* = 20 per experiment). *A. muciniphila* Muc^T^ was cultured anaerobically in a synthetic medium as previously described [19,29]. Gavages were performed daily for three weeks in total. After two weeks of gavages, a colonoscopic controlled perforation was performed following the procedure described below (day 0). Gavages were continued until day 7, when mice were killed under anesthesia and tissues were collected (see below). 

Experiment 3 aimed at investigating whether mice with an intestinal epithelial (IEC)-specific deletion of Myd88 (IEC-Myd88 knock-out (KO)) were developing a different phenotype than control mice on body weight, mortality, and peritonitis severity after transmural perforation using the colonoscopic leakage model. IEC-Myd88 KO mice were generated by crossing mice bearing a tamoxifen-dependent Cre recombinase expressed under the control of the villin promoter (Villin Cre-ERT2) with mice harboring a *loxP*-flanked *Myd88* allele (C57BL/6 background, Jackson-Laboratory), as previously described [36]. The deletion was induced at 9 weeks of age by intra peritoneal (i.p.) injection of 100 μL tamoxifen (10 mg mL^−1^) for 5 consecutive days. Tamoxifen was prepared by addition of ethanol to 50 mg of tamoxifen (tamoxifen-free base, MP Biomedicals) to obtain a 10 mg per 100 μL of tamoxifen suspension. A 10 mg ml^−1^ tamoxifen solution was prepared by addition of filtered sunflower oil, followed by 30 min sonication. The 10 mg mL^−1^ of tamoxifen solution was stored at 4 °C for up to 1 week. The tamoxifen solution was sonicated just before use. In parallel, the control group was injected intraperitoneally (i.p.) with 100 μL saline solution for 5 consecutive days. Due to COVID-19-related restrictions, the colonic leakage procedure on these mice, as described below, was performed at the age of 22 weeks. 

Experiment 4 aimed at investigating whether mice treated with *A. muciniphila* but lacking Myd88 specifically in the intestinal epithelial cells (IEC-Myd88 KO) were responding or not to the treatment and whether they developed a different phenotype than control mice on body weight, mortality, peritonitis severity, and intestinal markers in the colonoscopic leakage model. For this experiment, we used 11-week-old, male, IEC-Myd88 KO mice that we obtained as mentioned above, but with the usual wash out period. They were randomly assigned to one of two conditions: the control IEC-MyD88 KO group, gavaged with vehicle (IEC-Myd88 KO, *n* = 20), or the test group gavaged with 200 µL of a solution containing 1 × 10^9^ live cells of *A. muciniphila* per mL (IEC-Myd88 KO Akkermansia, *n* = 17), with gavages performed for 2 weeks before proceeding to the colonoscopic controlled perforation (day 0). The mice were followed up closely and the gavages were continued for one week until the sacrifice (day 7), when the mice were killed under anesthesia and tissues were collected for analysis. 

### 2.3. Colonoscopic Leakage Model

The colonoscopy leakage model was performed as described in Bachmann et al., Gut, 2020 [18]. This model has several advantages over the traditionally used “anastomotic leakage model” and was shown to decrease invasiveness and ameliorate recovery of the animals while remaining close to the human situation [18]. 

In brief, mice were anaesthetized using isoflurane gas (Forene, Abbott, Queenborough, Kent, UK), in accordance with welfare criteria (3% induction and of 2.5% maintenance dose). A colonoscopy was performed, during which a transmural wounding was induced just before the left colic flexure (i.e., 3.5 cm ab anno and lateral in regard to the colonic mesentery with a diameter of 5 mm, analogues to the grasper jaws, which correspond to the gap between two stiches of a “bad” anastomosis) by using 3Fr-biopsy forceps. Mice quickly returned to their habitual environment and were observed while recovering. Mice that showed signs of severe discomfort or pain in the first few hours after the procedure were eliminated from analysis, as this was considered to be a direct cause of the procedure rather than of the transmural wound, and they showed no overt sign of peritonitis (*n* = 1 in the 1st experiment, *n* = 0 in the 2nd experiment, *n* = 0 in the 3rd experiment, *n* = 2 in the 4th experiment). One of the advantages of using this technique is that the mice do not need a preoperative bowel preparation, nor do they need to be fasted before the intervention. 

### 2.4. Follow Up 

Body weight was monitored before and every day after the procedure and pain/discomfort was assessed daily. Mice were sacrificed after 7 days, which corresponds to the last phase of wound remodeling, during which the new epithelium is developed, and the final scar tissue is formed. In all our procedures, we abstained from using antibiotics either pre- or post-intervention.

### 2.5. Sacrifice/Peritonitis Score

At sacrifice, 7 days after colonic perforation, we could distinguish different levels of severity of peritonitis and attribute a corresponding score: 

(0) Mice in which no peritonitis was observed. 

(1) Mice with a peritonitis that was localized only at the site of perforation and that did not result in death. 

(2) Mice with a generalized peritonitis affecting the whole abdominal cavity, but that did not result in death.

(3) Mice that died before the end of the 7-day period that had only a localized peritonitis.

(4) Mice that died and displayed clear signs of a generalized peritonitis.

This scoring allowed for a sensitive appreciation of the outcome and a fast comparative evaluation of our experimental groups. 

### 2.6. Tissue Sampling

At the end of the experimental period, mice were anesthetized with isoflurane (3% induction) (Forene; Abbott) before exsanguination and tissue sampling, then mice were sacrificed by cervical dislocation. Liver, spleen, and caecum were precisely dissected and weighed.

After dissection of the colon and establishment of the peritonitis score, the zone of trauma was localized using 20× binoculars and samples were taken of three different locations: the proximal colon (healthy colon), the zone next to the perforation zone at a distance of 15 mm, and the perforation zone itself. 

In experiment 1, the colonic tissue collected of the three different zones was used for histological analysis, whereas in the second experiment it was used for qPCR analysis.

### 2.7. Histological Analysis

For experiment 1, the thickness of the mucus layer was measured at the site of perforation and at a more distal, unaltered section of the colon. The tissues were fixed in Carnoy’s solution (ethanol 6: acid acetic 3: chloroform 1, *v*/*v*) for 24 h at room temperature. Samples were then immersed in 100% ethanol for 24 h before processing for paraffin embedding and preparation of 5 µm tissue sections. Paraffin sections of 5 μm were stained with Alcian blue [37]. Analysis was performed on at least 2 high-resolution whole-slide images per mouse and a minimum of 20 different measurements were made perpendicular to the inner mucus layer per field. Analyses were performed using ImageJ (version 1.48r, National Institutes of Health, Bethesda, MD, USA) in a blinded manner by two different observers. 

For histopathological examination, sequenced sections every 100 μm spanning the entire perforation site were stained with H&E and analyzed by an experienced pathologist in a blinded manner. For this purpose, we used the recently developed histological score [38]. 

This score takes into consideration parameters such as “Fibroblasts”, “Inflammatory cells”, “Blood vessel ingrowth”, and “Collagen formation”, in order to assess in which of the different phases of wound repair the tissue of the collected sample is located. This score permits to globally assess the evolution of the wound healing, by quantifying the “Number of healed layers”, “Epithelium closed”, and “Crypt architecture restored” and determining the “Overall healing quality”.

### 2.8. RNA Preparation and Real-Time qPCR Analysis 

Total RNA was prepared from collected tissues using TriPure reagent (Roche, Basel, Switzerland). Quantification and integrity analysis of total RNA was performed by running 1 μL of each sample on an Agilent 2100 Bioanalyzer (Agilent RNA 6000 Nano Kit, Agilent, Santa Clara, CA, USA). cDNA was prepared by reverse transcription of 1 µg total RNA using a Reverse Transcription System Kit (Promega, Madison, WI, USA). Real-time PCR was performed with the CFX96 Real-time PCR system and CFX manager 3.1 software (BioRad, Hercules, CA, USA) using GoTaq qPCR Master Mix (Promega) for detection, according to the manufacturer’s instructions. RPL19 RNA was chosen as the housekeeping gene, and data were analyzed according to the 2^−ΔΔCT^ method. The identity and purity of the amplified product were assessed by melting curve analysis at the end of amplification.

### 2.9. Proof-of-Concept Study of Local Instillation of A. muciniphila in Healthy Humans 

In parallel with the rodent study, we performed an exploratory study of local instillation of 10^11^ cells/day (in 100 mL PBS) of the alive form of *A. muciniphila* Muc^T^ through a catheter placed with the aid of colonoscopy in the left colon, with continuous dripping of the liquid for two hours. *A. muciniphila* Muc^T^ (ATTC BAA-835) was produced at a food-grade level according to the HACCP quality system as described [29] and tested in a human cohort [39]. The volunteers did not have a colonic prep prior to the intervention. They were split in two groups, one placebo (*n* = 3) receiving only 100 mL PBS, and the other one (*n* = 7) receiving *A. muciniphila*, as previously described [39].

The design of the procedure was to start with a fecal sampling and a distal colonoscopy including mucosal biopsies, before placing the catheter in the distal descending colon region to administrate the live *A. muciniphila.* After two hours of continuous administration, the catheter was removed, and new mucosal biopsy specimens were sampled in the same region. A last mucosal biopsy with stool sample collection was performed at one week after the perfusion procedure. 

The biopsies were analyzed for total human transcriptome on microarrays, and they were used equally to quantify the *A. muciniphila* levels at the mucus level. The fecal samples were equally analyzed for *A. muciniphila* levels using qPCR and for total microbiota composition by 16S rRNA phylogenetic microarray. Detailed methods for the labelling and subsequent hybridizations of the arrays are described in the eukaryotic section of the GeneChip Expression Analysis Technical Manual, from Affymetrix (Affimetrix, Santa Clara, CA, USA). The data can be found in GEO repository under the number GSE211506 (https://www.ncbi.nlm.nih.gov/geo/query/acc.cgi?acc=GSE211506 accessed on 18 August 2022).

### 2.10. Statistical Analysis

**Figure 1:**
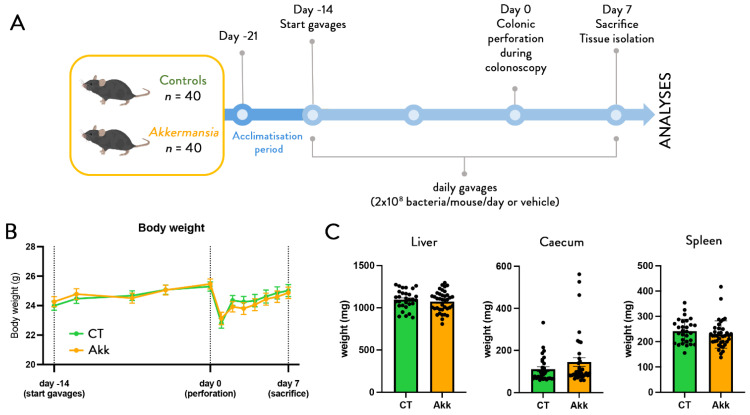
***A. muciniphila* does not affect body weight and organ weights.** (**A**) Experimental design. (**B**) Body weight evolution before and after colonic perforation in control mice and mice treated with *A. muciniphila*. (**C**) Weight of liver, caecum, and spleen (*n* = 40 per group) corresponding to the results of two independent experiments. Data are represented as mean +/− SEM and black dots (dotplots) represent individual values for each animal, statistical difference assessed using *t*-test, *p*-value > 0.05.

**Figure 2:**
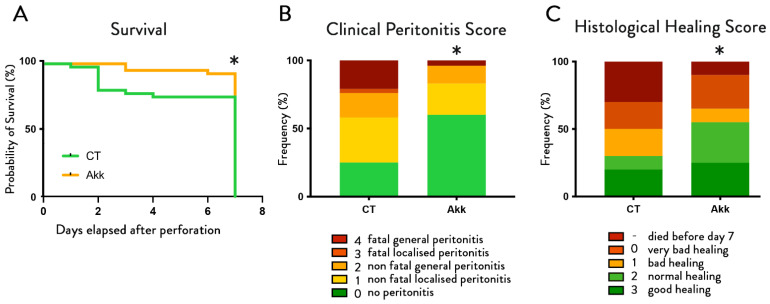
***A. muciniphila* administration reduces mortality and peritonitis severity and improves healing score**. Effect of *A. muciniphila* administration on (**A**) survival (*n* = 40 per group) and (**B**) peritonitis severity (*n* = 40 per group) and (**C**) healing score (*n* = 20 per group) in a colonoscopic leakage model. Survival *p*-value = 0.0224 Gehan–Breslow–Wilcoxon test, clinical peritonitis score * corresponds to the statistics: *p*-value < 0.0001, and histological healing score *p*-value < 0.0001 Chi-square test.

**Figure 3:**
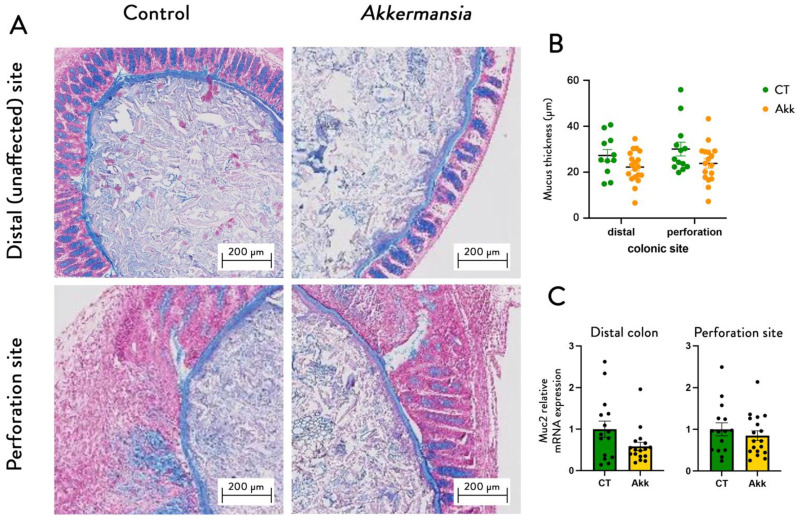
***A. muciniphila* administration does not affect mucus thickness and *Muc2* expression.** (**A**) Representative Alcian blue-stained images of the mucus layer in the distal colon and the perforation site of mice treated with or without *A. muciniphila*. (**B**) Mucus thickness measurements. (**C**) Relative mRNA expression of Muc2. Data are represented as mean +/− SEM and black dots (dotplots) represent individual values for each animal. Statistical difference assessed using non-parametric Mann–Whitney test, *p*-value > 0.05.

**Figure 4:**
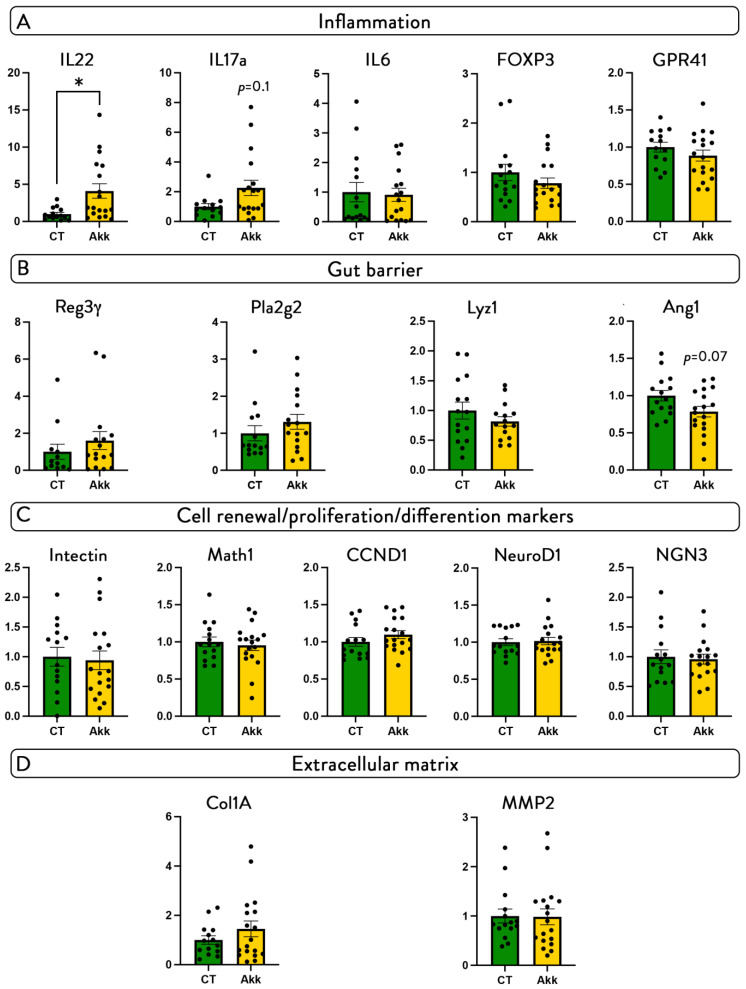
***A. muciniphila* administration increases IL22 and IL17 without affecting other markers of inflammation, gut barrier, cell renewal, proliferation markers, and matrix formation.** Relative mRNA expression of markers of (**A**) inflammation, (**B**) gut barrier integrity, (**C**) cell renewal, proliferation, and differentiation, (**D**) extracellular matrix at the site of perforation. Data are shown as mean ± SEM, black dots (dotplots) represent individual values for each animal; * *p*-values were obtained after non-parametric Mann–Whitney test.

**Figure 5:**
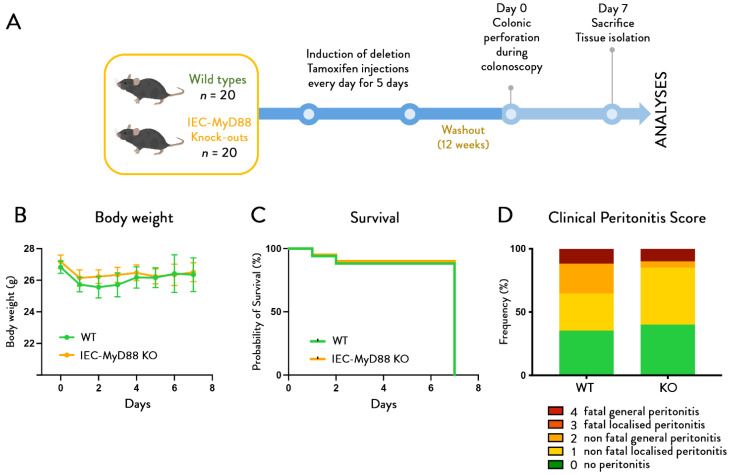
**Mice with intestinal-epithelial-cell-specific deletion of Myd88 are similarly sensitive as wild-type mice to mortality and peritonitis.** (**A**) Experimental design. (**B**) Body weight evolution, (**C**) survival rates, and (**D**) peritonitis scores after colonic perforation in control mice and mice with an intestinal-epithelial-cell-specific deletion of Myd88 (IEC-villinMYD88 knock-outs (*n* = 20 per group). Data are shown as mean ± SEM. Probability of survival assessed using Gehan–Breslow–Wilcoxon test and Chi-square test for clinical peritonitis score, *p*-value > 0.05.

**Figure 6:**
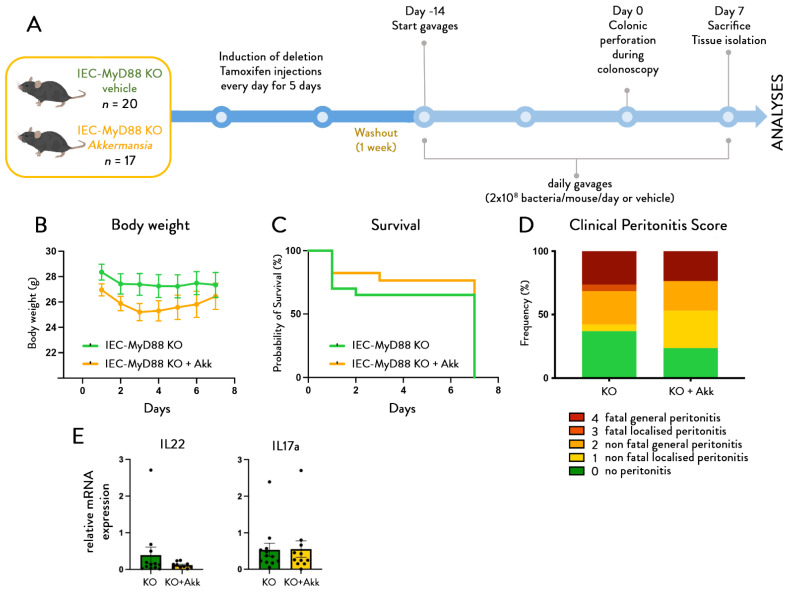
**Mice with intestinal epithelial cell-specific deletion of Myd88 are not responding to the beneficial effects of *A. muciniphila* on mortality and peritonitis**. (**A**) Experimental design. (**B**) Body weight evolution, (**C**) survival rates, and (**D**) peritonitis scores after colonic perforation. (**E**) IL22 and IL17 in the colon of mice with an intestinal-epithelial-cell-specific deletion of Myd88 (IEC-MYD88 KO) with or without treatment with *A. muciniphila* (*n* = 20 per group). Data are shown as mean ± SEM, black dots (dotplots) represent individual values for each animal. Probability of survival assessed using Gehan–Breslow–Wilcoxon test and Chi-square test for clinical peritonitis score, *p*-value > 0.05. Non-parametric Mann–Whitney test used to assess IL22 and IL17, *p*-value > 0.05.

The data are presented as the means ± SEM. The statistical significance of the difference was evaluated by different specific tests specifically mentioned in the legends of the figures: *t*-test for the organ weights (Figure 1C), Gehan–Breslow–Wilcoxon test for the survival curve (Figure 2A, Figure 5C and Figure 6C), Chi-square test for the clinical peritonitis score and the histological healing score (Figure 2B,C, Figure 5D and Figure 6D), and non-parametric Mann–Whitney test in Figure 3, Figure 4 and Figure 6E. All the analyses were performed using GraphPad Prism version 9.00 for Mac (GraphPad Software, San Diego, CA, USA). The presence of outliers was assessed using the Grubbs test.

## 3. Results

### 3.1. A. muciniphila Treatment Reduces Mortality and Peritonitis

The procedure (Figure 1A) resulted in only 1/80 peri-interventional mortality on a total of 80 mice (replicate experiments 1 and 2). The mice recovered fast and displayed normal behavior as soon as the anesthetics wore off. Body weight loss was limited, transient, and did not differ between groups (Figure 1B). There were no overt differences in weights of liver, spleen, nor caecum (Figure 1C).

Mice treated with *A. muciniphila* had lower mortality rates (*p*-value = 0.0224) (Figure 2A). At sacrifice, 7 days after colonic perforation, we could distinguish different levels of severity of peritonitis and attributed a corresponding score to each mouse. The reproducibility of this scoring was confirmed independently by two different examiners, blinded for the groups, without any difference in the evaluation. This showed a significantly (*p*-value < 0.0001) lower incidence of peritonitis for the *A.-muciniphila*-treated mice (Figure 2B). In the control group, 25% of the mice did not show any signs of peritonitis at day 7 (10/40 mice), whereas daily *A. muciniphila* administration significantly increased this number to 60% (24/40 mice). Among the mice that developed peritonitis (75% for CT vs. 40% for mice treated with *A. muciniphila*), 26% were fatal in CT mice, whereas this number dropped to only 5% when mice were treated with *A. muciniphila*. 

### 3.2. A. muciniphila Improves Wound Healing without Affecting the Mucus Layer Thickness

Histological scoring of the wound healing process at the site of perforation after one week showed an improvement of the overall healing quality after *A. muciniphila* supplementation with a “normal” or “good” overall healing quality of 55% in the *A. muciniphila*-treated mice, as compared to only 30% in the control group (*p*-value < 0.0001) (Figure 2C). The percentage of wounds with a closed epithelium were 60% (12/20 mice) in the *A. muciniphila*-treated mice and 40% (8/20 mice) in control mice. 

We analyzed the thickness of the mucus layer in both groups at two different sites: within the site of perforation and in a more distal, undisturbed part of the colon. This revealed no differences between the sites, nor groups (Figure 3), nor did we find any differences in expression profiles of Muc2, a marker for mucus production. Quantification of this mucus layer thickness revealed no differences between the sites, nor groups (Figure 3B), nor did we find any differences in expression profiles of Muc2 (Figure 3C). 

### 3.3. A. muciniphila Increases the Expression of IL22 in the Colon 

IL22 is a cytokine immediately produced to initiate immune response against gut barrier impairment and is known as a key mediator involved in wound healing, tissue regeneration, and mucous production, thereby acting as a protective factor against pathogens [40]. Moreover, *A. muciniphila* has been shown to modulate the immune system [41]. Interestingly, we found that *A. muciniphila* significantly upregulated the expression of IL22 by 400% (*p*-value = 0.008) and doubled the expression of IL17A (Figure 4A), although this effect did not reach significance (*p*-value = 0.1). The other markers were not affected by the treatment (Figure 4A).

Next, we analyzed different relevant markers of gut barrier integrity, including antimicrobial peptides (Reg3γ, Lyz1, Ang1, Pla2g2) and markers of intestinal cell renewal, differentiation, and proliferation (CCND1, intectin, Math1, NGN3 and NeuroD1), but found no differences in gene expression between both groups, both at the unharmed distal part of the colon (not shown) and at the site of perforation (Figure 4B,C). We also found no effect on collagen 1A (COL1A) and matrix metalloproteinase 2 (MMP2), which is involved in extracellular remodeling (Figure 4D). 

### 3.4. A. muciniphila Reduces Mortality and Peritonitis and Improves Wound Healing by an Intestinal-Epithelial-Cell-Specific MyD88-Dependent Mechanism 

The intestinal innate immune system is one of the most important factors involved in the interactions between gut microbes and hosts. As stated above, *A. muciniphila* has been shown to activate TLR2 and TLR4 [27,28,29]. Although we could not observe any increase in the expression of TLR2 and TLR4 in the colon of mice treated with *A. muciniphila* (Figure S1), it is known that the recognition of pathogen-associated molecular patterns by the intestinal epithelial cells is important and is mediated mostly via the protein MyD88. MyD88 is involved in the control of IL22 expression and regulation. Given that *A. muciniphila* has been shown to activate TLRs to reinforce the gut barrier [27,28,29], and that IL22 was strongly upregulated by the treatment with *A. muciniphila,* we investigated whether the beneficial effects of *A. muciniphila* on the wound healing were mechanistically dependent on the expression of MyD88 on the intestinal epithelial cells. 

To this aim, we used mice with an inducible intestinal-epithelial-cell-specific deletion of MyD88. In a first experiment (Figure 5A), we determined whether the intestinal-epithelial-cell-specific inactivation of MyD88 itself had any effect on body weight, survival, and peritonitis severity. We found that intestinal-epithelial-cell-specific deletion of MyD88 did not affect the development of peritonitis, and no differences between both genotypes were observed for any of the parameters tested (Figure 5B–D). 

Next, we determined whether *A. muciniphila* could still positively affect the phenotype of IEC-MyD88 KO mice (Figure 6A,B). We found that daily administration of *A. muciniphila* in mice lacking intestinal MyD88 did not improve survival (Figure 6C) or peritonitis scores, and the *Akkermansia*-treated mice had a worse incidence of developing peritonitis then the non-treated ones (Figure 6D), in mice lacking intestinal MyD88, thereby showing that *A. muciniphila* needs a functional intestinal epithelial MyD88 protein to exert its protective effects. In addition, we found *A. muciniphila* treatment failed to increase both IL22 and IL17 (Figure 6E), thereby also showing that mice lacking intestinal MyD88 are not responding to the beneficial effects of *A. muciniphila.*

### 3.5. A. muciniphila Changes the Expression of Genes Involved in Cell Cycling, Gene Transcription, and Immune and Inflammatory Pathways in the Colon of Healthy Humans 

Although the data obtained in rodents are interesting, the present findings should be confirmed in humans. However, before starting any large-scale clinical trial, we need to verify whether *A. muciniphila* can directly affect the expression of genes in the colonic mucosa and whether the exposure to *A. muciniphila* is well tolerated and does not induce any adverse events. Therefore, we performed a proof-of-concept, exploratory study in healthy humans. Our data showed that the local intestinal instillation of *A. muciniphila* is not associated with any negative effects on the subjects, as none of them experienced any side effects.

**Figure 7:**
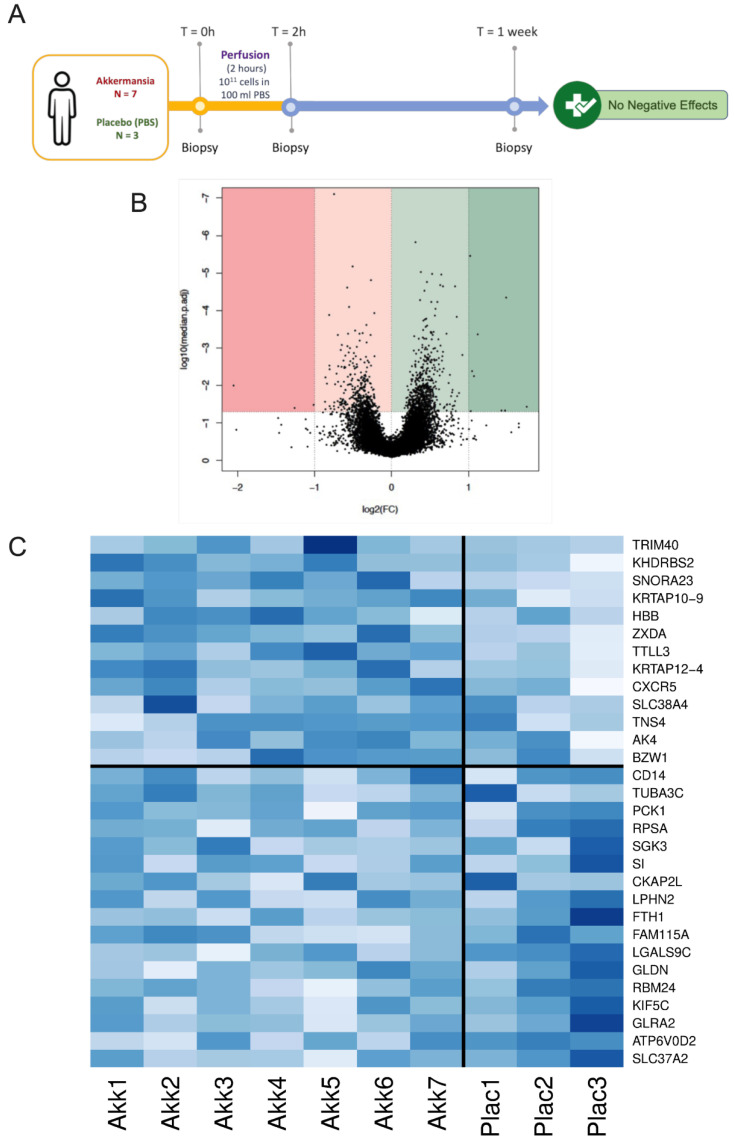
**Proof-of-concept study in healthy humans treated 2 h with *A. muciniphila* on gene expression versus baseline treatment and placebo**. (**A**) Experimental design and volcano plot showing transcriptional analysis on colonic biopsies after 2 h perfusion versus basal. (**B**) Dark and light pink left parts of the volcano plot represent the genes downregulated 2 h after exposure to *A. muciniphila*, whereas the dark and light green on the right part represent the genes upregulated 2 h after exposure to *A. muciniphila*. Both dark pink and dark green parts are showing genes for which the expression is changed by a magnitude of at least 2-fold after 2 h perfusion with *A. muciniphila* versus basal (i.e., before exposure). (**C**) The heatmap is based on the 30 genes displaying significant (adj-*p* values smaller than 0.05) and largest absolute fold changes in the comparison *A. muciniphila* (Akk) vs. placebo (Plac). In the heatmap, the genes presented above the horizontal line represent upregulation of *A. muciniphila* vs. placebo while the genes presented below the horizontal line represent downregulation of *A. muciniphila* vs. placebo. Every single column represents one subject, i.e., exposed to *A. muciniphila* (left of the vertical line) or exposed to placebo (right of the vertical line). The genes changed, their levels of expression, names, and statistical changes are shown in Supplementary Table S1.

The quantitative analysis of the *A. muciniphila* levels did not show an effect in the fecal samples but, interestingly, an increased level at the mucus layer, in the mucosal biopsies taken at two hours after start of perfusion, but not persisting in the samples taken after one week (not shown). There was no significant effect found when analyzing the fecal microbiota composition prior and after *A. muciniphila* instillation. Interestingly, by using a transcriptomic analysis, we found that numerous genes were decreased or increased due to the *A. muciniphila* treatment, mostly genes involved in cell cycling, gene transcription, and immune and inflammatory pathways, but also in metabolic pathways of the intestinal cells (Figure 7 and Supplementary Table S1). More specifically, among the specific genes increased by *A. muciniphila*, we found a significant increase in genes related to the regulation of immunity and inflammation. For instance, TRIM40 (tripartite motif containing 40) is a gene described to be involved in the regulation of the activity of the TLR4 signaling pathway and NF-κB (nuclear factor-kappa B) [42] (Figure 7). TRIM40 is known to promote the neddylation of the inhibitor of NF-κB subunit gamma, a crucial regulator for NF-κB activation, and as a consequence leads to strong inhibition of the NF-κB activity [43]. Along the same line, we found that the expression of the gene coding for MARCO (macrophage receptor with collagenous structure) was significantly increased (Supplementary Table S1). This marker is a scavenger receptor expressed on the cell surface of macrophages that mediates opsonin-independent phagocytosis. It has also been suggested that MARCO regulates the activation status of the immune system and can be involved in balancing a proper immune response to infection. This regulation could also be important in maintaining tolerance [44,45]. We also identified numerous changes in the expression of the gene coding for gene transcription regulation, energy metabolism, and cell cycling (e.g., regulation of alternative splicing, ribosome biogenesis, tubulin formation, transporters). These data are in line with previous studies in rodents showing that the monocolonization of mice with *A. muciniphila* modulates the expression of different colonic genes [41]. 

## 4. Discussion

More and more studies suggest that the local intestinal microbiota play an influential role during healing of a colonic anastomosis [46]. A recent metanalysis focusing on clinical trials, studying the impact of the pre- and postoperative administration of available pre- and probiotics, showed positive results with a reduction in the length of stay in hospital, as well as lower rates of surgical site infections. Unfortunately, these measures were not sufficient to avoid colonic anastomotic leakages [47] and the need for adequate interventions remains. For this reason, we decided to test the impact of the administration of *A. muciniphila* using the newly developed ‘colonoscopic leakage model’. 

We found that the administration of *A. muciniphila* two weeks prior to and one week after the operative intervention resulted in a significant improvement of the survival rate and was associated with lower rates of peritonitis and less severe peritonitis. Histological examination revealed considerable improvement of healing outcomes and these effects were linked to higher IL22 production. More importantly, we performed a first proof-of-concept study in human volunteers by instilling *A. muciniphila* directly in the colon and measuring transcriptomic markers in colonic biopsies 2 h after the continuous exposure to the bacterium. Our data suggest that *A. muciniphila* is safe and can rapidly induce changes in the expression of genes in the colonic mucosa exposed to *A. muciniphila*. 

There have been several mechanisms described by which *A. muciniphila* can exert its beneficial effects and affect host physiology [11]. These include improvement of the gut barrier function [27,28,29,30], immunomodulation of the adaptive immune system [41], and activation of anti-inflammatory responses [28,48]. *A. muciniphila* has previously been proposed to restore mucin production [30,49] by being involved in a positive feedback loop, whereby through mucin degradation it also stimulates mucin production [50]. Therefore, we measured mucus thickness in our mice to evaluate whether this might contribute to an improved gut barrier function and have a protective effect on wound healing. However, we found no impact of *A. muciniphila* administration on mucus production. Nevertheless, it should be noted that the studies showing a restoration of the mucus production after the administration of *A. muciniphila* have been conducted in mice fed with high-fat diets [29,30,51] and aging mice [24]. In this context, the mucus production is impaired, and the mucus layer is thinner. In the present study, the mucus layer thickness is comparable to that in a normal colon [50].

In addition to the physical protection conferred by the mucus layer to keep pathogens from getting into the body and helping with the wound healing, the biochemical barrier is another important mechanism that helps keep commensal bacteria at a distance and fend off pathogenic bacteria. Specific antimicrobial peptides (AMPs) [52] such as regenerating islet derived-3 gamma (Reg3γ), phospholipase A2 group II (Pla2g2), Lysozyme C (Lyz1), and angiogenin 1 (Ang1) act as a firewall against certain bacteria by affecting their viability and are involved in intestinal epithelial cell turnover [53]. However, our analysis of several relevant markers of gut barrier integrity did not show any differences between treated and untreated mice.

Although we found no effects on markers of gut barrier integrity, cell renewal, or extracellular matrix, we found an increase in IL22 and IL17a expression at the site of perforation. IL22 has been shown to contribute to epithelial cell expansion, proliferation, and AMP production and to improve barrier function in human and mouse models [40].

Like the *A. muciniphila* effect, mucins are highly inducible by IL22 and constitute the thick mucosal layer that coats the epithelium and that is impenetrable to many commensal bacteria, thereby limiting their potential to cause inflammation [54]. IL22 has been shown to upregulate several mucus-associated genes, such as MUC1, MUC3, MUC10, and MUC13 in colonic epithelial cells [55]. 

In the acute phase, IL22 is known to recruit neutrophils toward the site of infection as an inflammatory response [56]. It may act independently but it can also synergize with other cytokines such as IL17 to elicit its role [57]. Regenerating islet-derived protein 3γ (REG3γ) mRNA expression in enterocytes and Paneth cells is controlled by microorganism-associated molecular patterns through Toll-like receptors (TLRs) and is dependent on the TLR signaling adaptor myeloid differentiation primary response protein 88 (MYD88) [58]. *Akkermansia muciniphila* had previously been positively correlated with intestinal expression of Reg3γ [30,53], a finding that we could not confirm in this study. However, the previous observations were made in the context of a high-fat diet and a downregulation of this marker. *A. muciniphila* has been proposed as a mediator of Myd88/TLR2 innate immune signaling and some evidence indicates that MyD88 is involved in the modulation of wound healing [59,60], but the underlying mechanism is still unclear. It was previously demonstrated that the TLR-induced secretion of IL22 requires Myd88 signaling and that a strong reduction in IL22 in MyD88 −/− was observed [61]. In line with this observation, our experiments using inducible intestinal-epithelial-cell-specific deletion of MyD88 revealed that *A. muciniphila* loses its beneficial effects if MyD88 is absent, suggesting that this adapter protein is required for transfer of *A. muciniphila* signaling. 

Although *A. muciniphila* seems to play an important role in the better outcome observed in our model, we cannot exclude that the local microbiota may also be involved by influencing the production of the intestinal antimicrobial proteins (AMPs) independent of this pathway [62], for example through the production of short chain fatty acid butyrate in the colon through microbial fermentation of dietary fiber [63]. 

Finally, by using a proof-of-concept approach we administered *A. muciniphila* directly in the colon of human volunteers for two hours. By using mucosal biopsies before, two hours, and one week after the initial exposure to *A. muciniphila* versus placebo, we found that the administration of the bacteria did not elicit any specific adverse events and modulates the expression of several genes involved in the regulation of immunity, gut barrier, and host metabolism. However, it is worth noting that among the genes induced by the exposition to *A. muciniphila,* several genes were identified that are under direct control of the TLR/MyD88/NF-κB pathways (e.g., higher TRIM40 and MARCO).

In addition, the safety profile observed here is in line with another human study showing that oral administration for 3 months of either live or pasteurized *A. muciniphila* is safe and well tolerated [29,39]. Therefore, our findings could be relevant for future human interventions. Although this pilot study is underpowered, this first exploratory study will be useful to design future prospective interventional studies to investigate the putative wound healing effects of *A. muciniphila* on the human colon. 

We have previously found in both mice and humans that *A. muciniphila* treatment was not associated with a specific change in the gut microbiota composition [30,39]. Although the microbiota was not assessed in the mouse study described here, we speculate that there are likely no specific changes in the microbiota, since we also found in the present human intervention that the exposure to *A. muciniphila* was indeed enriching the mucosa of the subject in *A. muciniphila* after the two hours of exposure, and this effect was transient and also did not affect the overall microbiota composition of the subjects (not shown). This is perfectly in line with our previous human study showing that the oral administration of *A. muciniphila* for 3 months of either live or pasteurized *A. muciniphila* did not affect the overall microbiota composition measured by sequencing [41]. 

Our animal model, which showed a significant enhancement of the intestinal wound healing, is based on a preoperative administration of *A. muciniphila* for 2 weeks. The current standard prehabilitation program (which is meant to improve the preoperative status of the patients with the help of physiotherapy, nutritional support, and psychological counseling) has a standard duration of 3 weeks and showed some improvement on the outcome but unfortunately no reduction in the anastomotic leakage rate [64]. If our results should prove to also be translatable to humans, it could be considered to add *A. muciniphila* supplementation to the existing protocol of prehabilitation, without delaying the timeline from diagnosis to operation.

## 5. Conclusions

**Figure 8:**
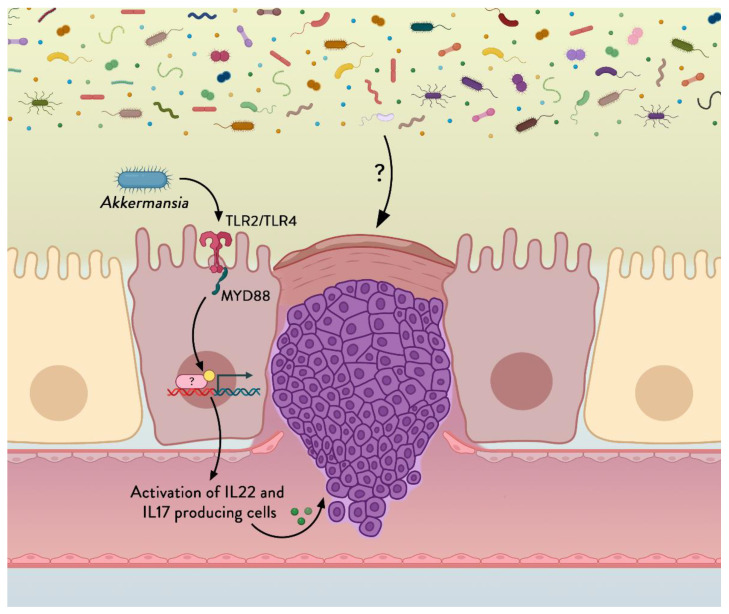
**Working and mechanistic model proposed.***A. muciniphila* activates Toll-like receptors (TLRs) and a signal is transferred via the adaptor protein MYD88 leading to the expression of certain yet unknown mediators that activate the production of IL22 and IL17, which in turn results in improved wound healing and better general outcome. (?) depicted potential other unidentified signals from the gut microbiota that may also influence this process.

Administrating live *A. muciniphila* improves wound healing of a transmural colonic wall defect (i.e., colonic anastomosis) in a mouse model through a mechanism requiring the MyD88-dependent pathway and possibly requiring IL22 signaling (Figure 8). Regarding the proof-of-concept study performed on human volunteers, we can conclude that in healthy human subjects the local administration of *A. muciniphila* is well tolerated and significantly changes the expression of genes involved in immune pathways.

This study illustrates how understanding the complex interactions between host and a specific potential beneficial microbe may support the development of future therapeutic strategies targeting the gut microbiota. However, the question remains if we can extrapolate these in vivo animal findings to humans, and additional experiments in humans are therefore inevitable.

## Data Availability

All the details regarding analytic methods and study are made available to other researchers as Supplementary Table S1.

## Supplementary Materials

The following supporting information can be downloaded at: https://www.mdpi.com/article/10.3390/cells11172666/s1, Supplementary Table S1: Genes changed, their levels of expression, names and statistical changes, Supplementary Figure S1: Expression of TLR2 and TLR4 in the colon of mice.
