# Supplementary material for: Akkermansia muciniphila Reduces Peritonitis and Improves Intestinal Tissue Wound Healing after a Colonic Transmural Defect by a MyD88-Dependent Mechanism
Source: Cells. 2022 Aug 27;11(17):2666. doi: 10.3390/cells11172666 (PMC9454966; doi:10.3390/cells11172666)
Supplement: Supplementary file 1 [file cells-11-02666-s001.zip › cells-1815005-supplementary.pdf]

Supplementary Figure S1 and Supplementary Table S1

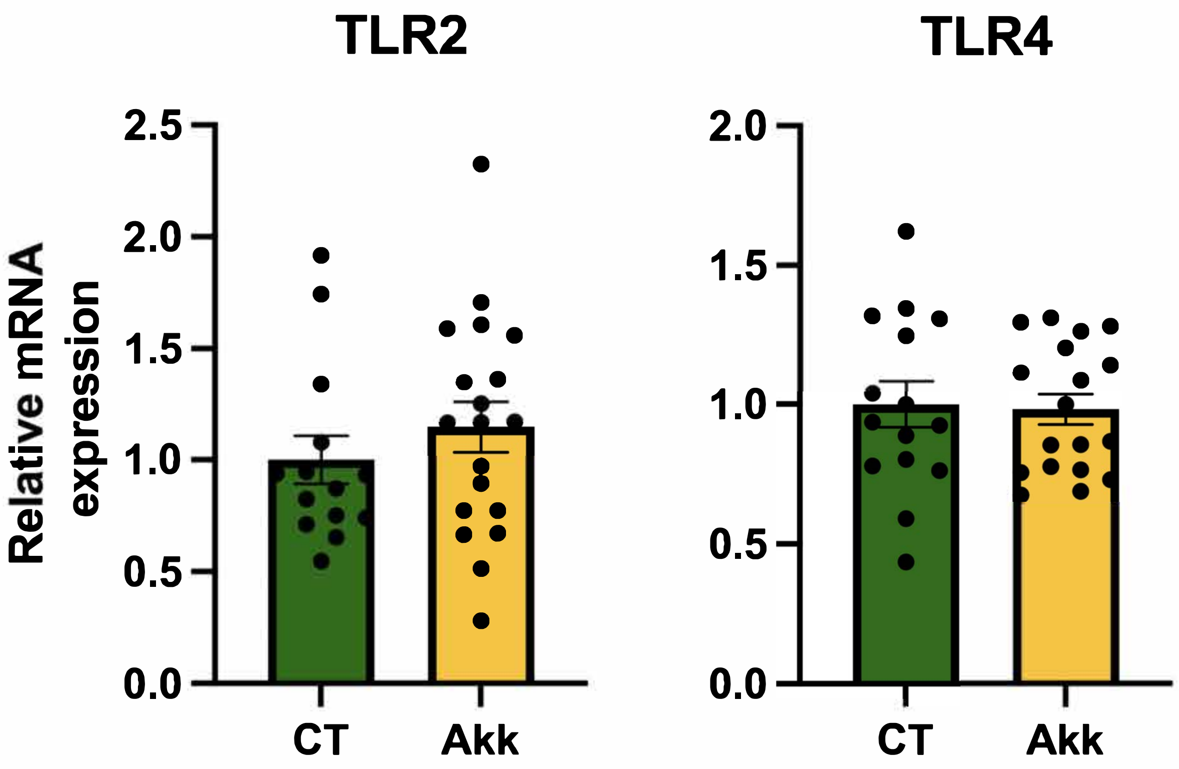

Supplementary Figure S1

Figure S1: Expression of TLR2 and TLR4 in the colon of mice

Supplementary Table S1 : Genes changed, their levels of expression, names and statistical changes

Bootstrap.median.coef	Bootstrap.median.padj	hgnc_symbo	entrezgene	description		
1.75425563095151	0.0380266869158546	HBB	3043	hemoglobin, beta [Source:HGNC Symbol;Acc:HGNC:4827]		
1.65057152292233	0.104309312384429		644316			
1.64899334906734	0.13511572419977		28670			
1.55382253255918	0.180619950539535	POTEC	388468	POTE ankyrin domain family, member C [Source:HGNC Symbol;Acc:HGNC:33894]		
1.48566022949559	4.53359313718493e-05	DUOXA2	405753	dual oxidase maturation factor 2 [Source:HGNC Symbol;Acc:HGNC:32698]		
1.47131140044583	0.0476646347453729	RPSA	3921	ribosomal protein SA [Source:HGNC Symbol;Acc:HGNC:6502]		
1.42550425026009	0.0475550837450573	TRIM40	135644	tripartite motif containing 40 [Source:HGNC Symbol;Acc:HGNC:18736]		
1.22833800611733	0.119561245179349	DUOX2	50506	dual oxidase 2 [Source:HGNC Symbol;Acc:HGNC:13273]		
1.1181936427167	0.000439356188258359	SGK3	23678	serum/glucocorticoid regulated kinase family, member 3 [Source:HGNC Symbol;Acc:HGNC:10812]		
1.07017878658419	0.00577308402440224		729080			
1.04621852119364	0.00423680763704285	SNORA66	26782	small nucleolar RNA, H/ACA box 66 [Source:HGNC Symbol;Acc:HGNC:10223]		
1.03996621651566	0.0885971902207602	RIMBP2	102724399	RIMS binding protein 2 [Source:HGNC Symbol;Acc:HGNC:30339]		
1.02867234244311	0.0495058256191728	TUBA3C	113457	tubulin, alpha 3c [Source:HGNC Symbol;Acc:HGNC:12408]		
1.02183630723827	3.44784700977278e-06	JMJD7	100137047	jumonji domain containing 7 [Source:HGNC Symbol;Acc:HGNC:34397]		
1.00481149671431	0.148756820290756		6974			
0.922430296504716	0.143190229959573	RPS15A	6210	ribosomal protein S15a [Source:HGNC Symbol;Acc:HGNC:10389]		
0.918933567870218	0.00156989145109039		400870			
0.907372884993112	0.196907402983972	VSNL1	7447	visinin-like 1 [Source:HGNC Symbol;Acc:HGNC:12722]		
0.875547805683607	0.0158139955929459	SOHLH2	54937	spermatogenesis and oogenesis specific basic helix-loop-helix 2 [Source:HGNC Symbol;Acc:HGNC:26026]		
0.870267626984605	0.0763026050400545	sept-05	5413	septin 5 [Source:HGNC Symbol;Acc:HGNC:9164]		
0.867469889924238	0.144172840051932	RPL13A	23521	ribosomal protein L13a [Source:HGNC Symbol;Acc:HGNC:10304]		
0.863871210837615	0.102396334992706	BRD7	29117	bromodomain containing 7 [Source:HGNC Symbol;Acc:HGNC:14310]		
0.849307640833656	0.0191705056982869	AK4	205	adenylate kinase 4 [Source:HGNC Symbol;Acc:HGNC:363]		
0.844009660843761	0.000152100727333339	SNORA23	677808	small nucleolar RNA, H/ACA box 23 [Source:HGNC Symbol;Acc:HGNC:32613]		
0.830984320187307	0.0481228956199483		728613			
0.823971018099701	0.0265473449727632	KRTAP10-9	386676	keratin associated protein 10-9 [Source:HGNC Symbol;Acc:HGNC:22971]		
0.823177950422004	2.25557305754141e-05	EYA2	2139	EYA transcriptional coactivator and phosphatase 2 [Source:HGNC Symbol;Acc:HGNC:3520]		
0.811932576768185	0.0352310627835041	ZXDA	7789	zinc finger, X-linked, duplicated A [Source:HGNC Symbol;Acc:HGNC:13198]		
0.80966908021506	0.082479394197989	CEP55	55165	centrosomal protein 55kDa [Source:HGNC Symbol;Acc:HGNC:1161]		
0.805974235445612	0.118311111787003		6983			
0.799977700216837	0.0802055585979907	CELP	1057	carboxyl ester lipase pseudogene [Source:HGNC Symbol;Acc:HGNC:1849]		
0.796229649566993	0.0812547041177988	NPEPL1	79716	aminopeptidase-like 1 [Source:HGNC Symbol;Acc:HGNC:16244]		
0.792991058053995	0.0359209846403291	SLC38A4	55089	solute carrier family 38, member 4 [Source:HGNC Symbol;Acc:HGNC:14679]		
0.790720761262143	0.0431826811249963	SNORD36A	26815	small nucleolar RNA, C/D box 36A [Source:HGNC Symbol;Acc:HGNC:10163]		
0.790151971414246	0.0132104139100015	ZNF503-AS2	100131213	ZNF503 antisense RNA 2 [Source:HGNC Symbol;Acc:HGNC:23525]		
0.788113245389458	0.193816806425746	HYPK	25764	huntingtin interacting protein K [Source:HGNC Symbol;Acc:HGNC:18418]		
0.787383864309299	0.0641891099020212		400709			
0.78666168618743	0.0885769474692941	CENPI	2491	centromere protein I [Source:HGNC Symbol;Acc:HGNC:3968]		
0.78501464329641	0.0157918650634903	PCDHA4	56144	protocadherin alpha 4 [Source:HGNC Symbol;Acc:HGNC:8670]		
0.774381177359694	0.0731350491577018	GGT1	2678	gamma-glutamyltransferase 1 [Source:HGNC Symbol;Acc:HGNC:4250]		
0.773071643819253	0.122574199164256	HIST1H2BM	8342	histone cluster 1, H2bm [Source:HGNC Symbol;Acc:HGNC:4750]		
0.772670713568634	0.0194321778573167	TNS4	84951	tensin 4 [Source:HGNC Symbol;Acc:HGNC:24352]		
0.772201945673727	0.050857240341917	LRRC37A4P	55073	leucine rich repeat containing 37, member A4, pseudogene [Source:HGNC Symbol;Acc:HGNC:25479]		
0.771918986794213	0.0567725356483673	GSTA1	2938	glutathione S-transferase alpha 1 [Source:HGNC Symbol;Acc:HGNC:4626]		
0.755704090659927	0.185234924486307	FST	10468	follistatin [Source:HGNC Symbol;Acc:HGNC:3971]		
0.754984008454195	0.00526838738792397		100533990			
0.75497037332074	0.0325830390260325	KHDRBS2	202559	KH domain containing, RNA binding, signal transduction associated 2 [Source:HGNC Symbol;Acc:HGNC:18114]		
0.7502931766651	0.0800236649756642	KRTAP10-2	386679	keratin associated protein 10-2 [Source:HGNC Symbol;Acc:HGNC:22967]		
0.739658590729137	0.0086711950082788	CKAP2L	150468	cytoskeleton associated protein 2-like [Source:HGNC Symbol;Acc:HGNC:26877]		
0.729824962791786	0.0913989892336977	CDC6	990	cell division cycle 6 [Source:HGNC Symbol;Acc:HGNC:1744]		
0.72073720695006	0.0951793327194653	KRTAP19-5	337972	keratin associated protein 19-5 [Source:HGNC Symbol;Acc:HGNC:18940]		
0.71989147229639	0.0324506753012523	TTLL3	26140	tubulin tyrosine ligase-like family, member 3 [Source:HGNC Symbol;Acc:HGNC:24483]		
0.717185415940509	0.0677296290337204	KCTD21-AS1	100289388	KCTD21 antisense RNA 1 [Source:HGNC Symbol;Acc:HGNC:48674]		
0.716732171702644	0.0465523133034053	FAM182B	100132948	family with sequence similarity 182, member B [Source:HGNC Symbol;Acc:HGNC:34503]		
0.714479658890528	0.0329387440424469	CXCR5	643	chemokine (C-X-C motif) receptor 5 [Source:HGNC Symbol;Acc:HGNC:1060]	cxc13 receptor	
0.714075195840959	0.000428083131585236	KRTAP12-4	386684	keratin associated protein 12-4 [Source:HGNC Symbol;Acc:HGNC:20532]		
0.70847043781097	0.0872524114997303	MKI67	4288	marker of proliferation Ki-67 [Source:HGNC Symbol;Acc:HGNC:7107]		
0.707967488783195	0.161647326432689	UBD	10537	ubiquitin D [Source:HGNC Symbol;Acc:HGNC:18795]		
0.693316315047072	0.10764498508904		196549			
0.691881071100415	0.112565270194905	ACKR4	51554	atypical chemokine receptor 4 [Source:HGNC Symbol;Acc:HGNC:1611]		
0.691591991127043	0.109267266975618	HIST1H4E	8359	histone cluster 1, H4e [Source:HGNC Symbol;Acc:HGNC:4790]		
0.688764264883051	0.0367858612423791	HIST1H2BB	3018	histone cluster 1, H2bb [Source:HGNC Symbol;Acc:HGNC:4751]		
0.683716956238685	0.149557533962872	ALPP	250	alkaline phosphatase, placental [Source:HGNC Symbol;Acc:HGNC:439]		
0.678421558449322	0.0402634884782849	MTHFD1L	25902	methylenetetrahydrofolate dehydrogenase (NADP+ dependent) 1-like [Source:HGNC Symbol;Acc:HGNC:21055]		
0.675852968934389	0.189205692215678	GINS2	51659	GINS complex subunit 2 (Psf2 homolog) [Source:HGNC Symbol;Acc:HGNC:24575]		
0.674109337561377	0.00355057082476739	DEFB132	400830	defensin, beta 132 [Source:HGNC Symbol;Acc:HGNC:33806]	SNPs in hunter gatherers	
0.66770509911139	0.00125849313065779	KCNV1	27012	potassium channel, subfamily V, member 1 [Source:HGNC Symbol;Acc:HGNC:18861]		
0.666774959793796	2.2637732615476e-05	HEYL	26508	hes-related family bHLH transcription factor with YRPW motif-like [Source:HGNC Symbol;Acc:HGNC:4882]	Hairy/enhancer-of-split related with YRPW motif-lik	tumor supressor
0.664373983808938	0.16520787583937	CCDC183	84960	coiled-coil domain containing 183 [Source:HGNC Symbol;Acc:HGNC:28236]		
0.66007162706512	0.00168547403024986	SNORD24	26820	small nucleolar RNA, C/D box 24 [Source:HGNC Symbol;Acc:HGNC:10146]		
0.659245805150523	0.00431739092327972	CYP4Z1	199974	cytochrome P450, family 4, subfamily Z, polypeptide 1 [Source:HGNC Symbol;Acc:HGNC:205[illegible]	angiogeneisis ?	
0.655172745976188	0.0900284570365717	PAIP1	10605	poly(A) binding protein interacting protein 1 [Source:HGNC Symbol;Acc:HGNC:16945]		
0.653105031846669	0.00221966297057792	TRIM58	25893	tripartite motif containing 58 [Source:HGNC Symbol;Acc:HGNC:24150]		
0.652129691332437	0.18083888258803		400533	uncharacterized LOC400533 [Source:EntrezGene;Acc:400533]		
0.645458494888616	1.11584025097501e-05	C20orf27	54976	chromosome 20 open reading frame 27 [Source:HGNC Symbol;Acc:HGNC:15873]		
0.644299665156775	0.124785510584882	LINC01089	338799	long intergenic non-protein coding RNA 1089 [Source:HGNC Symbol;Acc:HGNC:27886]		
0.64256330564113	0.193835107120387		440173			
0.641868223964742	0.122364311089595		100996325			
0.639717187308726	0.124311298300497	MAGEB4	4115	melanoma antigen family B, 4 [Source:HGNC Symbol;Acc:HGNC:6811]		
0.634760312697862	0.000863723315644014	CLDN1	9076	claudin 1 [Source:HGNC Symbol;Acc:HGNC:2032]		
0.631994601160444	2.12564810870878e-05	C1orf159	54991	chromosome 1 open reading frame 159 [Source:HGNC Symbol;Acc:HGNC:26062]		
0.629230644611504	0.0320698311668783	MEG3	55384	maternally expressed 3 (non-protein coding) [Source:HGNC Symbol;Acc:HGNC:14575]		
0.625129994486655	0.0799132205956834	ITGA2	3673	integrin, alpha 2 (CD49B, alpha 2 subunit of VLA-2 receptor) [Source:HGNC Symbol;Acc:HGNC:6137]		
0.624909645978863	0.00381362230041927	GPR6	2830	G protein-coupled receptor 6 [Source:HGNC Symbol;Acc:HGNC:4515]		
0.624334081030718	0.121097008725026	VAMP1	6843	vesicle-associated membrane protein 1 (synaptobrevin 1) [Source:HGNC Symbol;Acc:HGNC:12642]		
0.623449371162344	0.00550843550949727	SNHG1	9298	small nucleolar RNA host gene 1 (non-protein coding) [Source:HGNC Symbol;Acc:HGNC:32688]		
0.617324482212691	0.0807880304531244	ZNF547	284306	zinc finger protein 547 [Source:HGNC Symbol;Acc:HGNC:26432]		
0.61710451823178	0.166161455576902	MAD2L1	4085	MAD2 mitotic arrest deficient-like 1 (yeast) [Source:HGNC Symbol;Acc:HGNC:6763]		
0.614293285124698	0.0126818960683828		80164	putative uncharacterized protein FLJ22184 [Source:EntrezGene;Acc:80164]		
0.612207784471106	0.00189145023465836	ENOX1	55068	ecto-NOX disulfide-thiol exchanger 1 [Source:HGNC Symbol;Acc:HGNC:25474]	NADH oxidase	
0.612022801453519	0.0027133746876292	MYL4	4635	myosin, light chain 4, alkali; atrial, embryonic [Source:HGNC Symbol;Acc:HGNC:7585]		
0.6099888312128	0.0485041454565628	KRTAP6-1	337966	keratin associated protein 6-1 [Source:HGNC Symbol;Acc:HGNC:18931]		
0.60980871658019	0.0480104572541123	FSIP2	401024	fibrous sheath interacting protein 2 [Source:HGNC Symbol;Acc:HGNC:21675]		
0.608316351133062	0.0930588104134937	C1QTNF9	338872	C1q and tumor necrosis factor related protein 9 [Source:HGNC Symbol;Acc:HGNC:28732]		
0.608247153566226	0.00060195987358099	AMER3	205147	APC membrane recruitment protein 3 [Source:HGNC Symbol;Acc:HGNC:26771]	activator wnt signallig	
0.607684930481913	0.0760508450699453	TRIM15	89870	tripartite motif containing 15 [Source:HGNC Symbol;Acc:HGNC:16284]		
0.606383776536366	0.106466220351563	UHRF1	29128	ubiquitin-like with PHD and ring finger domains 1 [Source:HGNC Symbol;Acc:HGNC:12556]		
0.600523573175638	0.119104069467201		100505502			
0.595668599678596	0.111362957837606	SNORD42A	26809	small nucleolar RNA, C/D box 42A [Source:HGNC Symbol;Acc:HGNC:10180]		
0.595109251602077	0.00420471428026255	DOC2A	8448	double C2-like domains, alpha [Source:HGNC Symbol;Acc:HGNC:2985]		
0.594856073118095	0.0475729251707252	IQGAP3	128239	IQ motif containing GTPase activating protein 3 [Source:HGNC Symbol;Acc:HGNC:20669]		
0.594105781695901	0.12380483627382	ACAP1	9744	ArfGAP with coiled-coil, ankyrin repeat and PH domains 1 [Source:HGNC Symbol;Acc:HGNC:16467]		
0.592934781851395	0.0608094052236626	NPPC	4880	natriuretic peptide C [Source:HGNC Symbol;Acc:HGNC:7941]		
0.592665848985363	0.00124422666380345	C7orf34	135927	chromosome 7 open reading frame 34 [Source:HGNC Symbol;Acc:HGNC:21750]		
0.592643714070476	5.91934573532838e-05	LPIN1	23175	lipin 1 [Source:HGNC Symbol;Acc:HGNC:13345]	TAG	
0.591470663843754	0.14031534211906	OR5K1	26339	olfactory receptor, family 5, subfamily K, member 1 [Source:HGNC Symbol;Acc:HGNC:8349]		
0.590909002113916	0.148702755924931		653160			
0.58874554620312	0.0245430188849134	LYPD5	284348	LY6/PLAUR domain containing 5 [Source:HGNC Symbol;Acc:HGNC:26397]		
0.588598195512655	0.0675409832376687	AURKB	9212	aurora kinase B [Source:HGNC Symbol;Acc:HGNC:11390]		
0.588490265301588	0.069919714401474	KRTAP10-1	386677	keratin associated protein 10-1 [Source:HGNC Symbol;Acc:HGNC:22966]		
0.585275562269433	0.146247747267484		100130370			
0.582058035290855	0.00209898835207963	GABRD	2563	gamma-aminobutyric acid (GABA) A receptor, delta [Source:HGNC Symbol;Acc:HGNC:4084]		
0.581927413967297	0.144394247702604	HMMR	3161	hyaluronan-mediated motility receptor (RHAMM) [Source:HGNC Symbol;Acc:HGNC:5012]		
0.580445423218876	0.0203755864393017	MYEOV	26579	myeloma overexpressed [Source:HGNC Symbol;Acc:HGNC:7563]		
0.580030424575493	0.120879752552187		56651			
0.579613668029515	0.0582151097665408	HAUS6	54801	HAUS augmin-like complex, subunit 6 [Source:HGNC Symbol;Acc:HGNC:25948]		
0.579015541827338	0.0121603357097377		54886	Lipid phosphate phosphatase-related protein type 1 [Source:UniProtKB/Swiss-Prot;Acc:Q8TBJ4]		
0.578457360085023	0.0686693852242589	NDC80	10403	NDC80 kinetochore complex component [Source:HGNC Symbol;Acc:HGNC:16909]		
0.577943414668505	0.0565386456778934		100133091			
0.577510692007006	0.157307127603601	HELLS	3070	helicase, lymphoid-specific [Source:HGNC Symbol;Acc:HGNC:4861]		
0.576089956575614	0.00810823326416891	TOPAZ1	375337	testis and ovary specific PAZ domain containing 1 [Source:HGNC Symbol;Acc:HGNC:24746]		
0.576078848157347	0.110960376959896	MEF2BNB	729991	MEF2B neighbor [Source:HGNC Symbol;Acc:HGNC:37247]		
0.575582700877061	0.187884219547231	OR10G4	390264	olfactory receptor, family 10, subfamily G, member 4 [Source:HGNC Symbol;Acc:HGNC:14809]		
0.573435760959257	0.0508008832876629	MCM3AP-AS	114044	MCM3AP antisense RNA 1 [Source:HGNC Symbol;Acc:HGNC:16417]		
0.569925641129642	0.0201187229875775	PABPC1L	80336	poly(A) binding protein, cytoplasmic 1-like [Source:HGNC Symbol;Acc:HGNC:15797]		
0.569865397111811	0.15769240749763	PYHIN1	149628	pyrin and HIN domain family, member 1 [Source:HGNC Symbol;Acc:HGNC:28894]		
0.569554766284193	9.04173371083862e-05	ZSCAN5B	342933	zinc finger and SCAN domain containing 5B [Source:HGNC Symbol;Acc:HGNC:34246]		
0.565224240025959	0.0106367626233061	HIST3H3	8290	histone cluster 3, H3 [Source:HGNC Symbol;Acc:HGNC:4778]		
0.565007496691488	0.00102165958282143		441728			
0.564730539906957	0.0332560267502583	MYBL2	4605	v-myb avian myeloblastosis viral oncogene homolog-like 2 [Source:HGNC Symbol;Acc:HGNC:7548]		
0.564622256146385	0.0372073113579228	SOX15	6665	SRY (sex determining region Y)-box 15 [Source:HGNC Symbol;Acc:HGNC:11196]		
0.563721949915235	0.0962495585133454	NNMT	101928916	nicotinamide N-methyltransferase [Source:HGNC Symbol;Acc:HGNC:7861]		
0.563395343982661	0.0331116813398304	P2RY4	5030	pyrimidinergic receptor P2Y, G-protein coupled, 4 [Source:HGNC Symbol;Acc:HGNC:8542]		
0.562731575299703	0.0501930137104163	FAM129C	199786	family with sequence similarity 129, member C [Source:HGNC Symbol;Acc:HGNC:24130]		
0.562666140319284	0.000140309349791276	INHBA	3624	inhibin, beta A [Source:HGNC Symbol;Acc:HGNC:6066]		
0.562367025195242	0.00336284123664016	XAGE2B	9502	X antigen family, member 2B [Source:HGNC Symbol;Acc:HGNC:30680]		
0.56141271523267	0.00388361524555507	ANKRD23	200539	ankyrin repeat domain 23 [Source:HGNC Symbol;Acc:HGNC:24470]		
0.560436781340485	0.0284935834647754	BCL2A1	597	BCL2-related protein A1 [Source:HGNC Symbol;Acc:HGNC:991]		
0.559087293431817	0.00219698246284309	NLRP9	338321	NLR family, pyrin domain containing 9 [Source:HGNC Symbol;Acc:HGNC:22941]		
0.557234505537416	0.0448067584085311		100507191			

0.556664645549388	0.0405860158462038	FSCB	84075	fibrous sheath CABYR binding protein [Source:HGNC Symbol;Acc:HGNC:20494]
0.556163160533934	0.147354107644229	NLRC5	84166	NLR family, CARD domain containing 5 [Source:HGNC Symbol;Acc:HGNC:29933]
0.555693759482368	0.0737068968545615	STARD4	134429	StAR-related lipid transfer (START) domain containing 4 [Source:HGNC Symbol;Acc:HGNC:18058]
0.555377786318476	0.171316858482328	MELK	9833	maternal embryonic leucine zipper kinase [Source:HGNC Symbol;Acc:HGNC:16870]
0.555350835641728	0.0873177778424158	RBAKDN	389458	RBAK downstream neighbor (non-protein coding) [Source:HGNC Symbol;Acc:HGNC:33770]
0.553954333744857	0.00204562588249337	RNU6-76P	100873777	RNA, U6 small nuclear 76, pseudogene [Source:HGNC Symbol;Acc:HGNC:42566]
0.553728879905749	0.0722730303982464	SNORA75	654321	small nucleolar RNA, H/ACA box 75 [Source:HGNC Symbol;Acc:HGNC:32661]
0.553615635405536	0.0816569506332994	HTR3A	3359	5-hydroxytryptamine (serotonin) receptor 3A, ionotropic [Source:HGNC Symbol;Acc:HGNC:5297]
0.553571649650228	0.0699275701005849	GINS3	64785	GINS complex subunit 3 (Psf3 homolog) [Source:HGNC Symbol;Acc:HGNC:25851]
0.55298999454426	0.0654660452154971	OR5M9	390162	olfactory receptor, family 5, subfamily M, member 9 [Source:HGNC Symbol;Acc:HGNC:15294]
0.552529680504135	0.0844191179235227	PCSK9	255738	proprotein convertase subtilisin/kexin type 9 [Source:HGNC Symbol;Acc:HGNC:20001]
0.55189672260305	0.0312221449886206	ZBP1	81030	Z-DNA binding protein 1 [Source:HGNC Symbol;Acc:HGNC:16176]
0.549345453430089	0.128583212130403	IL2RA	3559	interleukin 2 receptor, alpha [Source:HGNC Symbol;Acc:HGNC:6008]
0.549229124118227	0.186980554178737	PER3	8863	period circadian clock 3 [Source:HGNC Symbol;Acc:HGNC:8847]
0.54719226093985	0.00286535559022294	C15orf37	283687	chromosome 15 open reading frame 37 [Source:HGNC Symbol;Acc:HGNC:27521]
0.547047130143591	0.00502633644586692	KCNA1	3736	potassium voltage-gated channel, shaker-related subfamily, member 1 (episodic ataxia with myokymia) [Source:HGNC Symbol;Acc:HGNC:6218]
0.546802278920303	0.0356356333572679	DUSP2	1844	dual specificity phosphatase 2 [Source:HGNC Symbol;Acc:HGNC:3068]
0.545517935341186	0.0115267816433611	MIR196A1	406972	microRNA 196a-1 [Source:HGNC Symbol;Acc:HGNC:31567]
0.544907326130367	0.0763949735072645	TICRR	90381	TOPBP1-interacting checkpoint and replication regulator [Source:HGNC Symbol;Acc:HGNC:28704]
0.544183345912589	0.000370937594139591	STRIP2	57464	striatin interacting protein 2 [Source:HGNC Symbol;Acc:HGNC:22209]
0.543194027874735	0.0182925446135911	SNORA45B	677826	small nucleolar RNA, H/ACA box 45B [Source:HGNC Symbol;Acc:HGNC:32638]
0.542053131064277	0.00863398152523515	PRSS50	29122	protease, serine, 50 [Source:HGNC Symbol;Acc:HGNC:17910]
0.541345925198831	0.00107336280867452	FBF1	85302	Fas (TNFRSF6) binding factor 1 [Source:HGNC Symbol;Acc:HGNC:24674]
0.538831182135347	0.109033064654085	LINC00893	100131434	long intergenic non-protein coding RNA 893 [Source:HGNC Symbol;Acc:HGNC:44265]
0.53860075491964	0.0202165422415355	SEMG1	6406	semenogelin I [Source:HGNC Symbol;Acc:HGNC:10742]
0.535912719057156	0.0158012754878103		619498	
0.535507564297038	0.0688077279504816	SFTPC	6440	surfactant protein C [Source:HGNC Symbol;Acc:HGNC:10802]
0.535052685478794	0.0406783215288207		28673	
0.534561076046012	0.0186864967434864	FCRL4	83417	Fc receptor-like 4 [Source:HGNC Symbol;Acc:HGNC:18507]
0.534466439849289	0.0701307086462866	EIF5A	1984	eukaryotic translation initiation factor 5A [Source:HGNC Symbol;Acc:HGNC:3300]
0.533991015599079	0.0506945043783565	KIAA0226L	80183	KIAA0226-like [Source:HGNC Symbol;Acc:HGNC:20420]
0.533199505372642	0.000450091554381135	LINC00028	140875	long intergenic non-protein coding RNA 28 [Source:HGNC Symbol;Acc:HGNC:16224]
0.533047679404365	0.17851575873925		9560	C-C motif chemokine 4-like [Source:UniProtKB/Swiss-Prot;Acc:Q8NHW4]
0.532606644349686	0.0141957637554494	MIR6080	146880	microRNA 6080 [Source:HGNC Symbol;Acc:HGNC:50142]
0.532508504751171	0.00743013624683804	BMX	660	BMX non-receptor tyrosine kinase [Source:HGNC Symbol;Acc:HGNC:1079]
0.532131080308246	0.0630646351412252	RBL1	5933	retinoblastoma-like 1 [Source:HGNC Symbol;Acc:HGNC:9893]
0.531301359283505	0.025973645371131	GRAP2	9402	GRB2-related adaptor protein 2 [Source:HGNC Symbol;Acc:HGNC:4563]
0.53047984315144	0.0662048225180786	SNHG16	677850	small nucleolar RNA host gene 16 (non-protein coding) [Source:HGNC Symbol;Acc:HGNC:44352]
0.529839162351425	0.00351690606510244	DLL3	10683	delta-like 3 (Drosophila) [Source:HGNC Symbol;Acc:HGNC:2909]
0.529266830314217	0.0260621016253724	NANOS2	339345	nanos homolog 2 (Drosophila) [Source:HGNC Symbol;Acc:HGNC:23292]
0.52875698512005	0.012891833555507	NEUROG3	50674	neurogenin 3 [Source:HGNC Symbol;Acc:HGNC:13806]
0.52793692573298	0.0554238568955084	C5orf55	116349	chromosome 5 open reading frame 55 [Source:HGNC Symbol;Acc:HGNC:25175]
0.526918459541598	0.14492114823924	RRM2	6241	ribonucleotide reductase M2 [Source:HGNC Symbol;Acc:HGNC:10452]
0.526278093685549	0.0130621264939066	MRGPRD	116512	MAS-related GPR, member D [Source:HGNC Symbol;Acc:HGNC:29626]
0.525847078030828	0.138685489222364	CDC20	991	cell division cycle 20 [Source:HGNC Symbol;Acc:HGNC:1723]
0.525358360864293	0.161980569086124		102723566	
0.525337571573691	1.07976300935264e-05	PSTPIP1	9051	proline-serine-threonine phosphatase interacting protein 1 [Source:HGNC Symbol;Acc:HGNC:9580]
0.524743999977634	0.0021124305431454	ZNF416	55659	zinc finger protein 416 [Source:HGNC Symbol;Acc:HGNC:20645]
0.52438521351465	0.162363364810921	C12orf29	91298	chromosome 12 open reading frame 29 [Source:HGNC Symbol;Acc:HGNC:25322]
0.522484697220054	0.0774218125277422	RNU6-63P	100873768	RNA, U6 small nuclear 63, pseudogene [Source:HGNC Symbol;Acc:HGNC:42553]
0.522380131105855	0.170994862534341	ZNF204P	7754	zinc finger protein 204, pseudogene [Source:HGNC Symbol;Acc:HGNC:12995]
0.522136751834596	0.0540762462445645	TYW1	55253	tRNA-yW synthesizing protein 1 homolog (S. cerevisiae) [Source:HGNC Symbol;Acc:HGNC:25598]
0.522041289392105	0.085670953185961	SLC19A1	6573	solute carrier family 19 (folate transporter), member 1 [Source:HGNC Symbol;Acc:HGNC:10937]
0.521495525194651	0.173912504905333	KIF11	3832	kinesin family member 11 [Source:HGNC Symbol;Acc:HGNC:6388]
0.521486057374749	0.0774161045411978	PSMG4	389362	proteasome (prosome, macropain) assembly chaperone 4 [Source:HGNC Symbol;Acc:HGNC:21108]
0.520393039279903	0.000593310627626501		101928612	
0.519753170863211	0.139948600711447	IDI1	3422	isopentenyl-diphosphate delta isomerase 1 [Source:HGNC Symbol;Acc:HGNC:5387]
0.519650667989476	0.169935627608411	KIF14	9928	kinesin family member 14 [Source:HGNC Symbol;Acc:HGNC:19181]
0.518818408875814	0.0442834368421291	RTP4	64108	receptor (chemosensory) transporter protein 4 [Source:HGNC Symbol;Acc:HGNC:23992]
0.518775773898276	0.159076228331278	VRK1	7443	vaccinia related kinase 1 [Source:HGNC Symbol;Acc:HGNC:12718]
0.51784427559401	0.00656615831192614	RPH3AL	9501	rabphilin 3A-like (without C2 domains) [Source:HGNC Symbol;Acc:HGNC:10296]
0.517760698284035	0.0430106282002825	MARCO	8685	macrophage receptor with collagenous structure [Source:HGNC Symbol;Acc:HGNC:6895]
0.517390827494028	0.000402178186347339	CCNF	899	cyclin F [Source:HGNC Symbol;Acc:HGNC:1591]
0.516724097414473	6.02971020002625e-05	LOR	4014	loricrin [Source:HGNC Symbol;Acc:HGNC:6663]
0.515819338781935	0.00209199536899065	PRRT2	112476	proline-rich transmembrane protein 2 [Source:HGNC Symbol;Acc:HGNC:30500]
0.515505565335265	0.00135158334293844	RAMP1	10267	receptor (G protein-coupled) activity modifying protein 1 [Source:HGNC Symbol;Acc:HGNC:9843]
0.515448388353193	0.0434319761581895	NXPH3	11248	neurexophilin 3 [Source:HGNC Symbol;Acc:HGNC:8077]
0.515384700477445	0.00755754278938333	ATP6V1C2	245973	ATPase, H+ transporting, lysosomal 42kDa, V1 subunit C2 [Source:HGNC Symbol;Acc:HGNC:18264]
0.514760662449278	0.112768786718735	ZNF671	79891	zinc finger protein 671 [Source:HGNC Symbol;Acc:HGNC:26279]
0.514063183379181	0.0373990777860485	PDE9A	5152	phosphodiesterase 9A [Source:HGNC Symbol;Acc:HGNC:8795]
0.513476930599065	0.00242491205882552	DHX34	9704	DEAH (Asp-Glu-Ala-His) box polypeptide 34 [Source:HGNC Symbol;Acc:HGNC:16719]
0.513298996393482	0.0854892244529127		254896	
0.512780659347746	0.0218589014161569	SHISA2	387914	shisa family member 2 [Source:HGNC Symbol;Acc:HGNC:20366]
0.511235688172878	0.181456403932269	CYP24A1	1591	cytochrome P450, family 24, subfamily A, polypeptide 1 [Source:HGNC Symbol;Acc:HGNC:2602]
0.511008154201056	0.0396190475992305		155060	
0.510835800220802	0.0151722267216752	C1orf189	388701	chromosome 1 open reading frame 189 [Source:HGNC Symbol;Acc:HGNC:32305]
0.510642756072952	0.0252672902579652	TCTE3	6991	t-complex-associated-testis-expressed 3 [Source:HGNC Symbol;Acc:HGNC:11695]
0.509425522860298	0.000394063049453654	GPC2	221914	glypican 2 [Source:HGNC Symbol;Acc:HGNC:4450]
0.509304538307023	0.0108196667294159	LCN6	158062	lipocalin 6 [Source:HGNC Symbol;Acc:HGNC:17337]
0.509207758803911	0.000382370589522407	GPR150	285601	G protein-coupled receptor 150 [Source:HGNC Symbol;Acc:HGNC:23628]
0.509164996194329	0.0293834395495483	EPS8L1	54869	EPS8-like 1 [Source:HGNC Symbol;Acc:HGNC:21295]
0.509142623345757	0.0125631202867052	GNG8	94235	guanine nucleotide binding protein (G protein), gamma 8 [Source:HGNC Symbol;Acc:HGNC:19664]
0.508469559439811	0.0193364338215823	GPR97	222487	G protein-coupled receptor 97 [Source:HGNC Symbol;Acc:HGNC:13728]
0.508330468187074	0.0175495637037203	MYBPH	4608	myosin binding protein H [Source:HGNC Symbol;Acc:HGNC:7552]
0.508105583443442	0.119415792750545	PSMB9	5698	proteasome (prosome, macropain) subunit, beta type, 9 [Source:HGNC Symbol;Acc:HGNC:9546]
0.507674940845967	0.0788717568767038	IL13RA2	3598	interleukin 13 receptor, alpha 2 [Source:HGNC Symbol;Acc:HGNC:5975]
0.50652157573705	0.191225919632952	MCM10	55388	minichromosome maintenance complex component 10 [Source:HGNC Symbol;Acc:HGNC:18043]
0.505295811716464	0.125790077066438	RFC2	5982	replication factor C (activator 1) 2, 40kDa [Source:HGNC Symbol;Acc:HGNC:9970]
0.505108836459836	0.0588489144151853		100130771	
0.504669503410812	0.0992735555380206	LIPM	340654	lipase, family member M [Source:HGNC Symbol;Acc:HGNC:23455]
0.503786412982647	0.0373487439206838	TNF	7124	tumor necrosis factor [Source:HGNC Symbol;Acc:HGNC:11892]
0.503092065512904	0.137433632754386		284802	
0.50202914220259	0.0023991419735088	C2orf48	348738	chromosome 2 open reading frame 48 [Source:HGNC Symbol;Acc:HGNC:26322]
0.501671558198009	0.112386556368962	BUB1B	701	BUB1 mitotic checkpoint serine/threonine kinase B [Source:HGNC Symbol;Acc:HGNC:1149]
0.500666056463223	0.0265856824572132	IL11	3589	interleukin 11 [Source:HGNC Symbol;Acc:HGNC:5966]
0.500575329084678	0.0107760418238536	APOBEC3A	200315	apolipoprotein B mRNA editing enzyme, catalytic polypeptide-like 3A [Source:HGNC Symbol;Acc:HGNC:17343]
0.500215230329875	0.00750417570371735	TBX20	57057	T-box 20 [Source:HGNC Symbol;Acc:HGNC:11598]
0.499713493043669	0.0341609773116472	MOBP	4336	myelin-associated oligodendrocyte basic protein [Source:HGNC Symbol;Acc:HGNC:7189]
0.499705588977016	0.0568425464771669	SNORD4B	26772	small nucleolar RNA, C/D box 4B [Source:HGNC Symbol;Acc:HGNC:10098]
0.499649952521847	0.174231142993444	GBA3	57733	glucosidase, beta, acid 3 (gene/pseudogene) [Source:HGNC Symbol;Acc:HGNC:19069]
0.499197676552609	0.114938703023658		646813	
0.498866475219203	0.0330990098236284	KCNG1	3755	potassium voltage-gated channel, subfamily G, member 1 [Source:HGNC Symbol;Acc:HGNC:6248]
0.498770295164794	0.0763821898872555	TSPYL4	23270	TSPY-like 4 [Source:HGNC Symbol;Acc:HGNC:21559]
0.497630970536322	0.0138079631518141	OR6Q1	219952	olfactory receptor, family 6, subfamily Q, member 1 [Source:HGNC Symbol;Acc:HGNC:15302]
0.496973453151697	0.00422214036376295	SEZ6	124925	seizure related 6 homolog (mouse) [Source:HGNC Symbol;Acc:HGNC:15955]
0.496330946906359	0.01561349868356	SOX18	54345	SRY (sex determining region Y)-box 18 [Source:HGNC Symbol;Acc:HGNC:11194]
0.495922590325097	0.044060263949045	OR51I1	390063	olfactory receptor, family 51, subfamily I, member 1 [Source:HGNC Symbol;Acc:HGNC:15200]
0.495595670665863	0.00167061222875999	ZNF668	79759	zinc finger protein 668 [Source:HGNC Symbol;Acc:HGNC:25821]
0.495266451578074	0.0494058352913246	GFAP	2670	glial fibrillary acidic protein [Source:HGNC Symbol;Acc:HGNC:4235]
0.494846645438606	0.0042152534528467	TUBBP5	643224	tubulin, beta pseudogene 5 [Source:HGNC Symbol;Acc:HGNC:23674]
0.494741987449861	0.0961836426608558	MIR185	406961	microRNA 185 [Source:HGNC Symbol;Acc:HGNC:31556]
0.494289888754478	0.189193591521925	SLC10A2	6555	solute carrier family 10 (sodium/bile acid cotransporter), member 2 [Source:HGNC Symbol;Acc:HGNC:10906]
0.493833111318045	0.0450945097709499		645769	
0.493730383453059	0.00596687939843079	GAS2L2	246176	growth arrest-specific 2 like 2 [Source:HGNC Symbol;Acc:HGNC:24846]
0.492907412761159	0.135520434255247	CMPK2	129607	cytidine monophosphate (UMP-CMP) kinase 2, mitochondrial [Source:HGNC Symbol;Acc:HGNC:27015]
0.492300354278079	0.0124882129876895	MYF5	4617	myogenic factor 5 [Source:HGNC Symbol;Acc:HGNC:7565]
0.492300009835892	0.0455057032753332	OR52B2	255725	olfactory receptor, family 52, subfamily B, member 2 [Source:HGNC Symbol;Acc:HGNC:15207]
0.491138007408765	0.0470362139503048	RTN2	6253	reticulon 2 [Source:HGNC Symbol;Acc:HGNC:10468]
0.49044623144868	0.0247864617271008	C15orf52	388115	chromosome 15 open reading frame 52 [Source:HGNC Symbol;Acc:HGNC:33488]
0.490022315238265	0.0717321731884613	LINC00339	29092	long intergenic non-protein coding RNA 339 [Source:HGNC Symbol;Acc:HGNC:25011]
0.48970622619799	0.128663028179765	TRIM48	79097	tripartite motif containing 48 [Source:HGNC Symbol;Acc:HGNC:19021]
0.489118689357482	0.0270611228476724	ZDHHC8P1	150244	zinc finger, DHHC-type containing 8 pseudogene 1 [Source:HGNC Symbol;Acc:HGNC:26461]
0.488754600091098	0.139412197553559	SNORA61	677838	small nucleolar RNA, H/ACA box 61 [Source:HGNC Symbol;Acc:HGNC:32655]
0.487749013689734	0.0872707262607794		642648	
0.48736811051998	0.109972848487959	SCARNA8	677776	small Cajal body-specific RNA 8 [Source:HGNC Symbol;Acc:HGNC:32564]
0.487294517452202	0.0197387002784052	ZIC5	85416	Zic family member 5 [Source:HGNC Symbol;Acc:HGNC:20322]
0.486968478146476	0.0229063712014537	REEP2	51308	receptor accessory protein 2 [Source:HGNC Symbol;Acc:HGNC:17975]
0.486655527244397	0.0262584199912809	FGR	2268	FGR proto-oncogene, Src family tyrosine kinase [Source:HGNC Symbol;Acc:HGNC:3697]
0.486420934069746	0.078482100680471	HIST1H3D	8358	histone cluster 1, H3d [Source:HGNC Symbol;Acc:HGNC:4767]
0.485511331191158	0.0240888144541981	REC8	9985	REC8 meiotic recombination protein [Source:HGNC Symbol;Acc:HGNC:16879]
0.485185078920401	0.116428843085686	CA5BP1	340591	carbonic anhydrase VB pseudogene 1 [Source:HGNC Symbol;Acc:HGNC:29544]
0.484746633564657	0.0642689261224781	ESPL1	9700	extra spindle pole bodies homolog 1 (S. cerevisiae) [Source:HGNC Symbol;Acc:HGNC:16856]
0.484495357731693	0.0780776969086427	E2F8	79733	E2F transcription factor 8 [Source:HGNC Symbol;Acc:HGNC:24727]
0.484282763916834	0.00088161047208087	GPR179	440435	G protein-coupled receptor 179 [Source:HGNC Symbol;Acc:HGNC:31371]
0.482892349673126	0.0183763845246442	TFAP2A-AS1	100130275	TFAP2A antisense RNA 1 [Source:HGNC Symbol;Acc:HGNC:40579]
0.482860610012865	0.133503663720359	ADAMTS7	11173	ADAM metallopeptidase with thrombospondin type 1 motif, 7 [Source:HGNC Symbol;Acc:HGNC:223]
0.48283992313574	0.0368151162008052	TBX15	6913	T-box 15 [Source:HGNC Symbol;Acc:HGNC:11594]
0.482401907648897	0.165043921931421		100526833	
0.482139938881389	0.0301423639307272	CAMK2N2	94032	calcium/calmodulin-dependent protein kinase II inhibitor 2 [Source:HGNC Symbol;Acc:HGNC:24197]
0.481262873846022	0.101542060507876	PSORS1C1	170679	psoriasis susceptibility 1 candidate 1 [Source:HGNC Symbol;Acc:HGNC:17202]
0.481128825642821	0.00185594400311189	LINC00242	401288	long intergenic non-protein coding RNA 242 [Source:HGNC Symbol;Acc:HGNC:21249]

0.481061756074431	0.0112854923309257	ASPHD1	253982	aspartate beta-hydroxylase domain containing 1 [Source:HGNC Symbol;Acc:HGNC:27380]
0.48080609166649	0.0021069662893473	PRM1	5619	protamine 1 [Source:HGNC Symbol;Acc:HGNC:9447]
0.480428144654472	0.0570337165609847	HIST1H2AI	8330	histone cluster 1, H2ai [Source:HGNC Symbol;Acc:HGNC:4725]
0.480164059064299	0.0822389625487506	SLC35F1	222553	solute carrier family 35, member F1 [Source:HGNC Symbol;Acc:HGNC:21483]
0.479839507312587	0.118930874264288	SCUBE2	57758	signal peptide, CUB domain, EGF-like 2 [Source:HGNC Symbol;Acc:HGNC:30425]
0.479570917407667	0.0525618372012035	TREML3P	340206	triggering receptor expressed on myeloid cells-like 3, pseudogene [Source:HGNC Symbol;Acc:HGNC:30806]
0.479294764541477	0.107556510436641	AHRR	57491	aryl-hydrocarbon receptor repressor [Source:HGNC Symbol;Acc:HGNC:346]
0.479210657588289	0.0887373853641026	SUGCT	79783	succinyl-CoA:glutarate-CoA transferase [Source:HGNC Symbol;Acc:HGNC:16001]
0.478797147581125	0.0742067000927862	NTF4	4909	neurotrophin 4 [Source:HGNC Symbol;Acc:HGNC:8024]
0.478671187517793	0.0331012074941468	DFFA	1676	DNA fragmentation factor, 45kDa, alpha polypeptide [Source:HGNC Symbol;Acc:HGNC:2772]
0.478317774331188	0.0765144024667227	GDPGP1	390637	GDP-D-glucose phosphorylase 1 [Source:HGNC Symbol;Acc:HGNC:34360]
0.477717926384068	0.0830160582695875	KLK13	26085	kallikrein-related peptidase 13 [Source:HGNC Symbol;Acc:HGNC:6361]
0.476663555334676	0.00721374354820325	RAMP2	10266	receptor (G protein-coupled) activity modifying protein 2 [Source:HGNC Symbol;Acc:HGNC:9844]
0.475204134416864	0.00151777480946624	C8G	733	complement component 8, gamma polypeptide [Source:HGNC Symbol;Acc:HGNC:1354]
0.475004402144062	0.00585473811994816	TMEM143	55260	transmembrane protein 143 [Source:HGNC Symbol;Acc:HGNC:25603]
0.474358727763703	0.0180666191380472	VARS2	57176	valyl-tRNA synthetase 2, mitochondrial [Source:HGNC Symbol;Acc:HGNC:21642]
0.473709838974159	0.0886123957275821	AASS	10157	aminoadipate-semialdehyde synthase [Source:HGNC Symbol;Acc:HGNC:17366]
0.473492137885268	0.0529682044717553	TIAF1	9220	TGFB1-induced anti-apoptotic factor 1 [Source:HGNC Symbol;Acc:HGNC:11803]
0.472924767306809	0.0101879173317748	EGFL7	51162	EGF-like-domain, multiple 7 [Source:HGNC Symbol;Acc:HGNC:20594]
0.472572256948976	0.00350955175572199	RINL	126432	Ras and Rab interactor-like [Source:HGNC Symbol;Acc:HGNC:24795]
0.472490217212948	0.082767674035608	OR2M7	391196	olfactory receptor, family 2, subfamily M, member 7 [Source:HGNC Symbol;Acc:HGNC:19594]
0.471553205232754	0.013139052604244	CALCB	797	calcitonin-related polypeptide beta [Source:HGNC Symbol;Acc:HGNC:1438]
0.471457638092053	0.174936508435494	KIR2DL4	3805	killer cell immunoglobulin-like receptor, two domains, long cytoplasmic tail, 4 [Source:HGNC Symbol;Acc:HGNC:6332]
0.471450952142543	0.0149271068199807	CD1A	909	CD1a molecule [Source:HGNC Symbol;Acc:HGNC:1634]
0.4710157369026	0.0319871056936898	SLC2A5	6518	solute carrier family 2 (facilitated glucose/fructose transporter), member 5 [Source:HGNC Symbol;Acc:HGNC:11010]
0.470829505075445	0.152058517557475	NCAPG	64151	non-SMC condensin I complex, subunit G [Source:HGNC Symbol;Acc:HGNC:24304]
0.470762415181006	0.0106332199448436	OR11L1	391189	olfactory receptor, family 11, subfamily L, member 1 [Source:HGNC Symbol;Acc:HGNC:14998]
0.470498186684829	0.0505804055604571		400658	uncharacterized protein LOC400658 [Source:RefSeq peptide;Acc:NP_001259022]
0.469845746945144	0.0439597844558943	FUZ	80199	fuzzy planar cell polarity protein [Source:HGNC Symbol;Acc:HGNC:26219]
0.469361666755719	0.165792244554495	ASF1B	55723	anti-silencing function 1B histone chaperone [Source:HGNC Symbol;Acc:HGNC:20996]
0.469276342480775	0.0872685081456982	PSCA	8000	prostate stem cell antigen [Source:HGNC Symbol;Acc:HGNC:9500]
0.468578757827134	0.165273372254732	AZIN2	113451	antizyme inhibitor 2 [Source:HGNC Symbol;Acc:HGNC:29957]
0.467417859907086	0.135110144462777	CHAF1B	8208	chromatin assembly factor 1, subunit B (p60) [Source:HGNC Symbol;Acc:HGNC:1911]
0.466680755900102	0.0401062561397047	RNU105C	26766	RNA, U105C small nucleolar [Source:HGNC Symbol;Acc:HGNC:10104]
0.466427466129519	0.135395093357118	UBE2L6	9246	ubiquitin-conjugating enzyme E2L 6 [Source:HGNC Symbol;Acc:HGNC:12490]
0.46618717796234	0.00870252610860392	BPESC1	60467	blepharophimosis, epicanthus inversus and ptosis, candidate 1 (non-protein coding) [Source:HGNC Symbol;Acc:HGNC:13228]
0.465902292793546	0.185819472658171	OIP5	11339	Opa interacting protein 5 [Source:HGNC Symbol;Acc:HGNC:20300]
0.465569824697377	0.0360401070071004	FAIM	55179	Fas apoptotic inhibitory molecule [Source:HGNC Symbol;Acc:HGNC:18703]
0.465501121056185	0.0456088252450927	TSKS	60385	testis-specific serine kinase substrate [Source:HGNC Symbol;Acc:HGNC:30719]
0.465484442632752	0.00265640540179027	ARMC5	79798	armadillo repeat containing 5 [Source:HGNC Symbol;Acc:HGNC:25781]
0.465466893211805	0.0913678155130273	NOP58	51602	NOP58 ribonucleoprotein [Source:HGNC Symbol;Acc:HGNC:29926]
0.464957417349555	0.0614417073812529	MIRLET7G	406890	microRNA let-7g [Source:HGNC Symbol;Acc:HGNC:31485]
0.464268327235011	0.0272220373065574	OLIG1	116448	oligodendrocyte transcription factor 1 [Source:HGNC Symbol;Acc:HGNC:16983]
0.463767134934269	0.0122542842972224	GJD4	219770	gap junction protein, delta 4, 40.1kDa [Source:HGNC Symbol;Acc:HGNC:23296]
0.462827624793355	0.0202504494935405	DKKL1	27120	dickkopf-like 1 [Source:HGNC Symbol;Acc:HGNC:16528]
0.462539489879511	0.186583537108863	KCNJ12	3768	potassium inwardly-rectifying channel, subfamily J, member 12 [Source:HGNC Symbol;Acc:HGNC:6258]
0.462006062394018	0.112209028379428	MIR202	574448	microRNA 202 [Source:HGNC Symbol;Acc:HGNC:32080]
0.461584758767508	0.00204370707364024	NFKBID	84807	nuclear factor of kappa light polypeptide gene enhancer in B-cells inhibitor, delta [Source:HGNC Symbol;Acc:HGNC:15671]
0.461168530023214	0.130015775695007	HIST1H2AJ	8331	histone cluster 1, H2aj [Source:HGNC Symbol;Acc:HGNC:4727]
0.461164202592954	0.00178416140962927	ARR3	407	arrestin 3, retinal (X-arrestin) [Source:HGNC Symbol;Acc:HGNC:710]
0.461127009715855	0.00108622934131168		645188	
0.460701008310803	0.0374644890320074	CSAG1	158511	chondrosarcoma associated gene 1 [Source:HGNC Symbol;Acc:HGNC:24294]
0.460360759196135	0.0327783655509119	CNR2	1269	cannabinoid receptor 2 (macrophage) [Source:HGNC Symbol;Acc:HGNC:2160]
0.460334187116011	0.000256792766919273	TUBB8	347688	tubulin, beta 8 class VIII [Source:HGNC Symbol;Acc:HGNC:20773]
0.460211470343404	0.164821113068562	OR10K2	391107	olfactory receptor, family 10, subfamily K, member 2 [Source:HGNC Symbol;Acc:HGNC:14826]
0.460172316905271	0.0236352114112414	CARD14	79092	caspase recruitment domain family, member 14 [Source:HGNC Symbol;Acc:HGNC:16446]
0.460129292329286	0.063960904896013	CELF5	60680	CUGBP, Elav-like family member 5 [Source:HGNC Symbol;Acc:HGNC:14058]
0.459983177583367	0.01174086337301	NUDT17	200035	nudix (nucleoside diphosphate linked moiety X)-type motif 17 [Source:HGNC Symbol;Acc:HGNC:26618]
0.459665014141461	0.140696203160606		202181	
0.459630258160067	0.0732942375948756	ZSCAN22	342945	zinc finger and SCAN domain containing 22 [Source:HGNC Symbol;Acc:HGNC:4929]
0.45947144573099	0.164201147942085	BCL6	604	B-cell CLL/lymphoma 6 [Source:HGNC Symbol;Acc:HGNC:1001]
0.45945036745513	1.76233109886796e-05	GZMM	3004	granzyme M (lymphocyte met-ase 1) [Source:HGNC Symbol;Acc:HGNC:4712]
0.458971397802389	0.156437357846772	ARG2	384	arginase 2 [Source:HGNC Symbol;Acc:HGNC:664]
0.458951269177059	0.00987776877956408	CARNS1	57571	carnosine synthase 1 [Source:HGNC Symbol;Acc:HGNC:29268]
0.458830225918534	0.0343088452466232	ZNF689	115509	zinc finger protein 689 [Source:HGNC Symbol;Acc:HGNC:25173]
0.458681368185944	0.00179035178998425	FEZF1	389549	FEZ family zinc finger 1 [Source:HGNC Symbol;Acc:HGNC:22788]
0.458582188255764	0.00366646660947725	RELT	84957	RELT tumor necrosis factor receptor [Source:HGNC Symbol;Acc:HGNC:13764]
0.458546648480726	0.178719741797132	KCNJ14	3770	potassium inwardly-rectifying channel, subfamily J, member 14 [Source:HGNC Symbol;Acc:HGNC:6260]
0.457933267800229	0.0218435438327328	HP	3240	haptoglobin [Source:HGNC Symbol;Acc:HGNC:5141]
0.457011387447677	0.129761037738799	NEIL3	55247	nei endonuclease VIII-like 3 (E. coli) [Source:HGNC Symbol;Acc:HGNC:24573]
0.456782503599411	0.036402786770162	DMRTC2	63946	DMRT-like family C2 [Source:HGNC Symbol;Acc:HGNC:13911]
0.456614128229687	0.167076004246827	MGAT4EP	641515	MGAT4 family, member E, pseudogene [Source:HGNC Symbol;Acc:HGNC:49418]
0.456512356122801	0.0556186553055639	TMEM92	162461	transmembrane protein 92 [Source:HGNC Symbol;Acc:HGNC:26579]
0.456406780750583	0.00907670041273699	SPEM1	374768	spermatid maturation 1 [Source:HGNC Symbol;Acc:HGNC:32429]
0.456386038908742	0.0654277682969358	LY6G5B	58496	lymphocyte antigen 6 complex, locus G5B [Source:HGNC Symbol;Acc:HGNC:13931]
0.456292149669418	0.0590790475515062	C16orf92	146378	chromosome 16 open reading frame 92 [Source:HGNC Symbol;Acc:HGNC:26346]
0.456052855659023	0.0121673726092539	FGFBP3	143282	fibroblast growth factor binding protein 3 [Source:HGNC Symbol;Acc:HGNC:23428]
0.455573883169341	0.0738811813587743	RLBP1	6017	retinaldehyde binding protein 1 [Source:HGNC Symbol;Acc:HGNC:10024]
0.455543097336042	0.0180492777135413	MIR132	406921	microRNA 132 [Source:HGNC Symbol;Acc:HGNC:31516]
0.455524835277491	0.0559635365787614	GBX2	2637	gastrulation brain homeobox 2 [Source:HGNC Symbol;Acc:HGNC:4186]
0.455492507579666	0.00413043169172022	ZCCHC4	29063	zinc finger, CCHC domain containing 4 [Source:HGNC Symbol;Acc:HGNC:22917]
0.455325255573451	0.0182796309684344	TRIM54	57159	tripartite motif containing 54 [Source:HGNC Symbol;Acc:HGNC:16008]
0.455230862194536	0.125980018366796	HIST1H4E	8368	histone cluster 1, H4e [Source:HGNC Symbol;Acc:HGNC:4790]
0.454455642076999	0.0691227566388519	LCE1F	353137	late cornified envelope 1F [Source:HGNC Symbol;Acc:HGNC:29467]
0.45343170178393	0.0676752102968446		643036	proteoglycan 3 pseudogene [Source:EntrezGene;Acc:643036]
0.45334143591223	0.0148879874366098	TFAP2E	339488	transcription factor AP-2 epsilon (activating enhancer binding protein 2 epsilon) [Source:HGNC Symbol;Acc:HGNC:30774]
0.45332485846519	0.0649751496670152	KRTAP12-1	353332	keratin associated protein 12-1 [Source:HGNC Symbol;Acc:HGNC:20529]
0.453305699294148	0.167788482842171	C2orf81	388963	chromosome 2 open reading frame 81 [Source:HGNC Symbol;Acc:HGNC:34350]
0.452577977556759	0.0567089102256569	AANAT	15	aralkylamine N-acetyltransferase [Source:HGNC Symbol;Acc:HGNC:19]
0.452379312574279	0.0742759583126271	KIAA1644	85352	KIAA1644 [Source:HGNC Symbol;Acc:HGNC:29335]
0.45227050065322	0.00482762763899438	SPATA3	130560	spermatogenesis associated 3 [Source:HGNC Symbol;Acc:HGNC:17884]
0.451441696157547	0.144892362954573	ZNF534	147658	zinc finger protein 534 [Source:HGNC Symbol;Acc:HGNC:26337]
0.451433061291719	0.0977379035813202	CHAC2	494143	ChaC, cation transport regulator homolog 2 (E. coli) [Source:HGNC Symbol;Acc:HGNC:32363]
0.451219608872411	0.000165317016269378	OR2Y1	134083	olfactory receptor, family 2, subfamily Y, member 1 [Source:HGNC Symbol;Acc:HGNC:14837]
0.451084951117448	0.0341459190075643	DUSP13	51207	dual specificity phosphatase 13 [Source:HGNC Symbol;Acc:HGNC:19681]
0.450939222386221	0.0145453516631581	HOGA1	112817	4-hydroxy-2-oxoglutarate aldolase 1 [Source:HGNC Symbol;Acc:HGNC:25155]
0.450895190629363	0.00224646253764195	CXCL3	2921	chemokine (C-X-C motif) ligand 3 [Source:HGNC Symbol;Acc:HGNC:4604]
0.450871461612169	0.0304814968126525		101928751	
0.450838530479484	0.059428224444711	STAC2	342667	SH3 and cysteine rich domain 2 [Source:HGNC Symbol;Acc:HGNC:23990]
0.450619255900106	0.121781387305848	SNORA80B	100302743	small nucleolar RNA, H/ACA box 80B [Source:HGNC Symbol;Acc:HGNC:34355]
0.450312137937904	0.10450128585092	TSPAN10	83882	tetraspanin 10 [Source:HGNC Symbol;Acc:HGNC:29942]
0.450289748319782	0.0108046845521825	CCDC172	374355	coiled-coil domain containing 172 [Source:HGNC Symbol;Acc:HGNC:30524]
0.449947853907095	0.0146862676756767	NAT14	57106	N-acetyltransferase 14 (GCN5-related, putative) [Source:HGNC Symbol;Acc:HGNC:28918]
0.449777767021513	0.0547189609821158	GLTSCR1	29998	glioma tumor suppressor candidate region gene 1 [Source:HGNC Symbol;Acc:HGNC:4332]
0.449581256243055	0.000567908454246453	C19orf57	79173	chromosome 19 open reading frame 57 [Source:HGNC Symbol;Acc:HGNC:28153]
0.449515917500187	0.122802105077801	OR10G7	390265	olfactory receptor, family 10, subfamily G, member 7 [Source:HGNC Symbol;Acc:HGNC:14842]
0.449332694953566	0.107570569205579	HAO2	51179	hydroxyacid oxidase 2 (long chain) [Source:HGNC Symbol;Acc:HGNC:4810]
0.449158109126059	0.0963427723587981	OR8D2	283160	olfactory receptor, family 8, subfamily D, member 2 [Source:HGNC Symbol;Acc:HGNC:8482]
0.449105857783277	0.00717506133299778	OR10H5	284433	olfactory receptor, family 10, subfamily H, member 5 [Source:HGNC Symbol;Acc:HGNC:15389]
0.449007724135499	0.0203103165468563	KLKP1	606293	kallikrein pseudogene 1 [Source:HGNC Symbol;Acc:HGNC:21260]
0.448953690147145	0.0551550925657279	METAP1D	254042	methionyl aminopeptidase type 1D (mitochondrial) [Source:HGNC Symbol;Acc:HGNC:32583]
0.448836249225616	0.019529173259448	KIR2DS4	3809	killer cell immunoglobulin-like receptor, two domains, short cytoplasmic tail, 4 [Source:HGNC Symbol;Acc:HGNC:6336]
0.448378162243283	0.0357016480919273	C15orf41	84529	chromosome 15 open reading frame 41 [Source:HGNC Symbol;Acc:HGNC:26929]
0.448179262645195	0.0283946304114335	NKX6-3	157848	NK6 homeobox 3 [Source:HGNC Symbol;Acc:HGNC:26328]
0.447981751632136	0.0678736540907547	CXCL11	6373	chemokine (C-X-C motif) ligand 11 [Source:HGNC Symbol;Acc:HGNC:10638]
0.447848866542721	0.0713712681808361	DPY19L2P4	442523	DPY19L2 pseudogene 4 [Source:HGNC Symbol;Acc:HGNC:22176]
0.447541609994014	0.0432548432827119	SLC7A5	8140	solute carrier family 7 (amino acid transporter light chain, L system), member 5 [Source:HGNC Symbol;Acc:HGNC:11063]
0.447396982520731	0.0233519384191111	C6orf25	80739	chromosome 6 open reading frame 25 [Source:HGNC Symbol;Acc:HGNC:13937]
0.447330370480198	0.0113599028881865	NCR1	9437	natural cytotoxicity triggering receptor 1 [Source:HGNC Symbol;Acc:HGNC:6731]
0.447201180387031	0.0465288104962425	SNORD46	94161	small nucleolar RNA, C/D box 46 [Source:HGNC Symbol;Acc:HGNC:10186]
0.447198249917552	0.0093462748203737	MAGEB17	645864	melanoma antigen family B, 17 [Source:HGNC Symbol;Acc:HGNC:17418]
0.446751405741309	0.0646833789311975	OR2T8	343172	olfactory receptor, family 2, subfamily T, member 8 [Source:HGNC Symbol;Acc:HGNC:15020]
0.446729017420971	0.00483955017303933	MIR184	406960	microRNA 184 [Source:HGNC Symbol;Acc:HGNC:31555]
0.446237013755442	0.12732345302047	ZNF726	730087	zinc finger protein 726 [Source:HGNC Symbol;Acc:HGNC:32462]
0.445940941023015	0.00454088759087547	UBASH3A	53347	ubiquitin associated and SH3 domain containing A [Source:HGNC Symbol;Acc:HGNC:12462]
0.445676960218596	0.0154680552855524	KLK9	284366	kallikrein-related peptidase 9 [Source:HGNC Symbol;Acc:HGNC:6370]
0.445029128519723	0.174710370152222	ARRDC3	57561	arrestin domain containing 3 [Source:HGNC Symbol;Acc:HGNC:29263]
0.444725281769853	0.195707790830037	PSAT1	29968	phosphoserine aminotransferase 1 [Source:HGNC Symbol;Acc:HGNC:19129]
0.444715641220413	0.0354304831860265	SNORD104	692227	small nucleolar RNA, C/D box 104 [Source:HGNC Symbol;Acc:HGNC:32768]
0.444690378029829	0.0584417524108634	SLC5A9	200010	solute carrier family 5 (sodium/sugar cotransporter), member 9 [Source:HGNC Symbol;Acc:HGNC:22146]
0.444359153214297	0.0935667790075287	MXD3	83463	MAX dimerization protein 3 [Source:HGNC Symbol;Acc:HGNC:14008]
0.444284626052332	0.0632020563343278	CYP11B1	1584	cytochrome P450, family 11, subfamily B, polypeptide 1 [Source:HGNC Symbol;Acc:HGNC:2591]
0.444132430200804	0.129884654175847	SASH3	54440	SAM and SH3 domain containing 3 [Source:HGNC Symbol;Acc:HGNC:15975]
0.444130685925009	0.0911578048994273	CSAD	51380	cysteine sulfinic acid decarboxylase [Source:HGNC Symbol;Acc:HGNC:18966]
0.443731138531824	0.000997559786163351	FBP2	8789	fructose-1,6-bisphosphatase 2 [Source:HGNC Symbol;Acc:HGNC:3607]
0.443651114756862	0.0464896718675681	TMEM241	85019	transmembrane protein 241 [Source:HGNC Symbol;Acc:HGNC:31723]
0.443251433987404	0.0950440528650104	ISYNA1	51477	inositol-3-phosphate synthase 1 [Source:HGNC Symbol;Acc:HGNC:29821]
0.443142648150713	0.195602115531994	TYR	7299	tyrosinase [Source:HGNC Symbol;Acc:HGNC:12442]
0.443009746603048	0.0161426930401427	NANOGNB	360030	NANOG neighbor homeobox [Source:HGNC Symbol;Acc:HGNC:24958]
0.442897610442469	0.000275539465043955	MC2R	4158	melanocortin 2 receptor (adrenocorticotropic hormone) [Source:HGNC Symbol;Acc:HGNC:6930]
0.442537274146247	0.108208511672791	YBX2	51087	Y box binding protein 2 [Source:HGNC Symbol;Acc:HGNC:17948]

0.441943826695842	0.0544586969946489	KRTAP19-2	337969	keratin associated protein 19-2 [Source:HGNC Symbol;Acc:HGNC:18937]
0.441679151255403	0.076755250567702	D2HGDH	728294	D-2-hydroxyglutarate dehydrogenase [Source:HGNC Symbol;Acc:HGNC:28358]
0.441101179423939	0.027785329968692	ODF3B	440836	outer dense fiber of sperm tails 3B [Source:HGNC Symbol;Acc:HGNC:34388]
0.440872230338168	0.112566991910132	IL9R	3581	interleukin 9 receptor [Source:HGNC Symbol;Acc:HGNC:6030]
0.440288131712292	0.199585064010241	SVOPL	136306	SVOP-like [Source:HGNC Symbol;Acc:HGNC:27034]
0.440283114785612	0.154794210003231	ZBTB12	221527	zinc finger and BTB domain containing 12 [Source:HGNC Symbol;Acc:HGNC:19066]
0.440105326866933	0.105116460323455	PCDHGA6	56109	protocadherin gamma subfamily A, 6 [Source:HGNC Symbol;Acc:HGNC:8704]
0.439606725017439	0.115058549398071	LCE4A	199834	late cornified envelope 4A [Source:HGNC Symbol;Acc:HGNC:16613]
0.438789259812238	0.0450252157707543	CILP2	148113	cartilage intermediate layer protein 2 [Source:HGNC Symbol;Acc:HGNC:24213]
0.438405486398622	0.164367945118583	NXPH4	11247	neurexophilin 4 [Source:HGNC Symbol;Acc:HGNC:8078]
0.437792859540286	0.00177607205427933	NKX3-1	4824	NK3 homeobox 1 [Source:HGNC Symbol;Acc:HGNC:7838]
0.437645559619096	0.0266894290346373	TTC24	164118	tetratricopeptide repeat domain 24 [Source:HGNC Symbol;Acc:HGNC:32348]
0.437629092657384	0.0280220178899778	HAS1	3036	hyaluronan synthase 1 [Source:HGNC Symbol;Acc:HGNC:4818]
0.437167977683005	0.0384678524515498	C12orf74	338809	chromosome 12 open reading frame 74 [Source:HGNC Symbol;Acc:HGNC:27887]
0.436608107656455	0.187534300521137	DGKZ	8525	diacylglycerol kinase, zeta [Source:HGNC Symbol;Acc:HGNC:2857]
0.436402075509109	0.105104989969768	PNMT	5409	phenylethanolamine N-methyltransferase [Source:HGNC Symbol;Acc:HGNC:9160]
0.436186606262666	0.00967758594439239	RASL11B	65997	RAS-like, family 11, member B [Source:HGNC Symbol;Acc:HGNC:23804]
0.436108396619262	0.0679372122656788	CA11	770	carbonic anhydrase XI [Source:HGNC Symbol;Acc:HGNC:1370]
0.435854726823893	0.0769950920099584	SLC22A16	85413	solute carrier family 22 (organic cation/carnitine transporter), member 16 [Source:HGNC Symbol;Acc:HGNC:20302]
0.435533284384638	0.19936143978916	CLEC2D	29121	C-type lectin domain family 2, member D [Source:HGNC Symbol;Acc:HGNC:14351]
0.435447369970424	0.0656926174253283	KCNQ4	9132	potassium voltage-gated channel, KQT-like subfamily, member 4 [Source:HGNC Symbol;Acc:HGNC:6298]
0.435395857625994	0.073530129831823	AFF2	2334	AF4/FMR2 family, member 2 [Source:HGNC Symbol;Acc:HGNC:3776]
0.435339494256137	0.0365918885694237	HAPLN3	145864	hyaluronan and proteoglycan link protein 3 [Source:HGNC Symbol;Acc:HGNC:21446]
0.435070593636298	0.0899344428356472	DCAF12L1	139170	DDB1 and CUL4 associated factor 12-like 1 [Source:HGNC Symbol;Acc:HGNC:29395]
0.43503735340133	0.0715642661912074	WWC2	53842	WW and C2 domain containing 2 [Source:HGNC Symbol;Acc:HGNC:24148]
0.434897482991328	0.168237509421035	DAND5	199699	DAN domain family member 5, BMP antagonist [Source:HGNC Symbol;Acc:HGNC:26780]
0.434719443987111	0.0850344950016214	SAA2	6289	serum amyloid A2 [Source:HGNC Symbol;Acc:HGNC:10514]
0.434293954727862	0.0871936396066001	MIR425	494337	microRNA 425 [Source:HGNC Symbol;Acc:HGNC:31882]
0.434272807350186	0.0317375720271266	NUP210	23225	nucleoporin 210kDa [Source:HGNC Symbol;Acc:HGNC:30052]
0.434033646216429	0.000174596393111654	ADAMTS17	170691	ADAM metallopeptidase with thrombospondin type 1 motif, 17 [Source:HGNC Symbol;Acc:HGNC:17109]
0.433430044513574	0.077115548192599	ADCY8	114	adenylate cyclase 8 (brain) [Source:HGNC Symbol;Acc:HGNC:239]
0.433198177826985	0.168059741022036		284379	
0.432850278678371	0.0205791892407686	TBX5	6910	T-box 5 [Source:HGNC Symbol;Acc:HGNC:11604]
0.432697358614749	0.154576353795304	PRR3	80742	proline rich 3 [Source:HGNC Symbol;Acc:HGNC:21149]
0.432536609416138	0.0108004643355033	TTC21A	199223	tetratricopeptide repeat domain 21A [Source:HGNC Symbol;Acc:HGNC:30761]
0.432382174052565	0.00163832128735148	WISP1-OT1	8840	WISP1 overlapping transcript 1 [Source:HGNC Symbol;Acc:HGNC:49126]
0.432197014355332	0.106454960476816	CCL27	10850	chemokine (C-C motif) ligand 27 [Source:HGNC Symbol;Acc:HGNC:10626]
0.431951658901337	0.192635324463118	LINC01521	54944	long intergenic non-protein coding RNA 1521 [Source:HGNC Symbol;Acc:HGNC:26029]
0.431897136775147	0.11403789271316		374387	
0.431654314665328	0.038490502375731	ZNF775	285971	zinc finger protein 775 [Source:HGNC Symbol;Acc:HGNC:28501]
0.431077351876619	0.0100599760546688	WWC2-AS2	152641	WWC2 antisense RNA 2 [Source:HGNC Symbol;Acc:HGNC:26390]
0.430925404177394	0.113022947993046	IFNK	56832	interferon, kappa [Source:HGNC Symbol;Acc:HGNC:21714]
0.430918160715344	0.120595277140903	SCARNA11	677780	small Cajal body-specific RNA 11 [Source:HGNC Symbol;Acc:HGNC:32568]
0.430824987356573	0.164899147738091	DDIAS	220042	DNA damage-induced apoptosis suppressor [Source:HGNC Symbol;Acc:HGNC:26351]
0.430803550937411	0.00108040584299452	ARL5C	390790	ADP-ribosylation factor-like 5C [Source:HGNC Symbol;Acc:HGNC:31111]
0.430791364134506	0.1742759097462	HOXA6	3203	homeobox A6 [Source:HGNC Symbol;Acc:HGNC:5107]
0.430542606844056	0.00229883608828587	TRIP6	7205	thyroid hormone receptor interactor 6 [Source:HGNC Symbol;Acc:HGNC:12311]
0.43000021609299	0.0436537229095703	LINC00469	283982	long intergenic non-protein coding RNA 469 [Source:HGNC Symbol;Acc:HGNC:26863]
0.429341682579757	0.185762536830566	ADAM21	8747	ADAM metallopeptidase domain 21 [Source:HGNC Symbol;Acc:HGNC:200]
0.428752325264354	0.0291435640718939	CPA2	1358	carboxypeptidase A2 (pancreatic) [Source:HGNC Symbol;Acc:HGNC:2297]
0.428624901839932	0.117415281194305	C16orf95	100506581	chromosome 16 open reading frame 95 [Source:HGNC Symbol;Acc:HGNC:40033]
0.428604671325648	0.0287404998837093	DRD5	1816	dopamine receptor D5 [Source:HGNC Symbol;Acc:HGNC:3026]
0.428295625137609	0.13488706886454	TEAD4	7004	TEA domain family member 4 [Source:HGNC Symbol;Acc:HGNC:11717]
0.428199660102266	0.184554593664572	TOP2A	7153	topoisomerase (DNA) II alpha 170kDa [Source:HGNC Symbol;Acc:HGNC:11989]
0.427837205091737	0.155395240805073	PRSS57	400668	protease, serine, 57 [Source:HGNC Symbol;Acc:HGNC:31397]
0.427811681403842	0.0240259439251342	SLC4A3	6508	solute carrier family 4 (anion exchanger), member 3 [Source:HGNC Symbol;Acc:HGNC:11029]
0.427706739644599	0.054276794449675	ARMC12	221481	armadillo repeat containing 12 [Source:HGNC Symbol;Acc:HGNC:21099]
0.427425710892597	0.162378064776965	SNORA55	677834	small nucleolar RNA, H/ACA box 55 [Source:HGNC Symbol;Acc:HGNC:32649]
0.426409056230331	0.0124461547974361	NLE1	54475	notchless homolog 1 (Drosophila) [Source:HGNC Symbol;Acc:HGNC:19889]
0.426189991614994	0.0775443826343277	SPRY3	10251	sprouty homolog 3 (Drosophila) [Source:HGNC Symbol;Acc:HGNC:11271]
0.426095964941917	0.0201612178046428	TKTL2	84076	transketolase-like 2 [Source:HGNC Symbol;Acc:HGNC:25313]
0.426083443568068	2.97792692779043e-05	HOXB8	3218	homeobox B8 [Source:HGNC Symbol;Acc:HGNC:5119]
0.426017334677799	0.0940699341997955	RP9	6100	retinitis pigmentosa 9 (autosomal dominant) [Source:HGNC Symbol;Acc:HGNC:10288]
0.425931802606495	0.0972871104654772	GPR135	64582	G protein-coupled receptor 135 [Source:HGNC Symbol;Acc:HGNC:19991]
0.425862597297013	0.17851370932595	H2BFM	286436	H2B histone family, member M [Source:HGNC Symbol;Acc:HGNC:27867]
0.42575148743215	0.0399486511880774	FAM189A2	9413	family with sequence similarity 189, member A2 [Source:HGNC Symbol;Acc:HGNC:24820]
0.425365042708101	0.0810033079775468	OR6T1	219874	olfactory receptor, family 6, subfamily T, member 1 [Source:HGNC Symbol;Acc:HGNC:14848]
0.425085249722764	0.122401767836776	FAM184A	79632	family with sequence similarity 184, member A [Source:HGNC Symbol;Acc:HGNC:20991]
0.42499223058654	0.000582917816599641	LYPD4	147719	LY6/PLAUR domain containing 4 [Source:HGNC Symbol;Acc:HGNC:28659]
0.424317380067229	0.117908061236981		83747	
0.424308319079331	0.0224173852228988	LGALS12	85329	lectin, galactoside-binding, soluble, 12 [Source:HGNC Symbol;Acc:HGNC:15788]
0.424158560715714	0.0217483130386026		132200	
0.424076094251335	0.0418853021850163	CHRNG	1146	cholinergic receptor, nicotinic, gamma (muscle) [Source:HGNC Symbol;Acc:HGNC:1967]
0.423578950825233	0.0389175123742324	PRM3	58531	protamine 3 [Source:HGNC Symbol;Acc:HGNC:13732]
0.423328491595112	0.0183663301630037	SSBP3-AS1	619518	SSBP3 antisense RNA 1 [Source:HGNC Symbol;Acc:HGNC:32328]
0.423024554650967	0.0260101593190102	DHODH	1723	dihydroorotate dehydrogenase (quinone) [Source:HGNC Symbol;Acc:HGNC:2867]
0.422735860869274	0.0232464974077696	VTN	7448	vitronectin [Source:HGNC Symbol;Acc:HGNC:12724]
0.422059303218959	0.0198490738117495	LYSMD2	256586	LysM, putative peptidoglycan-binding, domain containing 2 [Source:HGNC Symbol;Acc:HGNC:28571]
0.421984201628325	0.0668796883515089	SFXN2	118980	sideroflexin 2 [Source:HGNC Symbol;Acc:HGNC:16086]
0.421968369180221	0.0205761783276101	RGS19	10287	regulator of G-protein signaling 19 [Source:HGNC Symbol;Acc:HGNC:13735]
0.42172947295647	0.0232124582136399	DACT2	168002	dishevelled-binding antagonist of beta-catenin 2 [Source:HGNC Symbol;Acc:HGNC:21231]
0.420949816926348	0.0619321788772604	FAM132B	151176	family with sequence similarity 132, member B [Source:HGNC Symbol;Acc:HGNC:26727]
0.420752132616184	0.0223027953879384	HOXC10	3226	homeobox C10 [Source:HGNC Symbol;Acc:HGNC:5122]
0.420639251458833	0.0191354974644986	ANKRD18A	253650	ankyrin repeat domain 18A [Source:HGNC Symbol;Acc:HGNC:23643]
0.420547599201865	0.0453093911367284	SLC25A48	153328	solute carrier family 25, member 48 [Source:HGNC Symbol;Acc:HGNC:30451]
0.42022562045547	0.145808866423172	HIST1H4E	8363	histone cluster 1, H4e [Source:HGNC Symbol;Acc:HGNC:4790]
0.420051092157428	0.169534785587149	PARD6B	84612	par-6 family cell polarity regulator beta [Source:HGNC Symbol;Acc:HGNC:16245]
0.419639574848677	0.00400303714577614	MLC1	23209	megalencephalic leukoencephalopathy with subcortical cysts 1 [Source:HGNC Symbol;Acc:HGNC:17082]
0.418630704541181	0.00209872701418745	MMEL1	79258	membrane metallo-endopeptidase-like 1 [Source:HGNC Symbol;Acc:HGNC:14668]
0.418435505423444	0.0254779181488987	SSTR4	6754	somatostatin receptor 4 [Source:HGNC Symbol;Acc:HGNC:11333]
0.418037469796959	0.010593436938551	CATIP	375307	ciliogenesis associated TTC17 interacting protein [Source:HGNC Symbol;Acc:HGNC:25062]
0.417605197902647	0.0179107610835545	COL28A1	340267	collagen, type XXVIII, alpha 1 [Source:HGNC Symbol;Acc:HGNC:22442]
0.41748780353046	0.0958310245866883	PLA1A	51365	phospholipase A1 member A [Source:HGNC Symbol;Acc:HGNC:17661]
0.416969298242279	0.189129376727712	UNC5CL	222643	unc-5 homolog C (C. elegans)-like [Source:HGNC Symbol;Acc:HGNC:21203]
0.416834487073472	0.0103240577703932	KCNJ11	3767	potassium inwardly-rectifying channel, subfamily J, member 11 [Source:HGNC Symbol;Acc:HGNC:6257]
0.416634359899182	0.159350910824708	NUGGC	389643	nuclear GTPase, germinal center associated [Source:HGNC Symbol;Acc:HGNC:33550]
0.41643320652347	0.0119711565882754	CEACAM21	90273	carcinoembryonic antigen-related cell adhesion molecule 21 [Source:HGNC Symbol;Acc:HGNC:28834]
0.416417221044759	0.0244444141881081	FAM205B	389715	family with sequence similarity 205, member B [Source:HGNC Symbol;Acc:HGNC:24504]
0.416241578454731	0.0612106644118727	GPR62	118442	G protein-coupled receptor 62 [Source:HGNC Symbol;Acc:HGNC:13301]
0.416033967269089	0.110248042293824		100532732	
0.416004387943066	0.0722932811804983	B3GAT2	135152	beta-1,3-glucuronyltransferase 2 [Source:HGNC Symbol;Acc:HGNC:922]
0.415992503157875	0.191898855438405	FER1L6-AS2	157376	FER1L6 antisense RNA 2 [Source:HGNC Symbol;Acc:HGNC:26534]
0.415986770816496	0.159673682325689	ESPNP	284729	espin pseudogene [Source:HGNC Symbol;Acc:HGNC:23285]
0.415639803074098	0.052196033751721	SIRT4	23409	sirtuin 4 [Source:HGNC Symbol;Acc:HGNC:14932]
0.415551649303218	0.155375618715689	EME2	197342	essential meiotic structure-specific endonuclease subunit 2 [Source:HGNC Symbol;Acc:HGNC:27289]
0.414852452826877	0.0980052079927305	SCN8A	6334	sodium channel, voltage gated, type VIII, alpha subunit [Source:HGNC Symbol;Acc:HGNC:10596]
0.414441939663846	0.10116537047687	TNFSF14	8740	tumor necrosis factor (ligand) superfamily, member 14 [Source:HGNC Symbol;Acc:HGNC:11930]
0.414269312882586	0.00281134779032529	FAM163A	148753	family with sequence similarity 163, member A [Source:HGNC Symbol;Acc:HGNC:28274]
0.414170498446173	0.0793288884377873	DPH7	92715	diphthamide biosynthesis 7 [Source:HGNC Symbol;Acc:HGNC:25199]
0.414131518027927	0.131588876198633	PRR22	163154	proline rich 22 [Source:HGNC Symbol;Acc:HGNC:28354]
0.414064888204805	0.047620990467073	RPRML	388394	reprimo-like [Source:HGNC Symbol;Acc:HGNC:32422]
0.413913804801826	0.0157128026847052	LHX9	56956	LIM homeobox 9 [Source:HGNC Symbol;Acc:HGNC:14222]
0.413697602219311	0.0839698268484963	LINC00087	644596	long intergenic non-protein coding RNA 87 [Source:HGNC Symbol;Acc:HGNC:34500]
0.413664974627071	0.169429511228524	IFNA8	3445	interferon, alpha 8 [Source:HGNC Symbol;Acc:HGNC:5429]
0.413645413630316	0.0729961951962186	KIR3DL2	3812	killer cell immunoglobulin-like receptor, three domains, long cytoplasmic tail, 2 [Source:HGNC Symbol;Acc:HGNC:6339]
0.413430490704113	0.165591472074413	SNORD102	26771	small nucleolar RNA, C/D box 102 [Source:HGNC Symbol;Acc:HGNC:10099]
0.413057751060197	0.133956243745105	RPL23AP7	118433	ribosomal protein L23a pseudogene 7 [Source:HGNC Symbol;Acc:HGNC:17336]
0.412921551346137	0.0103002406208102		101928841	
0.4128064163065	0.0334514090914828	OR5H15	403274	olfactory receptor, family 5, subfamily H, member 15 [Source:HGNC Symbol;Acc:HGNC:31287]
0.41270063174256	0.0708710667562324	RASAL3	64926	RAS protein activator like 3 [Source:HGNC Symbol;Acc:HGNC:26129]
0.412576609699216	0.0206194551460751	HOXB1	3211	homeobox B1 [Source:HGNC Symbol;Acc:HGNC:5111]
0.412493908392966	0.000187094382671385	MATK	4145	megakaryocyte-associated tyrosine kinase [Source:HGNC Symbol;Acc:HGNC:6906]
0.412358495797394	0.147126652669552	OR3A3	8392	olfactory receptor, family 3, subfamily A, member 3 [Source:HGNC Symbol;Acc:HGNC:8284]
0.412186813949156	0.126273130115021	DHX38	9785	DEAH (Asp-Glu-Ala-His) box polypeptide 38 [Source:HGNC Symbol;Acc:HGNC:17211]
0.411801791335337	0.128145442287116	APITD1	378708	apoptosis-inducing, TAF9-like domain 1 [Source:HGNC Symbol;Acc:HGNC:23163]
0.411699120434843	0.184424128036784	ZNF154	7710	zinc finger protein 154 [Source:HGNC Symbol;Acc:HGNC:12939]
0.411611455482722	0.0697289212548263	ONECUT1	3175	one cut homeobox 1 [Source:HGNC Symbol;Acc:HGNC:8138]
0.411381268343656	0.0524376650531972	SERPINA9	327657	serpin peptidase inhibitor, clade A (alpha-1 antiproteinase, antitrypsin), member 9 [Source:HGNC Symbol;Acc:HGNC:15995]
0.411067196406379	0.022444061532037		441239	
0.411042428341807	0.00413602283709345	SNPH	9751	syntaphilin [Source:HGNC Symbol;Acc:HGNC:15931]
0.411040235418249	0.0156066764533141	GPR26	2849	G protein-coupled receptor 26 [Source:HGNC Symbol;Acc:HGNC:4481]
0.410829866552089	0.167938894276948	WISP2	8839	WNT1 inducible signaling pathway protein 2 [Source:HGNC Symbol;Acc:HGNC:12770]
0.410726691411409	0.0150252026020443	C6orf223	221416	chromosome 6 open reading frame 223 [Source:HGNC Symbol;Acc:HGNC:28692]
0.41066999374903	0.00666878528836795	CST8	10047	cystatin 8 (cystatin-related epididymal specific) [Source:HGNC Symbol;Acc:HGNC:2480]
0.41048622987849	0.000333668997353668	SLC25A53	401612	solute carrier family 25, member 53 [Source:HGNC Symbol;Acc:HGNC:31894]
0.410240329310185	0.145713364794669	COX8C	341947	cytochrome c oxidase subunit VIIIC [Source:HGNC Symbol;Acc:HGNC:24382]
0.410078791781214	0.142504461132646	UCN2	90226	urocortin 2 [Source:HGNC Symbol;Acc:HGNC:18414]
0.410032930678778	0.036472292856294	HTR7P1	93164	5-hydroxytryptamine (serotonin) receptor 7 pseudogene 1 [Source:HGNC Symbol;Acc:HGNC:30411]
0.409731508673321	0.00920664628614841	VSIG1	340547	V-set and immunoglobulin domain containing 1 [Source:HGNC Symbol;Acc:HGNC:28675]
0.409318716226833	0.0515625492680473	FTHL17	53940	ferritin, heavy polypeptide-like 17 [Source:HGNC Symbol;Acc:HGNC:3987]
0.409123757403478	0.107639026259939	NPAP1	23742	nuclear pore associated protein 1 [Source:HGNC Symbol;Acc:HGNC:1190]

0.408653430188631	0.111309436344789	CST4	1472	cystatin S [Source:HGNC Symbol;Acc:HGNC:2476]
0.408585873985889	0.00752555249953912	TERC	7012	telomerase RNA component [Source:HGNC Symbol;Acc:HGNC:11727]
0.408554432123772	0.154609290227503	KRTAP10-10	353333	keratin associated protein 10-10 [Source:HGNC Symbol;Acc:HGNC:22972]
0.408545641349175	0.140575612326804	CCDC64	92558	coiled-coil domain containing 64 [Source:HGNC Symbol;Acc:HGNC:28095]
0.408503706973481	0.145946346307436	SULT2B1	6820	sulfotransferase family, cytosolic, 2B, member 1 [Source:HGNC Symbol;Acc:HGNC:11459]
0.407518448941604	0.0870681847857136	STPG2	285555	sperm-tail PG-rich repeat containing 2 [Source:HGNC Symbol;Acc:HGNC:28712]
0.407278416979148	0.0440165899024785	A4GALT	53947	alpha 1,4-galactosyltransferase [Source:HGNC Symbol;Acc:HGNC:18149]
0.406328101124286	0.030285386643498	WFDC12	128488	WAP four-disulfide core domain 12 [Source:HGNC Symbol;Acc:HGNC:16115]
0.406286567829921	0.0628378495887602	CYP1A1	1543	cytochrome P450, family 1, subfamily A, polypeptide 1 [Source:HGNC Symbol;Acc:HGNC:2595]
0.406037003513146	0.0563271349555369	MKRN3	7681	makorin ring finger protein 3 [Source:HGNC Symbol;Acc:HGNC:7114]
0.405810147609974	0.197521828885404	SPC25	57405	SPC25, NDC80 kinetochore complex component [Source:HGNC Symbol;Acc:HGNC:24031]
0.405593114106346	0.123860332802052	NEIL1	79661	nei endonuclease VIII-like 1 (E. coli) [Source:HGNC Symbol;Acc:HGNC:18448]
0.405334100264392	0.000529087607990644	PDCD1	5133	programmed cell death 1 [Source:HGNC Symbol;Acc:HGNC:8760]
0.405183407564922	0.0842035000189701	PACSIN1	29993	protein kinase C and casein kinase substrate in neurons 1 [Source:HGNC Symbol;Acc:HGNC:8570]
0.405123242732881	0.0454913156698522	FOSL1	8061	FOS-like antigen 1 [Source:HGNC Symbol;Acc:HGNC:13718]
0.404889871513376	0.0179794711619495	WFDC10B	280664	WAP four-disulfide core domain 10B [Source:HGNC Symbol;Acc:HGNC:20479]
0.404752237224008	0.0163046949607653	FAM170B	170370	family with sequence similarity 170, member B [Source:HGNC Symbol;Acc:HGNC:19736]
0.404699987539206	0.167525719622991	RPL13A	26817	ribosomal protein L13a [Source:HGNC Symbol;Acc:HGNC:10304]
0.404065422645715	0.0772719903039177	SNORA70B	100124537	small nucleolar RNA, H/ACA box 70B [Source:HGNC Symbol;Acc:HGNC:33618]
0.403529275743911	0.0162916558674355	PRODH	5625	proline dehydrogenase (oxidase) 1 [Source:HGNC Symbol;Acc:HGNC:9453]
0.402999731451905	0.0503678976627229	CHAC1	79094	ChaC, cation transport regulator homolog 1 (E. coli) [Source:HGNC Symbol;Acc:HGNC:28680]
0.402712772113251	0.0181127081160274	IRX2	153572	iroquois homeobox 2 [Source:HGNC Symbol;Acc:HGNC:14359]
0.402671065493047	0.00341152385801701	FGF19	9965	fibroblast growth factor 19 [Source:HGNC Symbol;Acc:HGNC:3675]
0.402663675938298	0.0551883081929864	HES3	390992	hes family bHLH transcription factor 3 [Source:HGNC Symbol;Acc:HGNC:26226]
0.402597912646903	0.0632242525555501	TMEM178A	130733	transmembrane protein 178A [Source:HGNC Symbol;Acc:HGNC:28517]
0.402586728710723	0.0475328864502064	ADAM33	80332	ADAM metallopeptidase domain 33 [Source:HGNC Symbol;Acc:HGNC:15478]
0.401658246588975	0.0032649408734743		360226	
0.401642935315426	0.141375395546924	LRRCC1	85444	leucine rich repeat and coiled-coil centrosomal protein 1 [Source:HGNC Symbol;Acc:HGNC:29373]
0.401184430780623	0.00490091360437645	TMPRSS3	64699	transmembrane protease, serine 3 [Source:HGNC Symbol;Acc:HGNC:11877]
0.400895305030713	0.0701009721079067	KCNB1	3745	potassium voltage-gated channel, Shab-related subfamily, member 1 [Source:HGNC Symbol;Acc:HGNC:6231]
0.400883325609041	0.0470751059804815	RTN4R	65078	reticulon 4 receptor [Source:HGNC Symbol;Acc:HGNC:18601]
0.40073830928398	0.166670797224306	OR2B2	81697	olfactory receptor, family 2, subfamily B, member 2 [Source:HGNC Symbol;Acc:HGNC:13966]
0.400575338440344	0.127286911837314	SACS	26278	sacsin molecular chaperone [Source:HGNC Symbol;Acc:HGNC:10519]
0.400459394003668	0.190210795896652	ANKRD61	100310846	ankyrin repeat domain 61 [Source:HGNC Symbol;Acc:HGNC:22467]
0.399922859885009	0.000970368040768657	HFE2	148738	hemochromatosis type 2 (juvenile) [Source:HGNC Symbol;Acc:HGNC:4887]
0.399656853233849	0.113112135341897	C3orf56	285311	chromosome 3 open reading frame 56 [Source:HGNC Symbol;Acc:HGNC:32481]
0.399119023906796	0.0678604024735918	ARHGAP4	393	Rho GTPase activating protein 4 [Source:HGNC Symbol;Acc:HGNC:674]
0.398626262541056	0.000850226643422032		100130083	
0.398526559380892	0.0906903846423992	WNT9B	7484	wingless-type MMTV integration site family, member 9B [Source:HGNC Symbol;Acc:HGNC:12779]
0.398461717415497	0.0611408439683995	UBXN11	91544	UBX domain protein 11 [Source:HGNC Symbol;Acc:HGNC:30600]
0.39826709031591	0.056908943271274	TUSC5	286753	tumor suppressor candidate 5 [Source:HGNC Symbol;Acc:HGNC:29592]
0.398087812382098	0.0132061811806891	CCDC88B	283234	coiled-coil domain containing 88B [Source:HGNC Symbol;Acc:HGNC:26757]
0.398035966684212	0.0246355343911994		373861	
0.397976506178529	0.148124198908024	TEKT4	150483	tektin 4 [Source:HGNC Symbol;Acc:HGNC:31012]
0.397507341933856	0.054718215976865	ADORA2A-A	646023	ADORA2A antisense RNA 1 [Source:HGNC Symbol;Acc:HGNC:37122]
0.397499719301371	0.050461452983378	LCE1B	353132	late cornified envelope 1B [Source:HGNC Symbol;Acc:HGNC:16611]
0.397450613101536	0.0448094675181435	FAM133A	286499	family with sequence similarity 133, member A [Source:HGNC Symbol;Acc:HGNC:26748]
0.397407515967771	0.0453659931568452	KIF24	347240	kinesin family member 24 [Source:HGNC Symbol;Acc:HGNC:19916]
0.397003972911513	0.104532598677436	TMPRSS11A	339967	transmembrane protease, serine 11A [Source:HGNC Symbol;Acc:HGNC:27954]
0.396812136748246	0.136981621550381	GFI1B	8328	growth factor independent 1B transcription repressor [Source:HGNC Symbol;Acc:HGNC:4238]
0.396430578989546	0.0877446476981309	FAM71C	196472	family with sequence similarity 71, member C [Source:HGNC Symbol;Acc:HGNC:28594]
0.396401864653839	0.0104372009266404	KCNH6	81033	potassium voltage-gated channel, subfamily H (eag-related), member 6 [Source:HGNC Symbol;Acc:HGNC:18862]
0.396360427800799	0.080463731375095	TMEM89	440955	transmembrane protein 89 [Source:HGNC Symbol;Acc:HGNC:32372]
0.396335762422924	0.105793102299024	DUSP9	1852	dual specificity phosphatase 9 [Source:HGNC Symbol;Acc:HGNC:3076]
0.396282341760131	0.0629869952900836	C20orf62	140834	chromosome 20 open reading frame 62 [Source:HGNC Symbol;Acc:HGNC:16195]
0.396191229221312	0.0844906490432614	ZNF568	374900	zinc finger protein 568 [Source:HGNC Symbol;Acc:HGNC:25392]
0.396141395963216	0.0200961865999528	BAHCC1	57597	BAH domain and coiled-coil containing 1 [Source:HGNC Symbol;Acc:HGNC:29279]
0.396091408082188	0.0103273267922661	SLC24A4	123041	solute carrier family 24 (sodium/potassium/calcium exchanger), member 4 [Source:HGNC Symbol;Acc:HGNC:10978]
0.395918883439864	0.00757301101489802	EIF4A1	652965	eukaryotic translation initiation factor 4A1 [Source:HGNC Symbol;Acc:HGNC:3282]
0.395801798854416	0.0837339991410267	FAM83D	81610	family with sequence similarity 83, member D [Source:HGNC Symbol;Acc:HGNC:16122]
0.395682444454266	0.0986178494287298	LINC00094	266655	long intergenic non-protein coding RNA 94 [Source:HGNC Symbol;Acc:HGNC:24742]
0.395481650236158	0.15247903614733	CDH24	64403	cadherin 24, type 2 [Source:HGNC Symbol;Acc:HGNC:14265]
0.395340953434243	0.0977959835553621	ACTL9	284382	actin-like 9 [Source:HGNC Symbol;Acc:HGNC:28494]
0.395208709329454	0.198096261547839	ZNF263	283876	zinc finger protein 263 [Source:HGNC Symbol;Acc:HGNC:13056]
0.394849345591	0.113702274876067	SIX2	10736	SIX homeobox 2 [Source:HGNC Symbol;Acc:HGNC:10888]
0.394715892121534	0.0398590482432972	CYP26C1	340665	cytochrome P450, family 26, subfamily C, polypeptide 1 [Source:HGNC Symbol;Acc:HGNC:20577]
0.39452461025214	0.0752140572807951	LIF	3976	leukemia inhibitory factor [Source:HGNC Symbol;Acc:HGNC:6596]
0.394307944348545	0.176196505954258	ANKRD33	341405	ankyrin repeat domain 33 [Source:HGNC Symbol;Acc:HGNC:13788]
0.394040137420147	0.105035573494614	ATRIP	84126	ATR interacting protein [Source:HGNC Symbol;Acc:HGNC:33499]
0.39399873812578	0.0320818858283225	INE1	8552	inactivation escape 1 (non-protein coding) [Source:HGNC Symbol;Acc:HGNC:6060]
0.393947042381576	0.15946365974556	ZNF239	8187	zinc finger protein 239 [Source:HGNC Symbol;Acc:HGNC:13031]
0.39371199995753	0.0914678257667786		644903	
0.393644671133306	0.125248683745926	MAGEB2	4113	melanoma antigen family B, 2 [Source:HGNC Symbol;Acc:HGNC:6809]
0.393546920640385	0.0891030720444646	KRTAP4-1	85285	keratin associated protein 4-1 [Source:HGNC Symbol;Acc:HGNC:18907]
0.393491438783044	0.0793878414814458	RASL10A	10633	RAS-like, family 10, member A [Source:HGNC Symbol;Acc:HGNC:16954]
0.392939864976472	0.163502660764794		100129476	
0.392667424437631	0.0472529275079309	FPR2	2358	formyl peptide receptor 2 [Source:HGNC Symbol;Acc:HGNC:3827]
0.391978323809488	0.122662589051749	LSMEM2	132228	leucine-rich single-pass membrane protein 2 [Source:HGNC Symbol;Acc:HGNC:26781]
0.391823894467407	0.119531846037912	SLFN13	146857	schlafen family member 13 [Source:HGNC Symbol;Acc:HGNC:26481]
0.391470539026637	0.0683834978836191	TRH	7200	thyrotropin-releasing hormone [Source:HGNC Symbol;Acc:HGNC:12298]
0.391394822604613	0.11063772075559	MIR516B1	574490	microRNA 516b-1 [Source:HGNC Symbol;Acc:HGNC:32122]
0.391380892936168	0.0834119866473738	IGFALS	3483	insulin-like growth factor binding protein, acid labile subunit [Source:HGNC Symbol;Acc:HGNC:5468]
0.390921965034891	0.144797381305857	CXCL5	6374	chemokine (C-X-C motif) ligand 5 [Source:HGNC Symbol;Acc:HGNC:10642]
0.390752567016243	0.173485121651843	MIR503HG	84848	MIR503 host gene (non-protein coding) [Source:HGNC Symbol;Acc:HGNC:28258]
0.390651280168638	0.0912839711627034	PARP6	56965	poly (ADP-ribose) polymerase family, member 6 [Source:HGNC Symbol;Acc:HGNC:26921]
0.390350353683944	0.132993292723482	OR5A2	219981	olfactory receptor, family 5, subfamily A, member 2 [Source:HGNC Symbol;Acc:HGNC:15249]
0.390296460117701	0.191608004574876	MAGEA3	4102	melanoma antigen family A, 3 [Source:HGNC Symbol;Acc:HGNC:6801]
0.39016882420471	0.0265140505609677	IGFL4	444882	IGF-like family member 4 [Source:HGNC Symbol;Acc:HGNC:32931]
0.39013305504553	0.108238508008134	MIR193A	406968	microRNA 193a [Source:HGNC Symbol;Acc:HGNC:31563]
0.390114947999387	0.121617277502461	LTB4R2	56413	leukotriene B4 receptor 2 [Source:HGNC Symbol;Acc:HGNC:19260]
0.389913819656697	0.00525738159841203	EFCAB12	90288	EF-hand calcium binding domain 12 [Source:HGNC Symbol;Acc:HGNC:28061]
0.389605474888834	0.0409948465738239	VPREB1	7441	pre-B lymphocyte 1 [Source:HGNC Symbol;Acc:HGNC:12709]
0.38937162297373	0.0797762799824512	AQP9	366	aquaporin 9 [Source:HGNC Symbol;Acc:HGNC:643]
0.389225936956537	0.0195278309459164	MAGEA1	4100	melanoma antigen family A, 1 (directs expression of antigen MZ2-E) [Source:HGNC Symbol;Acc:HGNC:6796]
0.389119810303267	0.0292170194588244	MIR194-2	406970	microRNA 194-2 [Source:HGNC Symbol;Acc:HGNC:31565]
0.388714816854915	0.0614252816031465	GCM2	9247	glial cells missing homolog 2 (Drosophila) [Source:HGNC Symbol;Acc:HGNC:4198]
0.38870403785194	0.149394260592597	KCNC4	3749	potassium voltage-gated channel, Shaw-related subfamily, member 4 [Source:HGNC Symbol;Acc:HGNC:6236]
0.38868826610413	0.0501359475913965	PRB4	5545	proline-rich protein BstNI subfamily 4 [Source:HGNC Symbol;Acc:HGNC:9340]
0.388626360107835	0.0113478066408926	SPDYE4	388333	speedy/RINGO cell cycle regulator family member E4 [Source:HGNC Symbol;Acc:HGNC:35463]
0.388617977369418	0.084273107599614	BCAS4	55653	breast carcinoma amplified sequence 4 [Source:HGNC Symbol;Acc:HGNC:14367]
0.38851742680231	0.0964730010513869	NRL	4901	neural retina leucine zipper [Source:HGNC Symbol;Acc:HGNC:8002]
0.388356695832395	0.0561894272798622	CXorf57	55086	chromosome X open reading frame 57 [Source:HGNC Symbol;Acc:HGNC:25486]
0.388317237838469	0.0158104069511463	VWA2	340706	von Willebrand factor A domain containing 2 [Source:HGNC Symbol;Acc:HGNC:24709]
0.388256111065419	0.00533215658627015	GRHL3	57822	grainyhead-like 3 (Drosophila) [Source:HGNC Symbol;Acc:HGNC:25839]
0.388159215217337	0.0537367903625394	NAMPT	10135	nicotinamide phosphoribosyltransferase [Source:HGNC Symbol;Acc:HGNC:30092]
0.387946836998084	0.0350451332606328	LYPD3	27076	LY6/PLAUR domain containing 3 [Source:HGNC Symbol;Acc:HGNC:24880]
0.387796846583485	0.077576265539467	NWD2	57495	NACHT and WD repeat domain containing 2 [Source:HGNC Symbol;Acc:HGNC:29229]
0.38764044466588	0.102267713509428	PAK6	56924	p21 protein (Cdc42/Rac)-activated kinase 6 [Source:HGNC Symbol;Acc:HGNC:16061]
0.387606561888647	0.0205280166024769	KLK7	5650	kallikrein-related peptidase 7 [Source:HGNC Symbol;Acc:HGNC:6368]
0.387504384078191	0.134079554978794	RBMXL2	27288	RNA binding motif protein, X-linked-like 2 [Source:HGNC Symbol;Acc:HGNC:17886]
0.386717656840665	0.111697348377916	LGALS13	29124	lectin, galactoside-binding, soluble, 13 [Source:HGNC Symbol;Acc:HGNC:15449]
0.386586424777308	0.188619913057956	GAST	2520	gastrin [Source:HGNC Symbol;Acc:HGNC:4164]
0.386416135328114	0.0460474345369064	LINC00208	83655	long intergenic non-protein coding RNA 208 [Source:HGNC Symbol;Acc:HGNC:15535]
0.38640673114415	0.0550772903051442	ART4	420	ADP-ribosyltransferase 4 (Dombrock blood group) [Source:HGNC Symbol;Acc:HGNC:726]
0.386396937569229	0.0145474853680448	CHRM3	1131	cholinergic receptor, muscarinic 3 [Source:HGNC Symbol;Acc:HGNC:1952]
0.386323568215149	0.0568504372266986	INCA1	388324	inhibitor of CDK, cyclin A1 interacting protein 1 [Source:HGNC Symbol;Acc:HGNC:32224]
0.386281606072824	0.186849656922824	ULBP3	79465	UL16 binding protein 3 [Source:HGNC Symbol;Acc:HGNC:14895]
0.386120742104497	0.114231558207482	RAB3C	115827	RAB3C, member RAS oncogene family [Source:HGNC Symbol;Acc:HGNC:30269]
0.38603250492721	0.150282757385189	TIGIT	201633	T cell immunoreceptor with Ig and ITIM domains [Source:HGNC Symbol;Acc:HGNC:26838]
0.385875780584745	0.192600288637797	ANKRD20A1	391267	ankyrin repeat domain 20 family, member A11, pseudogene [Source:HGNC Symbol;Acc:HGNC:42024]
0.385411111922082	0.0181932947008398	HRH2	3274	histamine receptor H2 [Source:HGNC Symbol;Acc:HGNC:5183]
0.385400028707084	0.154622179963923	LBX2	85474	ladybird homeobox 2 [Source:HGNC Symbol;Acc:HGNC:15525]
0.38520187778522	0.168662564445836	DEFB113	245927	defensin, beta 113 [Source:HGNC Symbol;Acc:HGNC:18094]
0.384883298945631	0.0398801249415924	POU3F3	5455	POU class 3 homeobox 3 [Source:HGNC Symbol;Acc:HGNC:9216]
0.384858420993668	0.0193384172752108	MSGN1	343930	mesogenin 1 [Source:HGNC Symbol;Acc:HGNC:14907]
0.384846699113465	0.00135667054843948		646976	
0.384553447133636	0.0866410512694785	SLC25A41	284427	solute carrier family 25, member 41 [Source:HGNC Symbol;Acc:HGNC:28533]
0.384385680042276	0.0850644732867487	ZNF557	79230	zinc finger protein 557 [Source:HGNC Symbol;Acc:HGNC:28632]
0.384358063931255	0.0978811360353102	VWA5B1	127731	von Willebrand factor A domain containing 5B1 [Source:HGNC Symbol;Acc:HGNC:26538]
0.384188510898442	0.0441709254086586	C22orf23	84645	chromosome 22 open reading frame 23 [Source:HGNC Symbol;Acc:HGNC:18589]
0.384052177794699	0.0728474602787569	GLB1L3	112937	galactosidase, beta 1-like 3 [Source:HGNC Symbol;Acc:HGNC:25147]
0.383948945916435	0.0408141355337467	PLEKHA4	57664	pleckstrin homology domain containing, family A (phosphoinositide binding specific) member 4 [Source:HGNC Symbol;Acc:HGNC:14339]
0.38371962129945	0.174207133742085	LENG8	114823	leukocyte receptor cluster (LRC) member 8 [Source:HGNC Symbol;Acc:HGNC:15500]
0.383540873474723	0.0680421187127843	NPBWR1	2831	neuropeptides B/W receptor 1 [Source:HGNC Symbol;Acc:HGNC:4522]
0.383516411948686	0.158174623997743	DEFB124	245937	defensin, beta 124 [Source:HGNC Symbol;Acc:HGNC:18104]
0.38350974408401	0.0362681608023351	GSDMA	284110	gasdermin A [Source:HGNC Symbol;Acc:HGNC:13311]
0.383341182869234	0.00335853217312951	ADAMTS18	170692	ADAM metallopeptidase with thrombospondin type 1 motif, 18 [Source:HGNC Symbol;Acc:HGNC:17110]
0.383257718150241	0.07202256109453	DGKG	1608	diacylglycerol kinase, gamma 90kDa [Source:HGNC Symbol;Acc:HGNC:2853]
0.38316683111353	0.179169871673727	MOCS1	4337	molybdenum cofactor synthesis 1 [Source:HGNC Symbol;Acc:HGNC:7190]
0.382979875110615	0.0929485633550268	FOXP3	50943	forkhead box P3 [Source:HGNC Symbol;Acc:HGNC:6106]
0.382795420997431	0.10628809151825		100130285	

0.382649145456341	0.176683758001179	CYP2C9	1559	cytochrome P450, family 2, subfamily C, polypeptide 9 [Source:HGNC Symbol;Acc:HGNC:2623]
0.382558707089453	0.0116268984795083	KIAA1549L	25758	KIAA1549-like [Source:HGNC Symbol;Acc:HGNC:24836]
0.382340033872234	0.048834736769136	SERPIND1	3053	serpin peptidase inhibitor, clade D (heparin cofactor), member 1 [Source:HGNC Symbol;Acc:HGNC:4838]
0.381862638235049	0.145091616174144	HIST2H2AB	317772	histone cluster 2, H2ab [Source:HGNC Symbol;Acc:HGNC:20508]
0.38174388257682	0.0120840419943231	FAM228A	653140	family with sequence similarity 228, member A [Source:HGNC Symbol;Acc:HGNC:34418]
0.381698709853736	0.0709273801608787	NPAS1	4861	neuronal PAS domain protein 1 [Source:HGNC Symbol;Acc:HGNC:7894]
0.381614056102247	0.0393489830271287	RIN3	79890	Ras and Rab interactor 3 [Source:HGNC Symbol;Acc:HGNC:18751]
0.381570889379797	0.0142054044460598	OR9A4	130075	olfactory receptor, family 9, subfamily A, member 4 [Source:HGNC Symbol;Acc:HGNC:15095]
0.381373755298914	0.0730482440155591	PLCZ1	89869	phospholipase C, zeta 1 [Source:HGNC Symbol;Acc:HGNC:19218]
0.381348949878168	0.0203674570151649	SLCO5A1	81796	solute carrier organic anion transporter family, member 5A1 [Source:HGNC Symbol;Acc:HGNC:19046]
0.381186241015922	0.0298930879443744	ADAT2	134637	adenosine deaminase, tRNA-specific 2 [Source:HGNC Symbol;Acc:HGNC:21172]
0.381105330507893	0.0424107687451719	SCAMP5	192683	secretory carrier membrane protein 5 [Source:HGNC Symbol;Acc:HGNC:30386]
0.380947347226062	0.00537025963291289	KLF14	136259	Kruppel-like factor 14 [Source:HGNC Symbol;Acc:HGNC:23025]
0.380906035570784	0.18368739340552	TBX21	30009	T-box 21 [Source:HGNC Symbol;Acc:HGNC:11599]
0.380818127316444	0.116284293763293		399886	
0.380801487944906	0.0997385527408834	LGI2	55203	leucine-rich repeat LGI family, member 2 [Source:HGNC Symbol;Acc:HGNC:18710]
0.380565493062956	0.118451283692181	KLK5	25818	kallikrein-related peptidase 5 [Source:HGNC Symbol;Acc:HGNC:6366]
0.380462849416866	9.48972464814812e-06	DFNA5	1687	deafness, autosomal dominant 5 [Source:HGNC Symbol;Acc:HGNC:2810]
0.380153423771278	0.105190296250165	CLDN19	149461	claudin 19 [Source:HGNC Symbol;Acc:HGNC:2040]
0.379987259537694	0.0712426009719563	GDAP1L1	78997	ganglioside induced differentiation associated protein 1-like 1 [Source:HGNC Symbol;Acc:HGNC:4213]
0.379982951559456	0.0857553402155787	MCM2	4171	minichromosome maintenance complex component 2 [Source:HGNC Symbol;Acc:HGNC:6944]
0.379900027630461	0.11297349189546	POU4F1	5457	POU class 4 homeobox 1 [Source:HGNC Symbol;Acc:HGNC:9218]
0.379856994188272	0.0728777967977368	SPOCD1	90853	SPOC domain containing 1 [Source:HGNC Symbol;Acc:HGNC:26338]
0.379373946306263	0.10769147618294	ZNF575	284346	zinc finger protein 575 [Source:HGNC Symbol;Acc:HGNC:27606]
0.379193731519	0.128382961425958	CHAF1A	10036	chromatin assembly factor 1, subunit A (p150) [Source:HGNC Symbol;Acc:HGNC:1910]
0.379087097693462	0.0501932464643243	UPP2	151531	uridine phosphorylase 2 [Source:HGNC Symbol;Acc:HGNC:23061]
0.378970259075756	0.07255667499166 7	C20orf141	128653	chromosome 20 open reading frame 141 [Source:HGNC Symbol;Acc:HGNC:16134]
0.378727578800961	0.0425104075248319	COL4A3	1285	collagen, type IV, alpha 3 (Goodpasture antigen) [Source:HGNC Symbol;Acc:HGNC:2204]
0.378639661391492	0.0183667102665589	NOX5	79400	NADPH oxidase, EF-hand calcium binding domain 5 [Source:HGNC Symbol;Acc:HGNC:14874]
0.378631230131131	0.0902742353729635	AQP10	89872	aquaporin 10 [Source:HGNC Symbol;Acc:HGNC:16029]
0.378028313488488	0.0605008140394964	NAT6	24142	N-acetyltransferase 6 (GCN5-related) [Source:HGNC Symbol;Acc:HGNC:30252]
0.37791900483948	0.0821174151299073	SPATA16	83893	spermatogenesis associated 16 [Source:HGNC Symbol;Acc:HGNC:29935]
0.377578115273873	0.00190848133474067	BZRAP1	9256	benzodiazepine receptor (peripheral) associated protein 1 [Source:HGNC Symbol;Acc:HGNC:16831]
0.377400228276582	0.0491890423602897	GPR61	83873	G protein-coupled receptor 61 [Source:HGNC Symbol;Acc:HGNC:13300]
0.377343136798651	0.0492342753004423		10748	
0.377310951024738	0.0504456545268035	ZC3HAV1L	92092	zinc finger CCCH-type, antiviral 1-like [Source:HGNC Symbol;Acc:HGNC:22423]
0.377096807097132	0.119064809009309	LY6H	4062	lymphocyte antigen 6 complex, locus H [Source:HGNC Symbol;Acc:HGNC:6728]
0.377042158835388	0.158384717550083	ZNF345	25850	zinc finger protein 345 [Source:HGNC Symbol;Acc:HGNC:16367]
0.377008155767839	0.017741515704606		401557	
0.376625921093693	0.141653104072569	SLC17A1	6568	solute carrier family 17 (organic anion transporter), member 1 [Source:HGNC Symbol;Acc:HGNC:10929]
0.37661905312788	0.0931522991230798	VLDLR-AS1	401491	VLDLR antisense RNA 1 [Source:HGNC Symbol;Acc:HGNC:49621]
0.376560913735512	0.070771288030818	SLITRK5	26050	SLIT and NTRK-like family, member 5 [Source:HGNC Symbol;Acc:HGNC:20295]
0.376047382204284	0.186958307516963	AVPR1B	553	arginine vasopressin receptor 1B [Source:HGNC Symbol;Acc:HGNC:896]
0.375981118877274	0.00628957304445949	IDI2	91734	isopentenyl-diphosphate delta isomerase 2 [Source:HGNC Symbol;Acc:HGNC:23487]
0.37596768379596	0.0605898203028814	ZMYND10	51364	zinc finger, MYND-type containing 10 [Source:HGNC Symbol;Acc:HGNC:19412]
0.375786241570301	0.00639829639564552	ADAMTS16	170690	ADAM metallopeptidase with thrombospondin type 1 motif, 16 [Source:HGNC Symbol;Acc:HGNC:17108]
0.375634488513948	0.0855952713267646	PPP3R2	5535	protein phosphatase 3, regulatory subunit B, beta [Source:HGNC Symbol;Acc:HGNC:9318]
0.375548076552217	0.0803171169325003	ALKBH4	54784	alkB, alkylation repair homolog 4 (E. coli) [Source:HGNC Symbol;Acc:HGNC:21900]
0.375402934571424	0.040303764453986	CPXM1	56265	carboxypeptidase X (M14 family), member 1 [Source:HGNC Symbol;Acc:HGNC:15771]
0.37531780369412	0.10969037090811	SLC35G2	80723	solute carrier family 35, member G2 [Source:HGNC Symbol;Acc:HGNC:28480]
0.375133807676164	0.0457795451113906	SYNPO2L	79933	synaptopodin 2-like [Source:HGNC Symbol;Acc:HGNC:23532]
0.374724830645201	0.127144148383885	BARHL1	56751	BarH-like homeobox 1 [Source:HGNC Symbol;Acc:HGNC:953]
0.3746884166788	0.133458626368592	NGFR	4804	nerve growth factor receptor [Source:HGNC Symbol;Acc:HGNC:7809]
0.374607426977096	0.19394546619233 2	GPCPD1	56261	glycerophosphocholine phosphodiesterase GDE1 homolog (S. cerevisiae) [Source:HGNC Symbol;Acc:HGNC:26957]
0.374579457901605	0.11264975806795	MLKL	197259	mixed lineage kinase domain-like [Source:HGNC Symbol;Acc:HGNC:26617]
0.374425480219705	0.108902337190517	B3GNTL1	146712	UDP-GlcNAc:betaGal beta-1,3-N-acetylglucosaminyltransferase-like 1 [Source:HGNC Symbol;Acc:HGNC:21727]
0.374167198593952	0.147793563120299	CRHR2	1395	corticotropin releasing hormone receptor 2 [Source:HGNC Symbol;Acc:HGNC:2358]
0.373861811474452	0.0522938933947955	RETN	56729	resistin [Source:HGNC Symbol;Acc:HGNC:20389]
0.373708797190614	0.0592760990739123	HOXD4	3233	homeobox D4 [Source:HGNC Symbol;Acc:HGNC:5138]
0.373639725072696	0.166394580801189	CASKIN1	57524	CASK interacting protein 1 [Source:HGNC Symbol;Acc:HGNC:20879]
0.373424188878318	0.0802665248776182		148696	
0.373306350995857	0.0417481428098407	OR10C1	442194	olfactory receptor, family 10, subfamily C, member 1 (gene/pseudogene) [Source:HGNC Symbol;Acc:HGNC:8165]
0.372967725629328	0.12058857426307		153684	
0.372873704359153	0.00457642467571739	GRIN2D	2906	glutamate receptor, ionotropic, N-methyl D-aspartate 2D [Source:HGNC Symbol;Acc:HGNC:4588]
0.372442876383699	0.119809684537936	HAMP	57817	hepcidin antimicrobial peptide [Source:HGNC Symbol;Acc:HGNC:15598]
0.372282005546517	0.183499968540002	PTPN7	5778	protein tyrosine phosphatase, non-receptor type 7 [Source:HGNC Symbol;Acc:HGNC:9659]
0.372253252929075	0.148520043469841	CERS4	79603	ceramide synthase 4 [Source:HGNC Symbol;Acc:HGNC:23747]
0.372189636194226	0.113603541568157	VCX	26609	variable charge, X-linked [Source:HGNC Symbol;Acc:HGNC:12667]
0.372049616213937	0.0108700528168806	COL11A2	1302	collagen, type XI, alpha 2 [Source:HGNC Symbol;Acc:HGNC:2187]
0.371788607167316	0.0525029633310375	FAM215B	23591	family with sequence similarity 215, member B (non-protein coding) [Source:HGNC Symbol;Acc:HGNC:43639]
0.371636197440477	0.0135014992826649	RNF222	643904	ring finger protein 222 [Source:HGNC Symbol;Acc:HGNC:34517]
0.371495976183903	0.059541897082993	ZNF252P-AS	286103	ZNF252P antisense RNA 1 [Source:HGNC Symbol;Acc:HGNC:27821]
0.371258127389708	0.18197137595853	SNORA51	677831	small nucleolar RNA, H/ACA box 51 [Source:HGNC Symbol;Acc:HGNC:32644]
0.370984855591394	0.0982359703808767	C3orf65	646600	chromosome 3 open reading frame 65 [Source:HGNC Symbol;Acc:HGNC:32674]
0.370980427619082	0.00582688392201298	OVOL3	728361	ovo-like zinc finger 3 [Source:HGNC Symbol;Acc:HGNC:14186]
0.370918324077584	0.0474053245387013	GNAZ	2781	guanine nucleotide binding protein (G protein), alpha z polypeptide [Source:HGNC Symbol;Acc:HGNC:4395]
0.370888481931911	0.0577262281611025	CDHR4	389118	cadherin-related family member 4 [Source:HGNC Symbol;Acc:HGNC:34527]
0.370812874543744	0.124529293199987	DPPA5	340168	developmental pluripotency associated 5 [Source:HGNC Symbol;Acc:HGNC:19201]
0.370580407164908	0.174330436200555	SLC2A4	6517	solute carrier family 2 (facilitated glucose transporter), member 4 [Source:HGNC Symbol;Acc:HGNC:11009]
0.370528285657767	0.0568919216760258	TRAIP	10293	TRAF interacting protein [Source:HGNC Symbol;Acc:HGNC:30764]
0.370245375854345	0.160212291103727		401509	zinc finger protein 658B, pseudogene [Source:EntrezGene;Acc:401509]
0.370230703336851	0.0329084143995814	CCDC85B	11007	coiled-coil domain containing 85B [Source:HGNC Symbol;Acc:HGNC:24926]
0.370131796526446	0.179205759199301	TINCR	257000	tissue differentiation-inducing non-protein coding RNA [Source:HGNC Symbol;Acc:HGNC:14607]
0.369932020987289	0.150443369796181	CELA3A	10136	chymotrypsin-like elastase family, member 3A [Source:HGNC Symbol;Acc:HGNC:15944]
0.369670649959728	0.00587216209840831	OR2W3	343171	olfactory receptor, family 2, subfamily W, member 3 [Source:HGNC Symbol;Acc:HGNC:15021]
0.369547903658879	0.119440700142046	XXYLT1	152002	xyloside xylosyltransferase 1 [Source:HGNC Symbol;Acc:HGNC:26639]
0.369367734760085	0.0671287991263364	GSN-AS1	57000	GSN antisense RNA 1 [Source:HGNC Symbol;Acc:HGNC:23372]
0.369290003231645	0.117144067117838	DACH2	117154	dachshund family transcription factor 2 [Source:HGNC Symbol;Acc:HGNC:16814]
0.369103747287372	0.0556975169759838	ERVW-1	30816	endogenous retrovirus group W, member 1 [Source:HGNC Symbol;Acc:HGNC:13525]
0.368895940166826	0.0813189338936266	MAPK8IP3	23162	mitogen-activated protein kinase 8 interacting protein 3 [Source:HGNC Symbol;Acc:HGNC:6884]
0.368623710560789	0.138013946859147	NCR2	9436	natural cytotoxicity triggering receptor 2 [Source:HGNC Symbol;Acc:HGNC:6732]
0.368448168422217	0.0930758989589104	IGSF10	285313	immunoglobulin superfamily, member 10 [Source:HGNC Symbol;Acc:HGNC:26384]
0.368332944651726	0.138544279342837	SEC14L4	284904	SEC14-like 4 (S. cerevisiae) [Source:HGNC Symbol;Acc:HGNC:20627]
0.368318065736222	5.27877868978274e-05	FOXG1	2290	forkhead box G1 [Source:HGNC Symbol;Acc:HGNC:3811]
0.368312588847552	0.143390687940338	KCNK16	83795	potassium channel, subfamily K, member 16 [Source:HGNC Symbol;Acc:HGNC:14464]
0.368267595612448	0.161725086785676	VAX2	25806	ventral anterior homeobox 2 [Source:HGNC Symbol;Acc:HGNC:12661]
0.368169986856073	0.0385997556556736	KRTAP1-3	81850	keratin associated protein 1-3 [Source:HGNC Symbol;Acc:HGNC:16771]
0.36804066069775	0.19055005723845	TMEM106A	113277	transmembrane protein 106A [Source:HGNC Symbol;Acc:HGNC:28288]
0.368015116535446	0.0717587018836259	GADL1	339896	glutamate decarboxylase-like 1 [Source:HGNC Symbol;Acc:HGNC:27949]
0.367954465016577	0.0972789793742351	LTC4S	4056	leukotriene C4 synthase [Source:HGNC Symbol;Acc:HGNC:6719]
0.367933402908315	0.171493890190553	KLK6	5653	kallikrein-related peptidase 6 [Source:HGNC Symbol;Acc:HGNC:6367]
0.36790171535659	0.024136516362483	PERM1	84808	PPARGC1 and ESRR induced regulator, muscle 1 [Source:HGNC Symbol;Acc:HGNC:28208]
0.367880161265831	0.0569076906946614	C14orf183	196913	chromosome 14 open reading frame 183 [Source:HGNC Symbol;Acc:HGNC:27285]
0.367860866615443	0.044346565886095	USP35	57558	ubiquitin specific peptidase 35 [Source:HGNC Symbol;Acc:HGNC:20061]
0.367736415044255	0.0934056281910503	TTLL10	254173	tubulin tyrosine ligase-like family, member 10 [Source:HGNC Symbol;Acc:HGNC:26693]
0.367588718483904	0.0941361039348029	COX4I2	84701	cytochrome c oxidase subunit IV isoform 2 (lung) [Source:HGNC Symbol;Acc:HGNC:16232]
0.367570547291769	0.103540708815164		102724384	
0.36729914697763	0.022309345784063	MDC1	9656	mediator of DNA-damage checkpoint 1 [Source:HGNC Symbol;Acc:HGNC:21163]
0.367023598399235	0.0505948399986789	PPOX	5498	protoporphyrinogen oxidase [Source:HGNC Symbol;Acc:HGNC:9280]
0.366902429199302	0.0102251274507563	CYP2F1	1572	cytochrome P450, family 2, subfamily F, polypeptide 1 [Source:HGNC Symbol;Acc:HGNC:2632]
0.366739312966877	0.148786061197679	GLS2	27165	glutaminase 2 (liver, mitochondrial) [Source:HGNC Symbol;Acc:HGNC:29570]
0.366610608192891	0.12222137254396 8	C11orf86	254439	chromosome 11 open reading frame 86 [Source:HGNC Symbol;Acc:HGNC:34442]
0.366399828683161	0.0165980757773916	C17orf102	400591	chromosome 17 open reading frame 102 [Source:HGNC Symbol;Acc:HGNC:34412]
0.36635167012159	0.150919311422595	GGACT	87769	gamma-glutamylamine cyclotransferase [Source:HGNC Symbol;Acc:HGNC:25100]
0.366198854511006	0.0531270334142311	CHEK2	11200	checkpoint kinase 2 [Source:HGNC Symbol;Acc:HGNC:16627]
0.366069077796238	0.050678797977592	MORN3	283385	MORN repeat containing 3 [Source:HGNC Symbol;Acc:HGNC:29807]
0.365966975896441	0.0563921740929237	MBLAC1	255374	metallo-beta-lactamase domain containing 1 [Source:HGNC Symbol;Acc:HGNC:22180]
0.365786359890336	0.0211797196907839		348303	selenoprotein V [Source:EntrezGene;Acc:348303]
0.365314662639927	0.0472543187926818	ZNF530	348327	zinc finger protein 530 [Source:HGNC Symbol;Acc:HGNC:29297]
0.365254196710716	0.0943933988022456	PALM3	342979	paralemmin 3 [Source:HGNC Symbol;Acc:HGNC:33274]
0.365120123468807	0.198317573298498	TCL6	27004	T-cell leukemia/lymphoma 6 (non-protein coding) [Source:HGNC Symbol;Acc:HGNC:13463]
0.365057770051767	0.186630268826993	RNVU1-15	101954267	RNA, variant U1 small nuclear 15 [Source:HGNC Symbol;Acc:HGNC:48320]
0.364770894099807	0.114395752876365	MAGEA10	4109	melanoma antigen family A, 10 [Source:HGNC Symbol;Acc:HGNC:6797]
0.364735294136253	0.171192056478741	PLEKHO1	51177	pleckstrin homology domain containing, family O member 1 [Source:HGNC Symbol;Acc:HGNC:24310]
0.364615996972262	0.0875363686676196	OTOP3	347741	otopetrin 3 [Source:HGNC Symbol;Acc:HGNC:19658]
0.364551351996945	0.0887097990454417	WDFY2	115825	WD repeat and FYVE domain containing 2 [Source:HGNC Symbol;Acc:HGNC:20482]
0.3643553449186	0.0524959048141686	SSMEM1	136263	serine-rich single-pass membrane protein 1 [Source:HGNC Symbol;Acc:HGNC:29580]
0.364276424104559	0.118175575388399	FMO3	2328	flavin containing monooxygenase 3 [Source:HGNC Symbol;Acc:HGNC:3771]
0.364229957774002	0.133790670406241	TNFRSF13B	23495	tumor necrosis factor receptor superfamily, member 13B [Source:HGNC Symbol;Acc:HGNC:18153]
0.363917088684068	0.00483616848622121		79922	rRNA methyltransferase 1, mitochondrial [Source:UniProtKB/Swiss-Prot;Acc:Q6IN84]
0.363896446520418	0.10968256053804	SLC14A2	8170	solute carrier family 14 (urea transporter), member 2 [Source:HGNC Symbol;Acc:HGNC:10919]
0.363501801707964	0.144028112269901	BCL7A	605	B-cell CLL/lymphoma 7A [Source:HGNC Symbol;Acc:HGNC:1004]
0.363384478036398	0.124743123664628	CCDC116	164592	coiled-coil domain containing 116 [Source:HGNC Symbol;Acc:HGNC:26688]
0.363380016553212	0.188842734040715	NEK2	4751	NIMA-related kinase 2 [Source:HGNC Symbol;Acc:HGNC:7745]
0.363352714744732	0.029196621375305	RIC3	79608	RIC3 acetylcholine receptor chaperone [Source:HGNC Symbol;Acc:HGNC:30338]
0.363280027530124	0.0801903079601381	ZNF430	80264	zinc finger protein 430 [Source:HGNC Symbol;Acc:HGNC:20808]
0.363150272386205	0.129940382766598	ASB11	140456	ankyrin repeat and SOCS box containing 11, E3 ubiquitin protein ligase [Source:HGNC Symbol;Acc:HGNC:17186]
0.362832350605392	0.160973804052194	PANX2	56666	pannexin 2 [Source:HGNC Symbol;Acc:HGNC:8600]
0.362823791990638	0.153847774704628	MESP2	145873	mesoderm posterior basic helix-loop-helix transcription factor 2 [Source:HGNC Symbol;Acc:HGNC:29659]
0.362675519280363	0.096090180669792	PRKCQ	5588	protein kinase C, theta [Source:HGNC Symbol;Acc:HGNC:9410]
0.362640957863495	0.0667484271512587	C17orf105	284067	chromosome 17 open reading frame 105 [Source:HGNC Symbol;Acc:HGNC:37241]

0.362594263635136	0.0931470354079679	SSUH2	51066	ssu-2 homolog (C. elegans) [Source:HGNC Symbol;Acc:HGNC:24809]
0.36236731749518	0.024373743119664	CCT8L2	150160	chaperonin containing TCP1, subunit 8 (theta)-like 2 [Source:HGNC Symbol;Acc:HGNC:15553]
0.362218726526163	0.0561108382787221	SOX3	6658	SRY (sex determining region Y)-box 3 [Source:HGNC Symbol;Acc:HGNC:11199]
0.362056081931324	0.156890887575697	TMIE	259236	transmembrane inner ear [Source:HGNC Symbol;Acc:HGNC:30800]
0.361524304164589	0.0379079861313892	CDH26	60437	cadherin 26 [Source:HGNC Symbol;Acc:HGNC:15902]
0.361451453422222	0.083162216637516	SLC22A6	9356	solute carrier family 22 (organic anion transporter), member 6 [Source:HGNC Symbol;Acc:HGNC:10970]
0.361450054061641	0.111451844923472	PON1	5444	paraoxonase 1 [Source:HGNC Symbol;Acc:HGNC:9204]
0.361356647455333	0.0712380624934874	F2RL3	9002	coagulation factor II (thrombin) receptor-like 3 [Source:HGNC Symbol;Acc:HGNC:3540]
0.36128686264171	0.153758707073548	REG1A	5967	regenerating islet-derived 1 alpha [Source:HGNC Symbol;Acc:HGNC:9951]
0.361086728877446	0.193756908794063	SEC14L2	23541	SEC14-like 2 (S. cerevisiae) [Source:HGNC Symbol;Acc:HGNC:10699]
0.360954472781086	0.0291173783491726	CAMK2B	816	calcium/calmodulin-dependent protein kinase II beta [Source:HGNC Symbol;Acc:HGNC:1461]
0.360726169136348	0.0671983317097566	IL36A	27179	interleukin 36, alpha [Source:HGNC Symbol;Acc:HGNC:15562]
0.360154300981962	0.0992950090296244	FGF18	8817	fibroblast growth factor 18 [Source:HGNC Symbol;Acc:HGNC:3674]
0.360088547201748	0.00775093042152702	KATNA1	11104	katanin p60 (ATPase containing) subunit A 1 [Source:HGNC Symbol;Acc:HGNC:6216]
0.359905614464924	0.0843105631564144	TMEM208	29100	transmembrane protein 208 [Source:HGNC Symbol;Acc:HGNC:25015]
0.35989500416959	0.039766046881272	CACNG2	10369	calcium channel, voltage-dependent, gamma subunit 2 [Source:HGNC Symbol;Acc:HGNC:1406]
0.359849337110144	0.0267632215987013	MCEMP1	199675	mast cell-expressed membrane protein 1 [Source:HGNC Symbol;Acc:HGNC:27291]
0.35978262170362	0.0591550283408587	CLASRP	11129	CLK4-associating serine/arginine rich protein [Source:HGNC Symbol;Acc:HGNC:17731]
0.359763636559643	0.174635001550436	SH2D2A	9047	SH2 domain containing 2A [Source:HGNC Symbol;Acc:HGNC:10821]
0.359736500017041	0.0434217801455725	HOXA3	3200	homeobox A3 [Source:HGNC Symbol;Acc:HGNC:5104]
0.359698001581076	0.101596413572857	SLC1A2	6506	solute carrier family 1 (glial high affinity glutamate transporter), member 2 [Source:HGNC Symbol;Acc:HGNC:10940]
0.359641011488211	0.10540458055358	SIGLEC10	89790	sialic acid binding Ig-like lectin 10 [Source:HGNC Symbol;Acc:HGNC:15620]
0.359586227586533	0.145629586621807	TCP10L2	102724205	t-complex 10-like 2 [Source:HGNC Symbol;Acc:HGNC:21254]
0.359570537093654	0.190686117183825	PTCHD1	139411	patched domain containing 1 [Source:HGNC Symbol;Acc:HGNC:26392]
0.359491282597499	0.194735066372793	MIR331	442903	microRNA 331 [Source:HGNC Symbol;Acc:HGNC:31772]
0.359219753805699	0.0294726921829021	AADACL3	126767	arylacetamide deacetylase-like 3 [Source:HGNC Symbol;Acc:HGNC:32037]
0.359031926810326	0.0631458569762234	C19orf67	646457	chromosome 19 open reading frame 67 [Source:HGNC Symbol;Acc:HGNC:34354]
0.359027174262267	0.0266792929915696	PLXNB3	5365	plexin B3 [Source:HGNC Symbol;Acc:HGNC:9105]
0.358898905128912	0.153834809855032	CFAP43	80217	cilia and flagella associated protein 43 [Source:HGNC Symbol;Acc:HGNC:26684]
0.35870416379731	0.034155880910082	AKIRIN2	55122	akirin 2 [Source:HGNC Symbol;Acc:HGNC:21407]
0.358513415325849	0.0808021252924711	ZMYND12	84217	zinc finger, MYND-type containing 12 [Source:HGNC Symbol;Acc:HGNC:21192]
0.358215036258664	0.0608922097654168	ZNF648	127665	zinc finger protein 648 [Source:HGNC Symbol;Acc:HGNC:18190]
0.35813908376848	0.147952154450442	LMNB1	4001	lamin B1 [Source:HGNC Symbol;Acc:HGNC:6637]
0.358107478126427	0.0138630028051455	SYNGR3	9143	synaptogyrin 3 [Source:HGNC Symbol;Acc:HGNC:11501]
0.358083141859602	0.0110073110558832	KCNQ3	3786	potassium voltage-gated channel, KQT-like subfamily, member 3 [Source:HGNC Symbol;Acc:HGNC:6297]
0.357983856199976	0.0386308186704692	OTULIN	90268	OTU deubiquitinase with linear linkage specificity [Source:HGNC Symbol;Acc:HGNC:25118]
0.357666819171279	0.0379003040749683	EZH2	2146	enhancer of zeste 2 polycomb repressive complex 2 subunit [Source:HGNC Symbol;Acc:HGNC:3527]
0.357450376831345	0.0716854898650655	CEP72	55722	centrosomal protein 72kDa [Source:HGNC Symbol;Acc:HGNC:25547]
0.357244459176937	0.111512145876225	ZNF225	7768	zinc finger protein 225 [Source:HGNC Symbol;Acc:HGNC:13018]
0.357212526219189	0.109272639531432	HMX3	340784	H6 family homeobox 3 [Source:HGNC Symbol;Acc:HGNC:5019]
0.356954641837185	0.111519015324098	ALX3	257	ALX homeobox 3 [Source:HGNC Symbol;Acc:HGNC:449]
0.356916788849028	0.114757765234001	RSG1	79363	REM2 and RAB-like small GTPase 1 [Source:HGNC Symbol;Acc:HGNC:28127]
0.356866276861413	0.0388820959565111	TMEM179	388021	transmembrane protein 179 [Source:HGNC Symbol;Acc:HGNC:20137]
0.35679146839803	0.112312446058157	LINC00477	144360	long intergenic non-protein coding RNA 477 [Source:HGNC Symbol;Acc:HGNC:26557]
0.356484603002801	0.121696157104508	C10orf126	283080	chromosome 10 open reading frame 126 [Source:HGNC Symbol;Acc:HGNC:28693]
0.35635349612241	0.0892955872760909	SEC14L3	266629	SEC14-like 3 (S. cerevisiae) [Source:HGNC Symbol;Acc:HGNC:18655]
0.355744151601923	0.0497821772537256	FGF8	2253	fibroblast growth factor 8 (androgen-induced) [Source:HGNC Symbol;Acc:HGNC:3686]
0.355648879522418	0.17227870432669	KIFC2	90990	kinesin family member C2 [Source:HGNC Symbol;Acc:HGNC:29530]
0.35543515836638	0.123743762388315	LY6G5C	80741	lymphocyte antigen 6 complex, locus G5C [Source:HGNC Symbol;Acc:HGNC:13932]
0.355105782829538	0.0768234824744399	MIR212	406994	microRNA 212 [Source:HGNC Symbol;Acc:HGNC:31589]
0.355083276437189	0.0760478127327344	SMYD5	10322	SMYD family member 5 [Source:HGNC Symbol;Acc:HGNC:16258]
0.354959241986331	0.0561736821319382	TOMM20L	387990	translocase of outer mitochondrial membrane 20 homolog (yeast)-like [Source:HGNC Symbol;Acc:HGNC:33752]
0.354936030129034	0.180302609163791		388282	
0.354878315066516	0.177996070054811	SLC32A1	140679	solute carrier family 32 (GABA vesicular transporter), member 1 [Source:HGNC Symbol;Acc:HGNC:11018]
0.354874343441965	0.10948295807615	SNORA70C	100124538	small nucleolar RNA, H/ACA box 70C [Source:HGNC Symbol;Acc:HGNC:33619]
0.354800126615081	0.0374944790352914	UBE2DNL	100131816	ubiquitin-conjugating enzyme E2D N-terminal like (pseudogene) [Source:HGNC Symbol;Acc:HGNC:28656]
0.354713424093414	0.00113597315380882	C16orf3	750	chromosome 16 open reading frame 3 [Source:HGNC Symbol;Acc:HGNC:1197]
0.354597322579332	0.0235202290010216	HYI	81888	hydroxypyruvate isomerase (putative) [Source:HGNC Symbol;Acc:HGNC:26948]
0.35456624706895	0.0962094090624016	FAM3B	54097	family with sequence similarity 3, member B [Source:HGNC Symbol;Acc:HGNC:1253]
0.354504126199126	0.139080067338704	C20orf144	128864	chromosome 20 open reading frame 144 [Source:HGNC Symbol;Acc:HGNC:16137]
0.354499092785108	0.0261517370765307	AWAT1	158833	acyl-CoA wax alcohol acyltransferase 1 [Source:HGNC Symbol;Acc:HGNC:23252]
0.354220913961135	0.13743882541349	SLC51A	200931	solute carrier family 51, alpha subunit [Source:HGNC Symbol;Acc:HGNC:29955]
0.354127657843428	0.0575524051778081	MIR33A	407039	microRNA 33a [Source:HGNC Symbol;Acc:HGNC:31634]
0.354098397333102	0.0521145733360109	MAP6D1	79929	MAP6 domain containing 1 [Source:HGNC Symbol;Acc:HGNC:25753]
0.354070638721261	0.0867628100119246	OR4S2	219431	olfactory receptor, family 4, subfamily S, member 2 [Source:HGNC Symbol;Acc:HGNC:15183]
0.353926505234896	0.0521441089866216	C12orf76	400073	chromosome 12 open reading frame 76 [Source:HGNC Symbol;Acc:HGNC:33790]
0.353834989777788	0.171114816110859	PEX10	5192	peroxisomal biogenesis factor 10 [Source:HGNC Symbol;Acc:HGNC:8851]
0.353188927143597	0.128024341834633	LYNX1	66004	Ly6/neurotoxin 1 [Source:HGNC Symbol;Acc:HGNC:29604]
0.35310931260677	0.155034934883101	OR9K2	441639	olfactory receptor, family 9, subfamily K, member 2 [Source:HGNC Symbol;Acc:HGNC:15339]
0.353044989850727	0.0327254464102926	CDK5R1	8851	cyclin-dependent kinase 5, regulatory subunit 1 (p35) [Source:HGNC Symbol;Acc:HGNC:1775]
0.35286564930607	0.0161831156412573	TEDDM1	127670	transmembrane epididymal protein 1 [Source:HGNC Symbol;Acc:HGNC:30233]
0.352681908626719	0.000565333156682353	IRX5	10265	iroquois homeobox 5 [Source:HGNC Symbol;Acc:HGNC:14361]
0.352667912703656	0.0422012978396691	PRKACG	5568	protein kinase, cAMP-dependent, catalytic, gamma [Source:HGNC Symbol;Acc:HGNC:9382]
0.352657696687056	0.17840511163138	SNORD115-2	100036563	small nucleolar RNA, C/D box 115-24 [Source:HGNC Symbol;Acc:HGNC:33043]
0.352517792702537	0.122043909297468	SNX32	254122	sorting nexin 32 [Source:HGNC Symbol;Acc:HGNC:26423]
0.352516721164137	0.191002399387672	CYP2C19	1557	cytochrome P450, family 2, subfamily C, polypeptide 19 [Source:HGNC Symbol;Acc:HGNC:2621]
0.352337721975077	0.0245797514065676	GRK7	131890	G protein-coupled receptor kinase 7 [Source:HGNC Symbol;Acc:HGNC:17031]
0.35223320616687	0.152246263693406	SUSD5	26032	sushi domain containing 5 [Source:HGNC Symbol;Acc:HGNC:29061]
0.352169295774678	0.0428272170113305	CCDC129	223075	coiled-coil domain containing 129 [Source:HGNC Symbol;Acc:HGNC:27363]
0.352142834481075	0.074695976055655	RAMP2-AS1	100190938	RAMP2 antisense RNA 1 [Source:HGNC Symbol;Acc:HGNC:44358]
0.352008500184325	0.174026169252857	ZNF517	340385	zinc finger protein 517 [Source:HGNC Symbol;Acc:HGNC:27984]
0.351977771752677	0.178614059904609	C5orf52	100190949	chromosome 5 open reading frame 52 [Source:HGNC Symbol;Acc:HGNC:35121]
0.351931969550695	0.122650054010829	SRP19	6728	signal recognition particle 19kDa [Source:HGNC Symbol;Acc:HGNC:11300]
0.351919766055234	0.157754796071689		407002	hsa-mir-219a-1 [Source:miRBase;Acc:MI0000296]
0.35181089405115	0.031468438285657	PHF21B	112885	PHD finger protein 21B [Source:HGNC Symbol;Acc:HGNC:25161]
0.351748018300637	0.157213477711585	AZU1	566	azurocidin 1 [Source:HGNC Symbol;Acc:HGNC:913]
0.351586904532664	0.154374217617949	LINC00173	100287569	long intergenic non-protein coding RNA 173 [Source:HGNC Symbol;Acc:HGNC:33791]
0.35155227866395	0.103993374715023	IGLL1	3543	immunoglobulin lambda-like polypeptide 1 [Source:HGNC Symbol;Acc:HGNC:5870]
0.351543458441915	0.0422087873565982	PRSS38	339501	protease, serine, 38 [Source:HGNC Symbol;Acc:HGNC:29625]
0.351235621236982	0.129137805829039	FGFBP2	83888	fibroblast growth factor binding protein 2 [Source:HGNC Symbol;Acc:HGNC:29451]
0.351164751700313	0.0159994045559021	MMP7	4316	matrix metallopeptidase 7 (matrilysin, uterine) [Source:HGNC Symbol;Acc:HGNC:7174]
0.351132144249362	0.114200575738677	COL26A1	136227	collagen, type XXVI, alpha 1 [Source:HGNC Symbol;Acc:HGNC:18038]
0.35092583564498	0.0134838816783682	CMTM2	146225	CKLF-like MARVEL transmembrane domain containing 2 [Source:HGNC Symbol;Acc:HGNC:19173]
0.350676723439867	0.107163482038867	SNORD83B	116938	small nucleolar RNA, C/D box 83B [Source:HGNC Symbol;Acc:HGNC:17132]
0.350564895084438	0.0253256436205887	PNN	5411	pinin, desmosome associated protein [Source:HGNC Symbol;Acc:HGNC:9162]
0.350518264346799	0.00234002428870824	GDNF	2668	glial cell derived neurotrophic factor [Source:HGNC Symbol;Acc:HGNC:4232]
0.350377500456812	0.0418742650004125	MESP1	55897	mesoderm posterior basic helix-loop-helix transcription factor 1 [Source:HGNC Symbol;Acc:HGNC:29658]
0.350177561627892	0.0765286079066102	TBC1D29	26083	TBC1 domain family, member 29 [Source:HGNC Symbol;Acc:HGNC:24509]
0.349967217598815	0.0776140092336418	KIF21B	23046	kinesin family member 21B [Source:HGNC Symbol;Acc:HGNC:29442]
0.349899782567451	0.00639090614531765		284573	
0.349534232292387	0.101625586930708	LINC00982	440556	long intergenic non-protein coding RNA 982 [Source:HGNC Symbol;Acc:HGNC:48664]
0.349409511038024	0.190930346987959	TEX19	400629	testis expressed 19 [Source:HGNC Symbol;Acc:HGNC:33802]
0.34908960969469	0.148680897816163	MS4A3	932	membrane-spanning 4-domains, subfamily A, member 3 (hematopoietic cell-specific) [Source:HGNC Symbol;Acc:HGNC:7317]
0.349086854361141	0.112150082501155	SNORD61	26787	small nucleolar RNA, C/D box 61 [Source:HGNC Symbol;Acc:HGNC:10218]
0.349086242759146	0.0417951254193301	OR6B3	150681	olfactory receptor, family 6, subfamily B, member 3 [Source:HGNC Symbol;Acc:HGNC:15042]
0.349073155243988	0.15876214589984	OR10P1	121130	olfactory receptor, family 10, subfamily P, member 1 [Source:HGNC Symbol;Acc:HGNC:15378]
0.348738353638391	0.199777031230133	MTHFD2	10797	methylenetetrahydrofolate dehydrogenase (NADP+ dependent) 2, methenyltetrahydrofolate cyclohydrolase [Source:HGNC Symbol;Acc:HGNC:7434]
0.348695707139858	0.178082111660717	RMI2	116028	RecQ mediated genome instability 2 [Source:HGNC Symbol;Acc:HGNC:28349]
0.348187596508696	0.0126229486919994	RAD9A	5883	RAD9 homolog A (S. pombe) [Source:HGNC Symbol;Acc:HGNC:9827]
0.348170145709958	0.103346094312118	CENPL	91687	centromere protein L [Source:HGNC Symbol;Acc:HGNC:17879]
0.347971963658892	0.0266483919207652	MCTS1	28985	malignant T cell amplified sequence 1 [Source:HGNC Symbol;Acc:HGNC:23357]
0.347958428444919	0.0711283397187487	KRTAP12-3	386683	keratin associated protein 12-3 [Source:HGNC Symbol;Acc:HGNC:20531]
0.347907508063263	0.138495193399497	NHLRC1	378884	NHL repeat containing E3 ubiquitin protein ligase 1 [Source:HGNC Symbol;Acc:HGNC:21576]
0.347658072668196	0.115221264588494	ZNF283	284349	zinc finger protein 283 [Source:HGNC Symbol;Acc:HGNC:13077]
0.347637001360575	0.0653401968102106	MYLK3	91807	myosin light chain kinase 3 [Source:HGNC Symbol;Acc:HGNC:29826]
0.347527025409535	0.0120546755900204	TMEM52B	120939	transmembrane protein 52B [Source:HGNC Symbol;Acc:HGNC:26438]
0.347300868286263	0.0391097332638587	ZNF519	162655	zinc finger protein 519 [Source:HGNC Symbol;Acc:HGNC:30574]
0.347229071349826	0.143224907701924	ULBP2	80328	UL16 binding protein 2 [Source:HGNC Symbol;Acc:HGNC:14894]
0.346860064531569	0.0384952856920263	NKPD1	284353	NTPase, KAP family P-loop domain containing 1 [Source:HGNC Symbol;Acc:HGNC:24739]
0.346752402161266	0.0176820021375985		100507530	
0.346741433142546	0.0809702837599344	ADAMTS14	140766	ADAM metallopeptidase with thrombospondin type 1 motif, 14 [Source:HGNC Symbol;Acc:HGNC:14899]
0.346668989481953	0.0224205634249122	SHISA6	388336	shisa family member 6 [Source:HGNC Symbol;Acc:HGNC:34491]
0.346627257222304	0.114102868345278	TRIM45	80263	tripartite motif containing 45 [Source:HGNC Symbol;Acc:HGNC:19018]
0.34639567634798	0.188418253358196	SLC29A2	3177	solute carrier family 29 (equilibrative nucleoside transporter), member 2 [Source:HGNC Symbol;Acc:HGNC:11004]
0.346339676584931	0.0529263533698752	THOC6	79228	THO complex 6 homolog (Drosophila) [Source:HGNC Symbol;Acc:HGNC:28369]
0.346267778301324	0.141537806697376		255180	
0.346198526183022	0.0582783765518472	CGREF1	10669	cell growth regulator with EF-hand domain 1 [Source:HGNC Symbol;Acc:HGNC:16962]
0.345808981531126	0.129107537794241	LTB	4050	lymphotoxin beta (TNF superfamily, member 3) [Source:HGNC Symbol;Acc:HGNC:6711]
0.345683036431573	0.158142198786521	LINC00482	284185	long intergenic non-protein coding RNA 482 [Source:HGNC Symbol;Acc:HGNC:26816]
0.345606367501127	0.188753122374109	EGFL6	25975	EGF-like-domain, multiple 6 [Source:HGNC Symbol;Acc:HGNC:3235]
0.344955358175474	0.0912424607383999	MIR15A	406948	microRNA 15a [Source:HGNC Symbol;Acc:HGNC:31543]
0.344791908821171	0.189312829997637	HPX	3263	hemopexin [Source:HGNC Symbol;Acc:HGNC:5171]
0.344493259381152	0.0805791151535808	GGNBP1	449520	gametogenetin binding protein 1 (pseudogene) [Source:HGNC Symbol;Acc:HGNC:19427]
0.344437582443312	0.0297645152141943	ZNF526	116115	zinc finger protein 526 [Source:HGNC Symbol;Acc:HGNC:29415]
0.344369837478936	0.169635846136796	THAP5	168451	THAP domain containing 5 [Source:HGNC Symbol;Acc:HGNC:23188]
0.343916782175485	0.104847697349409	SLC9B2	133308	solute carrier family 9, subfamily B (NHA2, cation proton antiporter 2), member 2 [Source:HGNC Symbol;Acc:HGNC:25143]
0.343896274602495	0.13942005807375	MUC7	4589	mucin 7, secreted [Source:HGNC Symbol;Acc:HGNC:7518]
0.343860964349168	0.0960870679342894	CPT1C	126129	carnitine palmitoyltransferase 1C [Source:HGNC Symbol;Acc:HGNC:18540]
0.343825856244897	0.0664995442142204	AP5Z1	9907	adaptor-related protein complex 5, zeta 1 subunit [Source:HGNC Symbol;Acc:HGNC:22197]
0.343729346940044	0.0558230086063183	ZNRF4	148066	zinc and ring finger 4 [Source:HGNC Symbol;Acc:HGNC:17726]
0.343425084829778	0.0943258325332553	SGCA	6442	sarcoglycan, alpha (50kDa dystrophin-associated glycoprotein) [Source:HGNC Symbol;Acc:HGNC:10805]

0.343290030802389	0.175180866379573		257357	
0.343217567648972	0.195076728417131		729164	
0.343094661647488	0.155163812394526	C5orf45	51149	chromosome 5 open reading frame 45 [Source:HGNC Symbol;Acc:HGNC:30817]
0.342907484548392	0.166340172708661	SNORA67	26781	small nucleolar RNA, H/ACA box 67 [Source:HGNC Symbol;Acc:HGNC:10224]
0.342886882359521	0.0107992129426133	LHX2	9355	LIM homeobox 2 [Source:HGNC Symbol;Acc:HGNC:6594]
0.342613626693109	0.142391597718586	MCM5	4174	minichromosome maintenance complex component 5 [Source:HGNC Symbol;Acc:HGNC:6948]
0.342598518077458	0.17370702545353		285961	septin 7 pseudogene 9 [Source:EntrezGene;Acc:285961]
0.342425061388429	0.126267719781613	CENPM	79019	centromere protein M [Source:HGNC Symbol;Acc:HGNC:18352]
0.342134522876974	0.0635997345002049	SOX2	6657	SRY (sex determining region Y)-box 2 [Source:HGNC Symbol;Acc:HGNC:11195]
0.341889398888669	0.158953657708017	NFYC-AS1	100130557	NFYC antisense RNA 1 [Source:HGNC Symbol;Acc:HGNC:49451]
0.341888230927195	0.0200564612966845	MTL5	9633	metallothionein-like 5, testis-specific (tesmin) [Source:HGNC Symbol;Acc:HGNC:7446]
0.341722849142566	0.127748808064389	DLX2	1746	distal-less homeobox 2 [Source:HGNC Symbol;Acc:HGNC:2915]
0.341722443226103	0.0428007542790605	PDYN	5173	prodynorphin [Source:HGNC Symbol;Acc:HGNC:8820]
0.341663346172879	0.0472028139072735	CHRNE	1145	cholinergic receptor, nicotinic, epsilon (muscle) [Source:HGNC Symbol;Acc:HGNC:1966]
0.341596240955638	0.189278307789797	STK33	65975	serine/threonine kinase 33 [Source:HGNC Symbol;Acc:HGNC:14568]
0.34146219232058	0.193658016708599	NCR3	259197	natural cytotoxicity triggering receptor 3 [Source:HGNC Symbol;Acc:HGNC:19077]
0.341390355079231	0.167611893095861	FOXQ1	94234	forkhead box Q1 [Source:HGNC Symbol;Acc:HGNC:20951]
0.34098291766681	0.013411939946315	GRPR	2925	gastrin-releasing peptide receptor [Source:HGNC Symbol;Acc:HGNC:4609]
0.340944360938628	0.0802753207023089	CCNO	10309	cyclin O [Source:HGNC Symbol;Acc:HGNC:18576]
0.340923636196588	0.143672497943124	TMEM145	284339	transmembrane protein 145 [Source:HGNC Symbol;Acc:HGNC:26912]
0.340559818898047	0.149839615346722	DTX1	1840	deltex 1, E3 ubiquitin ligase [Source:HGNC Symbol;Acc:HGNC:3060]
0.34023747641097	0.0686937034189208	COLEC11	78989	collectin sub-family member 11 [Source:HGNC Symbol;Acc:HGNC:17213]
0.340232003711	0.164990239315294	TRIP13	9319	thyroid hormone receptor interactor 13 [Source:HGNC Symbol;Acc:HGNC:12307]
0.340153379821843	0.0276119815046309	CCDC110	256309	coiled-coil domain containing 110 [Source:HGNC Symbol;Acc:HGNC:28504]
0.339988933814397	0.00707650825765271	SERTM1	400120	serine-rich and transmembrane domain containing 1 [Source:HGNC Symbol;Acc:HGNC:33792]
0.339862737853692	0.0663953182932489	RDH12	145226	retinol dehydrogenase 12 (all-trans/9-cis/11-cis) [Source:HGNC Symbol;Acc:HGNC:19977]
0.339855784965978	0.0673539082477974	TRIM72	493829	tripartite motif containing 72, E3 ubiquitin protein ligase [Source:HGNC Symbol;Acc:HGNC:32671]
0.339771436257084	0.156754822215298	COL9A1	1297	collagen, type IX, alpha 1 [Source:HGNC Symbol;Acc:HGNC:2217]
0.339514583185429	0.114166883674187	TNFRSF8	943	tumor necrosis factor receptor superfamily, member 8 [Source:HGNC Symbol;Acc:HGNC:11923]
0.339489688158618	0.153204215081512	GRIA1	2890	glutamate receptor, ionotropic, AMPA 1 [Source:HGNC Symbol;Acc:HGNC:4571]
0.339435182796211	0.0593498473207327	B4GALNT1	2583	beta-1,4-N-acetyl-galactosaminyl transferase 1 [Source:HGNC Symbol;Acc:HGNC:4117]
0.338828472610234	0.0739580790847472	KLK2	3817	kallikrein-related peptidase 2 [Source:HGNC Symbol;Acc:HGNC:6363]
0.338622819196496	0.0382765258800094	ENDOG	2021	endonuclease G [Source:HGNC Symbol;Acc:HGNC:3346]
0.338513880355229	0.0771262454275969	ACSM6	142827	acyl-CoA synthetase medium-chain family member 6 [Source:HGNC Symbol;Acc:HGNC:31665]
0.338456411648314	0.0266982634953377		80307	
0.338435372965127	0.00537245510036203	NR5A1	2516	nuclear receptor subfamily 5, group A, member 1 [Source:HGNC Symbol;Acc:HGNC:7983]
0.338418709788688	0.123555614651836	KLK10	5655	kallikrein-related peptidase 10 [Source:HGNC Symbol;Acc:HGNC:6358]
0.338374038841101	0.10665759116616	SERPINA6	866	serpin peptidase inhibitor, clade A (alpha-1 antiproteinase, antitrypsin), member 6 [Source:HGNC Symbol;Acc:HGNC:1540]
0.338311417706316	0.143962817955823	AGBL2	79841	ATP/GTP binding protein-like 2 [Source:HGNC Symbol;Acc:HGNC:26296]
0.33816517825929	0.114641672897159	YJEFN3	374887	YjeF N-terminal domain containing 3 [Source:HGNC Symbol;Acc:HGNC:24785]
0.338009405794346	0.105538009743789	RIPK3	11035	receptor-interacting serine-threonine kinase 3 [Source:HGNC Symbol;Acc:HGNC:10021]
0.337575893794031	0.079112803012676	PIANP	196500	PILR alpha associated neural protein [Source:HGNC Symbol;Acc:HGNC:25338]
0.337369183570769	0.0384034523026686	LARS2	23395	leucyl-tRNA synthetase 2, mitochondrial [Source:HGNC Symbol;Acc:HGNC:17095]
0.337321118458276	0.178547374618203	SPIN3	169981	spindlin family, member 3 [Source:HGNC Symbol;Acc:HGNC:27272]
0.337129898137634	0.0790632944632253	C2orf57	165100	chromosome 2 open reading frame 57 [Source:HGNC Symbol;Acc:HGNC:28563]
0.33712322377051	0.137199685017614	ZIM3	114026	zinc finger, imprinted 3 [Source:HGNC Symbol;Acc:HGNC:16366]
0.337089207341914	0.157651666098828	CXCR3	2833	chemokine (C-X-C motif) receptor 3 [Source:HGNC Symbol;Acc:HGNC:4540]
0.337012453697976	0.116103786180149	C19orf48	84798	chromosome 19 open reading frame 48 [Source:HGNC Symbol;Acc:HGNC:29667]
0.336897123165803	0.162901410283846	ZNF169	169841	zinc finger protein 169 [Source:HGNC Symbol;Acc:HGNC:12957]
0.33680732373706	0.102759770798425	ELANE	1991	elastase, neutrophil expressed [Source:HGNC Symbol;Acc:HGNC:3309]
0.33678015085824	0.1688461771769	MYOD1	4654	myogenic differentiation 1 [Source:HGNC Symbol;Acc:HGNC:7611]
0.336706485799724	0.0645939850075927	ARMC4	55130	armadillo repeat containing 4 [Source:HGNC Symbol;Acc:HGNC:25583]
0.336700750879244	0.046614587406081	TRMT1	55621	tRNA methyltransferase 1 homolog (S. cerevisiae) [Source:HGNC Symbol;Acc:HGNC:25980]
0.336575788093034	0.0745797390817258	SLC26A1	10861	solute carrier family 26 (anion exchanger), member 1 [Source:HGNC Symbol;Acc:HGNC:10993]
0.336399419450908	0.188784144745341	MUM1L1	139221	melanoma associated antigen (mutated) 1-like 1 [Source:HGNC Symbol;Acc:HGNC:26583]
0.336014170059014	0.0993445572617738	SYN3	8224	synapsin III [Source:HGNC Symbol;Acc:HGNC:11496]
0.335364244537336	0.181896465693466	MANEAL	149175	mannosidase, endo-alpha-like [Source:HGNC Symbol;Acc:HGNC:26452]
0.335358753207174	0.109616762806323	NOBOX	135935	NOBOX oogenesis homeobox [Source:HGNC Symbol;Acc:HGNC:22448]
0.335137388663446	0.115925094199718	ZNF771	51333	zinc finger protein 771 [Source:HGNC Symbol;Acc:HGNC:29653]
0.334509671535062	0.0816905833771155	SOX17	64321	SRY (sex determining region Y)-box 17 [Source:HGNC Symbol;Acc:HGNC:18122]
0.334258557300618	0.149569221294086	OR1G1	8390	olfactory receptor, family 1, subfamily G, member 1 [Source:HGNC Symbol;Acc:HGNC:8204]
0.334215493502051	0.109096607056245	KCNK4	50801	potassium channel, subfamily K, member 4 [Source:HGNC Symbol;Acc:HGNC:6279]
0.333885814142026	0.190767544943992	SV2C	22987	synaptic vesicle glycoprotein 2C [Source:HGNC Symbol;Acc:HGNC:30670]
0.33378739877008	0.199618144653881	STH	246744	saitohin [Source:HGNC Symbol;Acc:HGNC:18839]
0.3337819977937	0.190979523301905	CNTD1	124817	cyclin N-terminal domain containing 1 [Source:HGNC Symbol;Acc:HGNC:26847]
0.333781991544964	0.0129167116808641	SPINT3	10816	serine peptidase inhibitor, Kunitz type, 3 [Source:HGNC Symbol;Acc:HGNC:11248]
0.333675919142855	0.198103298673938		162083	
0.333303298511507	0.16056517528561	CDCA3	83461	cell division cycle associated 3 [Source:HGNC Symbol;Acc:HGNC:14624]
0.333013649921315	0.1031250860625	POU5F2	134187	POU domain class 5, transcription factor 2 [Source:HGNC Symbol;Acc:HGNC:26367]
0.332725243773103	0.0912332822519196	NTRK3	4916	neurotrophic tyrosine kinase, receptor, type 3 [Source:HGNC Symbol;Acc:HGNC:8033]
0.332468169875873	0.00680205536455264	SNORD115-2	100036565	small nucleolar RNA, C/D box 115-28 [Source:HGNC Symbol;Acc:HGNC:33047]
0.332429298499004	0.114745103256165	NKX6-1	4825	NK6 homeobox 1 [Source:HGNC Symbol;Acc:HGNC:7839]
0.332417214514024	0.148592620868464	IQCA1P1	102723607	IQ motif containing with AAA domain 1 pseudogene 1 [Source:HGNC Symbol;Acc:HGNC:22831]
0.332140192619533	0.150010547547791	ANKRD6	22881	ankyrin repeat domain 6 [Source:HGNC Symbol;Acc:HGNC:17280]
0.332090198897522	0.0157496076431923	ZNF335	63925	zinc finger protein 335 [Source:HGNC Symbol;Acc:HGNC:15807]
0.332082078769355	0.138729755061692	TRPC2	7221	transient receptor potential cation channel, subfamily C, member 2, pseudogene [Source:HGNC Symbol;Acc:HGNC:12334]
0.3320336696782	0.115531418979875	SRRT	51593	serrate, RNA effector molecule [Source:HGNC Symbol;Acc:HGNC:24101]
0.331958952362277	0.0850334876363739	GNRHR2	114814	gonadotropin-releasing hormone (type 2) receptor 2, pseudogene [Source:HGNC Symbol;Acc:HGNC:16341]
0.331949382078756	0.128347847542854	ZNF497	162968	zinc finger protein 497 [Source:HGNC Symbol;Acc:HGNC:23714]
0.331740664092263	0.00550780147799554	DGKA	1606	diacylglycerol kinase, alpha 80kDa [Source:HGNC Symbol;Acc:HGNC:2849]
0.331095030898709	0.160720522947634		54883	Pre-mRNA-splicing factor CWC25 homolog [Source:UniProtKB/Swiss-Prot;Acc:Q9NXE8]
0.331050019375383	0.0285925499075508	MCF2L2	23101	MCF.2 cell line derived transforming sequence-like 2 [Source:HGNC Symbol;Acc:HGNC:30319]
0.331040311429193	0.0878645690023989	MYLK4	340156	myosin light chain kinase family, member 4 [Source:HGNC Symbol;Acc:HGNC:27972]
0.330919831226552	0.107331443297148	AMH	268	anti-Mullerian hormone [Source:HGNC Symbol;Acc:HGNC:464]
0.330826602911202	0.0435493988789681	SH3GL2	6456	SH3-domain GRB2-like 2 [Source:HGNC Symbol;Acc:HGNC:10831]
0.330657902299993	0.0755091040925711		4588	Mucin 6, oligomeric mucus/gel-forming [Source:UniProtKB/TrEMBL;Acc:H9XFB7]
0.330603272932882	0.128521971139745	SIM2	6493	single-minded family bHLH transcription factor 2 [Source:HGNC Symbol;Acc:HGNC:10883]
0.330583652235453	0.0718463810830711	LECT1	11061	leukocyte cell derived chemotaxin 1 [Source:HGNC Symbol;Acc:HGNC:17005]
0.33051331113484	0.0650669414733463	PROSER3	148137	proline and serine rich 3 [Source:HGNC Symbol;Acc:HGNC:25204]
0.330485887134987	0.0695369198068732	KRBA2	124751	KRAB-A domain containing 2 [Source:HGNC Symbol;Acc:HGNC:26989]
0.330485591774813	0.0838309177461527	MMP25	64386	matrix metallopeptidase 25 [Source:HGNC Symbol;Acc:HGNC:14246]
0.33023094591595	0.161318374094432	ENO3	2027	enolase 3 (beta, muscle) [Source:HGNC Symbol;Acc:HGNC:3354]
0.330015784163543	0.121255611948948	GRAMD2	196996	GRAM domain containing 2 [Source:HGNC Symbol;Acc:HGNC:27287]
0.329651144857458	0.184848443266328		100128653	
0.329346661738109	0.0186002824477631		284788	
0.329289207278448	0.122726103767053	CHRNA4	1137	cholinergic receptor, nicotinic, alpha 4 (neuronal) [Source:HGNC Symbol;Acc:HGNC:1958]
0.32913866574554	0.137650839052401	SLURP1	57152	secreted LY6/PLAUR domain containing 1 [Source:HGNC Symbol;Acc:HGNC:18746]
0.329035235550369	0.132325482655416	NPPA	4878	natriuretic peptide A [Source:HGNC Symbol;Acc:HGNC:7939]
0.328976743579658	0.0568626363134806	GMIP	51291	GEM interacting protein [Source:HGNC Symbol;Acc:HGNC:24852]
0.328444688873694	0.0126987102983529	DMRTB1	63948	DMRT-like family B with proline-rich C-terminal, 1 [Source:HGNC Symbol;Acc:HGNC:13913]
0.328374011666969	0.153295048820061	FMNL1	752	formin-like 1 [Source:HGNC Symbol;Acc:HGNC:1212]
0.328351303635015	0.186637350925891	IGFBP1	3484	insulin-like growth factor binding protein 1 [Source:HGNC Symbol;Acc:HGNC:5469]
0.328286897842689	0.142858009176619		124923	uncharacterized serine/threonine-protein kinase SgK494 [Source:EntrezGene;Acc:124923]
0.328082503627629	0.146460272438406	C9orf171	389799	chromosome 9 open reading frame 171 [Source:HGNC Symbol;Acc:HGNC:33776]
0.328072105181041	0.0123967845941406	CCDC87	55231	coiled-coil domain containing 87 [Source:HGNC Symbol;Acc:HGNC:25579]
0.327777027495181	0.0307508361363981	DNAH11	8701	dynein, axonemal, heavy chain 11 [Source:HGNC Symbol;Acc:HGNC:2942]
0.327665535357042	0.186514026454614	PGPEP1L	145814	pyroglutamyl-peptidase I-like [Source:HGNC Symbol;Acc:HGNC:27080]
0.3275496572915	0.177650879317546	GPN3	51184	GPN-loop GTPase 3 [Source:HGNC Symbol;Acc:HGNC:30186]
0.327477886282244	0.16205185440349	DGCR5	26220	DiGeorge syndrome critical region gene 5 (non-protein coding) [Source:HGNC Symbol;Acc:HGNC:16757]
0.327323348120855	0.15154835395599	TACC3	10460	transforming, acidic coiled-coil containing protein 3 [Source:HGNC Symbol;Acc:HGNC:11524]
0.327212709361865	0.0701551961290869	SLC12A4	6560	solute carrier family 12 (potassium/chloride transporter), member 4 [Source:HGNC Symbol;Acc:HGNC:10913]
0.327077461159492	0.0846203447473607	F2	2147	coagulation factor II (thrombin) [Source:HGNC Symbol;Acc:HGNC:3535]
0.326707580505022	0.198310280976846	C14orf178	283579	chromosome 14 open reading frame 178 [Source:HGNC Symbol;Acc:HGNC:26385]
0.326392252432657	0.0535660139502251	C9orf3	84909	chromosome 9 open reading frame 3 [Source:HGNC Symbol;Acc:HGNC:1361]
0.32634167223898	0.0308824333452997	GAB4	128954	GRB2-associated binding protein family, member 4 [Source:HGNC Symbol;Acc:HGNC:18325]
0.326334334481485	0.107427078249595	EPB41L4A-AS	54508	EPB41L4A antisense RNA 2 (head to head) [Source:HGNC Symbol;Acc:HGNC:25643]
0.326207907812303	0.101268008919288	GAS1	2619	growth arrest-specific 1 [Source:HGNC Symbol;Acc:HGNC:4165]
0.326164801756077	0.091943582561191	LINC00575	439934	long intergenic non-protein coding RNA 575 [Source:HGNC Symbol;Acc:HGNC:21342]
0.326122651465139	0.0980213423628827	C8orf58	541565	chromosome 8 open reading frame 58 [Source:HGNC Symbol;Acc:HGNC:32233]
0.32597271869211	0.0814192276454577	TMEM120B	144404	transmembrane protein 120B [Source:HGNC Symbol;Acc:HGNC:32008]
0.325735920320804	0.15408262324124		100128374	
0.32562020349147	0.135661892600219	KPTN	11133	kaptin (actin binding protein) [Source:HGNC Symbol;Acc:HGNC:6404]
0.325581930036348	0.179510316065018	SPC24	147841	SPC24, NDC80 kinetochore complex component [Source:HGNC Symbol;Acc:HGNC:26913]
0.325451719352538	0.114623778845512		728283	
0.325314600047277	0.0427047261084408	MCCD1	401250	mitochondrial coiled-coil domain 1 [Source:HGNC Symbol;Acc:HGNC:20668]
0.325191236293841	0.0971444179968007	IZUMO1	284359	izumo sperm-egg fusion 1 [Source:HGNC Symbol;Acc:HGNC:28539]
0.325190644318693	0.16914240830654	COL9A3	1299	collagen, type IX, alpha 3 [Source:HGNC Symbol;Acc:HGNC:2219]
0.325153920227553	0.127920730792906	SSR4P1	728039	signal sequence receptor, delta pseudogene 1 [Source:HGNC Symbol;Acc:HGNC:23131]
0.325151225835367	0.150750738220684	UTS2R	2837	urotensin 2 receptor [Source:HGNC Symbol;Acc:HGNC:4468]
0.325078383370862	0.0403477990616153	SP7	121340	Sp7 transcription factor [Source:HGNC Symbol;Acc:HGNC:17321]
0.325054177903019	0.0492473964747419	C3orf27	23434	chromosome 3 open reading frame 27 [Source:HGNC Symbol;Acc:HGNC:17099]
0.324980622407899	0.0880576959741099	LRIT1	26103	leucine-rich repeat, immunoglobulin-like and transmembrane domains 1 [Source:HGNC Symbol;Acc:HGNC:23404]
0.324932728772307	0.18637956149095	IDI2-AS1	55853	IDI2 antisense RNA 1 [Source:HGNC Symbol;Acc:HGNC:30885]
0.324891957339881	0.113168470811301	LINC00346	283487	long intergenic non-protein coding RNA 346 [Source:HGNC Symbol;Acc:HGNC:27492]
0.324651369873442	0.134897407208618	TMEM18	129787	transmembrane protein 18 [Source:HGNC Symbol;Acc:HGNC:25257]
0.324603531789101	0.187540793202895	XAGE3	170626	X antigen family, member 3 [Source:HGNC Symbol;Acc:HGNC:14618]
0.324585808075141	0.105231627187159	C9orf50	375759	chromosome 9 open reading frame 50 [Source:HGNC Symbol;Acc:HGNC:23677]
0.324412320847526	0.182441284427931	MT3	4504	metallothionein 3 [Source:HGNC Symbol;Acc:HGNC:7408]
0.324402275590685	0.0424913661901837	MSL3	10943	male-specific lethal 3 homolog (Drosophila) [Source:HGNC Symbol;Acc:HGNC:7370]
0.323987933150559	0.137675798812301	BCL2L15	440603	BCL2-like 15 [Source:HGNC Symbol;Acc:HGNC:33624]
0.32389288092208	0.101480759110742	EOMES	8320	eomesodermin [Source:HGNC Symbol;Acc:HGNC:3372]

0.323626151001309	0.122308518952331	GIPR	2696	gastric inhibitory polypeptide receptor [Source:HGNC Symbol;Acc:HGNC:4271]
0.323545719660921	0.0629091297853981	GCK	2645	glucokinase (hexokinase 4) [Source:HGNC Symbol;Acc:HGNC:4195]
0.323537299004639	0.186643978392183	PRAP1	118471	proline-rich acidic protein 1 [Source:HGNC Symbol;Acc:HGNC:23304]
0.323470142875799	0.0222319820601849	COL23A1	91522	collagen, type XXIII, alpha 1 [Source:HGNC Symbol;Acc:HGNC:22990]
0.323080355290591	0.038247667923201	ARX	170302	aristaless related homeobox [Source:HGNC Symbol;Acc:HGNC:18060]
0.323066415760874	0.167342407495734	TNFRSF10C	8794	tumor necrosis factor receptor superfamily, member 10c, decoy without an intracellular domain [Source:HGNC Symbol;Acc:HGNC:11906]
0.322920166770756	0.195020847153307	RPL39L	116832	ribosomal protein L39-like [Source:HGNC Symbol;Acc:HGNC:17094]
0.322593933836071	0.0628296444690641	FUNDC2P2	388965	FUN14 domain containing 2 pseudogene 2 [Source:HGNC Symbol;Acc:HGNC:17247]
0.322478919356488	0.191432852914138		643210	
0.322277892143337	0.0424592997406377	C21orf119	84996	chromosome 21 open reading frame 119 [Source:HGNC Symbol;Acc:HGNC:23128]
0.322215817183171	0.157416203804696	PRSS53	339105	protease, serine, 53 [Source:HGNC Symbol;Acc:HGNC:34407]
0.32211960199287	0.125302568884757	DLX1	1745	distal-less homeobox 1 [Source:HGNC Symbol;Acc:HGNC:2914]
0.32210932392712	0.127909368976999	SIX3	6496	SIX homeobox 3 [Source:HGNC Symbol;Acc:HGNC:10889]
0.322056440421212	0.0918042031136084	MEGF11	84465	multiple EGF-like-domains 11 [Source:HGNC Symbol;Acc:HGNC:29635]
0.321968543331281	0.106463872043363	HIST1H2BO	8348	histone cluster 1, H2bo [Source:HGNC Symbol;Acc:HGNC:4758]
0.321925112384446	0.120570457843222	NR2E1	7101	nuclear receptor subfamily 2, group E, member 1 [Source:HGNC Symbol;Acc:HGNC:7973]
0.321850949607914	0.00918214636053371	MBD3L1	85509	methyl-CpG binding domain protein 3-like 1 [Source:HGNC Symbol;Acc:HGNC:15774]
0.321796736406326	0.13964593614677	SNORA70	26778	small nucleolar RNA, H/ACA box 70 [Source:HGNC Symbol;Acc:HGNC:10231]
0.321741977383117	0.155516924743602	ZNF579	163033	zinc finger protein 579 [Source:HGNC Symbol;Acc:HGNC:26646]
0.321735423905385	0.0568977734262252		347088	
0.321688097366232	0.188033646776898	DNMT3L	29947	DNA (cytosine-5-)-methyltransferase 3-like [Source:HGNC Symbol;Acc:HGNC:2980]
0.321446863458578	0.197952874572526	TAAR8	83551	trace amine associated receptor 8 [Source:HGNC Symbol;Acc:HGNC:14964]
0.321406373327854	0.0045991663443615	CHRNB3	1142	cholinergic receptor, nicotinic, beta 3 (neuronal) [Source:HGNC Symbol;Acc:HGNC:1963]
0.321383610985483	0.168191919849433	MYO1F	4542	myosin IF [Source:HGNC Symbol;Acc:HGNC:7600]
0.321262245510244	0.0272034697321179	DLGAP3	58512	discs, large (Drosophila) homolog-associated protein 3 [Source:HGNC Symbol;Acc:HGNC:30368]
0.320848844015518	0.104414430554396	ADRB3	155	adrenoceptor beta 3 [Source:HGNC Symbol;Acc:HGNC:288]
0.320810357478866	0.0426243789736314		284498	
0.320763858891311	0.135255528750577	IL3	3562	interleukin 3 [Source:HGNC Symbol;Acc:HGNC:6011]
0.320703074786459	0.108733824336163	TUBB3	10381	tubulin, beta 3 class III [Source:HGNC Symbol;Acc:HGNC:20772]
0.320604609462822	0.18869544754391	KCNQ5	56479	potassium voltage-gated channel, KQT-like subfamily, member 5 [Source:HGNC Symbol;Acc:HGNC:6299]
0.320451034734464	0.163232134684657	SSC4D	136853	scavenger receptor cysteine rich family, 4 domains [Source:HGNC Symbol;Acc:HGNC:14461]
0.320365365715883	0.171905133957999	H1FNT	341567	H1 histone family, member N, testis-specific [Source:HGNC Symbol;Acc:HGNC:24893]
0.320258870334772	0.00963331201483493	LRRC34	151827	leucine rich repeat containing 34 [Source:HGNC Symbol;Acc:HGNC:28408]
0.320255643566159	0.190034359523632	PRPH	5630	peripherin [Source:HGNC Symbol;Acc:HGNC:9461]
0.319567443501549	0.0921320316558219	KRT18P55	284085	keratin 18 pseudogene 55 [Source:HGNC Symbol;Acc:HGNC:26874]
0.319538856664269	0.0660910531967042	ALDH3B2	222	aldehyde dehydrogenase 3 family, member B2 [Source:HGNC Symbol;Acc:HGNC:411]
0.319403045065272	0.162631408624981	PEBP4	157310	phosphatidylethanolamine-binding protein 4 [Source:HGNC Symbol;Acc:HGNC:28319]
0.319331184240128	0.0473705237827868	COL8A1	1295	collagen, type VIII, alpha 1 [Source:HGNC Symbol;Acc:HGNC:2215]
0.319064756648111	0.165015048933668	RRP12	23223	ribosomal RNA processing 12 homolog (S. cerevisiae) [Source:HGNC Symbol;Acc:HGNC:29100]
0.319062417114523	0.0381830438172844	KRTAP12-2	353323	keratin associated protein 12-2 [Source:HGNC Symbol;Acc:HGNC:20530]
0.319019821664137	0.014247605996285	BOD1L1	259282	biorientation of chromosomes in cell division 1-like 1 [Source:HGNC Symbol;Acc:HGNC:31792]
0.31874063351925	0.0441756891595758	CACNA1C	775	calcium channel, voltage-dependent, L type, alpha 1C subunit [Source:HGNC Symbol;Acc:HGNC:1390]
0.318709978186761	0.13771291083058	SYT12	91683	synaptotagmin XII [Source:HGNC Symbol;Acc:HGNC:18381]
0.318651634044963	0.0885399856791143	AK7	122481	adenylate kinase 7 [Source:HGNC Symbol;Acc:HGNC:20091]
0.318611496238024	0.19686942679302	OSMR	9180	oncostatin M receptor [Source:HGNC Symbol;Acc:HGNC:8507]
0.318468832836561	0.12013629601847	RIPPLY1	92129	ripply transcriptional repressor 1 [Source:HGNC Symbol;Acc:HGNC:25117]
0.318459207380659	0.0948536488036998	ENTHD1	150350	ENTH domain containing 1 [Source:HGNC Symbol;Acc:HGNC:26352]
0.318243214533496	0.0827818641602382	TRMT10B	158234	tRNA methyltransferase 10 homolog B (S. cerevisiae) [Source:HGNC Symbol;Acc:HGNC:26454]
0.318035981956877	0.159519463380619	DMRT1	1761	doublesex and mab-3 related transcription factor 1 [Source:HGNC Symbol;Acc:HGNC:2934]
0.31793214816783	0.127723283403561	MAPK15	225689	mitogen-activated protein kinase 15 [Source:HGNC Symbol;Acc:HGNC:24667]
0.317883580626309	0.0991043874218384	C1orf213	148898	chromosome 1 open reading frame 213 [Source:HGNC Symbol;Acc:HGNC:25122]
0.317875933399119	0.0618750206127847	RIPPLY3	53820	ripply transcriptional repressor 3 [Source:HGNC Symbol;Acc:HGNC:3047]
0.317822687228303	0.0367667349010313	C20orf173	140873	chromosome 20 open reading frame 173 [Source:HGNC Symbol;Acc:HGNC:16166]
0.317798432246643	0.0958475939233349	ZSCAN1	284312	zinc finger and SCAN domain containing 1 [Source:HGNC Symbol;Acc:HGNC:23712]
0.317776286195903	0.00143712909758026	ZAN	7455	zonadhesin (gene/pseudogene) [Source:HGNC Symbol;Acc:HGNC:12857]
0.317755939127994	0.0808949280390622	DUOXA1	90527	dual oxidase maturation factor 1 [Source:HGNC Symbol;Acc:HGNC:26507]
0.317753575040495	0.00651833960795958	UGT3A2	167127	UDP glycosyltransferase 3 family, polypeptide A2 [Source:HGNC Symbol;Acc:HGNC:27266]
0.317447026704577	0.136724116815445	POU3F1	5453	POU class 3 homeobox 1 [Source:HGNC Symbol;Acc:HGNC:9214]
0.317334295793862	0.114060928591211	METTL21C	196541	methyltransferase like 21C [Source:HGNC Symbol;Acc:HGNC:33717]
0.317102670772787	0.0248930434369756	SNORA5A	654319	small nucleolar RNA, H/ACA box 5A [Source:HGNC Symbol;Acc:HGNC:32588]
0.316993450760239	0.152604320666255	ITPA	3704	inosine triphosphatase (nucleoside triphosphate pyrophosphatase) [Source:HGNC Symbol;Acc:HGNC:6176]
0.316915933983661	0.131290822139185	MIR26A1	407015	microRNA 26a-1 [Source:HGNC Symbol;Acc:HGNC:31610]
0.316830622800925	0.0842204009914411	PLEKHH3	79990	pleckstrin homology domain containing, family H (with MyTH4 domain) member 3 [Source:HGNC Symbol;Acc:HGNC:26105]
0.316642425152755	0.11741468166163	SLC25A31	83447	solute carrier family 25 (mitochondrial carrier; adenine nucleotide translocator), member 31 [Source:HGNC Symbol;Acc:HGNC:25319]
0.316625577876352	0.0309232380694207	IAH1	285148	isoamyl acetate-hydrolyzing esterase 1 homolog (S. cerevisiae) [Source:HGNC Symbol;Acc:HGNC:27696]
0.316594682849632	0.113711009994026	FLRT1	23769	fibronectin leucine rich transmembrane protein 1 [Source:HGNC Symbol;Acc:HGNC:3760]
0.316483462617375	0.0563576115596653	MAGEB10	139422	melanoma antigen family B, 10 [Source:HGNC Symbol;Acc:HGNC:25377]
0.316467073534027	0.0985695835056436	DDX50	79009	DEAD (Asp-Glu-Ala-Asp) box polypeptide 50 [Source:HGNC Symbol;Acc:HGNC:17906]
0.316442922462419	0.154573923653621	DCK	1633	deoxycytidine kinase [Source:HGNC Symbol;Acc:HGNC:2704]
0.316259242869922	0.159357377915114	SH2D6	284948	SH2 domain containing 6 [Source:HGNC Symbol;Acc:HGNC:30439]
0.316226185053731	0.115788822262548	C5orf34	375444	chromosome 5 open reading frame 34 [Source:HGNC Symbol;Acc:HGNC:24738]
0.316041260760733	0.12099815571701	NUPR1L	389493	nuclear protein, transcriptional regulator, 1-like [Source:HGNC Symbol;Acc:HGNC:44164]
0.3159856214928	0.0837647202212755	PLA2G1B	5319	phospholipase A2, group IB (pancreas) [Source:HGNC Symbol;Acc:HGNC:9030]
0.315803488441378	0.13909253175317	NEK8	284086	NIMA-related kinase 8 [Source:HGNC Symbol;Acc:HGNC:13387]
0.315675386582146	0.0536279421997237	GJB7	375519	gap junction protein, beta 7, 25kDa [Source:HGNC Symbol;Acc:HGNC:16690]
0.315580427471615	0.0683268786516597	FRMPD1	22844	FERM and PDZ domain containing 1 [Source:HGNC Symbol;Acc:HGNC:29159]
0.315441969490934	0.142490524061051	FOXD1	2297	forkhead box D1 [Source:HGNC Symbol;Acc:HGNC:3802]
0.315346737328878	0.0452337711480677	GLT1D1	144423	glycosyltransferase 1 domain containing 1 [Source:HGNC Symbol;Acc:HGNC:26483]
0.315225047473755	0.16732362398424	ALPK2	115701	alpha-kinase 2 [Source:HGNC Symbol;Acc:HGNC:20565]
0.31497259448719	0.139369322950859	MIR138-1	406929	microRNA 138-1 [Source:HGNC Symbol;Acc:HGNC:31524]
0.314919097013581	0.188880599648464	MUCL1	118430	mucin-like 1 [Source:HGNC Symbol;Acc:HGNC:30588]
0.314704470070759	0.181015357902632	SLC25A14	9016	solute carrier family 25 (mitochondrial carrier, brain), member 14 [Source:HGNC Symbol;Acc:HGNC:10984]
0.314653471692772	0.180479049639141	AXDND1	126859	axonemal dynein light chain domain containing 1 [Source:HGNC Symbol;Acc:HGNC:26564]
0.314652924319959	0.143602917542376	AATK-AS1	388428	AATK antisense RNA 1 [Source:HGNC Symbol;Acc:HGNC:40053]
0.314531910584189	0.162314859987207	SLC17A2	10246	solute carrier family 17, member 2 [Source:HGNC Symbol;Acc:HGNC:10930]
0.314490107628938	0.0162592912284312	PINLYP	390940	phospholipase A2 inhibitor and LY6/PLAUR domain containing [Source:HGNC Symbol;Acc:HGNC:44206]
0.314179985293006	0.0247398125198873	ZFP69B	65243	ZFP69 zinc finger protein B [Source:HGNC Symbol;Acc:HGNC:28053]
0.314087839141126	0.148723342626407	FZD10	11211	frizzled class receptor 10 [Source:HGNC Symbol;Acc:HGNC:4039]
0.314063452443508	0.139223514953212	ROBO3	64221	roundabout, axon guidance receptor, homolog 3 (Drosophila) [Source:HGNC Symbol;Acc:HGNC:13433]
0.313963208149027	0.0956445831873702	HPN	3249	hepsin [Source:HGNC Symbol;Acc:HGNC:5155]
0.313941134348823	0.167102599600797	TDRD5	163589	tudor domain containing 5 [Source:HGNC Symbol;Acc:HGNC:20614]
0.31376794946597	0.0870182725037092	VASH2	79805	vasohibin 2 [Source:HGNC Symbol;Acc:HGNC:25723]
0.313631738330378	0.149671421114801	TMEM198	130612	transmembrane protein 198 [Source:HGNC Symbol;Acc:HGNC:33704]
0.313594722881057	0.1789105679158	LINGO4	339398	leucine rich repeat and Ig domain containing 4 [Source:HGNC Symbol;Acc:HGNC:31814]
0.313552152586708	0.0721317139721148	SFI1	9814	Sfi1 homolog, spindle assembly associated (yeast) [Source:HGNC Symbol;Acc:HGNC:29064]
0.313333639425001	0.0678330328481851	MIR124-2	406908	microRNA 124-2 [Source:HGNC Symbol;Acc:HGNC:31503]
0.313235154787271	0.0604435029644671	ESYT3	83850	extended synaptotagmin-like protein 3 [Source:HGNC Symbol;Acc:HGNC:24295]
0.31304539781474	0.0873428658250689	FGF17	8822	fibroblast growth factor 17 [Source:HGNC Symbol;Acc:HGNC:3673]
0.312498047248994	0.122249742277834	WNT1	7471	wingless-type MMTV integration site family, member 1 [Source:HGNC Symbol;Acc:HGNC:12774]
0.312396716068388	0.0389554242754008	ATG12	9140	autophagy related 12 [Source:HGNC Symbol;Acc:HGNC:588]
0.312204286895144	0.168940556022644		619207	scavenger receptor protein family member [Source:EntrezGene;Acc:619207]
0.312048963235893	0.0119316755413749	PBX4	80714	pre-B-cell leukemia homeobox 4 [Source:HGNC Symbol;Acc:HGNC:13403]
0.311913286304243	0.18031425803237	CACNA2D3	55799	calcium channel, voltage-dependent, alpha 2/delta subunit 3 [Source:HGNC Symbol;Acc:HGNC:15460]
0.311836236116419	0.140622393344456	KRTAP23-1	337963	keratin associated protein 23-1 [Source:HGNC Symbol;Acc:HGNC:18928]
0.311808554047872	0.124964788878761	RHBG	57127	Rh family, B glycoprotein (gene/pseudogene) [Source:HGNC Symbol;Acc:HGNC:14572]
0.311457315562221	0.0155635176115145	IRG1	730249	immunoresponsive 1 homolog (mouse) [Source:HGNC Symbol;Acc:HGNC:33904]
0.311428360020214	0.00738429959694055	KIF17	57576	kinesin family member 17 [Source:HGNC Symbol;Acc:HGNC:19167]
0.311282876715349	0.141636520814198	ISL2	64843	ISL LIM homeobox 2 [Source:HGNC Symbol;Acc:HGNC:18524]
0.311231116756256	0.135461105439097	PHEX	5251	phosphate regulating endopeptidase homolog, X-linked [Source:HGNC Symbol;Acc:HGNC:8918]
0.311180968454386	0.0525590014291548	IL31	386653	interleukin 31 [Source:HGNC Symbol;Acc:HGNC:19372]
0.311132545762952	0.0434568236412839	C15orf53	400359	chromosome 15 open reading frame 53 [Source:HGNC Symbol;Acc:HGNC:33796]
0.311081097637484	0.0768809361438475	KLHL40	131377	kelch-like family member 40 [Source:HGNC Symbol;Acc:HGNC:30372]
0.311050570790101	1.49073042723263e-06	ESX1	80712	ESX homeobox 1 [Source:HGNC Symbol;Acc:HGNC:14865]
0.31096378049918	0.0699182877497643	TREML2	79865	triggering receptor expressed on myeloid cells-like 2 [Source:HGNC Symbol;Acc:HGNC:21092]
0.310796398111003	0.178863750663327	PRIM1	5557	primase, DNA, polypeptide 1 (49kDa) [Source:HGNC Symbol;Acc:HGNC:9369]
0.310693568106128	0.121995699539309	ZDHHC22	283576	zinc finger, DHHC-type containing 22 [Source:HGNC Symbol;Acc:HGNC:20106]
0.310609815885711	0.124429598844305	BHLHE23	128408	basic helix-loop-helix family, member e23 [Source:HGNC Symbol;Acc:HGNC:16093]
0.310567361954957	0.0603705029829542	MASP2	10747	mannan-binding lectin serine peptidase 2 [Source:HGNC Symbol;Acc:HGNC:6902]
0.310411379471246	0.179614055748366	CCDC142	84865	coiled-coil domain containing 142 [Source:HGNC Symbol;Acc:HGNC:25889]
0.310389490381584	0.126662096408133		100533184	
0.310312940303493	0.192616175043788	FAM212A	389119	family with sequence similarity 212, member A [Source:HGNC Symbol;Acc:HGNC:32480]
0.310234882372464	0.14718711494556	TUSC1	286319	tumor suppressor candidate 1 [Source:HGNC Symbol;Acc:HGNC:31010]
0.31019264016095	0.00231547219634609	KCNJ8	3764	potassium inwardly-rectifying channel, subfamily J, member 8 [Source:HGNC Symbol;Acc:HGNC:6269]
0.310124125332124	0.00321016311770411	PAX2	5076	paired box 2 [Source:HGNC Symbol;Acc:HGNC:8616]
0.309958036247502	0.0232152348227733	RGL4	266747	ral guanine nucleotide dissociation stimulator-like 4 [Source:HGNC Symbol;Acc:HGNC:31911]
0.309908252019288	0.0322258926782885	WFIKKN2	124857	WAP, follistatin/kazal, immunoglobulin, kunitz and netrin domain containing 2 [Source:HGNC Symbol;Acc:HGNC:30916]
0.309834600486081	0.129960402675001	TCF23	150921	transcription factor 23 [Source:HGNC Symbol;Acc:HGNC:18602]
0.309818085456545	0.0433213341951096	C19orf73	55150	chromosome 19 open reading frame 73 [Source:HGNC Symbol;Acc:HGNC:25534]
0.309736041756385	0.130524814983385	ZMYND15	84225	zinc finger, MYND-type containing 15 [Source:HGNC Symbol;Acc:HGNC:20997]
0.309331376417037	0.0190016893276986	CIB4	130106	calcium and integrin binding family member 4 [Source:HGNC Symbol;Acc:HGNC:33703]
0.309230627721314	0.0747403177411715	ACYP1	97	acylphosphatase 1, erythrocyte (common) type [Source:HGNC Symbol;Acc:HGNC:179]
0.309198639859388	0.0577514833868708	ZFR2	23217	zinc finger RNA binding protein 2 [Source:HGNC Symbol;Acc:HGNC:29189]
0.309073898496673	0.158031309239898	TUBA3C	7278	tubulin, alpha 3c [Source:HGNC Symbol;Acc:HGNC:12408]
0.309073032310437	0.0865895429573909	SATL1	340562	spermidine/spermine N1-acetyl transferase-like 1 [Source:HGNC Symbol;Acc:HGNC:27992]
0.309052540543945	0.0946622841060187	PEX16	9409	peroxisomal biogenesis factor 16 [Source:HGNC Symbol;Acc:HGNC:8857]
0.308950911781109	0.147093802678318	EFCAB11	90141	EF-hand calcium binding domain 11 [Source:HGNC Symbol;Acc:HGNC:20357]
0.308852199434185	0.154925367048179	PKLR	5313	pyruvate kinase, liver and RBC [Source:HGNC Symbol;Acc:HGNC:9020]
0.308717531222258	0.00798084556856865	CCDC151	115948	coiled-coil domain containing 151 [Source:HGNC Symbol;Acc:HGNC:28303]
0.30862893488412	0.0407295272396879	SNORD21	6083	small nucleolar RNA, C/D box 21 [Source:HGNC Symbol;Acc:HGNC:10144]
0.308579863024505	0.0793856272566011	LINC00898	400932	long intergenic non-protein coding RNA 898 [Source:HGNC Symbol;Acc:HGNC:48581]
0.308543323876057	0.106231588451453		100294362	

0.308462488414023	0.0559417622733062	EXTL1	2134	exostosin-like glycosyltransferase 1 [Source:HGNC Symbol;Acc:HGNC:3515]
0.308398211727389	0.184702980504602	OR4C11	219429	olfactory receptor, family 4, subfamily C, member 11 [Source:HGNC Symbol;Acc:HGNC:15167]
0.308360216322207	0.0450260205624437	MYBBP1A	10514	MYB binding protein (P160) 1a [Source:HGNC Symbol;Acc:HGNC:7546]
0.308067048306683	0.16253338910476		730102	
0.308007084344822	0.123201504570862	OR2AG2	338755	olfactory receptor, family 2, subfamily AG, member 2 [Source:HGNC Symbol;Acc:HGNC:15143]
0.30800506638067	0.0696083502871701	LINC01121	400952	long intergenic non-protein coding RNA 1121 [Source:HGNC Symbol;Acc:HGNC:49266]
0.30798040735236	0.113816251954983	FGF22	27006	fibroblast growth factor 22 [Source:HGNC Symbol;Acc:HGNC:3679]
0.307974045107859	0.154755406101189	HOOK2	29911	hook microtubule-tethering protein 2 [Source:HGNC Symbol;Acc:HGNC:19885]
0.307911200181875	0.0874424816549151	RAB38	23682	RAB38, member RAS oncogene family [Source:HGNC Symbol;Acc:HGNC:9776]
0.307900101082176	0.0150869475278042	NPSR1-AS1	404744	NPSR1 antisense RNA 1 [Source:HGNC Symbol;Acc:HGNC:22128]
0.307790398858942	0.0886756372085202	TCP11	6954	t-complex 11, testis-specific [Source:HGNC Symbol;Acc:HGNC:11658]
0.307762689444306	0.197220224448345	CPN2	1370	carboxypeptidase N, polypeptide 2 [Source:HGNC Symbol;Acc:HGNC:2313]
0.307674434045221	0.00285777338800461	HNRNPD	3184	heterogeneous nuclear ribonucleoprotein D (AU-rich element RNA binding protein 1, 37kDa) [Source:HGNC Symbol;Acc:HGNC:5036]
0.307518683498406	0.17663188875606	CDT1	81620	chromatin licensing and DNA replication factor 1 [Source:HGNC Symbol;Acc:HGNC:24576]
0.307444805213214	0.163434005116761	CCDC158	339965	coiled-coil domain containing 158 [Source:HGNC Symbol;Acc:HGNC:26374]
0.307414581504569	0.136927318087872	LCE3A	353142	late cornified envelope 3A [Source:HGNC Symbol;Acc:HGNC:29461]
0.307335991073125	0.150678286381137	LEMD1	93273	LEM domain containing 1 [Source:HGNC Symbol;Acc:HGNC:18725]
0.307312361597796	0.13399281689118	FAM229B	619208	family with sequence similarity 229, member B [Source:HGNC Symbol;Acc:HGNC:33858]
0.30730933101693	0.147067116630198	MAP2K3	5606	mitogen-activated protein kinase kinase 3 [Source:HGNC Symbol;Acc:HGNC:6843]
0.307296384602915	0.159392109847328	PRR7	80758	proline rich 7 (synaptic) [Source:HGNC Symbol;Acc:HGNC:28130]
0.307164222720145	0.137231984663945	HRASLS	57110	HRAS-like suppressor [Source:HGNC Symbol;Acc:HGNC:14922]
0.307082798789533	0.165044184906879	SLC24A2	25769	solute carrier family 24 (sodium/potassium/calcium exchanger), member 2 [Source:HGNC Symbol;Acc:HGNC:10976]
0.30666322297868	0.0634692203356645	GPR83	10888	G protein-coupled receptor 83 [Source:HGNC Symbol;Acc:HGNC:4523]
0.306645886182353	0.00273460521810474		149830	
0.306360903829294	0.036871918956987	AVPR2	554	arginine vasopressin receptor 2 [Source:HGNC Symbol;Acc:HGNC:897]
0.306356944866743	0.108921211316779	RAMP3	10268	receptor (G protein-coupled) activity modifying protein 3 [Source:HGNC Symbol;Acc:HGNC:9845]
0.306341138509879	0.154556428573425	ZNF829	374899	zinc finger protein 829 [Source:HGNC Symbol;Acc:HGNC:34032]
0.306197007413638	0.188359689158955	KNTC1	9735	kinetochore associated 1 [Source:HGNC Symbol;Acc:HGNC:17255]
0.305917457536473	0.140605637939037	KIAA1045	23349	KIAA1045 [Source:HGNC Symbol;Acc:HGNC:29180]
0.305579775791212	0.120805079374146	BOC	91653	BOC cell adhesion associated, oncogene regulated [Source:HGNC Symbol;Acc:HGNC:17173]
0.305228818792845	0.146797818271106	DGKK	139189	diacylglycerol kinase, kappa [Source:HGNC Symbol;Acc:HGNC:32395]
0.305224797369917	0.089676164447708	CASC2	255082	cancer susceptibility candidate 2 (non-protein coding) [Source:HGNC Symbol;Acc:HGNC:22933]
0.305101730702946	0.0916206978635556	AQP6	363	aquaporin 6, kidney specific [Source:HGNC Symbol;Acc:HGNC:639]
0.304744002125416	0.180581359249742	TH	7054	tyrosine hydroxylase [Source:HGNC Symbol;Acc:HGNC:11782]
0.304553856730554	0.1718511980821	TUBA3FP	113691	tubulin, alpha 3f, pseudogene [Source:HGNC Symbol;Acc:HGNC:24067]
0.304481062354907	0.0827000733850399	SPATA31E1	286234	SPATA31 subfamily E, member 1 [Source:HGNC Symbol;Acc:HGNC:26672]
0.304378992945884	0.0588679372068561	TSSK4	283629	testis-specific serine kinase 4 [Source:HGNC Symbol;Acc:HGNC:19825]
0.304278586026983	0.168397615906439	IL19	29949	interleukin 19 [Source:HGNC Symbol;Acc:HGNC:5990]
0.304210891882645	0.06258694734393	GPX6	257202	glutathione peroxidase 6 (olfactory) [Source:HGNC Symbol;Acc:HGNC:4558]
0.30409716277456	0.0551830147230976	LRRC71	149499	leucine rich repeat containing 71 [Source:HGNC Symbol;Acc:HGNC:26556]
0.304092087328746	0.148433889942145	HSPA14	51182	heat shock 70kDa protein 14 [Source:HGNC Symbol;Acc:HGNC:29526]
0.303931597949546	0.136468351424664	MTUS2	23281	microtubule associated tumor suppressor candidate 2 [Source:HGNC Symbol;Acc:HGNC:20595]
0.303723338427831	0.105677543912806	BNC1	646	basonuclin 1 [Source:HGNC Symbol;Acc:HGNC:1081]
0.303672918811629	0.115594145696697	TMEM158	25907	transmembrane protein 158 (gene/pseudogene) [Source:HGNC Symbol;Acc:HGNC:30293]
0.303504652671741	0.05409593818992	BARX1	56033	BARX homeobox 1 [Source:HGNC Symbol;Acc:HGNC:955]
0.303440657446153	0.137987272634853	FAM27L	284123	family with sequence similarity 27-like [Source:HGNC Symbol;Acc:HGNC:32410]
0.303284613773246	0.123189845086633	CNTD2	79935	cyclin N-terminal domain containing 2 [Source:HGNC Symbol;Acc:HGNC:25805]
0.303017154463401	0.157107124939297	COX6B2	125965	cytochrome c oxidase subunit VIb polypeptide 2 (testis) [Source:HGNC Symbol;Acc:HGNC:24380]
0.302963159539091	0.132892483530298	C1RL-AS1	283314	C1RL antisense RNA 1 [Source:HGNC Symbol;Acc:HGNC:27461]
0.302917799050487	0.0425056551252809	RNU105B	26767	RNA, U105B small nucleolar [Source:HGNC Symbol;Acc:HGNC:10103]
0.302819459312562	0.19374516614547	GJB4	127534	gap junction protein, beta 4, 30.3kDa [Source:HGNC Symbol;Acc:HGNC:4286]
0.302723379185555	0.107264558049272	PTPMT1	114971	protein tyrosine phosphatase, mitochondrial 1 [Source:HGNC Symbol;Acc:HGNC:26965]
0.302668040934647	0.0310050415198714	MED31	51003	mediator complex subunit 31 [Source:HGNC Symbol;Acc:HGNC:24260]
0.302590638400691	0.0935001770698692	C17orf74	201243	chromosome 17 open reading frame 74 [Source:HGNC Symbol;Acc:HGNC:27315]
0.302444664007878	0.0367266328525277	MAGEL2	54551	MAGE-like 2 [Source:HGNC Symbol;Acc:HGNC:6814]
0.302290257712818	0.0404340154225264	TBATA	219793	thymus, brain and testes associated [Source:HGNC Symbol;Acc:HGNC:23511]
0.302252406298679	0.0834874777896209	CLPB	81570	ClpB caseinolytic peptidase B homolog (E. coli) [Source:HGNC Symbol;Acc:HGNC:30664]
0.301929279564846	0.124431647710847	SELE	6401	selectin E [Source:HGNC Symbol;Acc:HGNC:10718]
0.301504107856821	0.163673585595968	GBP6	163351	guanylate binding protein family, member 6 [Source:HGNC Symbol;Acc:HGNC:25395]
0.301052343652617	0.166859545053575	ART3	419	ADP-ribosyltransferase 3 [Source:HGNC Symbol;Acc:HGNC:725]
0.301050083677299	0.053061270712259	PROX2	283571	prospero homeobox 2 [Source:HGNC Symbol;Acc:HGNC:26715]
0.30101193933188	0.145810202154124	DNAH9	1770	dynein, axonemal, heavy chain 9 [Source:HGNC Symbol;Acc:HGNC:2953]
0.300721509528787	0.11140905849733	C7orf50	84310	chromosome 7 open reading frame 50 [Source:HGNC Symbol;Acc:HGNC:22421]
0.300623473954892	0.138472373990664	GAPDHS	26330	glyceraldehyde-3-phosphate dehydrogenase, spermatogenic [Source:HGNC Symbol;Acc:HGNC:24864]
0.300577612306133	0.123451772442698	FGF3	2248	fibroblast growth factor 3 [Source:HGNC Symbol;Acc:HGNC:3681]
0.300575745343681	0.198259927963914	C1orf177	163747	chromosome 1 open reading frame 177 [Source:HGNC Symbol;Acc:HGNC:26854]
0.300545130429242	0.117600346555726		100288797	CDNA FLJ26142 fis, clone TST04526; Transmembrane protein 239 [Source:UniProtKB/TrEMBL;Acc:Q6ZPB1]
0.300464420371371	0.127185245791896	GRIN2A	2903	glutamate receptor, ionotropic, N-methyl D-aspartate 2A [Source:HGNC Symbol;Acc:HGNC:4585]
0.30042126423755	0.052683282497885	RNU6-72P	100873775	RNA, U6 small nuclear 72, pseudogene [Source:HGNC Symbol;Acc:HGNC:42562]
0.300268531328151	0.10954316447904	MIR188	406964	microRNA 188 [Source:HGNC Symbol;Acc:HGNC:31559]
0.300260310924482	0.170713973483667	RNF126P1	376412	ring finger protein 126 pseudogene 1 [Source:HGNC Symbol;Acc:HGNC:30340]
0.300067372497596	0.155249016088726	TPRX1	284355	tetra-peptide repeat homeobox 1 [Source:HGNC Symbol;Acc:HGNC:32174]
0.300029357403062	0.16946801124402	TEX38	374973	testis expressed 38 [Source:HGNC Symbol;Acc:HGNC:29589]
0.299890711324222	0.0532474315344068	MIR9-3	407051	microRNA 9-3 [Source:HGNC Symbol;Acc:HGNC:31646]
0.299575350441867	0.158810066169355	ZFP41	286128	ZFP41 zinc finger protein [Source:HGNC Symbol;Acc:HGNC:26786]
0.299529459933667	0.186195841690228	SLC25A47	283600	solute carrier family 25, member 47 [Source:HGNC Symbol;Acc:HGNC:20115]
0.29902247446694	0.141232098932959	SMCO1	255798	single-pass membrane protein with coiled-coil domains 1 [Source:HGNC Symbol;Acc:HGNC:27407]
0.298992895070213	0.13092706185952	UACA	55075	uveal autoantigen with coiled-coil domains and ankyrin repeats [Source:HGNC Symbol;Acc:HGNC:15947]
0.298854850950415	0.0316774746002552	GPR78	27201	G protein-coupled receptor 78 [Source:HGNC Symbol;Acc:HGNC:4528]
0.298412006449633	0.0140093905201633	MITD1	129531	MIT, microtubule interacting and transport, domain containing 1 [Source:HGNC Symbol;Acc:HGNC:25207]
0.298388508417602	0.0555268714612154	MMD2	221938	monocyte to macrophage differentiation-associated 2 [Source:HGNC Symbol;Acc:HGNC:30133]
0.298352279702141	0.105571715366985	PNPLA5	150379	patatin-like phospholipase domain containing 5 [Source:HGNC Symbol;Acc:HGNC:24888]
0.298272378622676	0.171690076202956	CBR3	874	carbonyl reductase 3 [Source:HGNC Symbol;Acc:HGNC:1549]
0.298229989546907	0.116295103307419	PPM1E	22843	protein phosphatase, Mg2+/Mn2+ dependent, 1E [Source:HGNC Symbol;Acc:HGNC:19322]
0.298184172469784	0.121247800015913	SNORD95	619570	small nucleolar RNA, C/D box 95 [Source:HGNC Symbol;Acc:HGNC:32757]
0.298119662144004	0.181484414523536	ABCA13	154664	ATP-binding cassette, sub-family A (ABC1), member 13 [Source:HGNC Symbol;Acc:HGNC:14638]
0.297886245729518	0.0365907741524637	NAV2	89797	neuron navigator 2 [Source:HGNC Symbol;Acc:HGNC:15997]
0.297876648432382	0.0612670083293133	WT1	7490	Wilms tumor 1 [Source:HGNC Symbol;Acc:HGNC:12796]
0.297720711685614	0.133343844958561	NRXN2	9379	neurexin 2 [Source:HGNC Symbol;Acc:HGNC:8009]
0.297621356424768	0.0599072749591866	FAM73B	84895	family with sequence similarity 73, member B [Source:HGNC Symbol;Acc:HGNC:23621]
0.297311516972778	0.137804091445016	SPSB4	92369	splA/ryanodine receptor domain and SOCS box containing 4 [Source:HGNC Symbol;Acc:HGNC:30630]
0.297303686436968	0.125227593729341	CD2	914	CD2 molecule [Source:HGNC Symbol;Acc:HGNC:1639]
0.29721195889802	0.192844667206424	KAAG1	353219	kidney associated antigen 1 [Source:HGNC Symbol;Acc:HGNC:21031]
0.297132047449516	0.189175633118192	SMAD3	4088	SMAD family member 3 [Source:HGNC Symbol;Acc:HGNC:6769]
0.296863295668378	0.091729044249127	SRSF12	135295	serine/arginine-rich splicing factor 12 [Source:HGNC Symbol;Acc:HGNC:21220]
0.296804325730735	0.0744575809014368	MATN4	8785	matrilin 4 [Source:HGNC Symbol;Acc:HGNC:6910]
0.296692449793498	0.115928376396933	ATCAY	85300	ataxia, cerebellar, Cayman type [Source:HGNC Symbol;Acc:HGNC:779]
0.296689287395837	0.167567357127614	KRTAP4-3	85290	keratin associated protein 4-3 [Source:HGNC Symbol;Acc:HGNC:18908]
0.296333872753277	0.0825828111202019	TP53AIP1	63970	tumor protein p53 regulated apoptosis inducing protein 1 [Source:HGNC Symbol;Acc:HGNC:29984]
0.296222723462244	0.192271234514126	GOLT1A	127845	golgi transport 1A [Source:HGNC Symbol;Acc:HGNC:24766]
0.296192018076561	0.136902677274992	LRRIQ3	127255	leucine-rich repeats and IQ motif containing 3 [Source:HGNC Symbol;Acc:HGNC:28318]
0.29602362496704	0.0843129644490831	SCARNA23	677773	small Cajal body-specific RNA 23 [Source:HGNC Symbol;Acc:HGNC:32581]
0.295789617213652	0.17043027319365	TESC	54997	tescalcin [Source:HGNC Symbol;Acc:HGNC:26065]
0.295662949908389	0.124546722393433	MTMR9LP	339483	myotubularin related protein 9-like, pseudogene [Source:HGNC Symbol;Acc:HGNC:27920]
0.295439105619009	0.0671620921390306	PRODH2	58510	proline dehydrogenase (oxidase) 2 [Source:HGNC Symbol;Acc:HGNC:17325]
0.295387845837795	0.0917966820370619	CACNG8	59283	calcium channel, voltage-dependent, gamma subunit 8 [Source:HGNC Symbol;Acc:HGNC:13628]
0.29498522945264	0.122618266849959	FNDC8	54752	fibronectin type III domain containing 8 [Source:HGNC Symbol;Acc:HGNC:25286]
0.294935346890284	0.131120098353842	FAM69B	138311	family with sequence similarity 69, member B [Source:HGNC Symbol;Acc:HGNC:28290]
0.294864790175732	0.0851778846486789	PFKP	5214	phosphofructokinase, platelet [Source:HGNC Symbol;Acc:HGNC:8878]
0.294836142870733	0.147589964799944	ADCK5	203054	aarF domain containing kinase 5 [Source:HGNC Symbol;Acc:HGNC:21738]
0.294742145946204	0.0488900822985209	SPATA21	374955	spermatogenesis associated 21 [Source:HGNC Symbol;Acc:HGNC:28026]
0.294717822718135	0.167612369823659	MIR93	407050	microRNA 93 [Source:HGNC Symbol;Acc:HGNC:31645]
0.294570715433434	0.100340119616465	LEPRE1	64175	leucine proline-enriched proteoglycan (leprecan) 1 [Source:HGNC Symbol;Acc:HGNC:19316]
0.294558336428117	0.152820052028388	INPP5E	56623	inositol polyphosphate-5-phosphatase, 72 kDa [Source:HGNC Symbol;Acc:HGNC:21474]
0.294480197377611	0.137433193961361	FLG	2312	filaggrin [Source:HGNC Symbol;Acc:HGNC:3748]
0.294410475483694	0.117468007985483	RGS11	8786	regulator of G-protein signaling 11 [Source:HGNC Symbol;Acc:HGNC:9993]
0.294344073190245	0.137888350149921	C6orf15	29113	chromosome 6 open reading frame 15 [Source:HGNC Symbol;Acc:HGNC:13927]
0.294314504069617	0.121359183830545	IRF2BP1	26145	interferon regulatory factor 2 binding protein 1 [Source:HGNC Symbol;Acc:HGNC:21728]
0.294282719829668	0.189460886480678	STK17A	9263	serine/threonine kinase 17a [Source:HGNC Symbol;Acc:HGNC:11395]
0.294247630936245	0.175758812774872	GPR152	390212	G protein-coupled receptor 152 [Source:HGNC Symbol;Acc:HGNC:23622]
0.294198168522868	0.0562528737972232	PIM3	415116	Pim-3 proto-oncogene, serine/threonine kinase [Source:HGNC Symbol;Acc:HGNC:19310]
0.293871163552065	0.115360167991715		441394	
0.293794565782709	0.0375288087801618	ZACN	353174	zinc activated ligand-gated ion channel [Source:HGNC Symbol;Acc:HGNC:29504]
0.293560742235727	0.141633438282783	OGDHL	55753	oxoglutarate dehydrogenase-like [Source:HGNC Symbol;Acc:HGNC:25590]
0.293496159118131	0.0512691960193991		114794	
0.293193760222142	0.139382474906271	CHTF18	63922	CTF18, chromosome transmission fidelity factor 18 homolog (S. cerevisiae) [Source:HGNC Symbol;Acc:HGNC:18435]
0.293163385709639	0.122117858418891	C1QTNF1	114897	C1q and tumor necrosis factor related protein 1 [Source:HGNC Symbol;Acc:HGNC:14324]
0.293129269179318	0.131077602333112	FBXW9	84261	F-box and WD repeat domain containing 9 [Source:HGNC Symbol;Acc:HGNC:28136]
0.293004285917383	0.051989838078857	NR0B1	190	nuclear receptor subfamily 0, group B, member 1 [Source:HGNC Symbol;Acc:HGNC:7960]
0.292931576593947	0.000484385279153486	MAP3K14	9020	mitogen-activated protein kinase kinase kinase 14 [Source:HGNC Symbol;Acc:HGNC:6853]
0.29289161795917	0.115367756453905	TMX3	54495	thioredoxin-related transmembrane protein 3 [Source:HGNC Symbol;Acc:HGNC:24718]
0.292811206546138	0.068478128868815	ZNF496	84838	zinc finger protein 496 [Source:HGNC Symbol;Acc:HGNC:23713]
0.292421833193543	0.10335529886256	C4BPB	725	complement component 4 binding protein, beta [Source:HGNC Symbol;Acc:HGNC:1328]
0.292294795269305	0.169136353745204	HOXB-AS3	404266	HOXB cluster antisense RNA 3 [Source:HGNC Symbol;Acc:HGNC:40283]
0.292190566836855	0.123942906292193	CACNA1B	774	calcium channel, voltage-dependent, N type, alpha 1B subunit [Source:HGNC Symbol;Acc:HGNC:1389]
0.291883034758495	0.134983752716947	CES5A	221223	carboxylesterase 5A [Source:HGNC Symbol;Acc:HGNC:26459]
0.291843715190284	0.0245476891163609	CACYBP	27101	calcyclin binding protein [Source:HGNC Symbol;Acc:HGNC:30423]
0.291676636336046	0.0745346164642154	B3GAT1	27087	beta-1,3-glucuronyltransferase 1 [Source:HGNC Symbol;Acc:HGNC:921]
0.291594311181761	0.174643988995577	OFCC1	266553	orofacial cleft 1 candidate 1 [Source:HGNC Symbol;Acc:HGNC:21017]
0.291450168082827	0.171865332970628	PPP1R2P9	80316	protein phosphatase 1, regulatory (inhibitor) subunit 2 pseudogene 9 [Source:HGNC Symbol;Acc:HGNC:16324]
0.291368264548901	0.0640480449492237	NR2E3	10002	nuclear receptor subfamily 2, group E, member 3 [Source:HGNC Symbol;Acc:HGNC:7974]

0.291089968070937	0.0330815098651125	MROH2A	339766	maestro heat-like repeat family member 2A [Source:HGNC Symbol;Acc:HGNC:27936]
0.29064244330253	0.0841841899449042	KCNF1	3754	potassium voltage-gated channel, subfamily F, member 1 [Source:HGNC Symbol;Acc:HGNC:6246]
0.290616484151347	0.0831873796008573	EFS	10278	embryonal Fyn-associated substrate [Source:HGNC Symbol;Acc:HGNC:16898]
0.290559959860754	0.109671664673049	CYP27B1	1594	cytochrome P450, family 27, subfamily B, polypeptide 1 [Source:HGNC Symbol;Acc:HGNC:2606]
0.2905593536026	0.175555390954219	KRTAP11-1	337880	keratin associated protein 11-1 [Source:HGNC Symbol;Acc:HGNC:18922]
0.290499712893166	0.102848902097379	GTSF1L	149699	gametocyte specific factor 1-like [Source:HGNC Symbol;Acc:HGNC:16198]
0.290387449735534	0.178911525479546	ALDH3A1	218	aldehyde dehydrogenase 3 family, member A1 [Source:HGNC Symbol;Acc:HGNC:405]
0.290181324895198	0.106450501075408	MYBPC2	4606	myosin binding protein C, fast type [Source:HGNC Symbol;Acc:HGNC:7550]
0.289961244600149	0.0149979890806654	CRCT1	54544	cysteine-rich C-terminal 1 [Source:HGNC Symbol;Acc:HGNC:29875]
0.289889405738733	0.0724215610146065	KRT80	144501	keratin 80 [Source:HGNC Symbol;Acc:HGNC:27056]
0.289883510395964	0.132298739671903	SERPINB12	89777	serpin peptidase inhibitor, clade B (ovalbumin), member 12 [Source:HGNC Symbol;Acc:HGNC:14220]
0.2896951213893	0.0502174367739917	TULP1	7287	tubby like protein 1 [Source:HGNC Symbol;Acc:HGNC:12423]
0.289463782019632	0.073831413686923	SOX1	6656	SRY (sex determining region Y)-box 1 [Source:HGNC Symbol;Acc:HGNC:11189]
0.289403733981837	0.162718598088718	AK6	6880	adenylate kinase 6 [Source:HGNC Symbol;Acc:HGNC:49151]
0.289396741881308	0.0266131509338668	WNT3A	89780	wingless-type MMTV integration site family, member 3A [Source:HGNC Symbol;Acc:HGNC:15983]
0.289202142903049	0.181830035881826	HBM	3042	hemoglobin, mu [Source:HGNC Symbol;Acc:HGNC:4826]
0.288989576038798	0.0130642943558688	SLC36A3	285641	solute carrier family 36, member 3 [Source:HGNC Symbol;Acc:HGNC:19659]
0.288928955662274	0.114006355122491	GRM4	2914	glutamate receptor, metabotropic 4 [Source:HGNC Symbol;Acc:HGNC:4596]
0.288888692430972	0.187141942152059	RNU6-53P	100873762	RNA, U6 small nuclear 53, pseudogene [Source:HGNC Symbol;Acc:HGNC:42543]
0.288815541484432	0.0011964043549919	AP4B1	10717	adaptor-related protein complex 4, beta 1 subunit [Source:HGNC Symbol;Acc:HGNC:572]
0.288621985280303	0.0933490144764246	MAD2L2	10459	MAD2 mitotic arrest deficient-like 2 (yeast) [Source:HGNC Symbol;Acc:HGNC:6764]
0.288469066846409	0.169879036386661	DSN1	79980	DSN1, MIS12 kinetochore complex component [Source:HGNC Symbol;Acc:HGNC:16165]
0.288402219511274	0.130965660984432	MAP3K10	4294	mitogen-activated protein kinase kinase kinase 10 [Source:HGNC Symbol;Acc:HGNC:6849]
0.288026084812528	0.0771574952060585	CPNE6	9362	copine VI (neuronal) [Source:HGNC Symbol;Acc:HGNC:2319]
0.288003339764332	0.149349994649116	PNPLA1	285848	patatin-like phospholipase domain containing 1 [Source:HGNC Symbol;Acc:HGNC:21246]
0.287701466852047	0.099451776890176	TMEM204	79652	transmembrane protein 204 [Source:HGNC Symbol;Acc:HGNC:14158]
0.287668513719536	0.0198426603457101	WFDC8	90199	WAP four-disulfide core domain 8 [Source:HGNC Symbol;Acc:HGNC:16163]
0.287539956601537	0.199120878001212	MAP7D2	256714	MAP7 domain containing 2 [Source:HGNC Symbol;Acc:HGNC:25899]
0.287413280801275	0.0973342961412445	CCDC57	284001	coiled-coil domain containing 57 [Source:HGNC Symbol;Acc:HGNC:27564]
0.287315698771467	0.0240313802394756	STKLD1	169436	serine/threonine kinase-like domain containing 1 [Source:HGNC Symbol;Acc:HGNC:28669]
0.287188137310516	0.159803219332406	C2orf82	389084	chromosome 2 open reading frame 82 [Source:HGNC Symbol;Acc:HGNC:33763]
0.287181473526176	0.135901675915041	LINC00189	193629	long intergenic non-protein coding RNA 189 [Source:HGNC Symbol;Acc:HGNC:18461]
0.287087365300579	0.0404722889987987	GRIN1	2902	glutamate receptor, ionotropic, N-methyl D-aspartate 1 [Source:HGNC Symbol;Acc:HGNC:4584]
0.286981813485385	0.112173150377076	WDR53	348793	WD repeat domain 53 [Source:HGNC Symbol;Acc:HGNC:28786]
0.28690206936538	0.131331042495274	TMEM190	147744	transmembrane protein 190 [Source:HGNC Symbol;Acc:HGNC:29632]
0.286787098764323	0.192939238371552	UBE2W	55284	ubiquitin-conjugating enzyme E2W (putative) [Source:HGNC Symbol;Acc:HGNC:25616]
0.286767020939472	0.199121164606006	KRT37	8688	keratin 37 [Source:HGNC Symbol;Acc:HGNC:6455]
0.286729545365983	0.15887591431763	PCDHB5	26167	protocadherin beta 5 [Source:HGNC Symbol;Acc:HGNC:8690]
0.28670048413762	0.139119184883665		440508	
0.286483687331771	0.108508886169528	OPCML	4978	opioid binding protein/cell adhesion molecule-like [Source:HGNC Symbol;Acc:HGNC:8143]
0.286326383276719	0.116872630489942	MYL10	93408	myosin, light chain 10, regulatory [Source:HGNC Symbol;Acc:HGNC:29825]
0.286045255569592	0.154199931642596	CDH22	64405	cadherin 22, type 2 [Source:HGNC Symbol;Acc:HGNC:13251]
0.285707647123931	0.194080516736682	ERAS	3266	ES cell expressed Ras [Source:HGNC Symbol;Acc:HGNC:5174]
0.285643426868778	0.0135014152965868	ATP2B2	491	ATPase, Ca++ transporting, plasma membrane 2 [Source:HGNC Symbol;Acc:HGNC:815]
0.285625443312336	0.180795832781215	ATP6AP1L	92270	ATPase, H+ transporting, lysosomal accessory protein 1-like [Source:HGNC Symbol;Acc:HGNC:28091]
0.285615352456266	0.127664571745511	HTR2A	3356	5-hydroxytryptamine (serotonin) receptor 2A, G protein-coupled [Source:HGNC Symbol;Acc:HGNC:5293]
0.285505581581594	0.133309413398558	TMPRSS9	360200	transmembrane protease, serine 9 [Source:HGNC Symbol;Acc:HGNC:30079]
0.285368217380258	0.102966644151367	WNT5B	81029	wingless-type MMTV integration site family, member 5B [Source:HGNC Symbol;Acc:HGNC:16265]
0.285254992162764	0.0324994973298034	TBP	6908	TATA box binding protein [Source:HGNC Symbol;Acc:HGNC:11588]
0.285212316837037	0.0857081558532126		100131496	
0.28512014988254	0.0948931048864038	PRND	23627	prion protein 2 (dublet) [Source:HGNC Symbol;Acc:HGNC:15748]
0.284867346878569	0.165747098139268	LINC00200	399706	long intergenic non-protein coding RNA 200 [Source:HGNC Symbol;Acc:HGNC:30974]
0.284706910744752	0.152539519910306	GPR85	54329	G protein-coupled receptor 85 [Source:HGNC Symbol;Acc:HGNC:4536]
0.284516799106507	0.196709087317199	GOLGA2P9	440518	golgin A2 pseudogene 9 [Source:HGNC Symbol;Acc:HGNC:49921]
0.284493841874539	0.0838796708655084	SRMS	6725	src-related kinase lacking C-terminal regulatory tyrosine and N-terminal myristylation sites [Source:HGNC Symbol;Acc:HGNC:11298]
0.284464908002492	0.10583264728693	C17orf53	78995	chromosome 17 open reading frame 53 [Source:HGNC Symbol;Acc:HGNC:28460]
0.284458220150091	0.0993504109872302	COL13A1	1305	collagen, type XIII, alpha 1 [Source:HGNC Symbol;Acc:HGNC:2190]
0.28445531846254	0.0670771934351029	DSCR4	10281	Down syndrome critical region gene 4 [Source:HGNC Symbol;Acc:HGNC:3045]
0.284239783585751	0.0490007013074571	C5orf27	202299	chromosome 5 open reading frame 27 [Source:HGNC Symbol;Acc:HGNC:24687]
0.284099152600896	0.0963894985535238	PKIA	5569	protein kinase (cAMP-dependent, catalytic) inhibitor alpha [Source:HGNC Symbol;Acc:HGNC:9017]
0.283890540174493	0.0344448229263215	ATXN7L2	127002	ataxin 7-like 2 [Source:HGNC Symbol;Acc:HGNC:28713]
0.283830860025554	0.100659522320831	LMO2	4005	LIM domain only 2 (rhombotin-like 1) [Source:HGNC Symbol;Acc:HGNC:6642]
0.283651485351302	0.0873863986444877	GPR149	344758	G protein-coupled receptor 149 [Source:HGNC Symbol;Acc:HGNC:23627]
0.283635444153115	0.0692561175471887	GPHA2	170589	glycoprotein hormone alpha 2 [Source:HGNC Symbol;Acc:HGNC:18054]
0.283457730318171	0.158597266368033	GALP	85569	galanin-like peptide [Source:HGNC Symbol;Acc:HGNC:24840]
0.283306701365852	0.0884842404124397	COL8A2	1296	collagen, type VIII, alpha 2 [Source:HGNC Symbol;Acc:HGNC:2216]
0.283190701893316	0.195789473038946	NLRP3	114548	NLR family, pyrin domain containing 3 [Source:HGNC Symbol;Acc:HGNC:16400]
0.283153985502606	0.15925462977372	ACSM5	54988	acyl-CoA synthetase medium-chain family member 5 [Source:HGNC Symbol;Acc:HGNC:26060]
0.283018814497416	0.121403786817365	GADD45G	10912	growth arrest and DNA-damage-inducible, gamma [Source:HGNC Symbol;Acc:HGNC:4097]
0.282747340900059	0.129477336500085	C7orf72	100130988	chromosome 7 open reading frame 72 [Source:HGNC Symbol;Acc:HGNC:22564]
0.282641426581167	0.13110666149551	UNC5A	90249	unc-5 homolog A (C. elegans) [Source:HGNC Symbol;Acc:HGNC:12567]
0.282635971199437	0.169512023295699	KLF17	128209	Kruppel-like factor 17 [Source:HGNC Symbol;Acc:HGNC:18830]
0.282539034193988	0.0293544631214928	CLEC3A	10143	C-type lectin domain family 3, member A [Source:HGNC Symbol;Acc:HGNC:2052]
0.281930361849368	0.112123736357225	DYDC2	84332	DPY30 domain containing 2 [Source:HGNC Symbol;Acc:HGNC:23468]
0.28192194827964	0.126918156344331	BHLHE22	27319	basic helix-loop-helix family, member e22 [Source:HGNC Symbol;Acc:HGNC:11963]
0.28176883099403	0.0352320563077205	RAPH1	65059	Ras association (RalGDS/AF-6) and pleckstrin homology domains 1 [Source:HGNC Symbol;Acc:HGNC:14436]
0.281585421024794	0.0832033446484811	NLRP1	22861	NLR family, pyrin domain containing 1 [Source:HGNC Symbol;Acc:HGNC:14374]
0.28147541856485	0.0971948359838731	HSD11B1	3290	hydroxysteroid (11-beta) dehydrogenase 1 [Source:HGNC Symbol;Acc:HGNC:5208]
0.28132785356126	0.147004178065265	GKN2	200504	gastrokine 2 [Source:HGNC Symbol;Acc:HGNC:24588]
0.281272767998932	0.104352541070972	MCHR1	2847	melanin-concentrating hormone receptor 1 [Source:HGNC Symbol;Acc:HGNC:4479]
0.281268883329165	0.164380770951774	COPRS	55352	coordinator of PRMT5, differentiation stimulator [Source:HGNC Symbol;Acc:HGNC:28848]
0.280990286620554	0.103582012581447	RDH16	8608	retinol dehydrogenase 16 (all-trans) [Source:HGNC Symbol;Acc:HGNC:29674]
0.280791729257631	0.0984519752774858	TTC25	83538	tetratricopeptide repeat domain 25 [Source:HGNC Symbol;Acc:HGNC:25280]
0.280632196806821	0.088663656085128	PSMA8	143471	proteasome (prosome, macropain) subunit, alpha type, 8 [Source:HGNC Symbol;Acc:HGNC:22985]
0.28056218164848	0.0575791181241606	HSPB11	51668	heat shock protein family B (small), member 11 [Source:HGNC Symbol;Acc:HGNC:25019]
0.280431991415563	0.0983794647704503	SPATA42	642864	spermatogenesis associated 42 (non-protein coding) [Source:HGNC Symbol;Acc:HGNC:41198]
0.280400637332373	0.170547729356955	OR9I1	219954	olfactory receptor, family 9, subfamily I, member 1 [Source:HGNC Symbol;Acc:HGNC:14718]
0.2803560586307	0.165452260328566	MIR4453	54553	microRNA 4453 [Source:HGNC Symbol;Acc:HGNC:41892]
0.280260523939105	0.0503533113005584	OR6A2	8590	olfactory receptor, family 6, subfamily A, member 2 [Source:HGNC Symbol;Acc:HGNC:15301]
0.280106366465692	0.00540560788951748	UBE3D	90025	ubiquitin protein ligase E3D [Source:HGNC Symbol;Acc:HGNC:21381]
0.280083852820218	0.173048752132854	SLC25A42	284439	solute carrier family 25, member 42 [Source:HGNC Symbol;Acc:HGNC:28380]
0.279966923825353	0.096455463692153	ALAS2	212	aminolevulinate, delta-, synthase 2 [Source:HGNC Symbol;Acc:HGNC:397]
0.279924013300473	0.0659569787931265	TBL1Y	90665	transducin (beta)-like 1, Y-linked [Source:HGNC Symbol;Acc:HGNC:18502]
0.279574696676781	0.127083139648435	SCARNA12	677777	small Cajal body-specific RNA 12 [Source:HGNC Symbol;Acc:HGNC:32569]
0.279568184451432	0.0587148471580613		388289	
0.279518433712181	0.175893195807535	SFTPB	6439	surfactant protein B [Source:HGNC Symbol;Acc:HGNC:10801]
0.279471357258959	0.0521012586723311	SOHLH1	402381	spermatogenesis and oogenesis specific basic helix-loop-helix 1 [Source:HGNC Symbol;Acc:HGNC:27845]
0.27934852983057	0.0758686279735737	GUCY2D	3000	guanylate cyclase 2D, membrane (retina-specific) [Source:HGNC Symbol;Acc:HGNC:4689]
0.279144455843777	0.126899523161342	S100A3	6274	S100 calcium binding protein A3 [Source:HGNC Symbol;Acc:HGNC:10493]
0.27887615376523	0.0295764047927447	TTC18	118491	tetratricopeptide repeat domain 18 [Source:HGNC Symbol;Acc:HGNC:30726]
0.278760458165384	0.148680636408211	ITGB1BP2	26548	integrin beta 1 binding protein (melusin) 2 [Source:HGNC Symbol;Acc:HGNC:6154]
0.278711632109056	0.188699562001637	EVA1B	55194	eva-1 homolog B (C. elegans) [Source:HGNC Symbol;Acc:HGNC:25558]
0.278677839779052	0.190924235860519	ADM2	79924	adrenomedullin 2 [Source:HGNC Symbol;Acc:HGNC:28898]
0.278628887615223	0.11952059900187		442229	
0.278363471584058	0.02634767890619	ABCG5	64240	ATP-binding cassette, sub-family G (WHITE), member 5 [Source:HGNC Symbol;Acc:HGNC:13886]
0.278155910529811	0.067620771656566	PAX1	5075	paired box 1 [Source:HGNC Symbol;Acc:HGNC:8615]
0.278147852230813	0.180476302696511	HLA-L	3139	major histocompatibility complex, class I, L (pseudogene) [Source:HGNC Symbol;Acc:HGNC:4970]
0.278088835881617	0.0406377368545124	SLC26A5	375611	solute carrier family 26 (anion exchanger), member 5 [Source:HGNC Symbol;Acc:HGNC:9359]
0.277659020256447	0.0675443378827148	PF4V1	5197	platelet factor 4 variant 1 [Source:HGNC Symbol;Acc:HGNC:8862]
0.277652878518965	0.0533763954453434	DENND4B	9909	DENN/MADD domain containing 4B [Source:HGNC Symbol;Acc:HGNC:29044]
0.277571830355507	0.0135377670296258	SGCG	6445	sarcoglycan, gamma (35kDa dystrophin-associated glycoprotein) [Source:HGNC Symbol;Acc:HGNC:10809]
0.27731361642655	0.0595404704179091	RUNX1	861	runt-related transcription factor 1 [Source:HGNC Symbol;Acc:HGNC:10471]
0.277239818163633	0.137983268766347	TSGA10IP	254187	testis specific, 10 interacting protein [Source:HGNC Symbol;Acc:HGNC:26555]
0.277156139453526	0.108711757917336	PIGM	93183	phosphatidylinositol glycan anchor biosynthesis, class M [Source:HGNC Symbol;Acc:HGNC:18858]
0.277147759749987	0.178460989904192	MIR140	406932	microRNA 140 [Source:HGNC Symbol;Acc:HGNC:31527]
0.276949729111666	0.163359493327217		729862	lymphocyte-specific protein 1 pseudogene 3 [Source:EntrezGene;Acc:729862]
0.276944222761104	0.181105633156163	URAD	646625	ureidoimidazoline (2-oxo-4-hydroxy-4-carboxy-5-) decarboxylase [Source:HGNC Symbol;Acc:HGNC:17785]
0.276730247655022	0.0955110348419442	LINC00479	150135	long intergenic non-protein coding RNA 479 [Source:HGNC Symbol;Acc:HGNC:19727]
0.276636028065959	0.0911032401672422	SLC6A18	348932	solute carrier family 6 (neutral amino acid transporter), member 18 [Source:HGNC Symbol;Acc:HGNC:26441]
0.276539854621725	0.14392928507677	ALDH4A1	8659	aldehyde dehydrogenase 4 family, member A1 [Source:HGNC Symbol;Acc:HGNC:406]
0.276458322896435	0.0950857656478486	OPN1SW	611	opsin 1 (cone pigments), short-wave-sensitive [Source:HGNC Symbol;Acc:HGNC:1012]
0.276247882777392	0.035282683227276	PML	5371	promyelocytic leukemia [Source:HGNC Symbol;Acc:HGNC:9113]
0.276094352716651	0.0416854050596749	KCNJ15	3772	potassium inwardly-rectifying channel, subfamily J, member 15 [Source:HGNC Symbol;Acc:HGNC:6261]
0.275997855839529	0.0353548315827147	TMEM27	57393	transmembrane protein 27 [Source:HGNC Symbol;Acc:HGNC:29437]
0.275960289370641	0.0904003822024472	BAIAP2L2	80115	BAI1-associated protein 2-like 2 [Source:HGNC Symbol;Acc:HGNC:26203]
0.275884390440765	0.0195974527473838	ACTA1	58	actin, alpha 1, skeletal muscle [Source:HGNC Symbol;Acc:HGNC:129]
0.275476855504507	0.164018054261743	MDFI	4188	MyoD family inhibitor [Source:HGNC Symbol;Acc:HGNC:6967]
0.275375399845893	0.0512138067674648	OPTC	26254	opticin [Source:HGNC Symbol;Acc:HGNC:8158]
0.275342909958776	0.0385069042559963	KCNK7	10089	potassium channel, subfamily K, member 7 [Source:HGNC Symbol;Acc:HGNC:6282]
0.275113780982485	0.120320612247808	GPR31	2853	G protein-coupled receptor 31 [Source:HGNC Symbol;Acc:HGNC:4486]
0.274961902711262	0.0356113347342639	RBFA	79863	ribosome binding factor A (putative) [Source:HGNC Symbol;Acc:HGNC:26120]
0.274851441971129	0.156228040358258	ICAM4	3386	intercellular adhesion molecule 4 (Landsteiner-Wiener blood group) [Source:HGNC Symbol;Acc:HGNC:5347]
0.274672657378864	0.0246573662578687	TRABD	80305	TraB domain containing [Source:HGNC Symbol;Acc:HGNC:28805]
0.274569011048036	0.125789071176777	MIR129-2	406918	microRNA 129-2 [Source:HGNC Symbol;Acc:HGNC:31513]
0.274314539328011	0.14092083151414	ANKRD24	170961	ankyrin repeat domain 24 [Source:HGNC Symbol;Acc:HGNC:29424]
0.274185135476832	0.0326677587124094	VSTM2B	342865	V-set and transmembrane domain containing 2B [Source:HGNC Symbol;Acc:HGNC:33595]
0.274152866187349	0.180742925041752	EPHB6	2051	EPH receptor B6 [Source:HGNC Symbol;Acc:HGNC:3396]
0.274129428394727	0.08191626941478	PGF	5228	placental growth factor [Source:HGNC Symbol;Acc:HGNC:8893]
0.273989026084798	0.19713792806807	CD6	923	CD6 molecule [Source:HGNC Symbol;Acc:HGNC:1691]
0.273952957994767	0.122904670112859	SLC13A4	26266	solute carrier family 13 (sodium/sulfate symporter), member 4 [Source:HGNC Symbol;Acc:HGNC:15827]
0.273771595959886	0.105246348024048	LRRC16B	90668	leucine rich repeat containing 16B [Source:HGNC Symbol;Acc:HGNC:20272]

0.273736413631373	0.189957893873812	POLA1	5422	polymerase (DNA directed), alpha 1, catalytic subunit [Source:HGNC Symbol;Acc:HGNC:9173]	
0.273647963057245	0.189228413317885	MIR28	407020	microRNA 28 [Source:HGNC Symbol;Acc:HGNC:31615]	
0.273404769917697	0.0710652640902562	TAS1R3	83756	taste receptor, type 1, member 3 [Source:HGNC Symbol;Acc:HGNC:15661]	
0.273380288365736	0.156065933976086	LDHC	3948	lactate dehydrogenase C [Source:HGNC Symbol;Acc:HGNC:6544]	
0.273265117618719	0.102003712406917	HHLA1	10086	HERV-H LTR-associating 1 [Source:HGNC Symbol;Acc:HGNC:4904]	
0.273188713022414	0.179197120652671	SNORD105	692229	small nucleolar RNA, C/D box 105 [Source:HGNC Symbol;Acc:HGNC:32769]	
0.273022237591368	0.179653718956597	ABCB8	11194	ATP-binding cassette, sub-family B (MDR/TAP), member 8 [Source:HGNC Symbol;Acc:HGNC:49]	
0.272883390269103	0.0909204491525154	COL18A1-AS	378832	COL18A1 antisense RNA 1 [Source:HGNC Symbol;Acc:HGNC:23132]	
0.272875792277351	0.0068748976043482		123346		
0.272836516337092	0.157796888903421	PPP1R35	221908	protein phosphatase 1, regulatory subunit 35 [Source:HGNC Symbol;Acc:HGNC:28320]	
0.272785718941655	0.148617626835643	PLIN1	5346	perilipin 1 [Source:HGNC Symbol;Acc:HGNC:9076]	
0.272780154026201	0.198529516778137	NEURL1	9148	neuralized E3 ubiquitin protein ligase 1 [Source:HGNC Symbol;Acc:HGNC:7761]	
0.272761101885679	0.074138945035246	FAT3	120114	FAT atypical cadherin 3 [Source:HGNC Symbol;Acc:HGNC:23112]	
0.272623937482587	0.109220655955618	NYAP1	222950	neuronal tyrosine-phosphorylated phosphoinositide-3-kinase adaptor 1 [Source:HGNC Symbol;Acc:HGNC:22009]	
0.272575353663557	0.0596144442581765	MAPK7	5598	mitogen-activated protein kinase 7 [Source:HGNC Symbol;Acc:HGNC:6880]	
0.272547258328365	0.117018779958365	C1orf105	92346	chromosome 1 open reading frame 105 [Source:HGNC Symbol;Acc:HGNC:29591]	
0.272361558496546	0.0280230236330958	SPTBN5	51332	spectrin, beta, non-erythrocytic 5 [Source:HGNC Symbol;Acc:HGNC:15680]	
0.272057637810745	0.170254285231111	OR2A5	393046	olfactory receptor, family 2, subfamily A, member 5 [Source:HGNC Symbol;Acc:HGNC:8232]	
0.2718649169324	0.148633427306615	SNORD45C	692085	small nucleolar RNA, C/D box 45C [Source:HGNC Symbol;Acc:HGNC:32720]	
0.271857997178577	0.179045814428728	GOLGA6L2	283685	golgin A6 family-like 2 [Source:HGNC Symbol;Acc:HGNC:26695]	
0.271693574381579	0.0453609155222927	BAI1	575	brain-specific angiogenesis inhibitor 1 [Source:HGNC Symbol;Acc:HGNC:943]	
0.271315941269779	0.0781206354995213	FEV	54738	FEV (ETS oncogene family) [Source:HGNC Symbol;Acc:HGNC:18562]	
0.271238878132423	0.00531474672033506	RNASE12	493901	ribonuclease, RNase A family, 12 (non-active) [Source:HGNC Symbol;Acc:HGNC:24211]	
0.271197945942008	0.167833661308779	NOP14	8602	NOP14 nucleolar protein [Source:HGNC Symbol;Acc:HGNC:16821]	
0.271061996343092	0.120491131108281	KCNG3	170850	potassium voltage-gated channel, subfamily G, member 3 [Source:HGNC Symbol;Acc:HGNC:18306]	
0.27099535227247	0.120967443634627	GRIN3B	116444	glutamate receptor, ionotropic, N-methyl-D-aspartate 3B [Source:HGNC Symbol;Acc:HGNC:16768]	
0.270822433270174	0.128009920553226	OR52L1	338751	olfactory receptor, family 52, subfamily L, member 1 [Source:HGNC Symbol;Acc:HGNC:14785]	
0.270772601480873	0.112408786278728	LINGO2	158038	leucine rich repeat and Ig domain containing 2 [Source:HGNC Symbol;Acc:HGNC:21207]	
0.270727732892604	0.158002221720289	IL1RL2	8808	interleukin 1 receptor-like 2 [Source:HGNC Symbol;Acc:HGNC:5999]	
0.270441356441534	0.143490056970865	CDRT1	374286	CMT1A duplicated region transcript 1 [Source:HGNC Symbol;Acc:HGNC:14379]	
0.270434055615359	0.187066172387228	MIR382	494331	microRNA 382 [Source:HGNC Symbol;Acc:HGNC:31875]	
0.270236231527238	0.140224940080709	CCDC37	348807	coiled-coil domain containing 37 [Source:HGNC Symbol;Acc:HGNC:26842]	
0.26994614606003	0.198266083041092	LRRC75B	388886	leucine rich repeat containing 75B [Source:HGNC Symbol;Acc:HGNC:33155]	
0.269806701911157	0.153934152788207	ZNF80	7634	zinc finger protein 80 [Source:HGNC Symbol;Acc:HGNC:13155]	
0.269700203389848	0.159526324816836	GSX1	219409	GS homeobox 1 [Source:HGNC Symbol;Acc:HGNC:20374]	
0.269418363677084	0.157951439193922	CTRC	11330	chymotrypsin C (caldecrin) [Source:HGNC Symbol;Acc:HGNC:2523]	
0.269182842745518	0.178438396271202	PHLDB3	653583	pleckstrin homology-like domain, family B, member 3 [Source:HGNC Symbol;Acc:HGNC:30499]	
0.269117587614109	0.103981391762695	OR2C3	81472	olfactory receptor, family 2, subfamily C, member 3 [Source:HGNC Symbol;Acc:HGNC:15005]	
0.26909481174352	0.0617111018587472	NUP210L	91181	nucleoporin 210kDa-like [Source:HGNC Symbol;Acc:HGNC:29915]	
0.268756015654693	0.194785039171931		84791		
0.26870995874984	0.178905852063974	ZAR1	326340	zygote arrest 1 [Source:HGNC Symbol;Acc:HGNC:20436]	
0.268613980894097	0.0926257131971839	TDRD9	122402	tudor domain containing 9 [Source:HGNC Symbol;Acc:HGNC:20122]	
0.268498787409223	0.081521962294009	HK3	3101	hexokinase 3 (white cell) [Source:HGNC Symbol;Acc:HGNC:4925]	
0.268345024333626	0.0337184488618398	ACOT12	134526	acyl-CoA thioesterase 12 [Source:HGNC Symbol;Acc:HGNC:24436]	
0.268320442185803	0.196521340734281	MIRLET7F2	406889	microRNA let-7f-2 [Source:HGNC Symbol;Acc:HGNC:31484]	
0.268233323302973	0.0712923948695867		100132731		
0.26821156446377	0.179868860039225	FAM179A	165186	family with sequence similarity 179, member A [Source:HGNC Symbol;Acc:HGNC:33715]	
0.268142025931348	0.0240368787937145	TAF1L	138474	TAF1 RNA polymerase II, TATA box binding protein (TBP)-associated factor, 210kDa-like [Source:HGNC Symbol;Acc:HGNC:18056]	
0.268132925126795	0.197805059166956	CCL17	6361	chemokine (C-C motif) ligand 17 [Source:HGNC Symbol;Acc:HGNC:10615]	
0.267947077079964	0.0863853398313466	GABRB1	2560	gamma-aminobutyric acid (GABA) A receptor, beta 1 [Source:HGNC Symbol;Acc:HGNC:4081]	
0.267660184514257	0.0649075579687425	CENPP	401541	centromere protein P [Source:HGNC Symbol;Acc:HGNC:32933]	
0.267512957147524	0.150460308027655	EN1	2019	engrailed homeobox 1 [Source:HGNC Symbol;Acc:HGNC:3342]	
0.267416530778948	0.0778258467379743	TNR	7143	tenascin R [Source:HGNC Symbol;Acc:HGNC:11953]	
0.267357177256823	0.163965871709342	CCDC36	339834	coiled-coil domain containing 36 [Source:HGNC Symbol;Acc:HGNC:27945]	
0.26702809357646	0.0761314046797512	RNASE9	390443	ribonuclease, RNase A family, 9 (non-active) [Source:HGNC Symbol;Acc:HGNC:20673]	
0.266695066911921	0.0647668853832497	PTTG2	10744	pituitary tumor-transforming 2 [Source:HGNC Symbol;Acc:HGNC:9691]	
0.266219558046123	0.127586128878334	TDRD10	126668	tudor domain containing 10 [Source:HGNC Symbol;Acc:HGNC:25316]	
0.26621684501924	0.199555357239202	FRS3	10817	fibroblast growth factor receptor substrate 3 [Source:HGNC Symbol;Acc:HGNC:16970]	
0.26603029413595	0.130724351725139	A2ML1	144568	alpha-2-macroglobulin-like 1 [Source:HGNC Symbol;Acc:HGNC:23336]	
0.265884201780905	0.194846806119331	ARRDC1-AS1	85026	ARRDC1 antisense RNA 1 [Source:HGNC Symbol;Acc:HGNC:23395]	
0.26585206067307	0.134900065157869		57578		
0.265664142011506	0.191992917115111	PNPLA3	80339	patatin-like phospholipase domain containing 3 [Source:HGNC Symbol;Acc:HGNC:18590]	TG !!!!
0.265628100754098	0.0837144489334199	LY6K	54742	lymphocyte antigen 6 complex, locus K [Source:HGNC Symbol;Acc:HGNC:24225]	
0.265404093176706	0.145410363125175	KRBA1	84626	KRAB-A domain containing 1 [Source:HGNC Symbol;Acc:HGNC:22228]	
0.265346849466276	0.192637159259475	HOXB3	3213	homeobox B3 [Source:HGNC Symbol;Acc:HGNC:5114]	
0.265121944309952	0.0517106283389703	GOT1L1	137362	glutamic-oxaloacetic transaminase 1-like 1 [Source:HGNC Symbol;Acc:HGNC:28487]	
0.265085188336915	0.162522545712751	OR6C68	403284	olfactory receptor, family 6, subfamily C, member 68 [Source:HGNC Symbol;Acc:HGNC:31297]	
0.265063764165146	0.138858836299142	CCDC24	149473	coiled-coil domain containing 24 [Source:HGNC Symbol;Acc:HGNC:28688]	
0.265061486394733	0.0939037097724186	GDF6	392255	growth differentiation factor 6 [Source:HGNC Symbol;Acc:HGNC:4221]	
0.264938348686471	0.0781815281073509	WDR88	126248	WD repeat domain 88 [Source:HGNC Symbol;Acc:HGNC:26999]	
0.264520288356655	0.00305451783798721	ZNF541	84215	zinc finger protein 541 [Source:HGNC Symbol;Acc:HGNC:25294]	
0.264513536050485	0.150027930812343	HTR5A	3361	5-hydroxytryptamine (serotonin) receptor 5A, G protein-coupled [Source:HGNC Symbol;Acc:HGNC:5300]	
0.264460708472075	0.102265133389352	DCST2	127579	DC-STAMP domain containing 2 [Source:HGNC Symbol;Acc:HGNC:26562]	
0.264302411335664	0.162876736636966	HBQ1	3049	hemoglobin, theta 1 [Source:HGNC Symbol;Acc:HGNC:4833]	
0.263994345950158	0.153479442382697	SPANXB2	728695	SPANX family, member B2 [Source:HGNC Symbol;Acc:HGNC:14330]	
0.263889193459872	0.198326311703055	IL17REL	400935	interleukin 17 receptor E-like [Source:HGNC Symbol;Acc:HGNC:33808]	
0.263372677995312	0.19195765708702	ICAM5	7087	intercellular adhesion molecule 5, telencephalin [Source:HGNC Symbol;Acc:HGNC:5348]	
0.263346805504294	0.0929090575820148		149950		
0.263278847906558	0.0213445433687126	ZIC4	84107	Zic family member 4 [Source:HGNC Symbol;Acc:HGNC:20393]	
0.263107594347043	0.148594710643786	KLHL14	57565	kelch-like family member 14 [Source:HGNC Symbol;Acc:HGNC:29266]	
0.262978246279483	0.0373538498790086	PITX2	5308	paired-like homeodomain 2 [Source:HGNC Symbol;Acc:HGNC:9005]	
0.262924102939068	0.0443600282449004	APOC3	345	apolipoprotein C-III [Source:HGNC Symbol;Acc:HGNC:610]	
0.262885777271002	0.143037961072428	USHBP1	83878	Usher syndrome 1C binding protein 1 [Source:HGNC Symbol;Acc:HGNC:24058]	
0.262712577186153	0.102380293575641	TRPV6	55503	transient receptor potential cation channel, subfamily V, member 6 [Source:HGNC Symbol;Acc:HGNC:14006]	
0.262629635709937	0.0715642984976751	VSTM1	284415	V-set and transmembrane domain containing 1 [Source:HGNC Symbol;Acc:HGNC:29455]	
0.262375243872988	0.165801320790166	S1PR4	8698	sphingosine-1-phosphate receptor 4 [Source:HGNC Symbol;Acc:HGNC:3170]	
0.262129876649851	0.182397342667901	PGLYRP3	114771	peptidoglycan recognition protein 3 [Source:HGNC Symbol;Acc:HGNC:30014]	
0.261965094168608	0.0416570856103302	PKD1L2	114780	polycystic kidney disease 1-like 2 [Source:HGNC Symbol;Acc:HGNC:21715]	
0.261796671130106	0.195944012308706	RABGEF1	27342	RAB guanine nucleotide exchange factor (GEF) 1 [Source:HGNC Symbol;Acc:HGNC:17676]	
0.261781588356474	0.00773519513903923	TCF15	6939	transcription factor 15 (basic helix-loop-helix) [Source:HGNC Symbol;Acc:HGNC:11627]	
0.261753744869782	0.126903015225667	S100A12	6283	S100 calcium binding protein A12 [Source:HGNC Symbol;Acc:HGNC:10489]	
0.261552439583443	0.140707297633151	C1orf146	388649	chromosome 1 open reading frame 146 [Source:HGNC Symbol;Acc:HGNC:24032]	
0.261391197538786	0.167258650946581	TNNI3	7137	troponin I type 3 (cardiac) [Source:HGNC Symbol;Acc:HGNC:11947]	
0.261341452376384	0.177575603665457	ONECUT2	9480	one cut homeobox 2 [Source:HGNC Symbol;Acc:HGNC:8139]	
0.261067588290639	0.187943325149305	ZNF784	147808	zinc finger protein 784 [Source:HGNC Symbol;Acc:HGNC:33111]	
0.260958285996079	0.158024573973576	CLUL1	27098	clusterin-like 1 (retinal) [Source:HGNC Symbol;Acc:HGNC:2096]	
0.260947307614208	0.12382516872048	GBX1	2636	gastrulation brain homeobox 1 [Source:HGNC Symbol;Acc:HGNC:4185]	
0.260931648243579	0.196568937644714	PCDHB1	29930	protocadherin beta 1 [Source:HGNC Symbol;Acc:HGNC:8680]	
0.260797680815118	0.198341071287192	NMUR1	10316	neuromedin U receptor 1 [Source:HGNC Symbol;Acc:HGNC:4518]	
0.260749906674332	0.141535764609805	MMP26	56547	matrix metallopeptidase 26 [Source:HGNC Symbol;Acc:HGNC:14249]	
0.260581856635082	0.128553972030329	DHDH	27294	dihydrodiol dehydrogenase (dimeric) [Source:HGNC Symbol;Acc:HGNC:17887]	
0.260420422685029	0.0162689605882179	KCNK9	51305	potassium channel, subfamily K, member 9 [Source:HGNC Symbol;Acc:HGNC:6283]	
0.260075362416428	0.148848177652585	IGLON5	402665	IgLON family member 5 [Source:HGNC Symbol;Acc:HGNC:34550]	
0.260043041132148	0.179399783980131	BMP6	654	bone morphogenetic protein 6 [Source:HGNC Symbol;Acc:HGNC:1073]	
0.259759492928511	0.117029055851206	PCDHB16	57717	protocadherin beta 16 [Source:HGNC Symbol;Acc:HGNC:14546]	
0.259735673816456	0.124935016671549	ACOXL	55289	acyl-CoA oxidase-like [Source:HGNC Symbol;Acc:HGNC:25621]	
0.259684181848333	0.140737874820236	FMR1NB	158521	fragile X mental retardation 1 neighbor [Source:HGNC Symbol;Acc:HGNC:26372]	
0.259317885757129	0.0530277140683301	DNAH2	146754	dynein, axonemal, heavy chain 2 [Source:HGNC Symbol;Acc:HGNC:2948]	
0.259240494914727	0.193848505931711	EFCC1	79825	EF-hand and coiled-coil domain containing 1 [Source:HGNC Symbol;Acc:HGNC:25692]	
0.259236032065047	0.160217547372982	LRRC74B	400891	leucine rich repeat containing 74B [Source:HGNC Symbol;Acc:HGNC:34301]	
0.259058027817591	0.172880013442264	LIG1	3978	ligase I, DNA, ATP-dependent [Source:HGNC Symbol;Acc:HGNC:6598]	
0.25902895010833	0.100139613293946	GAL3ST3	89792	galactose-3-O-sulfotransferase 3 [Source:HGNC Symbol;Acc:HGNC:24144]	
0.25861140624637	0.174330337393568	CYB561D1	284613	cytochrome b561 family, member D1 [Source:HGNC Symbol;Acc:HGNC:26804]	
0.258592703355261	0.171874244326777	MDM4	4194	MDM4, p53 regulator [Source:HGNC Symbol;Acc:HGNC:6974]	
0.258474101608314	0.0799368572316378	NPRL3	8131	nitrogen permease regulator-like 3 (S. cerevisiae) [Source:HGNC Symbol;Acc:HGNC:14124]	
0.258350482847129	0.075440278194647	ODF2	4957	outer dense fiber of sperm tails 2 [Source:HGNC Symbol;Acc:HGNC:8114]	
0.258078036111881	0.0937390099119416	SPACA3	124912	sperm acrosome associated 3 [Source:HGNC Symbol;Acc:HGNC:16260]	
0.257663041951778	0.19372944675966	MYT1	4661	myelin transcription factor 1 [Source:HGNC Symbol;Acc:HGNC:7622]	
0.257399621496805	0.0992738457639562	NEIL2	252969	nei endonuclease VIII-like 2 (E. coli) [Source:HGNC Symbol;Acc:HGNC:18956]	
0.256919857994455	0.12103270718371	MBL2	4153	mannose-binding lectin (protein C) 2, soluble [Source:HGNC Symbol;Acc:HGNC:6922]	
0.256836125114521	0.0941528802891397	RIMS3	9783	regulating synaptic membrane exocytosis 3 [Source:HGNC Symbol;Acc:HGNC:21292]	
0.256603570840541	0.141472020088205	MRPL4	51073	mitochondrial ribosomal protein L4 [Source:HGNC Symbol;Acc:HGNC:14276]	
0.256449614530698	0.0489494258636201	JPH3	57338	junctophilin 3 [Source:HGNC Symbol;Acc:HGNC:14203]	
0.256387368856266	0.0901149997279047	MYCBPAP	84073	MYCBP associated protein [Source:HGNC Symbol;Acc:HGNC:19677]	
0.256172030134054	0.0154717367523448	PDCL2	132954	phosducin-like 2 [Source:HGNC Symbol;Acc:HGNC:29524]	
0.256126807118608	0.0408756370139881	PRSS55	203074	protease, serine, 55 [Source:HGNC Symbol;Acc:HGNC:30824]	
0.256071534064716	0.162840009558329	COLGALT2	23127	collagen beta(1-O)galactosyltransferase 2 [Source:HGNC Symbol;Acc:HGNC:16790]	
0.256061844614982	0.176496612702053	JRK	8629	Jrk homolog (mouse) [Source:HGNC Symbol;Acc:HGNC:6199]	
0.255901917607866	0.112203677173144	CRMP1	1400	collapsin response mediator protein 1 [Source:HGNC Symbol;Acc:HGNC:2365]	
0.255721165060185	0.00686918955699613	CYP3A43	64816	cytochrome P450, family 3, subfamily A, polypeptide 43 [Source:HGNC Symbol;Acc:HGNC:17450]	
0.255320041210754	0.181259570362779	ANO1	55107	anoctamin 1, calcium activated chloride channel [Source:HGNC Symbol;Acc:HGNC:21625]	
0.255228923118799	0.0950338139503522	RNASE7	84659	ribonuclease, RNase A family, 7 [Source:HGNC Symbol;Acc:HGNC:19278]	
0.255185172551983	0.184937544623184	DLK1	8788	delta-like 1 homolog (Drosophila) [Source:HGNC Symbol;Acc:HGNC:2907]	
0.255129435485381	0.0564814586903747	TACR1	6869	tachykinin receptor 1 [Source:HGNC Symbol;Acc:HGNC:11526]	
0.255095350262182	0.198578615044912	EWSR1	2130	EWS RNA-binding protein 1 [Source:HGNC Symbol;Acc:HGNC:3508]	
0.255091285764431	0.100351606042012	CAPZA3	93661	capping protein (actin filament) muscle Z-line, alpha 3 [Source:HGNC Symbol;Acc:HGNC:24205]	
0.254988360862548	0.0857192380310653	RNF145	153830	ring finger protein 145 [Source:HGNC Symbol;Acc:HGNC:20853]	
0.25496164413356	0.108208767719452	WBSCR17	64409	Williams-Beuren syndrome chromosome region 17 [Source:HGNC Symbol;Acc:HGNC:16347]	
0.254944088737823	0.111336573027058	FBXO43	286151	F-box protein 43 [Source:HGNC Symbol;Acc:HGNC:28521]	
0.254861094825639	0.134596879654001	BATF	10538	basic leucine zipper transcription factor, ATF-like [Source:HGNC Symbol;Acc:HGNC:958]	
0.254290443630904	0.0428285468481586	MS4A15	219995	membrane-spanning 4-domains, subfamily A, member 15 [Source:HGNC Symbol;Acc:HGNC:28573]	

0.254248179365827	0.124504150483456	GUSBP5	441046	glucuronidase, beta pseudogene 5 [Source:HGNC Symbol;Acc:HGNC:42319]
0.253911679212239	0.00942570776603584	ACAD9	28976	acyl-CoA dehydrogenase family, member 9 [Source:HGNC Symbol;Acc:HGNC:21497]
0.253841542298619	0.171586777218372	CCS	9973	copper chaperone for superoxide dismutase [Source:HGNC Symbol;Acc:HGNC:1613]
0.253766515540547	0.0487701537166485	SCRT1	83482	scratch family zinc finger 1 [Source:HGNC Symbol;Acc:HGNC:15950]
0.25369089289654	0.136647013823606	OR10J5	127385	olfactory receptor, family 10, subfamily J, member 5 [Source:HGNC Symbol;Acc:HGNC:14993]
0.25339850340874	0.0782561828389618	OR7E111P	79315	olfactory receptor, family 7, subfamily E, member 111 pseudogene [Source:HGNC Symbol;Acc:HGNC:15344]
0.253379187524815	0.146248378982923	CCDC136	64753	coiled-coil domain containing 136 [Source:HGNC Symbol;Acc:HGNC:22225]
0.253366041504873	0.0714982324582797	ASGR1	432	asialoglycoprotein receptor 1 [Source:HGNC Symbol;Acc:HGNC:742]
0.253345710729046	0.188602498846202	SKAP1	8631	src kinase associated phosphoprotein 1 [Source:HGNC Symbol;Acc:HGNC:15605]
0.253251270796397	0.160545081478825	SPATA12	353324	spermatogenesis associated 12 [Source:HGNC Symbol;Acc:HGNC:23221]
0.253120413843177	0.133083010484355	LINC00207	388910	long intergenic non-protein coding RNA 207 [Source:HGNC Symbol;Acc:HGNC:37255]
0.252991392864247	0.199049178653618	IZUMO4	113177	IZUMO family member 4 [Source:HGNC Symbol;Acc:HGNC:26950]
0.252175087242729	0.1881935543145	FAM124B	79843	family with sequence similarity 124B [Source:HGNC Symbol;Acc:HGNC:26224]
0.252042763233689	0.176395220383664	SSPO	23145	SCO-spondin [Source:HGNC Symbol;Acc:HGNC:21998]
0.251936716256653	0.161717176006215	SRSF7	6432	serine/arginine-rich splicing factor 7 [Source:HGNC Symbol;Acc:HGNC:10789]
0.251625462606975	0.176324194178817	F11	2160	coagulation factor XI [Source:HGNC Symbol;Acc:HGNC:3529]
0.251563953934517	0.0876919194613635	C1orf200	644997	chromosome 1 open reading frame 200 [Source:HGNC Symbol;Acc:HGNC:32346]
0.25144558514008	0.0547218266200958	VWC2	375567	von Willebrand factor C domain containing 2 [Source:HGNC Symbol;Acc:HGNC:30200]
0.251332620189453	0.0746831498945271	PRICKLE3	4007	prickle homolog 3 (Drosophila) [Source:HGNC Symbol;Acc:HGNC:6645]
0.251271644538049	0.151152784924573	RHO	6010	rhodopsin [Source:HGNC Symbol;Acc:HGNC:10012]
0.25125805590948	0.108735456224373	CCDC84	338657	coiled-coil domain containing 84 [Source:HGNC Symbol;Acc:HGNC:30460]
0.251166916913769	0.180461236335739	PIGL	9487	phosphatidylinositol glycan anchor biosynthesis, class L [Source:HGNC Symbol;Acc:HGNC:8966]
0.251064501865471	0.130420354662971	CABYR	26256	calcium binding tyrosine-(Y)-phosphorylation regulated [Source:HGNC Symbol;Acc:HGNC:15569]
0.250634040248631	0.163106474096597	FAM131B	9715	family with sequence similarity 131, member B [Source:HGNC Symbol;Acc:HGNC:22202]
0.250362312825414	0.10311675325833		339059	
0.250230152405998	0.085609861314806	LMO1	4004	LIM domain only 1 (rhombotin 1) [Source:HGNC Symbol;Acc:HGNC:6641]
0.250151108297577	0.11549189925867	GALNTL5	168391	polypeptide N-acetylgalactosaminyltransferase-like 5 [Source:HGNC Symbol;Acc:HGNC:21725]
0.250089666853231	0.108339193602166	FAM212B	55924	family with sequence similarity 212, member B [Source:HGNC Symbol;Acc:HGNC:28045]
0.250023298076639	0.145704922482053	SLC25A45	283130	solute carrier family 25, member 45 [Source:HGNC Symbol;Acc:HGNC:27442]
0.249906977408805	0.0455163684755145	GRIP2	80852	glutamate receptor interacting protein 2 [Source:HGNC Symbol;Acc:HGNC:23841]
0.249355891891704	0.135870727795791	HIST1H2BA	255626	histone cluster 1, H2ba [Source:HGNC Symbol;Acc:HGNC:18730]
0.249324050505108	0.0536908154191486	GPALPP1	55425	GPALPP motifs containing 1 [Source:HGNC Symbol;Acc:HGNC:20298]
0.249287768512333	0.145669134261336	SLAMF9	89886	SLAM family member 9 [Source:HGNC Symbol;Acc:HGNC:18430]
0.249228470504953	0.113580295578099	PPAPDC1A	196051	phosphatidic acid phosphatase type 2 domain containing 1A [Source:HGNC Symbol;Acc:HGNC:23531]
0.249227877701321	0.0760536105608682	L1TD1	54596	LINE-1 type transposase domain containing 1 [Source:HGNC Symbol;Acc:HGNC:25595]
0.249152034094256	0.0450378471828353	IL21R	50615	interleukin 21 receptor [Source:HGNC Symbol;Acc:HGNC:6006]
0.249118164775196	0.199106268782756	TSHR	7253	thyroid stimulating hormone receptor [Source:HGNC Symbol;Acc:HGNC:12373]
0.249063001613082	0.114122208913319	RNF17	56163	ring finger protein 17 [Source:HGNC Symbol;Acc:HGNC:10060]
0.249028976373384	0.138998126795741	C17orf97	400566	chromosome 17 open reading frame 97 [Source:HGNC Symbol;Acc:HGNC:33800]
0.248991758948877	0.138179342963034	HOXC6	3223	homeobox C6 [Source:HGNC Symbol;Acc:HGNC:5128]
0.248980462798745	0.105109258119552	KRTAP3-1	83896	keratin associated protein 3-1 [Source:HGNC Symbol;Acc:HGNC:16778]
0.248896355473157	0.133778258730864	DMRTA2	63950	DMRT-like family A2 [Source:HGNC Symbol;Acc:HGNC:13908]
0.248785148020514	0.1417597022605	CAMKV	79012	CaM kinase-like vesicle-associated [Source:HGNC Symbol;Acc:HGNC:28788]
0.248716661935947	0.0822112694059256	PDZD2	23037	PDZ domain containing 2 [Source:HGNC Symbol;Acc:HGNC:18486]
0.248490019764382	0.0422756434010722	CEP63	80254	centrosomal protein 63kDa [Source:HGNC Symbol;Acc:HGNC:25815]
0.248429057966018	0.0865971948245109	FTMT	94033	ferritin mitochondrial [Source:HGNC Symbol;Acc:HGNC:17345]
0.248296236474528	0.10460604149517	RNU6-71P	100873774	RNA, U6 small nuclear 71, pseudogene [Source:HGNC Symbol;Acc:HGNC:42561]
0.248121381808285	0.138262218807368	DNTT	1791	DNA nucleotidylexotransferase [Source:HGNC Symbol;Acc:HGNC:2983]
0.248118940685835	0.0245439313113955	IGF2-AS	51214	IGF2 antisense RNA [Source:HGNC Symbol;Acc:HGNC:14062]
0.247939450424317	0.167752928862987	KCNAB3	9196	potassium voltage-gated channel, shaker-related subfamily, beta member 3 [Source:HGNC Symbol;Acc:HGNC:6230]
0.247931076798241	0.150246943966445	FAM184B	27146	family with sequence similarity 184, member B [Source:HGNC Symbol;Acc:HGNC:29235]
0.247644991950825	0.0924425135064848	MRGBP	55257	MRG/MORF4L binding protein [Source:HGNC Symbol;Acc:HGNC:15866]
0.247606301776412	0.144817229476704	FCAR	2204	Fc fragment of IgA, receptor for [Source:HGNC Symbol;Acc:HGNC:3608]
0.24741334961161	0.160845407674389	VMAC	400673	vimentin-type intermediate filament associated coiled-coil protein [Source:HGNC Symbol;Acc:HGNC:33803]
0.24741200953445	0.17464598101986	SRPK3	26576	SRSF protein kinase 3 [Source:HGNC Symbol;Acc:HGNC:11402]
0.247391316762697	0.185155716032524	TFR2	7036	transferrin receptor 2 [Source:HGNC Symbol;Acc:HGNC:11762]
0.247280606213305	0.088293544312137	C6orf118	168090	chromosome 6 open reading frame 118 [Source:HGNC Symbol;Acc:HGNC:21233]
0.247207099257417	0.185229624331119	PSMG1	8624	proteasome (prosome, macropain) assembly chaperone 1 [Source:HGNC Symbol;Acc:HGNC:3043]
0.247194388836441	0.120120396404491	QTRT1	81890	queuine tRNA-ribosyltransferase 1 [Source:HGNC Symbol;Acc:HGNC:23797]
0.247169432585264	0.131908140148593	WT1-AS	51352	WT1 antisense RNA [Source:HGNC Symbol;Acc:HGNC:18135]
0.247128282334469	0.133920348156072	UCMA	221044	upper zone of growth plate and cartilage matrix associated [Source:HGNC Symbol;Acc:HGNC:25205]
0.247027805245867	0.150196757433272	CALML5	51806	calmodulin-like 5 [Source:HGNC Symbol;Acc:HGNC:18180]
0.246957476777188	0.166075482509775	CCDC27	148870	coiled-coil domain containing 27 [Source:HGNC Symbol;Acc:HGNC:26546]
0.24693403866096	0.165144494135965	LRRC2	79442	leucine rich repeat containing 2 [Source:HGNC Symbol;Acc:HGNC:14676]
0.246835848844663	0.0575118486031385	CASP14	23581	caspase 14, apoptosis-related cysteine peptidase [Source:HGNC Symbol;Acc:HGNC:1502]
0.246695617049128	0.0533286830984839	UQCC3	790955	ubiquinol-cytochrome c reductase complex assembly factor 3 [Source:HGNC Symbol;Acc:HGNC:34399]
0.246185775304621	0.0668972041607783	ASTN1	460	astrotactin 1 [Source:HGNC Symbol;Acc:HGNC:773]
0.24615906995218	0.141690607781452	RAD51B	5890	RAD51 paralog B [Source:HGNC Symbol;Acc:HGNC:9822]
0.245850323870988	0.0353104468503318	VCPKMT	79609	valosin containing protein lysine (K) methyltransferase [Source:HGNC Symbol;Acc:HGNC:20352]
0.245751050843856	0.0990643680309017	KRTAP1-5	83895	keratin associated protein 1-5 [Source:HGNC Symbol;Acc:HGNC:16777]
0.245640116179736	0.148646033822821	TMEM174	134288	transmembrane protein 174 [Source:HGNC Symbol;Acc:HGNC:28187]
0.245441809288896	0.196166194657203	LHCGR	3973	luteinizing hormone/choriogonadotropin receptor [Source:HGNC Symbol;Acc:HGNC:6585]
0.245282182230911	0.0803767097989635	C1orf52	148423	chromosome 1 open reading frame 52 [Source:HGNC Symbol;Acc:HGNC:24871]
0.245197894754586	0.167275458268107	MAGEB18	286514	melanoma antigen family B, 18 [Source:HGNC Symbol;Acc:HGNC:28515]
0.244905962312301	0.185988315166091	GCDH	2639	glutaryl-CoA dehydrogenase [Source:HGNC Symbol;Acc:HGNC:4189]
0.244875425620192	0.0682091883875735	PAQR9	344838	progestin and adipoQ receptor family member IX [Source:HGNC Symbol;Acc:HGNC:30131]
0.244810792494647	0.0194379366605098	ATP6V0B	533	ATPase, H+ transporting, lysosomal 21kDa, V0 subunit b [Source:HGNC Symbol;Acc:HGNC:861]
0.244788708674588	0.0871137821697134	ETV2	2116	ets variant 2 [Source:HGNC Symbol;Acc:HGNC:3491]
0.244770318223741	0.0681141757252091	HAL	3034	histidine ammonia-lyase [Source:HGNC Symbol;Acc:HGNC:4806]
0.24425772499826	0.0883701488740562	C1orf127	148345	chromosome 1 open reading frame 127 [Source:HGNC Symbol;Acc:HGNC:26730]
0.244148081969954	0.180802042817955	C16orf71	146562	chromosome 16 open reading frame 71 [Source:HGNC Symbol;Acc:HGNC:25081]
0.244135260310883	0.111098338964233	SLC22A12	116085	solute carrier family 22 (organic anion/urate transporter), member 12 [Source:HGNC Symbol;Acc:HGNC:17989]
0.244129532911052	0.126581225386705	ABCC10	89845	ATP-binding cassette, sub-family C (CFTR/MRP), member 10 [Source:HGNC Symbol;Acc:HGNC:52]
0.24373367694982	0.0296555804655119	PADI6	353238	peptidyl arginine deiminase, type VI [Source:HGNC Symbol;Acc:HGNC:20449]
0.243729291575981	0.0153183898117959	CETP	1071	cholesteryl ester transfer protein, plasma [Source:HGNC Symbol;Acc:HGNC:1869]
0.243301934258125	0.123125845482238	C3orf67	200844	chromosome 3 open reading frame 67 [Source:HGNC Symbol;Acc:HGNC:24763]
0.243088616967539	0.0883635956759513	TNP2	7142	transition protein 2 (during histone to protamine replacement) [Source:HGNC Symbol;Acc:HGNC:11952]
0.242073383650327	0.0984311117868829	MAGOH	4116	mago-nashi homolog, proliferation-associated (Drosophila) [Source:HGNC Symbol;Acc:HGNC:6815]
0.242058493916265	0.186619591722378	RFTN1	23180	raftlin, lipid raft linker 1 [Source:HGNC Symbol;Acc:HGNC:30278]
0.241835578067254	0.158020427205391	EFHB	151651	EF-hand domain family, member B [Source:HGNC Symbol;Acc:HGNC:26330]
0.241718285763952	0.199504305533358		414235	
0.241691344587295	0.128069226278892	SUPT7L	9913	suppressor of Ty 7 (S. cerevisiae)-like [Source:HGNC Symbol;Acc:HGNC:30632]
0.241669310661369	0.0715835322917759	FOXN1	8456	forkhead box N1 [Source:HGNC Symbol;Acc:HGNC:12765]
0.241531656668867	0.106040041847625	KIAA0319	9856	KIAA0319 [Source:HGNC Symbol;Acc:HGNC:21580]
0.241348757241128	0.0535246649204525	PLCH2	9651	phospholipase C, eta 2 [Source:HGNC Symbol;Acc:HGNC:29037]
0.241088477537324	0.144979929190709	NXF5	55998	nuclear RNA export factor 5 [Source:HGNC Symbol;Acc:HGNC:8075]
0.241071722016064	0.109074188668721	CLIC3	9022	chloride intracellular channel 3 [Source:HGNC Symbol;Acc:HGNC:2064]
0.240873098863922	0.132909936633861	CALHM1	255022	calcium homeostasis modulator 1 [Source:HGNC Symbol;Acc:HGNC:23494]
0.240601171857848	0.199235995305696	SLC25A37	51312	solute carrier family 25 (mitochondrial iron transporter), member 37 [Source:HGNC Symbol;Acc:HGNC:29786]
0.240369805373037	0.152638837314379	TEKT2	27285	tektin 2 (testicular) [Source:HGNC Symbol;Acc:HGNC:11725]
0.240307801526009	0.00437046419439162	SH3GL3	6457	SH3-domain GRB2-like 3 [Source:HGNC Symbol;Acc:HGNC:10832]
0.240097654972012	0.131921601154158	ZNF645	158506	zinc finger protein 645 [Source:HGNC Symbol;Acc:HGNC:26371]
0.24008697757747	0.144062986385272	NKX2-6	137814	NK2 homeobox 6 [Source:HGNC Symbol;Acc:HGNC:32940]
0.240026784397073	0.192744954528855	VPS37D	155382	vacuolar protein sorting 37 homolog D (S. cerevisiae) [Source:HGNC Symbol;Acc:HGNC:18287]
0.239928412328591	0.131399341173372	ABCC11	85320	ATP-binding cassette, sub-family C (CFTR/MRP), member 11 [Source:HGNC Symbol;Acc:HGNC:14639]
0.239837153807004	0.114608444571411	OBSCN	84033	obscurin, cytoskeletal calmodulin and titin-interacting RhoGEF [Source:HGNC Symbol;Acc:HGNC:15719]
0.23969316174221	0.193047105599761	WDR38	401551	WD repeat domain 38 [Source:HGNC Symbol;Acc:HGNC:23745]
0.239669506850621	0.173112889122243	CHRNA6	8973	cholinergic receptor, nicotinic, alpha 6 (neuronal) [Source:HGNC Symbol;Acc:HGNC:15963]
0.239552106128308	0.0715758731843292	PHOSPHO1	162466	phosphatase, orphan 1 [Source:HGNC Symbol;Acc:HGNC:16815]
0.239342670863442	0.175379993419132	ALDOB	229	aldolase B, fructose-bisphosphate [Source:HGNC Symbol;Acc:HGNC:417]
0.239341617720787	0.0322576673253168	TEX101	83639	testis expressed 101 [Source:HGNC Symbol;Acc:HGNC:30722]
0.239272535511882	0.189348380249531	GRHL1	29841	grainyhead-like 1 (Drosophila) [Source:HGNC Symbol;Acc:HGNC:17923]
0.239192104590399	0.123460283854929	C1orf64	149563	chromosome 1 open reading frame 64 [Source:HGNC Symbol;Acc:HGNC:28339]
0.239088674968256	0.180428848457987	ARHGAP33	115703	Rho GTPase activating protein 33 [Source:HGNC Symbol;Acc:HGNC:23085]
0.238953226079889	0.112657612200147	GFOD1	54438	glucose-fructose oxidoreductase domain containing 1 [Source:HGNC Symbol;Acc:HGNC:21096]
0.238645511896049	0.111910812689893	MEOX1	4222	mesenchyme homeobox 1 [Source:HGNC Symbol;Acc:HGNC:7013]
0.238572637587757	0.186325601519491	PACSIN3	29763	protein kinase C and casein kinase substrate in neurons 3 [Source:HGNC Symbol;Acc:HGNC:8572]
0.238351041475758	0.0181231080839118	FIGLA	344018	folliculogenesis specific basic helix-loop-helix [Source:HGNC Symbol;Acc:HGNC:24669]
0.238200456346461	0.0240015420134666		23370	Rho guanine nucleotide exchange factor 18 [Source:UniProtKB/TrEMBL;Acc:M0R125]
0.238047926568216	0.048050065268297	PRSS45	377047	protease, serine, 45 [Source:HGNC Symbol;Acc:HGNC:30717]
0.23784734533299	0.148449385568536	ST3GAL3	6487	ST3 beta-galactoside alpha-2,3-sialyltransferase 3 [Source:HGNC Symbol;Acc:HGNC:10866]
0.237729806300479	0.175870214815457	WDR16	146845	WD repeat domain 16 [Source:HGNC Symbol;Acc:HGNC:16053]
0.237386979328472	0.0787387900506673	CELF2	10659	CUGBP, Elav-like family member 2 [Source:HGNC Symbol;Acc:HGNC:2550]
0.237122295666789	0.18076354553027	MRE11A	4361	MRE11 meiotic recombination 11 homolog A (S. cerevisiae) [Source:HGNC Symbol;Acc:HGNC:7230]
0.237020311568937	0.0925290611376002	CWC27	10283	CWC27 spliceosome-associated protein homolog (S. cerevisiae) [Source:HGNC Symbol;Acc:HGNC:10664]
0.236728530610451	0.0825969450217248	TGM7	116179	transglutaminase 7 [Source:HGNC Symbol;Acc:HGNC:30790]
0.236495246995926	0.0689919759542301	EMX2	2018	empty spiracles homeobox 2 [Source:HGNC Symbol;Acc:HGNC:3341]
0.236484220862473	0.0987677681575136	SLX4IP	128710	SLX4 interacting protein [Source:HGNC Symbol;Acc:HGNC:16225]
0.236471024158985	0.166633697339121	HSH2D	84941	hematopoietic SH2 domain containing [Source:HGNC Symbol;Acc:HGNC:24920]
0.236198578655729	0.123614684310145	C1orf194	127003	chromosome 1 open reading frame 194 [Source:HGNC Symbol;Acc:HGNC:32331]
0.236092166074541	0.197601966457603	MIR128-1	406915	microRNA 128-1 [Source:HGNC Symbol;Acc:HGNC:31510]
0.236027692312521	0.143379536901999	WNT9A	7483	wingless-type MMTV integration site family, member 9A [Source:HGNC Symbol;Acc:HGNC:12778]
0.235812319839942	0.184950973970303	EPB42	2038	erythrocyte membrane protein band 4.2 [Source:HGNC Symbol;Acc:HGNC:3381]
0.235516461815002	0.0965128726593414	C8B	732	complement component 8, beta polypeptide [Source:HGNC Symbol;Acc:HGNC:1353]
0.235463640530977	0.168194906954519		145757	
0.234922028221483	0.196215696715632		100133315	
0.234799767028107	0.115366291211052	PADI4	23569	peptidyl arginine deiminase, type IV [Source:HGNC Symbol;Acc:HGNC:18368]
0.234481187926002	0.0291246002282386	ARHGAP36	158763	Rho GTPase activating protein 36 [Source:HGNC Symbol;Acc:HGNC:26388]
0.234117851905601	0.148546776210161	CST9	128822	cystatin 9 (testatin) [Source:HGNC Symbol;Acc:HGNC:13261]
0.233958657312798	0.176640383256004		140883	
0.233923802849222	0.0660096265251968	MYO1H	283446	myosin IH [Source:HGNC Symbol;Acc:HGNC:13879]

0.233833449570628	0.0669280249299389	RPAIN	84268	RPA interacting protein [Source:HGNC Symbol;Acc:HGNC:28641]
0.233800835243261	0.0972229575955376	ZNF681	148213	zinc finger protein 681 [Source:HGNC Symbol;Acc:HGNC:26457]
0.23370204163171	0.129652145500326	NMB	4828	neuromedin B [Source:HGNC Symbol;Acc:HGNC:7842]
0.233638525987436	0.129621136430937	RBM14	10432	RNA binding motif protein 14 [Source:HGNC Symbol;Acc:HGNC:14219]
0.233594483179122	0.12799677160367	CHTOP	26097	chromatin target of PRMT1 [Source:HGNC Symbol;Acc:HGNC:24511]
0.233289091101688	0.0742971659959556	DBX2	440097	developing brain homeobox 2 [Source:HGNC Symbol;Acc:HGNC:33186]
0.232668678455401	0.183878081335567	TLR9	54106	toll-like receptor 9 [Source:HGNC Symbol;Acc:HGNC:15633]
0.232606748475675	0.0786923931025801	CLNK	116449	cytokine-dependent hematopoietic cell linker [Source:HGNC Symbol;Acc:HGNC:17438]
0.232590576239164	0.0938831155075204	ARMC3	219681	armadillo repeat containing 3 [Source:HGNC Symbol;Acc:HGNC:30964]
0.232360467978319	0.162077775691465	SLC28A1	9154	solute carrier family 28 (concentrative nucleoside transporter), member 1 [Source:HGNC Symbol;Acc:HGNC:11001]
0.232143272418506	0.133828560105032	sept-03	55964	septin 3 [Source:HGNC Symbol;Acc:HGNC:10750]
0.232097746648216	0.169846056828658	C1orf141	400757	chromosome 1 open reading frame 141 [Source:HGNC Symbol;Acc:HGNC:32044]
0.232075908726795	0.103526754167039	SCGB1D2	10647	secretoglobin, family 1D, member 2 [Source:HGNC Symbol;Acc:HGNC:18396]
0.231880169461247	0.193165978383389		100128124	
0.231842833492955	0.078613781420164	TNFSF11	8600	tumor necrosis factor (ligand) superfamily, member 11 [Source:HGNC Symbol;Acc:HGNC:11926]
0.231712775093193	0.189283897223352	VSX2	338917	visual system homeobox 2 [Source:HGNC Symbol;Acc:HGNC:1975]
0.231619697788234	0.0979673111653463	PRPF3	9129	pre-mRNA processing factor 3 [Source:HGNC Symbol;Acc:HGNC:17348]
0.231603343302591	0.0834919951757926	GALNT8	3742	polypeptide N-acetylgalactosaminyltransferase 8 [Source:HGNC Symbol;Acc:HGNC:4130]
0.231428442816065	0.118080452091484	KRT3	3850	keratin 3 [Source:HGNC Symbol;Acc:HGNC:6440]
0.231423255407235	0.0694177406911756	SNX31	169166	sorting nexin 31 [Source:HGNC Symbol;Acc:HGNC:28605]
0.2312794512646	0.15470020709372	ORM2	5005	orosomucoid 2 [Source:HGNC Symbol;Acc:HGNC:8499]
0.231251672853033	0.0463575478298285	NFATC2IP	84901	nuclear factor of activated T-cells, cytoplasmic, calcineurin-dependent 2 interacting protein [Source:HGNC Symbol;Acc:HGNC:25906]
0.230891364233696	0.0537670628861703	NAP1L3	4675	nucleosome assembly protein 1-like 3 [Source:HGNC Symbol;Acc:HGNC:7639]
0.230637966558745	0.147691104861779	LINC00656	200261	long intergenic non-protein coding RNA 656 [Source:HGNC Symbol;Acc:HGNC:27304]
0.230458521701909	0.13407038506315	GRM2	2912	glutamate receptor, metabotropic 2 [Source:HGNC Symbol;Acc:HGNC:4594]
0.230326280415056	0.139057684715343	C9orf131	138724	chromosome 9 open reading frame 131 [Source:HGNC Symbol;Acc:HGNC:31418]
0.230182748347261	0.0927039658185108	MCF2L	23263	MCF.2 cell line derived transforming sequence-like [Source:HGNC Symbol;Acc:HGNC:14576]
0.230163297711968	0.177106464367841	C9orf62	157927	chromosome 9 open reading frame 62 [Source:HGNC Symbol;Acc:HGNC:28581]
0.229648863891637	0.150096972485349	SUSD2	56241	sushi domain containing 2 [Source:HGNC Symbol;Acc:HGNC:30667]
0.229609574759483	0.0588800307872304	DUSP27	92235	dual specificity phosphatase 27 (putative) [Source:HGNC Symbol;Acc:HGNC:25034]
0.229587790667996	0.123276419681564	HMX1	3166	H6 family homeobox 1 [Source:HGNC Symbol;Acc:HGNC:5017]
0.22949508928284	0.0992215293943827	MYH3	4621	myosin, heavy chain 3, skeletal muscle, embryonic [Source:HGNC Symbol;Acc:HGNC:7573]
0.229365594554337	0.161186292360849	ASAH2	56624	N-acylsphingosine amidohydrolase (non-lysosomal ceramidase) 2 [Source:HGNC Symbol;Acc:HGNC:18860]
0.22902467875156	0.112487856334263	TMPRSS12	283471	transmembrane (C-terminal) protease, serine 12 [Source:HGNC Symbol;Acc:HGNC:28779]
0.22900151011445	0.146212632598546	GABRQ	55879	gamma-aminobutyric acid (GABA) A receptor, theta [Source:HGNC Symbol;Acc:HGNC:14454]
0.228790367096848	0.132836443610743	CCDC170	80129	coiled-coil domain containing 170 [Source:HGNC Symbol;Acc:HGNC:21177]
0.2287573293256	0.0246426077500494	RAB7B	338382	RAB7B, member RAS oncogene family [Source:HGNC Symbol;Acc:HGNC:30513]
0.228633536381581	0.182360681279307	SUOX	6821	sulfite oxidase [Source:HGNC Symbol;Acc:HGNC:11460]
0.228482511891187	0.187716127327343	ACTN3	89	actinin, alpha 3 (gene/pseudogene) [Source:HGNC Symbol;Acc:HGNC:165]
0.228409583909965	0.164307141658444	B3GNT4	79369	UDP-GlcNAc:betaGal beta-1,3-N-acetylglucosaminyltransferase 4 [Source:HGNC Symbol;Acc:HGNC:15683]
0.228302830863209	0.127838622601897	PDIA2	64714	protein disulfide isomerase family A, member 2 [Source:HGNC Symbol;Acc:HGNC:14180]
0.228090284468802	0.117609877020729	NOD1	10392	nucleotide-binding oligomerization domain containing 1 [Source:HGNC Symbol;Acc:HGNC:16390]
0.227879452497226	0.145921285686151	TG	7038	thyroglobulin [Source:HGNC Symbol;Acc:HGNC:11764]
0.227867796367634	0.0778114725622217	GJA10	84694	gap junction protein, alpha 10, 62kDa [Source:HGNC Symbol;Acc:HGNC:16995]
0.227604568148505	0.161633902124436	RNF25	64320	ring finger protein 25 [Source:HGNC Symbol;Acc:HGNC:14662]
0.22757656624461	0.0820997368488956	SSTR3	6753	somatostatin receptor 3 [Source:HGNC Symbol;Acc:HGNC:11332]
0.227563500264536	0.0872487991601404	AQP5	362	aquaporin 5 [Source:HGNC Symbol;Acc:HGNC:638]
0.226570942537682	0.170236421961118	QRICH2	84074	glutamine rich 2 [Source:HGNC Symbol;Acc:HGNC:25326]
0.226480999514365	0.0487939227917377	MED15	51586	mediator complex subunit 15 [Source:HGNC Symbol;Acc:HGNC:14248]
0.22646133515904	0.162932652121009	KCNN2	3781	potassium intermediate/small conductance calcium-activated channel, subfamily N, member 2 [Source:HGNC Symbol;Acc:HGNC:6291]
0.226224928520321	0.191085201330724	CGA	1081	glycoprotein hormones, alpha polypeptide [Source:HGNC Symbol;Acc:HGNC:1885]
0.226103430806271	0.185318537953433	TUBA4B	80086	tubulin, alpha 4b (pseudogene) [Source:HGNC Symbol;Acc:HGNC:18637]
0.226037676284616	0.159710405506941	TAZ	6901	tafazzin [Source:HGNC Symbol;Acc:HGNC:11577]
0.225666766517741	0.110876745870412	SLC7A14	57709	solute carrier family 7, member 14 [Source:HGNC Symbol;Acc:HGNC:29326]
0.225472004900729	0.0259896065712251	DYDC1	143241	DPY30 domain containing 1 [Source:HGNC Symbol;Acc:HGNC:23460]
0.225328000923185	0.174436731408981	CXCL6	6372	chemokine (C-X-C motif) ligand 6 [Source:HGNC Symbol;Acc:HGNC:10643]
0.224982054489113	0.0280775189259567	MIR451A	574411	microRNA 451b [Source:HGNC Symbol;Acc:HGNC:41655]
0.224940689331891	0.199706820253127	ENOSF1	55556	enolase superfamily member 1 [Source:HGNC Symbol;Acc:HGNC:30365]
0.224856761896564	0.109456725431648	UROC1	131669	urocanate hydratase 1 [Source:HGNC Symbol;Acc:HGNC:26444]
0.224785106529346	0.183226163147957	TP63	8626	tumor protein p63 [Source:HGNC Symbol;Acc:HGNC:15979]
0.224603108562894	0.143085478785726	C6orf201	404220	chromosome 6 open reading frame 201 [Source:HGNC Symbol;Acc:HGNC:21620]
0.224465511852518	0.16458913172783	PAQR6	79957	progestin and adipoQ receptor family member VI [Source:HGNC Symbol;Acc:HGNC:30132]
0.224280428319085	0.185320364952617	NOS1	4842	nitric oxide synthase 1 (neuronal) [Source:HGNC Symbol;Acc:HGNC:7872]
0.223739248321426	0.0926777785452834	SLC5A5	6528	solute carrier family 5 (sodium/iodide cotransporter), member 5 [Source:HGNC Symbol;Acc:HGNC:11040]
0.22370144261399	0.11564695342984	RPRM	56475	reprimo, TP53 dependent G2 arrest mediator candidate [Source:HGNC Symbol;Acc:HGNC:24201]
0.223633466195653	0.0585141477081908	MIR3074	407012	microRNA 3074 [Source:HGNC Symbol;Acc:HGNC:38268]
0.223613016858224	0.0846251326953525	EXD1	161829	exonuclease 3'-5' domain containing 1 [Source:HGNC Symbol;Acc:HGNC:28507]
0.22356785004877	0.148796787514803	AMER2	219287	APC membrane recruitment protein 2 [Source:HGNC Symbol;Acc:HGNC:26360]
0.223171702221089	0.149581704549163	ARHGAP27	201176	Rho GTPase activating protein 27 [Source:HGNC Symbol;Acc:HGNC:31813]
0.223121951943571	0.154723091589264	SLC2A11	66035	solute carrier family 2 (facilitated glucose transporter), member 11 [Source:HGNC Symbol;Acc:HGNC:14239]
0.222462000831031	0.147844023518749	C17orf47	284083	chromosome 17 open reading frame 47 [Source:HGNC Symbol;Acc:HGNC:26844]
0.222406408027906	0.144677639359585	C7orf43	55262	chromosome 7 open reading frame 43 [Source:HGNC Symbol;Acc:HGNC:25604]
0.222359175335461	0.147661442512303	TRIM7	81786	tripartite motif containing 7 [Source:HGNC Symbol;Acc:HGNC:16278]
0.222240868598634	0.149015820533634	CLK2	1196	CDC-like kinase 2 [Source:HGNC Symbol;Acc:HGNC:2069]
0.222090010319928	0.168187802939693	KAT2A	2648	K(lysine) acetyltransferase 2A [Source:HGNC Symbol;Acc:HGNC:4201]
0.221868603402758	0.0971597835567227	UCP3	7352	uncoupling protein 3 (mitochondrial, proton carrier) [Source:HGNC Symbol;Acc:HGNC:12519]
0.221843777749219	0.167384319600996	GALNT15	117248	polypeptide N-acetylgalactosaminyltransferase 15 [Source:HGNC Symbol;Acc:HGNC:21531]
0.221426441714331	0.18083731538283	CAPSL	133690	calcyphosine-like [Source:HGNC Symbol;Acc:HGNC:28375]
0.22116941408441	0.161151198536419	SNORA69	26779	small nucleolar RNA, H/ACA box 69 [Source:HGNC Symbol;Acc:HGNC:10226]
0.221022532987207	0.153432037193128	HSPB3	8988	heat shock 27kDa protein 3 [Source:HGNC Symbol;Acc:HGNC:5248]
0.22100514298993	0.0650274002267125	STRADA	92335	STE20-related kinase adaptor alpha [Source:HGNC Symbol;Acc:HGNC:30172]
0.220956782384351	0.157495840949503	HGNC:9979	10739	Ret finger protein-like 2 [Source:UniProtKB/Swiss-Prot;Acc:O75678]
0.220834476877115	0.118494120490395	SUSD4	55061	sushi domain containing 4 [Source:HGNC Symbol;Acc:HGNC:25470]
0.220501316753201	0.110137028496099		401554	
0.22041639668599	0.0669469337866972	CLDN24	100132463	claudin 24 [Source:HGNC Symbol;Acc:HGNC:37200]
0.220219652567548	0.0701634573478869	RNU5F-1	26828	RNA, U5F small nuclear 1 [Source:HGNC Symbol;Acc:HGNC:10216]
0.220030761609086	0.194881003747059	MTNR1B	4544	melatonin receptor 1B [Source:HGNC Symbol;Acc:HGNC:7464]
0.219926727807058	0.115431739005777	DPPA2	151871	developmental pluripotency associated 2 [Source:HGNC Symbol;Acc:HGNC:19197]
0.219667064450782	0.0323472492039678	MIR219B	407003	microRNA 219b [Source:HGNC Symbol;Acc:HGNC:41823]
0.219332764249483	0.16584673977185	C10orf71	118461	chromosome 10 open reading frame 71 [Source:HGNC Symbol;Acc:HGNC:26973]
0.219156751932225	0.178911547141136		9808	
0.218891582911523	0.0919078681800226	TRIM68	55128	tripartite motif containing 68 [Source:HGNC Symbol;Acc:HGNC:21161]
0.218856230948093	0.134796615604983	C12orf60	144608	chromosome 12 open reading frame 60 [Source:HGNC Symbol;Acc:HGNC:28726]
0.218681524005846	0.196437270577815	COL5A3	50509	collagen, type V, alpha 3 [Source:HGNC Symbol;Acc:HGNC:14864]
0.21859214099996	0.125123571120199	SERPINA7	6906	serpin peptidase inhibitor, clade A (alpha-1 antiproteinase, antitrypsin), member 7 [Source:HGNC Symbol;Acc:HGNC:11583]
0.218457071377828	0.124822444591604	PZP	5858	pregnancy-zone protein [Source:HGNC Symbol;Acc:HGNC:9750]
0.218396727024561	0.153982564590032	PRDM8	56978	PR domain containing 8 [Source:HGNC Symbol;Acc:HGNC:13993]
0.218321634423295	0.139610471865165	PDLIM4	8572	PDZ and LIM domain 4 [Source:HGNC Symbol;Acc:HGNC:16501]
0.218291681244675	0.182130469783237	COL22A1	169044	collagen, type XXII, alpha 1 [Source:HGNC Symbol;Acc:HGNC:22989]
0.218213337246868	0.108674928813254	WDR74	54663	WD repeat domain 74 [Source:HGNC Symbol;Acc:HGNC:25529]
0.218092808979489	0.169070550838914	COL6A6	131873	collagen, type VI, alpha 6 [Source:HGNC Symbol;Acc:HGNC:27023]
0.217978250979656	0.0336242789827504	NCKAP5L	57701	NCK-associated protein 5-like [Source:HGNC Symbol;Acc:HGNC:29321]
0.217646373168497	0.199575295398591	PRPH2	5961	peripherin 2 (retinal degeneration, slow) [Source:HGNC Symbol;Acc:HGNC:9942]
0.217540018918561	0.130572198278386	PPP1R42	286187	protein phosphatase 1, regulatory subunit 42 [Source:HGNC Symbol;Acc:HGNC:33732]
0.217085427186574	0.0130756324699198	EPHA8	2046	EPH receptor A8 [Source:HGNC Symbol;Acc:HGNC:3391]
0.216953552221133	0.191888295445801	METRN	79006	meteorin, glial cell differentiation regulator [Source:HGNC Symbol;Acc:HGNC:14151]
0.21679579063789	0.158217796572949	CPLX4	339302	complexin 4 [Source:HGNC Symbol;Acc:HGNC:24330]
0.216307150958054	0.198146693124767	CHCHD6	84303	coiled-coil-helix-coiled-coil-helix domain containing 6 [Source:HGNC Symbol;Acc:HGNC:28184]
0.216140050220763	0.148534909404377	SAMD4B	55095	sterile alpha motif domain containing 4B [Source:HGNC Symbol;Acc:HGNC:25492]
0.215861561320692	0.181759949707864	TOR2A	27433	torsin family 2, member A [Source:HGNC Symbol;Acc:HGNC:11996]
0.215710296432016	0.166479293604999	OR10AD1	121275	olfactory receptor, family 10, subfamily AD, member 1 [Source:HGNC Symbol;Acc:HGNC:14819]
0.215296855889288	0.151371716442554	SBK1	388228	SH3 domain binding kinase 1 [Source:HGNC Symbol;Acc:HGNC:17699]
0.215170029780125	0.187422815177942	ARID5A	10865	AT rich interactive domain 5A (MRF1-like) [Source:HGNC Symbol;Acc:HGNC:17361]
0.215067467889098	0.140069106530111	SH2D4B	387694	SH2 domain containing 4B [Source:HGNC Symbol;Acc:HGNC:31440]
0.214988866095765	0.10842712964627		124871	uncharacterized FLJ40194 [Source:EntrezGene;Acc:124871]
0.214674823394173	0.183326695639694	NUDT2	318	nudix (nucleoside diphosphate linked moiety X)-type motif 2 [Source:HGNC Symbol;Acc:HGNC:8049]
0.214287052143116	0.12646225677475	LRIT2	340745	leucine-rich repeat, immunoglobulin-like and transmembrane domains 2 [Source:HGNC Symbol;Acc:HGNC:23443]
0.214171580535885	0.175142281481134	EIF4E1B	253314	eukaryotic translation initiation factor 4E family member 1B [Source:HGNC Symbol;Acc:HGNC:33179]
0.214164053053594	0.181061365882416	HIST1H1T	3010	histone cluster 1, H1t [Source:HGNC Symbol;Acc:HGNC:4720]
0.213965100463388	0.186147684175938	CRNN	49860	cornulin [Source:HGNC Symbol;Acc:HGNC:1230]
0.21379290299618	0.171635638579271		79136	
0.213584271012179	0.128647945288421		339803	
0.213370654575396	0.163359469244468	B3GALNT1	8706	beta-1,3-N-acetylgalactosaminyltransferase 1 (globoside blood group) [Source:HGNC Symbol;Acc:HGNC:918]
0.213200924315245	0.155602646087114	TEKT5	146279	tektin 5 [Source:HGNC Symbol;Acc:HGNC:26554]
0.213132260331871	0.0401807951532879	LRRFIP1	9208	leucine rich repeat (in FLII) interacting protein 1 [Source:HGNC Symbol;Acc:HGNC:6702]
0.21296096561209	0.0742799600119523	TEX33	339669	testis expressed 33 [Source:HGNC Symbol;Acc:HGNC:28568]
0.212902637451509	0.146885389571999	CYP26A1	1592	cytochrome P450, family 26, subfamily A, polypeptide 1 [Source:HGNC Symbol;Acc:HGNC:2603]
0.212713113794114	0.118012663447146	TRAP1	101929751	TNF receptor-associated protein 1 [Source:HGNC Symbol;Acc:HGNC:16264]
0.212702379884045	0.143297860457579	DNAI1	27019	dynein, axonemal, intermediate chain 1 [Source:HGNC Symbol;Acc:HGNC:2954]
0.212280717050124	0.192965102874935	LINC00951	401260	long intergenic non-protein coding RNA 951 [Source:HGNC Symbol;Acc:HGNC:48662]
0.211940481139262	0.124464147244936	IGF2BP1	10642	insulin-like growth factor 2 mRNA binding protein 1 [Source:HGNC Symbol;Acc:HGNC:28866]
0.211535352275586	0.187911070778028	KLF2	10365	Kruppel-like factor 2 [Source:HGNC Symbol;Acc:HGNC:6347]
0.211189839833637	0.0207380215848943	OR6W1P	89883	olfactory receptor, family 6, subfamily W, member 1 pseudogene [Source:HGNC Symbol;Acc:HGNC:15091]
0.211173215758491	0.141213467735211	C1orf111	284680	chromosome 1 open reading frame 111 [Source:HGNC Symbol;Acc:HGNC:27648]
0.2106981607406	0.185893241660874	C14orf180	400258	chromosome 14 open reading frame 180 [Source:HGNC Symbol;Acc:HGNC:33795]
0.210498725768643	0.143579758411202	C4orf32	132720	chromosome 4 open reading frame 32 [Source:HGNC Symbol;Acc:HGNC:26813]
0.210344086876362	0.114355299136257	TULP2	7288	tubby like protein 2 [Source:HGNC Symbol;Acc:HGNC:12424]
0.209790764272975	0.174685993047073	LINC00885	401109	long intergenic non-protein coding RNA 885 [Source:HGNC Symbol;Acc:HGNC:48571]
0.209769877171135	0.182306427188305	GALNT9	50614	polypeptide N-acetylgalactosaminyltransferase 9 [Source:HGNC Symbol;Acc:HGNC:4131]
0.209538405024153	0.115948511396137	RPH3A	22895	rabphilin 3A [Source:HGNC Symbol;Acc:HGNC:17056]
0.209129794729695	0.074114415154908	IRGC	56269	immunity-related GTPase family, cinema [Source:HGNC Symbol;Acc:HGNC:28835]

0.209050289812867	0.0748219968704368	ZNF732	654254	zinc finger protein 732 [Source:HGNC Symbol;Acc:HGNC:37138]	
0.209025945126108	0.125315093960297	RP1L1	94137	retinitis pigmentosa 1-like 1 [Source:HGNC Symbol;Acc:HGNC:15946]	
0.208996503659121	0.185195261901418	ZBED9	114821	zinc finger, BED-type containing 9 [Source:HGNC Symbol;Acc:HGNC:13851]	
0.208958532147552	0.180822612727152	OR4D2	124538	olfactory receptor, family 4, subfamily D, member 2 [Source:HGNC Symbol;Acc:HGNC:8294]	
0.208340007162929	0.144289494778029	BPIFA4P	317716	BPI fold containing family A, member 4, pseudogene [Source:HGNC Symbol;Acc:HGNC:20469]	
0.208110180448538	0.0358700392084652	SNX5	27131	sorting nexin 5 [Source:HGNC Symbol;Acc:HGNC:14969]	
0.20766089959013	0.175124631605621	PDGFRL	5157	platelet-derived growth factor receptor-like [Source:HGNC Symbol;Acc:HGNC:8805]	
0.207475534415441	0.178705342624795	IP6K3	117283	inositol hexakisphosphate kinase 3 [Source:HGNC Symbol;Acc:HGNC:17269]	
0.207176103037794	0.105510371750544	MAK	4117	male germ cell-associated kinase [Source:HGNC Symbol;Acc:HGNC:6816]	
0.206259384281357	0.190056971411215	MIR34C	407042	microRNA 34c [Source:HGNC Symbol;Acc:HGNC:31637]	
0.20616487293611	0.171305740449993	GPATCH11	253635	G patch domain containing 11 [Source:HGNC Symbol;Acc:HGNC:26768]	
0.205503387924859	0.170276226986504	BRD7P3	23629	bromodomain containing 7 pseudogene 3 [Source:HGNC Symbol;Acc:HGNC:24171]	
0.205381526047344	0.180173454090125		100996535		
0.205286125681727	0.0990051370801624	PIWIL3	440822	piwi-like RNA-mediated gene silencing 3 [Source:HGNC Symbol;Acc:HGNC:18443]	
0.205201012390822	0.0107565909433439	CCDC105	126402	coiled-coil domain containing 105 [Source:HGNC Symbol;Acc:HGNC:26866]	
0.205111842227486	0.18055040684703	BOD1L2	284257	biorientation of chromosomes in cell division 1-like 2 [Source:HGNC Symbol;Acc:HGNC:28505]	
0.204768407828589	0.109716131556084	SHOX	6473	short stature homeobox [Source:HGNC Symbol;Acc:HGNC:10853]	
0.204734556554558	0.130427597953247	CETN1	1068	centrin, EF-hand protein, 1 [Source:HGNC Symbol;Acc:HGNC:1866]	
0.204572260122408	0.195557609510934	OLAH	55301	oleoyl-ACP hydrolase [Source:HGNC Symbol;Acc:HGNC:25625]	
0.204074823161334	0.0626161595895233	ATP1A4	480	ATPase, Na+/K+ transporting, alpha 4 polypeptide [Source:HGNC Symbol;Acc:HGNC:14073]	
0.203950591570271	0.191460623399334	HEPACAM	220296	hepatic and glial cell adhesion molecule [Source:HGNC Symbol;Acc:HGNC:26361]	
0.203927731677908	0.141857073352144	APOA1	335	apolipoprotein A-I [Source:HGNC Symbol;Acc:HGNC:600]	
0.202896597890513	0.172422256718205	ASB14	142686	ankyrin repeat and SOCS box containing 14 [Source:HGNC Symbol;Acc:HGNC:19766]	
0.202881331794491	0.171202494122849	HDAC4	9759	histone deacetylase 4 [Source:HGNC Symbol;Acc:HGNC:14063]	
0.202870238404038	0.193242337651115	ZNF474	133923	zinc finger protein 474 [Source:HGNC Symbol;Acc:HGNC:23245]	
0.20264601282141	0.121421184969817	TJAP1	93643	tight junction associated protein 1 (peripheral) [Source:HGNC Symbol;Acc:HGNC:17949]	PILT small intestine
0.20254038005591	0.123348424045746	LINC00544	440131	long intergenic non-protein coding RNA 544 [Source:HGNC Symbol;Acc:HGNC:43679]	
0.202441800826685	0.0540872277159071	SNORD115-1	100033453	small nucleolar RNA, C/D box 115-15 [Source:HGNC Symbol;Acc:HGNC:33034]	
0.20210146001714	0.19979152225207	OSR2	116039	odd-skipped related transciption factor 2 [Source:HGNC Symbol;Acc:HGNC:15830]	
0.201698649626472	0.197699238458543	ITGA10	8515	integrin, alpha 10 [Source:HGNC Symbol;Acc:HGNC:6135]	
0.200026911729619	0.183688072536946	CHD7	55636	chromodomain helicase DNA binding protein 7 [Source:HGNC Symbol;Acc:HGNC:20626]	
0.200005573431521	0.0621250022321971		7170		
0.199831693719255	0.0953748030535363	C9orf173	441476	chromosome 9 open reading frame 173 [Source:HGNC Symbol;Acc:HGNC:37285]	
0.199498765904505	0.0895210019557765	MPL	4352	MPL proto-oncogene, thrombopoietin receptor [Source:HGNC Symbol;Acc:HGNC:7217]	
0.199300893857162	0.192463004457033	AHSP	51327	alpha hemoglobin stabilizing protein [Source:HGNC Symbol;Acc:HGNC:18075]	
0.199071673401025	0.120660372912613	SHISA4	149345	shisa family member 4 [Source:HGNC Symbol;Acc:HGNC:27139]	
0.198372330559753	0.146737208290903	SYNM	23336	synemin, intermediate filament protein [Source:HGNC Symbol;Acc:HGNC:24466]	
0.198299631176741	0.0924201661157696	SAG	6295	S-antigen; retina and pineal gland (arrestin) [Source:HGNC Symbol;Acc:HGNC:10521]	
0.197911569428321	0.186101105629826	CCDC70	83446	coiled-coil domain containing 70 [Source:HGNC Symbol;Acc:HGNC:25303]	
0.197831410983844	0.160349017512289	RNF165	494470	ring finger protein 165 [Source:HGNC Symbol;Acc:HGNC:31696]	
0.19760016020685	0.164856236585654	XIRP1	165904	xin actin-binding repeat containing 1 [Source:HGNC Symbol;Acc:HGNC:14301]	
0.197294882165638	0.184680215349266	CTHRC1	115908	collagen triple helix repeat containing 1 [Source:HGNC Symbol;Acc:HGNC:18831]	
0.197246418226608	0.131914280459172	WNT8B	7479	wingless-type MMTV integration site family, member 8B [Source:HGNC Symbol;Acc:HGNC:12789]	
0.19693783741739	0.141618839229359	PRAMEF12	390999	PRAME family member 12 [Source:HGNC Symbol;Acc:HGNC:22125]	
0.196365184015845	0.169127376169081	CYP27C1	339761	cytochrome P450, family 27, subfamily C, polypeptide 1 [Source:HGNC Symbol;Acc:HGNC:33480]	
0.195472334828889	0.136238820215283	COG4	25839	component of oligomeric golgi complex 4 [Source:HGNC Symbol;Acc:HGNC:18620]	
0.194987999924621	0.125664224813776	GTPBP1	9567	GTP binding protein 1 [Source:HGNC Symbol;Acc:HGNC:4669]	
0.194838468739895	0.152037800880586	CTAGE1	64693	cutaneous T-cell lymphoma-associated antigen 1 [Source:HGNC Symbol;Acc:HGNC:24346]	
0.194469612873633	0.141210406752161	PNLDC1	154197	poly(A)-specific ribonuclease (PARN)-like domain containing 1 [Source:HGNC Symbol;Acc:HGNC:21185]	
0.194337161930689	0.184351297201163	AK9	221264	adenylate kinase 9 [Source:HGNC Symbol;Acc:HGNC:33814]	
0.194134038100107	0.14979227534774	EXOSC2	23404	exosome component 2 [Source:HGNC Symbol;Acc:HGNC:17097]	
0.193689962018566	0.0767810309468935	ZNF232	7775	zinc finger protein 232 [Source:HGNC Symbol;Acc:HGNC:13026]	
0.193662318184872	0.182966419271919	GATA5	140628	GATA binding protein 5 [Source:HGNC Symbol;Acc:HGNC:15802]	
0.19351426726433	0.0751916083017695	IRS4	8471	insulin receptor substrate 4 [Source:HGNC Symbol;Acc:HGNC:6128]	
0.193256553495665	0.150812031125931	RUSC2	9853	RUN and SH3 domain containing 2 [Source:HGNC Symbol;Acc:HGNC:23625]	
0.193148032721427	0.0740253427337313	B4GALT5	9334	UDP-Gal:betaGlcNAc beta 1,4- galactosyltransferase, polypeptide 5 [Source:HGNC Symbol;Acc:HGNC:928]	
0.193082692548073	0.0287200991984437	NLRP12	91662	NLR family, pyrin domain containing 12 [Source:HGNC Symbol;Acc:HGNC:22938]	
0.193073692647418	0.199642026593185	SEMA5B	54437	sema domain, seven thrombospondin repeats (type 1 and type 1-like), transmembrane domain (TM) and short cytoplasmic domain, (semaphorin) 5B [Source:HGNC Symbol;Acc:HGNC:10737]	
0.192997622237746	0.162754050554283	C11orf85	283129	chromosome 11 open reading frame 85 [Source:HGNC Symbol;Acc:HGNC:27441]	
0.192720105712319	0.178277595535827	IFFO1	25900	intermediate filament family orphan 1 [Source:HGNC Symbol;Acc:HGNC:24970]	
0.192565657452107	0.145055077118572	CHST4	10164	carbohydrate (N-acetylglucosamine 6-O) sulfotransferase 4 [Source:HGNC Symbol;Acc:HGNC:1972]	
0.192260762353572	0.114057446797301	VIPR2	7434	vasoactive intestinal peptide receptor 2 [Source:HGNC Symbol;Acc:HGNC:12695]	
0.191675595979584	0.0798268236501691	SNORD115-2	100033437	small nucleolar RNA, C/D box 115-2 [Source:HGNC Symbol;Acc:HGNC:33021]	
0.191380959301769	0.022199536268168	SPEG	10290	SPEG complex locus [Source:HGNC Symbol;Acc:HGNC:16901]	
0.191301380800487	0.12707954576364	DCLRE1C	64421	DNA cross-link repair 1C [Source:HGNC Symbol;Acc:HGNC:17642]	
0.19114119585264	0.163440671272248	PSMC5	5705	proteasome (prosome, macropain) 26S subunit, ATPase, 5 [Source:HGNC Symbol;Acc:HGNC:9552]	
0.191078110690959	0.111345291800952	MPP2	4355	membrane protein, palmitoylated 2 (MAGUK p55 subfamily member 2) [Source:HGNC Symbol;Acc:HGNC:7220]	
0.190837669449679	0.0977530584223989	SPATA31D1	389763	SPATA31 subfamily D, member 1 [Source:HGNC Symbol;Acc:HGNC:37283]	
0.190592929516576	0.186729617470934	IQCC	55721	IQ motif containing C [Source:HGNC Symbol;Acc:HGNC:25545]	
0.189963052510209	0.157013731641619	MKL1	57591	megakaryoblastic leukemia (translocation) 1 [Source:HGNC Symbol;Acc:HGNC:14334]	
0.189933612227531	0.146076325323477	PDILT	204474	protein disulfide isomerase-like, testis expressed [Source:HGNC Symbol;Acc:HGNC:27338]	
0.189132409952855	0.162106237518086	EMILIN3	90187	elastin microfibril interfacer 3 [Source:HGNC Symbol;Acc:HGNC:16123]	
0.188825705423487	0.0778702882322301	TRPT1	83707	tRNA phosphotransferase 1 [Source:HGNC Symbol;Acc:HGNC:20316]	
0.188270730842714	0.187997278123238	LDHAL6B	92483	lactate dehydrogenase A-like 6B [Source:HGNC Symbol;Acc:HGNC:21481]	
0.187938149217401	0.137611373316478	CCK	885	cholecystokinin [Source:HGNC Symbol;Acc:HGNC:1569]	
0.187556977679069	0.129205417113669	MRPL14	64928	mitochondrial ribosomal protein L14 [Source:HGNC Symbol;Acc:HGNC:14279]	
0.186721862811455	0.0352998878328364	ATXN2L	11273	ataxin 2-like [Source:HGNC Symbol;Acc:HGNC:31326]	
0.186214095090291	0.16178233434863	LINC00277	283673	long intergenic non-protein coding RNA 277 [Source:HGNC Symbol;Acc:HGNC:26596]	
0.185967501012697	0.160863837347121	GATA4	2626	GATA binding protein 4 [Source:HGNC Symbol;Acc:HGNC:4173]	
0.185663509561613	0.171525332454975	NPHS2	7827	nephrosis 2, idiopathic, steroid-resistant (podocin) [Source:HGNC Symbol;Acc:HGNC:13394]	
0.184272831341817	0.111501450048331	NEUROG2	63973	neurogenin 2 [Source:HGNC Symbol;Acc:HGNC:13805]	
0.184050910931741	0.194817861678474	CCDC42	146849	coiled-coil domain containing 42 [Source:HGNC Symbol;Acc:HGNC:26528]	
0.182408262692592	0.105504982977391	UNC45B	146862	unc-45 homolog B (C. elegans) [Source:HGNC Symbol;Acc:HGNC:14304]	
0.179632895645343	0.0894352032738547	C1QTNF8	390664	C1q and tumor necrosis factor related protein 8 [Source:HGNC Symbol;Acc:HGNC:31374]	
0.179476396390435	0.130522766839936	AMBRA1	55626	autophagy/beclin-1 regulator 1 [Source:HGNC Symbol;Acc:HGNC:25990]	
0.17878261051544	0.140283617573828	EML4	27436	echinoderm microtubule associated protein like 4 [Source:HGNC Symbol;Acc:HGNC:1316]	
0.178613790344657	0.166591303287494	LAT2	7462	linker for activation of T cells family, member 2 [Source:HGNC Symbol;Acc:HGNC:12749]	
0.177882144784471	0.117190568206432	MUC16	94025	mucin 16, cell surface associated [Source:HGNC Symbol;Acc:HGNC:15582]	
0.177556073290991	0.065947369113906	MAST4	375449	microtubule associated serine/threonine kinase family member 4 [Source:HGNC Symbol;Acc:HGNC:19037]	
0.177543923501179	0.0700779027207566	NIPAL4	348938	NIPA-like domain containing 4 [Source:HGNC Symbol;Acc:HGNC:28018]	
0.176664813503701	0.162274884504131	MMP20	9313	matrix metallopeptidase 20 [Source:HGNC Symbol;Acc:HGNC:7167]	
0.176246998792261	0.169339059398864	KRT7	3855	keratin 7 [Source:HGNC Symbol;Acc:HGNC:6445]	
0.174963917669061	0.192300637207539	OLIG3	167826	oligodendrocyte transcription factor 3 [Source:HGNC Symbol;Acc:HGNC:18003]	
0.174767448917551	0.165742622082911	RAE1	8480	ribonucleic acid export 1 [Source:HGNC Symbol;Acc:HGNC:9828]	
0.173611601860131	0.178804917574035	ARID3A	1820	AT rich interactive domain 3A (BRIGHT-like) [Source:HGNC Symbol;Acc:HGNC:3031]	
0.172148409208776	0.126675177853102	mars-10	162333	membrane-associated ring finger (C3HC4) 10, E3 ubiquitin protein ligase [Source:HGNC Symbol;Acc:HGNC:26655]	
0.171914203769656	0.165314992744263	FAM110B	90362	family with sequence similarity 110, member B [Source:HGNC Symbol;Acc:HGNC:28587]	
0.171129565806393	0.139841465719635	DNAH17	8632	dynein, axonemal, heavy chain 17 [Source:HGNC Symbol;Acc:HGNC:2946]	
0.170712816430589	0.112876729392539	TMIGD2	126259	transmembrane and immunoglobulin domain containing 2 [Source:HGNC Symbol;Acc:HGNC:28324]	
0.169952491672037	0.166152117395352	DNER	92737	delta/notch-like EGF repeat containing [Source:HGNC Symbol;Acc:HGNC:24456]	
0.169553854041165	0.189096675480271	UPB1	51733	ureidopropionase, beta [Source:HGNC Symbol;Acc:HGNC:16297]	
0.169514296037253	0.0409553218968005	CRTC2	200186	CREB regulated transcription coactivator 2 [Source:HGNC Symbol;Acc:HGNC:27301]	
0.169402349324012	0.196560870710515	CXCR1	3577	chemokine (C-X-C motif) receptor 1 [Source:HGNC Symbol;Acc:HGNC:6026]	
0.169091301324031	0.0731328637409228	ITIH1	3697	inter-alpha-trypsin inhibitor heavy chain 1 [Source:HGNC Symbol;Acc:HGNC:6166]	
0.168424525729625	0.192182918074364	ATP1A3	478	ATPase, Na+/K+ transporting, alpha 3 polypeptide [Source:HGNC Symbol;Acc:HGNC:801]	
0.167449579648559	0.169962038337487	UBE2E1	7324	ubiquitin-conjugating enzyme E2E 1 [Source:HGNC Symbol;Acc:HGNC:12477]	
0.165506107810111	0.117219354553032	FAM173B	134145	family with sequence similarity 173, member B [Source:HGNC Symbol;Acc:HGNC:27029]	
0.162829188517509	0.146309161063008	COL16A1	1307	collagen, type XVI, alpha 1 [Source:HGNC Symbol;Acc:HGNC:2193]	
0.160702158223623	0.151147680835084		3975	LIM/homeobox protein Lhx1 [Source:UniProtKB/Swiss-Prot;Acc:P48742]	
0.160643773059557	0.129463199460591	ACD	65057	adrenocortical dysplasia homolog (mouse) [Source:HGNC Symbol;Acc:HGNC:25070]	
0.152495268828293	0.196640879353636	TCTEX1D1	200132	Tctex1 domain containing 1 [Source:HGNC Symbol;Acc:HGNC:26882]	
0.149488356188701	0.188350202473358	SUZ12	23512	SUZ12 polycomb repressive complex 2 subunit [Source:HGNC Symbol;Acc:HGNC:17101]	
0.149301422872983	0.0907849646784246	FBXO24	26261	F-box protein 24 [Source:HGNC Symbol;Acc:HGNC:13595]	
0.149124012517639	0.11656311957014	C10orf67	256815	chromosome 10 open reading frame 67 [Source:HGNC Symbol;Acc:HGNC:28716]	
0.147397812014986	0.0754332521658681	TOX4	9878	TOX high mobility group box family member 4 [Source:HGNC Symbol;Acc:HGNC:20161]	
0.145195532052407	0.144985098927113	L2HGDH	79944	L-2-hydroxyglutarate dehydrogenase [Source:HGNC Symbol;Acc:HGNC:20499]	
0.143879281342941	0.172061031749973	KIF25	3834	kinesin family member 25 [Source:HGNC Symbol;Acc:HGNC:6390]	
0.143455754361639	0.195039576366716	MTO1	25821	mitochondrial tRNA translation optimization 1 [Source:HGNC Symbol;Acc:HGNC:19261]	
0.137353054171731	0.068870886428269	GON4L	54856	gon-4-like (C. elegans) [Source:HGNC Symbol;Acc:HGNC:25973]	
0.135143775948884	0.178564176902184	NUDT10	170685	nudix (nucleoside diphosphate linked moiety X)-type motif 10 [Source:HGNC Symbol;Acc:HGNC:17621]	
0.128729346206492	0.177183234694807	PSPC1	55269	paraspeckle component 1 [Source:HGNC Symbol;Acc:HGNC:20320]	
0.123809010123901	0.173828429066185	RPA1	6117	replication protein A1, 70kDa [Source:HGNC Symbol;Acc:HGNC:10289]	
0.122007922392805	0.188285725090978	ZNF556	80032	zinc finger protein 556 [Source:HGNC Symbol;Acc:HGNC:25669]	
0.117786787959619	0.176222793688482	NUP85	79902	nucleoporin 85kDa [Source:HGNC Symbol;Acc:HGNC:8734]	
0.116316726605302	0.170760448144587	PAGE5	90737	P antigen family, member 5 (prostate associated) [Source:HGNC Symbol;Acc:HGNC:29992]	
0.0732684110424416	0.156660345972173	SYNC	81493	syncoilin, intermediate filament protein [Source:HGNC Symbol;Acc:HGNC:28897]	
0.0548106691423786	0.181423141870834	ZNF826P	664701	zinc finger protein 826, pseudogene [Source:HGNC Symbol;Acc:HGNC:33875]	
-2.04989414496716	0.0101514859043371	SLC37A2	219855	solute carrier family 37 (glucose-6-phosphate transporter), member 2 [Source:HGNC Symbol;Acc:HGNC:20644]	
-2.0166414131108	0.152467615644457	HOXD10	3236	homeobox D10 [Source:HGNC Symbol;Acc:HGNC:5133]	
-1.47165936107728	0.0735097805602636	HOXD13	3239	homeobox D13 [Source:HGNC Symbol;Acc:HGNC:5136]	
-1.45999846217456	0.188285013833262	ACSM2B	348158	acyl-CoA synthetase medium-chain family member 2B [Source:HGNC Symbol;Acc:HGNC:30931]	
-1.43237609000516	0.115705401753194	HOXD11	3237	homeobox D11 [Source:HGNC Symbol;Acc:HGNC:5134]	
-1.25824442774891	0.0398764084956715	LGALS9C	654346	lectin, galactoside-binding, soluble, 9C [Source:HGNC Symbol;Acc:HGNC:33874]	
-1.11766534469777	0.0806822892837456	FNDC3B	101928615	fibronectin type III domain containing 3B [Source:HGNC Symbol;Acc:HGNC:24670]	
-1.11425906918731	0.144597158130961	VNN1	8876	vanin 1 [Source:HGNC Symbol;Acc:HGNC:12705]	
-1.09168368774635	0.151753519570108	GUSBP2	387036	glucuronidase, beta pseudogene 2 [Source:HGNC Symbol;Acc:HGNC:18792]	
-1.05722151494951	0.189388015084787	CPB1	1360	carboxypeptidase B1 (tissue) [Source:HGNC Symbol;Acc:HGNC:2299]	
-1.01268021645462	0.0335486579328403		5382		
-0.968889463624247	0.128934654004985	ANKRD30BL	554226	ankyrin repeat domain 30B-like [Source:HGNC Symbol;Acc:HGNC:35167]	
-0.936628313518914	0.184096582167571	BAMBI	25805	BMP and activin membrane-bound inhibitor [Source:HGNC Symbol;Acc:HGNC:30251]	
-0.923184551237131	0.192337423093315	UGT2B17	7367	UDP glucuronosyltransferase 2 family, polypeptide B17 [Source:HGNC Symbol;Acc:HGNC:12547]	

-0.892129794472425	0.164432175390416		28434	
-0.891828248281125	0.0538031585898249	VN1R4	317703	vomeronasal 1 receptor 4 [Source:HGNC Symbol;Acc:HGNC:19871]
-0.878196790204917	0.125524376321243	UGT2B10	7365	UDP glucuronosyltransferase 2 family, polypeptide B10 [Source:HGNC Symbol;Acc:HGNC:12544]
-0.864326032171526	0.0274349988404197	FTH1	2495	ferritin, heavy polypeptide 1 [Source:HGNC Symbol;Acc:HGNC:3976]
-0.859155245556118	0.0431904603865137	GLRA2	2742	glycine receptor, alpha 2 [Source:HGNC Symbol;Acc:HGNC:4327]
-0.858939500040156	0.00628493196924249	ATP6V0D2	245972	ATPase, H+ transporting, lysosomal 38kDa, V0 subunit d2 [Source:HGNC Symbol;Acc:HGNC:18266]
-0.842042240912688	0.00905046299958003	KIF5C	3800	kinesin family member 5C [Source:HGNC Symbol;Acc:HGNC:6325]
-0.813782401095364	0.19060563656686	HOXD12	3238	homeobox D12 [Source:HGNC Symbol;Acc:HGNC:5135]
-0.812033760105975	0.000135122345924328	FAM115A	9747	family with sequence similarity 115, member A [Source:HGNC Symbol;Acc:HGNC:22201]
-0.809248009113692	0.00301760383643024	SI	6476	sucrase-isomaltase (alpha-glucosidase) [Source:HGNC Symbol;Acc:HGNC:10856]
-0.805834028477633	0.00373228290108934	LPHN2	23266	latrophilin 2 [Source:HGNC Symbol;Acc:HGNC:18582]
-0.804666024670428	0.0357644237010441	PCK1	5105	phosphoenolpyruvate carboxykinase 1 (soluble) [Source:HGNC Symbol;Acc:HGNC:8724]
-0.800005968883069	0.179710693728062		6362	C-C motif chemokine 18 CCL18(1-68) CCL18(3-69) CCL18(4-69) [Source:UniProtKB/Swiss-Prot;Acc:P55774]
-0.794439184473272	0.0599474118467616	ADH1B	125	alcohol dehydrogenase 1B (class I), beta polypeptide [Source:HGNC Symbol;Acc:HGNC:250]
-0.792239745955018	0.197440824769985	GDF15	9518	growth differentiation factor 15 [Source:HGNC Symbol;Acc:HGNC:30142]
-0.791534933504977	0.0423306450370904	BZW1	9689	basic leucine zipper and W2 domains 1 [Source:HGNC Symbol;Acc:HGNC:18380]
-0.783364061969604	0.0577953858214633	USP2	9099	ubiquitin specific peptidase 2 [Source:HGNC Symbol;Acc:HGNC:12618]
-0.777171456515271	0.0595361651439089	SMIM24	284422	small integral membrane protein 24 [Source:HGNC Symbol;Acc:HGNC:37244]
-0.768124312315247	0.0515975925935305	PLLP	51090	plasmolipin [Source:HGNC Symbol;Acc:HGNC:18553]
-0.765303524792951	0.127516025109201	RASSF10	644943	Ras association (RalGDS/AF-6) domain family (N-terminal) member 10 [Source:HGNC Symbol;Acc:HGNC:33984]
-0.758354138347562	0.16505762241537	CYP2A6	1548	cytochrome P450, family 2, subfamily A, polypeptide 6 [Source:HGNC Symbol;Acc:HGNC:2610]
-0.750041003135499	0.0364765150734957	CT45A5	102723631	cancer/testis antigen family 45, member A5 [Source:HGNC Symbol;Acc:HGNC:33270]
-0.746993062419981	7.84853983339449e-08	RGS5	8490	regulator of G-protein signaling 5 [Source:HGNC Symbol;Acc:HGNC:10001]
-0.744564933981262	0.0283748023212031	RBM24	221662	RNA binding motif protein 24 [Source:HGNC Symbol;Acc:HGNC:21539]
-0.743599656442643	0.0435660843084978	GLDN	342035	gliomedin [Source:HGNC Symbol;Acc:HGNC:29514]
-0.735708952068022	0.189254109431191	CNTNAP3B	79937	contactin associated protein-like 3B [Source:HGNC Symbol;Acc:HGNC:32035]
-0.733407875040836	0.00941327843917845	CD14	929	CD14 molecule [Source:HGNC Symbol;Acc:HGNC:1628]
-0.731266061370893	0.160577525392871		4671	NAIP protein [Source:UniProtKB/TrEMBL;Acc:B7ZLE8]
-0.716970551048234	0.123882995666952	ABCG2	9429	ATP-binding cassette, sub-family G (WHITE), member 2 [Source:HGNC Symbol;Acc:HGNC:74]
-0.712477510507239	0.00485323167444782	BMP5	653	bone morphogenetic protein 5 [Source:HGNC Symbol;Acc:HGNC:1072]
-0.709873063980207	0.0532769236307275	HLA-DRB6	3128	major histocompatibility complex, class II, DR beta 6 (pseudogene) [Source:HGNC Symbol;Acc:HGNC:4954]
-0.701142354601146	0.0261741311856135	FRAS1	80144	Fraser extracellular matrix complex subunit 1 [Source:HGNC Symbol;Acc:HGNC:19185]
-0.697417808268847	0.000463517966757969	GSTM5	2949	glutathione S-transferase mu 5 [Source:HGNC Symbol;Acc:HGNC:4637]
-0.694448921463646	0.00794138679670582	SDHC	6391	succinate dehydrogenase complex, subunit C, integral membrane protein, 15kDa [Source:HGNC Symbol;Acc:HGNC:10682]
-0.692156365625923	0.00306717708197096	PREX2	80243	phosphatidylinositol-3,4,5-trisphosphate-dependent Rac exchange factor 2 [Source:HGNC Symbol;Acc:HGNC:22950]
-0.690784353010656	0.0392438200541657	SLC26A2	1836	solute carrier family 26 (anion exchanger), member 2 [Source:HGNC Symbol;Acc:HGNC:10994]
-0.674135737069134	0.0288290150150621	POSTN	10631	periostin, osteoblast specific factor [Source:HGNC Symbol;Acc:HGNC:16953]
-0.668632720376007	0.0329479221842146	PDK2	5164	pyruvate dehydrogenase kinase, isozyme 2 [Source:HGNC Symbol;Acc:HGNC:8810]
-0.664625553575442	0.068930656807129	ST6GAL2	84620	ST6 beta-galactosamide alpha-2,6-sialyltranferase 2 [Source:HGNC Symbol;Acc:HGNC:10861]
-0.663548714603202	0.022896291234878	B3GNT2	10678	UDP-GlcNAc:betaGal beta-1,3-N-acetylglucosaminyltransferase 2 [Source:HGNC Symbol;Acc:HGNC:15629]
-0.661894155475548	0.0610247816035567	MOXD1	26002	monooxygenase, DBH-like 1 [Source:HGNC Symbol;Acc:HGNC:21063]
-0.659483585219464	0.00116456236003057	RAB3B	5865	RAB3B, member RAS oncogene family [Source:HGNC Symbol;Acc:HGNC:9778]
-0.657756530128276	0.0104470134673421	ELTD1	64123	EGF, latrophilin and seven transmembrane domain containing 1 [Source:HGNC Symbol;Acc:HGNC:20822]
-0.657180210284482	0.0290035339278606	KLK15	55554	kallikrein-related peptidase 15 [Source:HGNC Symbol;Acc:HGNC:20453]
-0.653285552479113	0.05635127570172	C21orf88	114041	chromosome 21 open reading frame 88 [Source:HGNC Symbol;Acc:HGNC:16424]
-0.651671266561359	0.0331578742950751	TUBAL3	79861	tubulin, alpha-like 3 [Source:HGNC Symbol;Acc:HGNC:23534]
-0.65107370857305	0.0913680214013968		3507	
-0.649269740435293	0.00325117236164815	LRRC2-AS1	83598	LRRC2 antisense RNA 1 [Source:HGNC Symbol;Acc:HGNC:15571]
-0.648994755126279	0.0460082175235988	TRPM6	140803	transient receptor potential cation channel, subfamily M, member 6 [Source:HGNC Symbol;Acc:HGNC:17995]
-0.646759318357114	0.000836014123792557	SPTSSB	165679	serine palmitoyltransferase, small subunit B [Source:HGNC Symbol;Acc:HGNC:24045]
-0.643338794190774	0.111879619591987		28825	
-0.641480555782332	0.026483425448349	CALCRL	10203	calcitonin receptor-like [Source:HGNC Symbol;Acc:HGNC:16709]
-0.640433320741022	0.0313094023271441	APOD	347	apolipoprotein D [Source:HGNC Symbol;Acc:HGNC:612]
-0.638198689676667	0.156209192236016	ABCD2	225	ATP-binding cassette, sub-family D (ALD), member 2 [Source:HGNC Symbol;Acc:HGNC:66]
-0.634966343602539	0.0579675105087505	AKR1C3	8644	aldo-keto reductase family 1, member C3 [Source:HGNC Symbol;Acc:HGNC:386]
-0.6344613281493	0.0249959315467049	SLC2A10	81031	solute carrier family 2 (facilitated glucose transporter), member 10 [Source:HGNC Symbol;Acc:HGNC:13444]
-0.629272924782843	0.0233767269251032	EPHX1	2052	epoxide hydrolase 1, microsomal (xenobiotic) [Source:HGNC Symbol;Acc:HGNC:3401]
-0.625609140598329	0.0373225107659778	GLP2R	9340	glucagon-like peptide 2 receptor [Source:HGNC Symbol;Acc:HGNC:4325]
-0.625417506257388	0.0854273515299368	GPNMB	10457	glycoprotein (transmembrane) nmb [Source:HGNC Symbol;Acc:HGNC:4462]
-0.624715499290184	0.00223571995642877	PSMD4	5710	proteasome (prosome, macropain) 26S subunit, non-ATPase, 4 [Source:HGNC Symbol;Acc:HGNC:9561]
-0.620257082294241	0.0162010331701727	FMN2	56776	formin 2 [Source:HGNC Symbol;Acc:HGNC:14074]
-0.619056522938879	0.00814090982037454	HOXB13	10481	homeobox B13 [Source:HGNC Symbol;Acc:HGNC:5112]
-0.6186831906154	0.14904909103315	TMEM37	140738	transmembrane protein 37 [Source:HGNC Symbol;Acc:HGNC:18216]
-0.616292214445586	0.152879504600633		28526	
-0.615194195581237	0.0586317034955019	ISX	91464	intestine-specific homeobox [Source:HGNC Symbol;Acc:HGNC:28084]
-0.613542428239636	0.0315019759517828	METTL24	728464	methyltransferase like 24 [Source:HGNC Symbol;Acc:HGNC:21566]
-0.612505202356526	0.00349455445931508	PDGFD	80310	platelet derived growth factor D [Source:HGNC Symbol;Acc:HGNC:30620]
-0.612313376704519	0.0297098874705463	TCP11L2	255394	t-complex 11, testis-specific-like 2 [Source:HGNC Symbol;Acc:HGNC:28627]
-0.610422993204168	0.0427282524186905	ATG4A	115201	autophagy related 4A, cysteine peptidase [Source:HGNC Symbol;Acc:HGNC:16489]
-0.6097771080939	0.00327970497347121	PDGFRA	5156	platelet-derived growth factor receptor, alpha polypeptide [Source:HGNC Symbol;Acc:HGNC:8803]
-0.605780821337409	0.0267289681972064	PPM1L	151742	protein phosphatase, Mg2+/Mn2+ dependent, 1L [Source:HGNC Symbol;Acc:HGNC:16381]
-0.602518071567235	0.165226995604715	REP15	387849	RAB15 effector protein [Source:HGNC Symbol;Acc:HGNC:33748]
-0.601325893289982	0.00328864273847373	BAG2	9532	BCL2-associated athanogene 2 [Source:HGNC Symbol;Acc:HGNC:938]
-0.6011693107502	0.0133336731533752	ZNF304	57343	zinc finger protein 304 [Source:HGNC Symbol;Acc:HGNC:13505]
-0.599481947447307	0.00162335855166972	KIAA1715	80856	KIAA1715 [Source:HGNC Symbol;Acc:HGNC:21610]
-0.598398961391614	0.0131446034568802	XYLT2	64132	xylosyltransferase II [Source:HGNC Symbol;Acc:HGNC:15517]
-0.596478469996079	0.105579531874628	EPHA7	2045	EPH receptor A7 [Source:HGNC Symbol;Acc:HGNC:3390]
-0.595619589149827	0.0328310550341708	PLVAP	83483	plasmalemma vesicle associated protein [Source:HGNC Symbol;Acc:HGNC:13635]
-0.595439304996702	0.0288559091115028	CAV1	857	caveolin 1, caveolae protein, 22kDa [Source:HGNC Symbol;Acc:HGNC:1527]
-0.594619060980754	0.0288020759850319	ENPP1	5167	ectonucleotide pyrophosphatase/phosphodiesterase 1 [Source:HGNC Symbol;Acc:HGNC:3356]
-0.594296456448138	0.0718726862605801	ANKRD50	57182	ankyrin repeat domain 50 [Source:HGNC Symbol;Acc:HGNC:29223]
-0.590605616483658	0.0585806655800276	TNNC1	7134	troponin C type 1 (slow) [Source:HGNC Symbol;Acc:HGNC:11943]
-0.587544984139792	0.0630091962124897	AOC3	8639	amine oxidase, copper containing 3 [Source:HGNC Symbol;Acc:HGNC:550]
-0.587204886321916	0.132020763594197	B3GNT7	93010	UDP-GlcNAc:betaGal beta-1,3-N-acetylglucosaminyltransferase 7 [Source:HGNC Symbol;Acc:HGNC:18811]
-0.582915558495335	0.0682488976315045	RFTN2	130132	raftlin family member 2 [Source:HGNC Symbol;Acc:HGNC:26402]
-0.578593613571247	0.0552236187457688	GFRA2	2675	GDNF family receptor alpha 2 [Source:HGNC Symbol;Acc:HGNC:4244]
-0.577197553434593	0.131047659743514		100505549	
-0.577100282036326	0.0450207528362334	ALDH1A1	216	aldehyde dehydrogenase 1 family, member A1 [Source:HGNC Symbol;Acc:HGNC:402]
-0.575253015435267	2.42970563900903e-05	PPP1R9A	55607	protein phosphatase 1, regulatory subunit 9A [Source:HGNC Symbol;Acc:HGNC:14946]
-0.574431031185927	0.137027060943575	SLITRK6	84189	SLIT and NTRK-like family, member 6 [Source:HGNC Symbol;Acc:HGNC:23503]
-0.573634453514599	0.000915960381019784	PRUNE	58497	prune exopolyphosphatase [Source:HGNC Symbol;Acc:HGNC:13420]
-0.5731327688781	0.0322749526694045	RBM8A	9939	RNA binding motif protein 8A [Source:HGNC Symbol;Acc:HGNC:9905]
-0.572651042662969	0.122518637432972	PRDX6	9588	peroxiredoxin 6 [Source:HGNC Symbol;Acc:HGNC:16753]
-0.571218677495449	0.0786108762365682	NQO1	1728	NAD(P)H dehydrogenase, quinone 1 [Source:HGNC Symbol;Acc:HGNC:2874]
-0.568791774718338	0.115048669429693	LIFR	3977	leukemia inhibitory factor receptor alpha [Source:HGNC Symbol;Acc:HGNC:6597]
-0.568298129910472	0.00969959959440969	FOXN3	1112	forkhead box N3 [Source:HGNC Symbol;Acc:HGNC:1928]
-0.56772855345384	0.00586645060908531	FERMT2	10979	fermitin family member 2 [Source:HGNC Symbol;Acc:HGNC:15767]
-0.566827766639491	0.059773707120362	C1QTNF3	114899	C1q and tumor necrosis factor related protein 3 [Source:HGNC Symbol;Acc:HGNC:14326]
-0.566671584926474	0.063441555629331	CPA3	1359	carboxypeptidase A3 (mast cell) [Source:HGNC Symbol;Acc:HGNC:2298]
-0.56635566812841	0.121214498782282	FAM171B	165215	family with sequence similarity 171, member B [Source:HGNC Symbol;Acc:HGNC:29412]
-0.565986471948152	0.102309242010867	ATP2B1	490	ATPase, Ca++ transporting, plasma membrane 1 [Source:HGNC Symbol;Acc:HGNC:814]
-0.565737152814157	0.000420200416749848	VAT1	10493	vesicle amine transport 1 [Source:HGNC Symbol;Acc:HGNC:16919]
-0.56332957284809	0.0580746994012735	HEXIM1	10614	hexamethylene bis-acetamide inducible 1 [Source:HGNC Symbol;Acc:HGNC:24953]
-0.561885765890433	0.0877386541958771	BEST2	54831	bestrophin 2 [Source:HGNC Symbol;Acc:HGNC:17107]
-0.561651570220965	0.161055223176171	STON1	11037	stonin 1 [Source:HGNC Symbol;Acc:HGNC:17003]
-0.561387353120892	0.0537831422801687	CHGA	1113	chromogranin A (parathyroid secretory protein 1) [Source:HGNC Symbol;Acc:HGNC:1929]
-0.5579178865742	0.00134923181533582	SLC25A20	788	solute carrier family 25 (carnitine/acylcarnitine translocase), member 20 [Source:HGNC Symbol;Acc:HGNC:1421]
-0.553898724240031	0.0421993765323631	FCGR3A	2214	Fc fragment of IgG, low affinity IIIa, receptor (CD16a) [Source:HGNC Symbol;Acc:HGNC:3619]
-0.551845252508405	0.0804954044609621	MFSD5	84975	major facilitator superfamily domain containing 5 [Source:HGNC Symbol;Acc:HGNC:28156]
-0.551679212544964	0.147270430016527	NT5E	4907	5'-nucleotidase, ecto (CD73) [Source:HGNC Symbol;Acc:HGNC:8021]
-0.551628527314753	0.11227564676179	TMEM70	54968	transmembrane protein 70 [Source:HGNC Symbol;Acc:HGNC:26050]
-0.550660562636785	0.0679505205097359	ATP5H	10476	ATP synthase, H+ transporting, mitochondrial Fo complex, subunit d [Source:HGNC Symbol;Acc:HGNC:845]
-0.550333333728759	7.98526506290896e-05	OLFML1	283298	olfactomedin-like 1 [Source:HGNC Symbol;Acc:HGNC:24473]
-0.549009424780583	0.0028448130233342	ZEB2	9839	zinc finger E-box binding homeobox 2 [Source:HGNC Symbol;Acc:HGNC:14881]
-0.548446304576485	0.0416934912885659	LRRC19	64922	leucine rich repeat containing 19 [Source:HGNC Symbol;Acc:HGNC:23379]
-0.548432677575631	0.0406661971536572	ERBB2	2064	v-erb-b2 avian erythroblastic leukemia viral oncogene homolog 2 [Source:HGNC Symbol;Acc:HGNC:3430]
-0.547047612939864	0.0437157880480044	C6orf211	79624	chromosome 6 open reading frame 211 [Source:HGNC Symbol;Acc:HGNC:17872]
-0.546609052132084	0.065081333561101	FAM198B	51313	family with sequence similarity 198, member B [Source:HGNC Symbol;Acc:HGNC:25312]
-0.546462902062953	0.00927482428811453	DYNLT3	6990	dynein, light chain, Tctex-type 3 [Source:HGNC Symbol;Acc:HGNC:11694]
-0.543434287227363	0.0231125567965173	SLC30A4	7782	solute carrier family 30 (zinc transporter), member 4 [Source:HGNC Symbol;Acc:HGNC:11015]
-0.542261555046906	0.082760031497814	CHL1	10752	cell adhesion molecule L1-like [Source:HGNC Symbol;Acc:HGNC:1939]
-0.541851616042935	0.167796614844507	CYP27A1	1593	cytochrome P450, family 27, subfamily A, polypeptide 1 [Source:HGNC Symbol;Acc:HGNC:2605]
-0.540983728197169	0.0010090096172064	PSD3	23362	pleckstrin and Sec7 domain containing 3 [Source:HGNC Symbol;Acc:HGNC:19093]
-0.540805187246628	0.0766890127186545	CHGB	1114	chromogranin B (secretogranin 1) [Source:HGNC Symbol;Acc:HGNC:1930]
-0.540240234236614	0.172499413540008	C3AR1	719	complement component 3a receptor 1 [Source:HGNC Symbol;Acc:HGNC:1319]
-0.539826485458499	0.144504994476365	TBC1D19	55296	TBC1 domain family, member 19 [Source:HGNC Symbol;Acc:HGNC:25624]
-0.536567517174062	0.123164873098007	LDHD	197257	lactate dehydrogenase D [Source:HGNC Symbol;Acc:HGNC:19708]
-0.536253210666822	0.0978964616187942	SCNN1B	6338	sodium channel, non-voltage-gated 1, beta subunit [Source:HGNC Symbol;Acc:HGNC:10600]
-0.535177988218732	0.00533039136461682	ACSF2	80221	acyl-CoA synthetase family member 2 [Source:HGNC Symbol;Acc:HGNC:26101]
-0.534250056960805	0.00232185413656258	METTL16	79066	methyltransferase like 16 [Source:HGNC Symbol;Acc:HGNC:28484]
-0.533961731271919	0.0863519385408842	FRZB	2487	frizzled-related protein [Source:HGNC Symbol;Acc:HGNC:3959]
-0.533544086427444	0.00508300168563596	IFT57	55081	intraflagellar transport 57 [Source:HGNC Symbol;Acc:HGNC:17367]
-0.530256297320622	0.113937854508397	HERC6	55008	HECT and RLD domain containing E3 ubiquitin protein ligase family member 6 [Source:HGNC Symbol;Acc:HGNC:26072]
-0.528607803665999	0.00950501056400429	ZFYVE9	9372	zinc finger, FYVE domain containing 9 [Source:HGNC Symbol;Acc:HGNC:6775]
-0.525660972573235	0.0824725059775989	PTPRB	5787	protein tyrosine phosphatase, receptor type, B [Source:HGNC Symbol;Acc:HGNC:9665]
-0.521416790539498	0.0210950791051024	PLBD1	79887	phospholipase B domain containing 1 [Source:HGNC Symbol;Acc:HGNC:26215]
-0.519760512176971	0.00750910179368186	FAM114A1	92689	family with sequence similarity 114, member A1 [Source:HGNC Symbol;Acc:HGNC:25087]
-0.518396559239474	0.0502284680912413	CLDN8	9073	claudin 8 [Source:HGNC Symbol;Acc:HGNC:2050]
-0.518249209932708	0.106901532939455	HSPB8	26353	heat shock 22kDa protein 8 [Source:HGNC Symbol;Acc:HGNC:30171]
-0.514436626978335	0.0385199162562263	KATNAL1	84056	katanin p60 subunit A-like 1 [Source:HGNC Symbol;Acc:HGNC:28361]
-0.513171729145732	0.0675668618061513	OAT	4942	ornithine aminotransferase [Source:HGNC Symbol;Acc:HGNC:8091]

-0.512108531703074	0.162840088914808	ZDHHC1	29800	zinc finger, DHHC-type containing 1 [Source:HGNC Symbol;Acc:HGNC:17916]
-0.511387037525747	0.0456846921818288	RIIAD1	284485	regulatory subunit of type II PKA R-subunit (RIIa) domain containing 1 [Source:HGNC Symbol;Acc:HGNC:26686]
-0.511259154560599	0.0485620412510411	DSTNP2	171220	destrin (actin depolymerizing factor) pseudogene 2 [Source:HGNC Symbol;Acc:HGNC:34546]
-0.510994363449434	0.0446882860034688	CNNM2	54805	cyclin and CBS domain divalent metal cation transport mediator 2 [Source:HGNC Symbol;Acc:HGNC:103]
-0.510808727956687	0.03393510064633	GCSAML	148823	germinal center-associated, signaling and motility-like [Source:HGNC Symbol;Acc:HGNC:29583]
-0.509623055656254	0.0128881450590178	GXYLT1	283464	glucoside xylosyltransferase 1 [Source:HGNC Symbol;Acc:HGNC:27482]
-0.509573683401218	0.0107101852994102	PROS1	5627	protein S (alpha) [Source:HGNC Symbol;Acc:HGNC:9456]
-0.508940556379818	0.0713326659722401	KRCC1	51315	lysine-rich coiled-coil 1 [Source:HGNC Symbol;Acc:HGNC:28039]
-0.508689782307265	0.0955632070861034	EFNA3	1944	ephrin-A3 [Source:HGNC Symbol;Acc:HGNC:3223]
-0.508524814012327	0.00668883274329975	IGFBP3	3486	insulin-like growth factor binding protein 3 [Source:HGNC Symbol;Acc:HGNC:5472]
-0.505914703230512	6.76589296718252e-06	GDPD1	284161	glycerophosphodiester phosphodiesterase domain containing 1 [Source:HGNC Symbol;Acc:HGNC:20883]
-0.505076953695252	0.179824724965325	TSPAN6	7105	tetraspanin 6 [Source:HGNC Symbol;Acc:HGNC:11858]
-0.504781108116083	0.0211102450551076	JUN	3725	jun proto-oncogene [Source:HGNC Symbol;Acc:HGNC:6204]
-0.504723354924272	0.136012008455147	SYNPO2	171024	synaptopodin 2 [Source:HGNC Symbol;Acc:HGNC:17732]
-0.504577132631553	0.0888167982169331	DOCK11	139818	dedicator of cytokinesis 11 [Source:HGNC Symbol;Acc:HGNC:23483]
-0.503546073943414	0.0848826489886844	KLHL5	51088	kelch-like family member 5 [Source:HGNC Symbol;Acc:HGNC:6356]
-0.503324222420289	0.00252242409912159	HINT2	84681	histidine triad nucleotide binding protein 2 [Source:HGNC Symbol;Acc:HGNC:18344]
-0.502697874103516	0.0361697244100406	CHST5	23563	carbohydrate (N-acetylglucosamine 6-O) sulfotransferase 5 [Source:HGNC Symbol;Acc:HGNC:1973]
-0.502651346203117	0.0885448844900609	DRAM1	55332	DNA-damage regulated autophagy modulator 1 [Source:HGNC Symbol;Acc:HGNC:25645]
-0.502025915481645	0.0467981697923335	MINPP1	9562	multiple inositol-polyphosphate phosphatase 1 [Source:HGNC Symbol;Acc:HGNC:7102]
-0.501870039487019	0.109038140926714	KANSL1L	151050	KAT8 regulatory NSL complex subunit 1-like [Source:HGNC Symbol;Acc:HGNC:26310]
-0.498387378178263	0.00195090104546355	ARHGAP31	57514	Rho GTPase activating protein 31 [Source:HGNC Symbol;Acc:HGNC:29216]
-0.497403376238474	0.0328589929002896	CTSE	1510	cathepsin E [Source:HGNC Symbol;Acc:HGNC:2530]
-0.497142509653339	0.0189732471150312	SPARCL1	8404	SPARC-like 1 (hevin) [Source:HGNC Symbol;Acc:HGNC:11220]
-0.495870955381631	0.172556059630139		339742	
-0.495395597636772	0.186830693957118	ACER2	340485	alkaline ceramidase 2 [Source:HGNC Symbol;Acc:HGNC:23675]
-0.495103478766658	0.0153381021007532	TWSG1	57045	twisted gastrulation BMP signaling modulator 1 [Source:HGNC Symbol;Acc:HGNC:12429]
-0.492635064497041	0.0727869940126085	GAA	2548	glucosidase, alpha; acid [Source:HGNC Symbol;Acc:HGNC:4065]
-0.490207307276253	0.13170246570258	MMP2	4313	matrix metallopeptidase 2 (gelatinase A, 72kDa gelatinase, 72kDa type IV collagenase) [Source:HGNC Symbol;Acc:HGNC:7166]
-0.48962861893728	0.00315144801392857	HGD	3081	homogentisate 1,2-dioxygenase [Source:HGNC Symbol;Acc:HGNC:4892]
-0.48897083399284	0.054545960265482	RNF152	220441	ring finger protein 152 [Source:HGNC Symbol;Acc:HGNC:26811]
-0.488695346351764	0.0013792228495199	RMND5A	64795	required for meiotic nuclear division 5 homolog A (S. cerevisiae) [Source:HGNC Symbol;Acc:HGNC:25850]
-0.488528714403239	0.126981839276015	PXDN	7837	peroxidasin homolog (Drosophila) [Source:HGNC Symbol;Acc:HGNC:14966]
-0.487171819566936	0.0649707548272824	PCSK1	5122	proprotein convertase subtilisin/kexin type 1 [Source:HGNC Symbol;Acc:HGNC:8743]
-0.487073133644109	0.0299939482479182	HPCAL1	3241	hippocalcin-like 1 [Source:HGNC Symbol;Acc:HGNC:5145]
-0.483603035980871	0.0383387584395395	PDCD4	27250	programmed cell death 4 (neoplastic transformation inhibitor) [Source:HGNC Symbol;Acc:HGNC:8763]
-0.483114033140418	0.0230407787478953	KHDC1L	100129128	KH homology domain containing 1-like [Source:HGNC Symbol;Acc:HGNC:37274]
-0.482881673091176	0.130905852062835	SLIT2	9353	slit homolog 2 (Drosophila) [Source:HGNC Symbol;Acc:HGNC:11086]
-0.481606102991263	0.177309580797262		79686	
-0.481421817767334	0.198999038374875	RASGRP3	25780	RAS guanyl releasing protein 3 (calcium and DAG-regulated) [Source:HGNC Symbol;Acc:HGNC:14545]
-0.481040773292418	0.0960071640166728	SEMA3A	10371	sema domain, immunoglobulin domain (Ig), short basic domain, secreted, (semaphorin) 3A [Source:HGNC Symbol;Acc:HGNC:10723]
-0.479655671573319	0.036021094975207	ZNF704	619279	zinc finger protein 704 [Source:HGNC Symbol;Acc:HGNC:32291]
-0.478732483382117	0.00356846903927392	CAB39L	81617	calcium binding protein 39-like [Source:HGNC Symbol;Acc:HGNC:20290]
-0.477598343139256	0.0127203338115539	GLUD2	2747	glutamate dehydrogenase 2 [Source:HGNC Symbol;Acc:HGNC:4336]
-0.477060423412855	0.11400326134228	DDX60	55601	DEAD (Asp-Glu-Ala-Asp) box polypeptide 60 [Source:HGNC Symbol;Acc:HGNC:25942]
-0.476362870570442	0.115253861385763	SLC27A4	10999	solute carrier family 27 (fatty acid transporter), member 4 [Source:HGNC Symbol;Acc:HGNC:10998]
-0.474770724105266	0.00514993392091159	TSPAN4	7106	tetraspanin 4 [Source:HGNC Symbol;Acc:HGNC:11859]
-0.474548686718624	0.181638075396668	TFEC	22797	transcription factor EC [Source:HGNC Symbol;Acc:HGNC:11754]
-0.474480757556198	0.0115853092889897	TTLL7	79739	tubulin tyrosine ligase-like family, member 7 [Source:HGNC Symbol;Acc:HGNC:26242]
-0.474253740828294	0.018335543002112	LPHN3	23284	latrophilin 3 [Source:HGNC Symbol;Acc:HGNC:20974]
-0.473782302159771	0.000348255151928608	RNF11	26994	ring finger protein 11 [Source:HGNC Symbol;Acc:HGNC:10056]
-0.471439486451661	0.163115415590843	SST	6750	somatostatin [Source:HGNC Symbol;Acc:HGNC:11329]
-0.470739796715434	0.0472518853549866	ZNF217	7764	zinc finger protein 217 [Source:HGNC Symbol;Acc:HGNC:13009]
-0.470506304318912	0.0915279982983272	CD163	9332	CD163 molecule [Source:HGNC Symbol;Acc:HGNC:1631]
-0.470415535731884	0.113785472284246	PPP4R2	151987	protein phosphatase 4, regulatory subunit 2 [Source:HGNC Symbol;Acc:HGNC:18296]
-0.46993186516938	0.0224384038732449	PECAM1	5175	platelet/endothelial cell adhesion molecule 1 [Source:HGNC Symbol;Acc:HGNC:8823]
-0.469083356182632	0.101871608532	ZNF717	100131827	zinc finger protein 717 [Source:HGNC Symbol;Acc:HGNC:29448]
-0.468108048471414	0.0794062723094929	BMP4	652	bone morphogenetic protein 4 [Source:HGNC Symbol;Acc:HGNC:1071]
-0.467625944237723	0.106064447399018	RAB3D	9545	RAB3D, member RAS oncogene family [Source:HGNC Symbol;Acc:HGNC:9779]
-0.467080581717785	0.0519757553681656	ID2	3398	inhibitor of DNA binding 2, dominant negative helix-loop-helix protein [Source:HGNC Symbol;Acc:HGNC:5361]
-0.466895621065085	0.016452320504166	SLC38A7	55238	solute carrier family 38, member 7 [Source:HGNC Symbol;Acc:HGNC:25582]
-0.466269147841801	0.16550010983416	LRRC66	339977	leucine rich repeat containing 66 [Source:HGNC Symbol;Acc:HGNC:34299]
-0.466056162518824	0.0395550458230111	AMICA1	120425	adhesion molecule, interacts with CXADR antigen 1 [Source:HGNC Symbol;Acc:HGNC:19084]
-0.466054268260716	0.00164853028769635	MAATS1	89876	MYCBP-associated, testis expressed 1 [Source:HGNC Symbol;Acc:HGNC:24010]
-0.46283893322306	0.0157017527280698	SIK2	23235	salt-inducible kinase 2 [Source:HGNC Symbol;Acc:HGNC:21680]
-0.462834124317842	0.0442653657221949	SCRN1	9805	secernin 1 [Source:HGNC Symbol;Acc:HGNC:22192]
-0.462477050097321	0.0437667543182413	AMIGO2	347902	adhesion molecule with Ig-like domain 2 [Source:HGNC Symbol;Acc:HGNC:24073]
-0.4623721540726	0.0843575977132971	FLT1	2321	fms-related tyrosine kinase 1 [Source:HGNC Symbol;Acc:HGNC:3763]
-0.461365127816304	0.0766769478766587	WDR45B	56270	WD repeat domain 45B [Source:HGNC Symbol;Acc:HGNC:25072]
-0.46117175761572	0.147451332695032	DESI2	51029	desumoylating isopeptidase 2 [Source:HGNC Symbol;Acc:HGNC:24264]
-0.461019407171903	0.0246009760043559	PRLR	5618	prolactin receptor [Source:HGNC Symbol;Acc:HGNC:9446]
-0.45944093844392	0.0316088344860993	PLXNC1	10154	plexin C1 [Source:HGNC Symbol;Acc:HGNC:9106]
-0.458874258226856	0.00333831225537839	PAM	5066	peptidylglycine alpha-amidating monooxygenase [Source:HGNC Symbol;Acc:HGNC:8596]
-0.45862608878081	0.0784206791771132	PLAGL2	5326	pleiomorphic adenoma gene-like 2 [Source:HGNC Symbol;Acc:HGNC:9047]
-0.45770015214693	0.140598338613414	PLIN2	123	perilipin 2 [Source:HGNC Symbol;Acc:HGNC:248]
-0.457017619916821	0.022708434255938	TBPL1	9519	TBP-like 1 [Source:HGNC Symbol;Acc:HGNC:11589]
-0.456940022594829	0.0334583458538219	A2M	2	alpha-2-macroglobulin [Source:HGNC Symbol;Acc:HGNC:7]
-0.45591422747004	0.0498243773776207		414318	
-0.455664749397541	0.187766662122077	DTX2	113878	deltex 2, E3 ubiquitin ligase [Source:HGNC Symbol;Acc:HGNC:15973]
-0.455091228593739	0.198863494952692	PKDCC	91461	protein kinase domain containing, cytoplasmic [Source:HGNC Symbol;Acc:HGNC:25123]
-0.454844359138203	0.0965243355582371	PSMD2	5708	proteasome (prosome, macropain) 26S subunit, non-ATPase, 2 [Source:HGNC Symbol;Acc:HGNC:9559]
-0.453336060087051	0.183634685684159	ZNF85	7639	zinc finger protein 85 [Source:HGNC Symbol;Acc:HGNC:13160]
-0.453237652752218	0.0342866932547537	SAT1	6303	spermidine/spermine N1-acetyltransferase 1 [Source:HGNC Symbol;Acc:HGNC:10540]
-0.45298051063582	0.000946206059576375	SLC9A6	10479	solute carrier family 9, subfamily A (NHE6, cation proton antiporter 6), member 6 [Source:HGNC Symbol;Acc:HGNC:11079]
-0.452503375150262	0.0424366069681481	SOSTDC1	25928	sclerostin domain containing 1 [Source:HGNC Symbol;Acc:HGNC:21748]
-0.452427749269106	0.00280840961220443	RAB34	83871	RAB34, member RAS oncogene family [Source:HGNC Symbol;Acc:HGNC:16519]
-0.450920670252535	0.158859104371403	C2orf88	84281	chromosome 2 open reading frame 88 [Source:HGNC Symbol;Acc:HGNC:28191]
-0.450421656417749	0.112902028886037		100528019	
-0.449072976957564	0.0816515711290058	RALBP1	10928	ralA binding protein 1 [Source:HGNC Symbol;Acc:HGNC:9841]
-0.44812704370256	0.086173076691364	RNASE4	6038	ribonuclease, RNase A family, 4 [Source:HGNC Symbol;Acc:HGNC:10047]
-0.447648214283728	0.0626320627643005	LONP1	9361	lon peptidase 1, mitochondrial [Source:HGNC Symbol;Acc:HGNC:9479]
-0.447556069106041	0.00316715797348168	KIAA0100	9703	KIAA0100 [Source:HGNC Symbol;Acc:HGNC:28960]
-0.446702364082512	0.0277600737303251	DMXL1	1657	Dmx-like 1 [Source:HGNC Symbol;Acc:HGNC:2937]
-0.44668101936524	0.0994363077786381		101927770	
-0.445826156364967	0.195039970339039	ZNF429	353088	zinc finger protein 429 [Source:HGNC Symbol;Acc:HGNC:20817]
-0.445619868027605	0.0144979619287133	AGA	175	aspartylglucosaminidase [Source:HGNC Symbol;Acc:HGNC:318]
-0.444927445835197	0.065395173899918	PDE7B	27115	phosphodiesterase 7B [Source:HGNC Symbol;Acc:HGNC:8792]
-0.444883889697645	0.0545161943696052	CXorf40B	541578	chromosome X open reading frame 40B [Source:HGNC Symbol;Acc:HGNC:17402]
-0.44334860334626	0.0945258252969778	PHLDB2	90102	pleckstrin homology-like domain, family B, member 2 [Source:HGNC Symbol;Acc:HGNC:29573]
-0.443163176769828	0.174601577383585	CIB2	10518	calcium and integrin binding family member 2 [Source:HGNC Symbol;Acc:HGNC:24579]
-0.442128284003403	0.176646278433501	THY1	7070	Thy-1 cell surface antigen [Source:HGNC Symbol;Acc:HGNC:11801]
-0.441928713992553	0.143771548168524	ST3GAL2	6483	ST3 beta-galactoside alpha-2,3-sialyltransferase 2 [Source:HGNC Symbol;Acc:HGNC:10863]
-0.441527728440417	0.178915231487683	MXRA5	25878	matrix-remodelling associated 5 [Source:HGNC Symbol;Acc:HGNC:7539]
-0.441091489327538	0.0614201329546656	FOXO4	4303	forkhead box O4 [Source:HGNC Symbol;Acc:HGNC:7139]
-0.440390888352086	0.0258401411855387	ASB13	79754	ankyrin repeat and SOCS box containing 13 [Source:HGNC Symbol;Acc:HGNC:19765]
-0.43972150871748	0.170445712224876	GNA11	2767	guanine nucleotide binding protein (G protein), alpha 11 (Gq class) [Source:HGNC Symbol;Acc:HGNC:4379]
-0.439504898137087	0.015936378305324	RNF185	91445	ring finger protein 185 [Source:HGNC Symbol;Acc:HGNC:26783]
-0.4391751595327	0.0629257167546577	MATN2	4147	matrilin 2 [Source:HGNC Symbol;Acc:HGNC:6908]
-0.438727812683355	0.00718953415649891	PLS3	5358	plastin 3 [Source:HGNC Symbol;Acc:HGNC:9091]
-0.438251215265618	0.126721481380808	SSBP3	23648	single stranded DNA binding protein 3 [Source:HGNC Symbol;Acc:HGNC:15674]
-0.437387265851313	0.0105791522877729	PID1	55022	phosphotyrosine interaction domain containing 1 [Source:HGNC Symbol;Acc:HGNC:26084]
-0.436640916933204	0.072153818307243		728392	
-0.436394821493675	0.0563077238827634	CCR2	729230	chemokine (C-C motif) receptor 2 [Source:HGNC Symbol;Acc:HGNC:1603]
-0.434913003106435	0.0145345270901609	DCAF10	79269	DDB1 and CUL4 associated factor 10 [Source:HGNC Symbol;Acc:HGNC:23686]
-0.434144996379304	0.125226425317392	NUDC	10726	nudC nuclear distribution protein [Source:HGNC Symbol;Acc:HGNC:8045]
-0.433765409408404	0.0749279037408952	RND3	390	Rho family GTPase 3 [Source:HGNC Symbol;Acc:HGNC:671]
-0.433091301003659	0.045997767987418	TMEM63B	55362	transmembrane protein 63B [Source:HGNC Symbol;Acc:HGNC:17735]
-0.432904904419669	0.0356998370755887	ZBTB4	57659	zinc finger and BTB domain containing 4 [Source:HGNC Symbol;Acc:HGNC:23847]
-0.432752202505871	0.157259084062451	PTPRN2	5799	protein tyrosine phosphatase, receptor type, N polypeptide 2 [Source:HGNC Symbol;Acc:HGNC:9677]
-0.43230222691776	0.0251823494331463	HMGCS2	3158	3-hydroxy-3-methylglutaryl-CoA synthase 2 (mitochondrial) [Source:HGNC Symbol;Acc:HGNC:5008]
-0.431843976073846	0.0191058580456868	EMP3	2014	epithelial membrane protein 3 [Source:HGNC Symbol;Acc:HGNC:3335]
-0.431818998367334	0.123922419534552	MFSD9	84804	major facilitator superfamily domain containing 9 [Source:HGNC Symbol;Acc:HGNC:28158]
-0.431658433853381	0.0220074596393579	NEK11	79858	NIMA-related kinase 11 [Source:HGNC Symbol;Acc:HGNC:18593]
-0.431595224126339	0.181913040854442	CCNG1	900	cyclin G1 [Source:HGNC Symbol;Acc:HGNC:1592]
-0.431432184734698	0.133463552190168	NDNF	79625	neuron-derived neurotrophic factor [Source:HGNC Symbol;Acc:HGNC:26256]
-0.431415854883095	0.0682771227937802	NIPAL3	57185	NIPA-like domain containing 3 [Source:HGNC Symbol;Acc:HGNC:25233]
-0.431408899651838	0.175992572700414	EMCN	51705	endomucin [Source:HGNC Symbol;Acc:HGNC:16041]
-0.431260533807595	0.0995523993944211	A1CF	29974	APOBEC1 complementation factor [Source:HGNC Symbol;Acc:HGNC:24086]
-0.43025245802694	0.0615431479024775	VCAN	1462	versican [Source:HGNC Symbol;Acc:HGNC:2464]
-0.430069612290666	0.104325759790523	C1orf115	79762	chromosome 1 open reading frame 115 [Source:HGNC Symbol;Acc:HGNC:25873]
-0.429737814101125	0.119930433170821	SNAI2	6591	snail family zinc finger 2 [Source:HGNC Symbol;Acc:HGNC:11094]
-0.428380909131151	0.0211046604473765	CLIC2	1193	chloride intracellular channel 2 [Source:HGNC Symbol;Acc:HGNC:2063]
-0.427899739887684	0.103999894540448	FCGR2B	2213	Fc fragment of IgG, low affinity IIb, receptor (CD32) [Source:HGNC Symbol;Acc:HGNC:3618]
-0.426553005004911	0.0735456763667468	CAP2	10486	CAP, adenylate cyclase-associated protein, 2 (yeast) [Source:HGNC Symbol;Acc:HGNC:20039]
-0.426299248151856	0.0339625446747976	KIAA1161	57462	KIAA1161 [Source:HGNC Symbol;Acc:HGNC:19918]
-0.425993989472238	0.0729940927030874	FAT4	79633	FAT atypical cadherin 4 [Source:HGNC Symbol;Acc:HGNC:23109]
-0.424900224308204	0.11622357385896	ORMDL3	94103	ORMDL sphingolipid biosynthesis regulator 3 [Source:HGNC Symbol;Acc:HGNC:16038]
-0.424860403976575	0.0197184830969333	C4orf19	55286	chromosome 4 open reading frame 19 [Source:HGNC Symbol;Acc:HGNC:25618]
-0.424646770895728	0.0348562567434024	RCAN2	10231	regulator of calcineurin 2 [Source:HGNC Symbol;Acc:HGNC:3041]
-0.42437904553305	0.0320814466238604	DCTN4	51164	dynactin 4 (p62) [Source:HGNC Symbol;Acc:HGNC:15518]
-0.423693877461115	0.144788309445578	PCDH18	54510	protocadherin 18 [Source:HGNC Symbol;Acc:HGNC:14268]

-0.423644723300494	0.0208010798259518	ALKBH5	54890	AlkB family member 5, RNA demethylase [Source:HGNC Symbol;Acc:HGNC:25996]
-0.423590453825993	0.0501451740414623	CPT1A	1374	carnitine palmitoyltransferase 1A (liver) [Source:HGNC Symbol;Acc:HGNC:2328]
-0.422944688992448	0.0261090518652685	MCOLN1	57192	mucolipin 1 [Source:HGNC Symbol;Acc:HGNC:13356]
-0.421695004869763	0.00759371300586627	ITGA9	3680	integrin, alpha 9 [Source:HGNC Symbol;Acc:HGNC:6145]
-0.421693439358896	0.0216413586401661	ZNF295-AS1	150142	ZNF295 antisense RNA 1 [Source:HGNC Symbol;Acc:HGNC:23130]
-0.420963671128339	0.108040006136258	GYG2	8908	glycogenin 2 [Source:HGNC Symbol;Acc:HGNC:4700]
-0.420351135835109	0.0435359550753303	DIEXF	27042	digestive organ expansion factor homolog (zebrafish) [Source:HGNC Symbol;Acc:HGNC:28440]
-0.420138892657553	0.0176205571079114	HOXA11	3207	homeobox A11 [Source:HGNC Symbol;Acc:HGNC:5101]
-0.420132575722687	0.147745091347808	KIAA0513	9764	KIAA0513 [Source:HGNC Symbol;Acc:HGNC:29058]
-0.419819660095845	0.107982840787756	PDGFC	56034	platelet derived growth factor C [Source:HGNC Symbol;Acc:HGNC:8801]
-0.419376201615452	0.0724508669670933	PGM5	5239	phosphoglucomutase 5 [Source:HGNC Symbol;Acc:HGNC:8908]
-0.419178348281177	0.0591576224070754	CLEC12A	160364	C-type lectin domain family 12, member A [Source:HGNC Symbol;Acc:HGNC:31713]
-0.418654961046324	0.0869788553309451	MKNK2	2872	MAP kinase interacting serine/threonine kinase 2 [Source:HGNC Symbol;Acc:HGNC:7111]
-0.418444350577841	0.0942079474792068		150967	DKFZp434H1419 [Source:EntrezGene;Acc:150967]
-0.416618471595476	0.187543764183359	ARSE	415	arylsulfatase E (chondrodysplasia punctata 1) [Source:HGNC Symbol;Acc:HGNC:719]
-0.416589321200496	0.0519120322788717	FZR1	51343	fizzy/cell division cycle 20 related 1 (Drosophila) [Source:HGNC Symbol;Acc:HGNC:24824]
-0.416396529026236	0.0816965789364863	PJA2	9867	praja ring finger 2, E3 ubiquitin protein ligase [Source:HGNC Symbol;Acc:HGNC:17481]
-0.41597398548857	0.0132134569286832	SAMD13	148418	sterile alpha motif domain containing 13 [Source:HGNC Symbol;Acc:HGNC:24582]
-0.415663940276648	0.000419515843181125	PTPN14	5784	protein tyrosine phosphatase, non-receptor type 14 [Source:HGNC Symbol;Acc:HGNC:9647]
-0.415131675030495	0.0732210710408473	EVI5	7813	ecotropic viral integration site 5 [Source:HGNC Symbol;Acc:HGNC:3501]
-0.414191315313074	0.0486233784015568	FAM103A1	83640	family with sequence similarity 103, member A1 [Source:HGNC Symbol;Acc:HGNC:31022]
-0.413619634196694	0.000650658775407105	PPP2R3A	5523	protein phosphatase 2, regulatory subunit B'', alpha [Source:HGNC Symbol;Acc:HGNC:9307]
-0.413493536362651	0.0738595474697787	SLC22A5	6584	solute carrier family 22 (organic cation/carnitine transporter), member 5 [Source:HGNC Symbol;Acc:HGNC:10969]
-0.412593811091911	0.159580315017788	FBXO33	254170	F-box protein 33 [Source:HGNC Symbol;Acc:HGNC:19833]
-0.412020184875138	0.0943765101614755	AGPAT6	137964	1-acylglycerol-3-phosphate O-acyltransferase 6 [Source:HGNC Symbol;Acc:HGNC:20880]
-0.41175747937883	0.10784567329604	ACER3	55331	alkaline ceramidase 3 [Source:HGNC Symbol;Acc:HGNC:16066]
-0.410468275407967	0.0369360794979638	NRROS	375387	negative regulator of reactive oxygen species [Source:HGNC Symbol;Acc:HGNC:24613]
-0.409954576091105	0.137141099823595	FZD3	7976	frizzled class receptor 3 [Source:HGNC Symbol;Acc:HGNC:4041]
-0.409693636812869	0.13311787962714	GRB10	2887	growth factor receptor-bound protein 10 [Source:HGNC Symbol;Acc:HGNC:4564]
-0.409123145760832	0.193779133806474	PRICKLE1	144165	prickle homolog 1 (Drosophila) [Source:HGNC Symbol;Acc:HGNC:17019]
-0.408900607544586	0.131383729368201	LUM	4060	lumican [Source:HGNC Symbol;Acc:HGNC:6724]
-0.408826007590102	0.036320001737497	CRYAB	1410	crystallin, alpha B [Source:HGNC Symbol;Acc:HGNC:2389]
-0.40872111599288	0.0817778547522804	NEDD9	4739	neural precursor cell expressed, developmentally down-regulated 9 [Source:HGNC Symbol;Acc:HGNC:7733]
-0.40815363488895	0.0116623317844006	NFU1	27247	NFU1 iron-sulfur cluster scaffold [Source:HGNC Symbol;Acc:HGNC:16287]
-0.408075829908059	0.124619935911247	MEP1A	4224	meprin A, alpha (PABA peptide hydrolase) [Source:HGNC Symbol;Acc:HGNC:7015]
-0.40804547550237	0.124825247805454	NCK2	8440	NCK adaptor protein 2 [Source:HGNC Symbol;Acc:HGNC:7665]
-0.40801098712304	0.0476866814546115	PHYH	5264	phytanoyl-CoA 2-hydroxylase [Source:HGNC Symbol;Acc:HGNC:8940]
-0.407986173505508	0.150177888941018	MINOS1	440574	mitochondrial inner membrane organizing system 1 [Source:HGNC Symbol;Acc:HGNC:32068]
-0.407887520419591	0.103071825773475	CD164	8763	CD164 molecule, sialomucin [Source:HGNC Symbol;Acc:HGNC:1632]
-0.406372408035217	0.176297280746902	CLIC6	54102	chloride intracellular channel 6 [Source:HGNC Symbol;Acc:HGNC:2065]
-0.406083620834212	0.13486563093828	ADAR	103	adenosine deaminase, RNA-specific [Source:HGNC Symbol;Acc:HGNC:225]
-0.406065890344623	0.099190684820221	RNF128	79589	ring finger protein 128, E3 ubiquitin protein ligase [Source:HGNC Symbol;Acc:HGNC:21153]
-0.40605717847727	0.131825057691922	MOSPD1	56180	motile sperm domain containing 1 [Source:HGNC Symbol;Acc:HGNC:25235]
-0.405897206343313	0.0997756979582243		102724208	
-0.40587297456568	0.142706373946237	XK	7504	X-linked Kx blood group [Source:HGNC Symbol;Acc:HGNC:12811]
-0.405303845043172	0.0301028537526286	MIR141	406933	microRNA 141 [Source:HGNC Symbol;Acc:HGNC:31528]
-0.405162179868288	0.0636696840654483	LGALSL	29094	lectin, galactoside-binding-like [Source:HGNC Symbol;Acc:HGNC:25012]
-0.404787184390565	0.0433192273422611	ATRN	8455	attractin [Source:HGNC Symbol;Acc:HGNC:885]
-0.404776027933095	0.0500875742315784	ENDOD1	23052	endonuclease domain containing 1 [Source:HGNC Symbol;Acc:HGNC:29129]
-0.404493899126383	0.0813429473676542	CYBRD1	79901	cytochrome b reductase 1 [Source:HGNC Symbol;Acc:HGNC:20797]
-0.403837746917088	0.0230825324874309		283349	
-0.403595838647262	0.191498493347748	SSX6	280657	synovial sarcoma, X breakpoint 6 (pseudogene) [Source:HGNC Symbol;Acc:HGNC:19652]
-0.403216301940748	0.0261705229637549	HNF4G	3174	hepatocyte nuclear factor 4, gamma [Source:HGNC Symbol;Acc:HGNC:5026]
-0.402858752861588	0.0830065916850547	C21orf91	54149	chromosome 21 open reading frame 91 [Source:HGNC Symbol;Acc:HGNC:16459]
-0.402041365187295	0.0496810963825698	BAD	572	BCL2-associated agonist of cell death [Source:HGNC Symbol;Acc:HGNC:936]
-0.401958983111048	0.0578718749583693	SLC31A2	1318	solute carrier family 31 (copper transporter), member 2 [Source:HGNC Symbol;Acc:HGNC:11017]
-0.401753912941871	0.075213862206461	PLXND1	23129	plexin D1 [Source:HGNC Symbol;Acc:HGNC:9107]
-0.40174843231746	0.00200582420245967	EPB41L4A	64097	erythrocyte membrane protein band 4.1 like 4A [Source:HGNC Symbol;Acc:HGNC:13278]
-0.401724037245209	0.0621971600225213	GM2A	2760	GM2 ganglioside activator [Source:HGNC Symbol;Acc:HGNC:4367]
-0.401610551174125	0.00861681079282178	ST7	7982	suppression of tumorigenicity 7 [Source:HGNC Symbol;Acc:HGNC:11351]
-0.401195009894275	0.19423307640342	DNAJB1	3337	DnaJ (Hsp40) homolog, subfamily B, member 1 [Source:HGNC Symbol;Acc:HGNC:5270]
-0.401070141193059	0.0921268772121625	ANKRD36BP	84832	ankyrin repeat domain 36B pseudogene 1 [Source:HGNC Symbol;Acc:HGNC:28169]
-0.400605745344359	0.139933908977298	PLXNA3	55558	plexin A3 [Source:HGNC Symbol;Acc:HGNC:9101]
-0.4005710458131	0.0190337645575834	MDH1	4190	malate dehydrogenase 1, NAD (soluble) [Source:HGNC Symbol;Acc:HGNC:6970]
-0.398796093386384	0.0386669208813169	BARX2	8538	BARX homeobox 2 [Source:HGNC Symbol;Acc:HGNC:956]
-0.398471299365008	0.00446331106738938	TPRG1L	127262	tumor protein p63 regulated 1-like [Source:HGNC Symbol;Acc:HGNC:27007]
-0.398270972434788	0.0501510487999315	HHLA2	11148	HERV-H LTR-associating 2 [Source:HGNC Symbol;Acc:HGNC:4905]
-0.397791949077237	0.0253452763885946	POR	5447	P450 (cytochrome) oxidoreductase [Source:HGNC Symbol;Acc:HGNC:9208]
-0.396954454463852	0.13246687289329	MAP1B	4131	microtubule-associated protein 1B [Source:HGNC Symbol;Acc:HGNC:6836]
-0.39683542017749	0.0208391444710944	LAMTOR4	389541	late endosomal/lysosomal adaptor, MAPK and MTOR activator 4 [Source:HGNC Symbol;Acc:HGNC:33772]
-0.39652586551812	0.187774847732442	ADTRP	84830	androgen-dependent TFPI-regulating protein [Source:HGNC Symbol;Acc:HGNC:21214]
-0.396381216538324	0.00646275712586728	RNASET2	8635	ribonuclease T2 [Source:HGNC Symbol;Acc:HGNC:21686]
-0.396060789076166	0.0876937393911111	PRSS8	5652	protease, serine, 8 [Source:HGNC Symbol;Acc:HGNC:9491]
-0.395970002409115	0.0115852291198184	ZFP91	80829	ZFP91 zinc finger protein [Source:HGNC Symbol;Acc:HGNC:14983]
-0.395915791126907	0.0337377624845477	GPC6	10082	glypican 6 [Source:HGNC Symbol;Acc:HGNC:4454]
-0.395672640557969	0.00980742163887645	UXS1	80146	UDP-glucuronate decarboxylase 1 [Source:HGNC Symbol;Acc:HGNC:17729]
-0.394241566745243	0.0842727005974941	LACTB	114294	lactamase, beta [Source:HGNC Symbol;Acc:HGNC:16468]
-0.394227492926474	0.0304592972678842	JAM2	58494	junctional adhesion molecule 2 [Source:HGNC Symbol;Acc:HGNC:14686]
-0.394180141540077	0.133052321014166	ZNF626	199777	zinc finger protein 626 [Source:HGNC Symbol;Acc:HGNC:30461]
-0.39415283102733	0.0334592964912998	GNA14	9630	guanine nucleotide binding protein (G protein), alpha 14 [Source:HGNC Symbol;Acc:HGNC:4382]
-0.393968238257646	0.0572678181283148	ANKFN1	162282	ankyrin-repeat and fibronectin type III domain containing 1 [Source:HGNC Symbol;Acc:HGNC:26766]
-0.393193441560206	0.0639041702505682	MFGE8	4240	milk fat globule-EGF factor 8 protein [Source:HGNC Symbol;Acc:HGNC:7036]
-0.393042911286192	0.0810295731960206	C10orf54	64115	chromosome 10 open reading frame 54 [Source:HGNC Symbol;Acc:HGNC:30085]
-0.392858359622849	0.13019052100131	KDM3A	55818	lysine (K)-specific demethylase 3A [Source:HGNC Symbol;Acc:HGNC:20815]
-0.392366973597834	0.120605057442651	RASSF4	83937	Ras association (RalGDS/AF-6) domain family member 4 [Source:HGNC Symbol;Acc:HGNC:20793]
-0.391167986007854	0.0566223598899496	ZNF438	220929	zinc finger protein 438 [Source:HGNC Symbol;Acc:HGNC:21029]
-0.390088334543314	0.188240095482745	KDR	3791	kinase insert domain receptor (a type III receptor tyrosine kinase) [Source:HGNC Symbol;Acc:HGNC:6307]
-0.38961922415471	0.11489490769434	TESK2	10420	testis-specific kinase 2 [Source:HGNC Symbol;Acc:HGNC:11732]
-0.389394389511436	0.0125528082111514	PPAP2A	8611	phosphatidic acid phosphatase type 2A [Source:HGNC Symbol;Acc:HGNC:9228]
-0.389051967374385	0.164177556157686	GLTSCR2	29997	glioma tumor suppressor candidate region gene 2 [Source:HGNC Symbol;Acc:HGNC:4333]
-0.389025527886478	0.177931299761303	GNAQ	2776	guanine nucleotide binding protein (G protein), q polypeptide [Source:HGNC Symbol;Acc:HGNC:4390]
-0.388543894142805	0.0789531263883283	FAM20B	9917	family with sequence similarity 20, member B [Source:HGNC Symbol;Acc:HGNC:23017]
-0.387898900848495	0.161612862251194	EXPH5	23086	exophilin 5 [Source:HGNC Symbol;Acc:HGNC:30578]
-0.387419588584703	0.0448781793713446	NAGA	4668	N-acetylgalactosaminidase, alpha- [Source:HGNC Symbol;Acc:HGNC:7631]
-0.387347296163173	0.129288992534773	SMIM14	201895	small integral membrane protein 14 [Source:HGNC Symbol;Acc:HGNC:27321]
-0.387280067891127	0.178122766146947	IGSF3	3321	immunoglobulin superfamily, member 3 [Source:HGNC Symbol;Acc:HGNC:5950]
-0.386903077780679	0.06780067629575	AP1M2	10053	adaptor-related protein complex 1, mu 2 subunit [Source:HGNC Symbol;Acc:HGNC:558]
-0.38646680421264	0.130576048237141	SEPP1	6414	selenoprotein P, plasma, 1 [Source:HGNC Symbol;Acc:HGNC:10751]
-0.386148376499451	0.0088466071391766	CDC14A	8556	cell division cycle 14A [Source:HGNC Symbol;Acc:HGNC:1718]
-0.385505669291877	0.00604545720362304	PECR	55825	peroxisomal trans-2-enoyl-CoA reductase [Source:HGNC Symbol;Acc:HGNC:18281]
-0.385362392261389	0.0964310570290088	PDLIM1	9124	PDZ and LIM domain 1 [Source:HGNC Symbol;Acc:HGNC:2067]
-0.385095459134488	0.184425987600631	SGCE	8910	sarcoglycan, epsilon [Source:HGNC Symbol;Acc:HGNC:10808]
-0.384990136114135	0.1755756726204	FBXL3	26224	F-box and leucine-rich repeat protein 3 [Source:HGNC Symbol;Acc:HGNC:13599]
-0.384807021943484	0.120738962325809	SLC40A1	30061	solute carrier family 40 (iron-regulated transporter), member 1 [Source:HGNC Symbol;Acc:HGNC:10909]
-0.3842520054106	0.0406577522754237	SRRD	402055	SRR1 domain containing [Source:HGNC Symbol;Acc:HGNC:33910]
-0.384152656728018	0.0309943248521918	CMPK1	51727	cytidine monophosphate (UMP-CMP) kinase 1, cytosolic [Source:HGNC Symbol;Acc:HGNC:18170]
-0.383724880509131	0.180900836822136	GPRC5B	51704	G protein-coupled receptor, class C, group 5, member B [Source:HGNC Symbol;Acc:HGNC:13308]
-0.383499286262271	0.0424014066803047	GPX3	2878	glutathione peroxidase 3 (plasma) [Source:HGNC Symbol;Acc:HGNC:4555]
-0.383169315704489	0.0865271280809169	S100A6	6277	S100 calcium binding protein A6 [Source:HGNC Symbol;Acc:HGNC:10496]
-0.383016988697691	0.190393476791604	mars-01	64757	mitochondrial amidoxime reducing component 1 [Source:HGNC Symbol;Acc:HGNC:26189]
-0.382793159119343	0.0881354045619018	RC3H2	54542	ring finger and CCCH-type domains 2 [Source:HGNC Symbol;Acc:HGNC:21461]
-0.38271337020302	0.121382798272605	LRRC39	127495	leucine rich repeat containing 39 [Source:HGNC Symbol;Acc:HGNC:28228]
-0.38267827541194	0.100880839795002	KIR3DL1	3811	killer cell immunoglobulin-like receptor, three domains, long cytoplasmic tail, 1 [Source:HGNC Symbol;Acc:HGNC:6338]
-0.382263705213445	0.126245174906678	TYW1B	441250	tRNA-yW synthesizing protein 1 homolog B (S. cerevisiae) [Source:HGNC Symbol;Acc:HGNC:33908]
-0.381747379756199	0.115086116868197	FBLN1	2192	fibulin 1 [Source:HGNC Symbol;Acc:HGNC:3600]
-0.381323762865578	0.0775702760707903	MOB1A	55233	MOB kinase activator 1A [Source:HGNC Symbol;Acc:HGNC:16015]
-0.380570670889207	0.0880358612286987	GABARAPL1	23710	GABA(A) receptor-associated protein like 1 [Source:HGNC Symbol;Acc:HGNC:4068]
-0.380487706724186	0.0117889964786978	REEP3	221035	receptor accessory protein 3 [Source:HGNC Symbol;Acc:HGNC:23711]
-0.380444991841692	0.0616417970341048	FTSJ3	117246	FtsJ homolog 3 (E. coli) [Source:HGNC Symbol;Acc:HGNC:17136]
-0.380140311128402	0.177172503911001	ZNF718	255403	zinc finger protein 718 [Source:HGNC Symbol;Acc:HGNC:26889]
-0.380012814533995	0.105737424929012	LIX1L	128077	Lix1 homolog (chicken) like [Source:HGNC Symbol;Acc:HGNC:28715]
-0.378879505337034	0.049966241197009	CTNS	1497	cystinosin, lysosomal cystine transporter [Source:HGNC Symbol;Acc:HGNC:2518]
-0.378472450365679	0.174835269519918	ECI2	10455	enoyl-CoA delta isomerase 2 [Source:HGNC Symbol;Acc:HGNC:14601]
-0.377993324395665	0.147558537539906	TAS2R46	259292	taste receptor, type 2, member 46 [Source:HGNC Symbol;Acc:HGNC:18877]
-0.37799214267543	0.193134405662737	PXDC1	221749	PX domain containing 1 [Source:HGNC Symbol;Acc:HGNC:21361]
-0.377506608000752	0.0794574836316458	CBX7	23492	chromobox homolog 7 [Source:HGNC Symbol;Acc:HGNC:1557]
-0.377482622031945	0.0283925816268923	TFCP2L1	29842	transcription factor CP2-like 1 [Source:HGNC Symbol;Acc:HGNC:17925]
-0.377212871648873	0.132364043081179	AHNAK2	113146	AHNAK nucleoprotein 2 [Source:HGNC Symbol;Acc:HGNC:20125]
-0.377187363280408	0.0218307253417545	PVRL3	25945	poliovirus receptor-related 3 [Source:HGNC Symbol;Acc:HGNC:17664]
-0.377135382148608	0.036352443093934	BVES	11149	blood vessel epicardial substance [Source:HGNC Symbol;Acc:HGNC:1152]
-0.377061690976285	0.0316753973386429	MAML3	55534	mastermind-like 3 (Drosophila) [Source:HGNC Symbol;Acc:HGNC:16272]
-0.37666536956237	0.131934562326151	DYNLL2	140735	dynein, light chain, LC8-type 2 [Source:HGNC Symbol;Acc:HGNC:24596]
-0.376457367365744	0.101106957808094	HMGCL	3155	3-hydroxymethyl-3-methylglutaryl-CoA lyase [Source:HGNC Symbol;Acc:HGNC:5005]
-0.376022596183756	0.0939727991489546	SLC25A23	79085	solute carrier family 25 (mitochondrial carrier; phosphate carrier), member 23 [Source:HGNC Symbol;Acc:HGNC:19375]
-0.375918600234106	0.0397401753813071	ARL2BP	23568	ADP-ribosylation factor-like 2 binding protein [Source:HGNC Symbol;Acc:HGNC:17146]
-0.375904495727183	0.0700441433583053	NDUFB6	4712	NADH dehydrogenase (ubiquinone) 1 beta subcomplex, 6, 17kDa [Source:HGNC Symbol;Acc:HGNC:7701]
-0.375885241529869	0.0514161077916359	NDUFC1	4717	NADH dehydrogenase (ubiquinone) 1, subcomplex unknown, 1, 6kDa [Source:HGNC Symbol;Acc:HGNC:7705]
-0.375574318784703	0.000403082817300207	CLN5	1203	ceroid-lipofuscinosis, neuronal 5 [Source:HGNC Symbol;Acc:HGNC:2076]
-0.375356533723587	0.0433796627516928	SEMA5A	9037	sema domain, seven thrombospondin repeats (type 1 and type 1-like), transmembrane domain (TM) and short cytoplasmic domain, (semaphorin) 5A [Source:HGNC Symbol;Acc:HGNC:10736]
-0.375240251596513	0.164010015750188	HERC5	51191	HECT and RLD domain containing E3 ubiquitin protein ligase 5 [Source:HGNC Symbol;Acc:HGNC:24368]
-0.37439089458362	0.0443372138358219	TGFBR3	7049	transforming growth factor, beta receptor III [Source:HGNC Symbol;Acc:HGNC:11774]

-0.374004195000954	0.0358342737362737	GPR125	166647	G protein-coupled receptor 125 [Source:HGNC Symbol;Acc:HGNC:13839]
-0.37368608880236	0.106033677811765	NAAA	27163	N-acylethanolamine acid amidase [Source:HGNC Symbol;Acc:HGNC:736]
-0.373644886650619	0.123249652649428	YPEL3	83719	yippee-like 3 (Drosophila) [Source:HGNC Symbol;Acc:HGNC:18327]
-0.372659450084738	0.00708069876449216		23766	GABA(A) receptors associated protein like 3, pseudogene [Source:EntrezGene;Acc:23766]
-0.372606554109444	0.0471694273904683	LRP6	4040	low density lipoprotein receptor-related protein 6 [Source:HGNC Symbol;Acc:HGNC:6698]
-0.372027126730034	0.13790387970966	PTH	5741	parathyroid hormone [Source:HGNC Symbol;Acc:HGNC:9606]
-0.371451084513128	0.0981773194022924	TMEM203	94107	transmembrane protein 203 [Source:HGNC Symbol;Acc:HGNC:28217]
-0.371384781427224	0.077054383823908	ZCCHC14	23174	zinc finger, CCHC domain containing 14 [Source:HGNC Symbol;Acc:HGNC:24134]
-0.370039875387299	0.181189469845871	NID1	4811	nidogen 1 [Source:HGNC Symbol;Acc:HGNC:7821]
-0.369156292073324	0.0809711656971623	S100A4	6275	S100 calcium binding protein A4 [Source:HGNC Symbol;Acc:HGNC:10494]
-0.368836740343545	0.0773449042932728	TMEM120A	83862	transmembrane protein 120A [Source:HGNC Symbol;Acc:HGNC:21697]
-0.368410247712045	0.179127807560083	PGAP3	93210	post-GPI attachment to proteins 3 [Source:HGNC Symbol;Acc:HGNC:23719]
-0.367637683664116	0.0721823538124599	WLS	79971	wntless Wnt ligand secretion mediator [Source:HGNC Symbol;Acc:HGNC:30238]
-0.367480284646004	0.194052260464732	PRRC1	133619	proline-rich coiled-coil 1 [Source:HGNC Symbol;Acc:HGNC:28164]
-0.367284126331951	0.00757660866269836	ATOX1	475	antioxidant 1 copper chaperone [Source:HGNC Symbol;Acc:HGNC:798]
-0.366705149984572	0.0806843315079124	MSL1	339287	male-specific lethal 1 homolog (Drosophila) [Source:HGNC Symbol;Acc:HGNC:27905]
-0.366599067563646	0.106563017342565	ACVR1	90	activin A receptor, type I [Source:HGNC Symbol;Acc:HGNC:171]
-0.366543556493785	0.18511676202861	FKBP9	11328	FK506 binding protein 9, 63 kDa [Source:HGNC Symbol;Acc:HGNC:3725]
-0.366462104069789	0.0493857642500326	DUSP3	1845	dual specificity phosphatase 3 [Source:HGNC Symbol;Acc:HGNC:3069]
-0.366391147364931	0.0135386347427164	ATP5J2	9551	ATP synthase, H+ transporting, mitochondrial Fo complex, subunit F2 [Source:HGNC Symbol;Acc:HGNC:848]
-0.366368312971209	0.0755804555056324	RNF135	84282	ring finger protein 135 [Source:HGNC Symbol;Acc:HGNC:21158]
-0.365975363140525	0.108208064154137	ATP6V1A	523	ATPase, H+ transporting, lysosomal 70kDa, V1 subunit A [Source:HGNC Symbol;Acc:HGNC:851]
-0.3657830312696	0.178808664819688	C15orf48	84419	chromosome 15 open reading frame 48 [Source:HGNC Symbol;Acc:HGNC:29898]
-0.365387594358823	0.0187206575126535	ZBTB38	253461	zinc finger and BTB domain containing 38 [Source:HGNC Symbol;Acc:HGNC:26636]
-0.364460898934532	0.146898870672695	NRBP1	29959	nuclear receptor binding protein 1 [Source:HGNC Symbol;Acc:HGNC:7993]
-0.364407934119826	0.113792557705799	CAV2	858	caveolin 2 [Source:HGNC Symbol;Acc:HGNC:1528]
-0.364168464105498	0.066576281382838	CHRNB1	1140	cholinergic receptor, nicotinic, beta 1 (muscle) [Source:HGNC Symbol;Acc:HGNC:1961]
-0.363811841388138	0.0108310240171313	CLCN3	1182	chloride channel, voltage-sensitive 3 [Source:HGNC Symbol;Acc:HGNC:2021]
-0.363627293622835	0.142398496514107	AKR1B1	231	aldo-keto reductase family 1, member B1 (aldose reductase) [Source:HGNC Symbol;Acc:HGNC:381]
-0.363061737716694	0.190365963726499	LCE3D	84648	late cornified envelope 3D [Source:HGNC Symbol;Acc:HGNC:16615]
-0.362784148338007	0.106734473485264	PTN	5764	pleiotrophin [Source:HGNC Symbol;Acc:HGNC:9630]
-0.362511484950323	0.0757219699971542	KRBOX4	55634	KRAB box domain containing 4 [Source:HGNC Symbol;Acc:HGNC:26007]
-0.362369944966291	0.0585311581968364	CFTR	1080	cystic fibrosis transmembrane conductance regulator (ATP-binding cassette sub-family C, member 7) [Source:HGNC Symbol;Acc:HGNC:1884]
-0.362250256735973	0.0659947495680809	FAM13C	220965	family with sequence similarity 13, member C [Source:HGNC Symbol;Acc:HGNC:19371]
-0.362115101613182	0.090587868579323	GEMIN8P4	492303	gem (nuclear organelle) associated protein 8 pseudogene 4 [Source:HGNC Symbol;Acc:HGNC:37979]
-0.361949402004585	0.0591708392750704	CCDC47	57003	coiled-coil domain containing 47 [Source:HGNC Symbol;Acc:HGNC:24856]
-0.361918342262996	0.0984656382336927	YWHAB	7529	tyrosine 3-monooxygenase/tryptophan 5-monooxygenase activation protein, beta [Source:HGNC Symbol;Acc:HGNC:12849]
-0.361850014688759	0.019476243771603	GID8	54994	GID complex subunit 8 [Source:HGNC Symbol;Acc:HGNC:15857]
-0.361807047221441	0.071799727134286	LGALS1	3956	lectin, galactoside-binding, soluble, 1 [Source:HGNC Symbol;Acc:HGNC:6561]
-0.361377897589429	0.137461370147066	EPC2	26122	enhancer of polycomb homolog 2 (Drosophila) [Source:HGNC Symbol;Acc:HGNC:24543]
-0.360994403459475	0.135976143032312	APOE	348	apolipoprotein E [Source:HGNC Symbol;Acc:HGNC:613]
-0.360804786596554	0.113996782680904	ATL3	25923	atlastin GTPase 3 [Source:HGNC Symbol;Acc:HGNC:24526]
-0.360728095122773	0.0279391547158054	DCHS2	54798	dachsous cadherin-related 2 [Source:HGNC Symbol;Acc:HGNC:23111]
-0.359805538178903	0.0936046027994407	ZBTB44	29068	zinc finger and BTB domain containing 44 [Source:HGNC Symbol;Acc:HGNC:25001]
-0.359633705315163	0.0555857070273954	FAM3D	131177	family with sequence similarity 3, member D [Source:HGNC Symbol;Acc:HGNC:18665]
-0.359574143230047	0.00639697989639867	RAP1A	5906	RAP1A, member of RAS oncogene family [Source:HGNC Symbol;Acc:HGNC:9855]
-0.359535660043296	0.132256234634206	IL1R2	7850	interleukin 1 receptor, type II [Source:HGNC Symbol;Acc:HGNC:5994]
-0.35915700270188	0.0204127206630304	TMCC1	23023	transmembrane and coiled-coil domain family 1 [Source:HGNC Symbol;Acc:HGNC:29116]
-0.358981014413281	0.092319560234574	NCAM1	4684	neural cell adhesion molecule 1 [Source:HGNC Symbol;Acc:HGNC:7656]
-0.358978494569937	0.0290502757109013	RNF181	51255	ring finger protein 181 [Source:HGNC Symbol;Acc:HGNC:28037]
-0.358589309242543	0.0691898803341307	OXR1	55074	oxidation resistance 1 [Source:HGNC Symbol;Acc:HGNC:15822]
-0.357796136738038	0.166658297954051	CTSL	1514	cathepsin L [Source:HGNC Symbol;Acc:HGNC:2537]
-0.357795570935222	0.0621626102531778	MYH10	4628	myosin, heavy chain 10, non-muscle [Source:HGNC Symbol;Acc:HGNC:7568]
-0.357763570459105	0.194350556636447	SIRPA	140885	signal-regulatory protein alpha [Source:HGNC Symbol;Acc:HGNC:9662]
-0.357415626158579	0.18524275047846	KLF11	8462	Kruppel-like factor 11 [Source:HGNC Symbol;Acc:HGNC:11811]
-0.357314659233061	0.119196136321046	C7orf26	79034	chromosome 7 open reading frame 26 [Source:HGNC Symbol;Acc:HGNC:21702]
-0.356903707290008	0.0476462062994423	TOR1AIP1	26092	torsin A interacting protein 1 [Source:HGNC Symbol;Acc:HGNC:29456]
-0.356751359159717	0.183429235304654	ANO6	196527	anoctamin 6 [Source:HGNC Symbol;Acc:HGNC:25240]
-0.356506847430128	0.17638848359132	RAC1	5879	ras-related C3 botulinum toxin substrate 1 (rho family, small GTP binding protein Rac1) [Source:HGNC Symbol;Acc:HGNC:9801]
-0.356484985456462	0.145843379370408	ANO5	203859	anoctamin 5 [Source:HGNC Symbol;Acc:HGNC:27337]
-0.35611555359154	0.131359877428807	TNFRSF17	608	tumor necrosis factor receptor superfamily, member 17 [Source:HGNC Symbol;Acc:HGNC:11913]
-0.355953910288642	0.0407378205862942	IL5RA	3568	interleukin 5 receptor, alpha [Source:HGNC Symbol;Acc:HGNC:6017]
-0.355934351274743	0.193947963511511	MFSD4	148808	major facilitator superfamily domain containing 4 [Source:HGNC Symbol;Acc:HGNC:25433]
-0.355479797633791	0.109885075199535	SRC	6714	SRC proto-oncogene, non-receptor tyrosine kinase [Source:HGNC Symbol;Acc:HGNC:11283]
-0.35506883452679	0.030320177752943	TGFBRAP1	9392	transforming growth factor, beta receptor associated protein 1 [Source:HGNC Symbol;Acc:HGNC:16836]
-0.354930461682867	0.0553798088449158	PTDSS2	81490	phosphatidylserine synthase 2 [Source:HGNC Symbol;Acc:HGNC:15463]
-0.354627913743559	0.00545800686035962	RAVER2	55225	ribonucleoprotein, PTB-binding 2 [Source:HGNC Symbol;Acc:HGNC:25577]
-0.354574358588055	0.145564440843174	PCYOX1	51449	prenylcysteine oxidase 1 [Source:HGNC Symbol;Acc:HGNC:20588]
-0.354360772649381	0.0837884505918565	POF1B	79983	premature ovarian failure, 1B [Source:HGNC Symbol;Acc:HGNC:13711]
-0.354322948728529	0.0746410617783843	MYO18A	399687	myosin XVIIIA [Source:HGNC Symbol;Acc:HGNC:31104]
-0.354217672943118	0.144108561022048	SH3BP4	23677	SH3-domain binding protein 4 [Source:HGNC Symbol;Acc:HGNC:10826]
-0.353979309555359	0.0255398155076956	CYGB	114757	cytoglobin [Source:HGNC Symbol;Acc:HGNC:16505]
-0.353732969625562	0.154592035220291	SCIN	85477	scinderin [Source:HGNC Symbol;Acc:HGNC:21695]
-0.353609124717858	0.0860756815883529	SNX12	29934	sorting nexin 12 [Source:HGNC Symbol;Acc:HGNC:14976]
-0.353460973625826	0.143934849833585	CDKN1B	1027	cyclin-dependent kinase inhibitor 1B (p27, Kip1) [Source:HGNC Symbol;Acc:HGNC:1785]
-0.353407460906368	0.161166277769972	ZNF57	126295	zinc finger protein 57 [Source:HGNC Symbol;Acc:HGNC:13125]
-0.353101091598607	0.130650271175887	GAB2	9846	GRB2-associated binding protein 2 [Source:HGNC Symbol;Acc:HGNC:14458]
-0.352504854500528	0.111076332649243	NRARP	441478	NOTCH-regulated ankyrin repeat protein [Source:HGNC Symbol;Acc:HGNC:33843]
-0.352143561456805	0.0957538813917425	NDUFC2	4718	NADH dehydrogenase (ubiquinone) 1, subcomplex unknown, 2, 14.5kDa [Source:HGNC Symbol;Acc:HGNC:7706]
-0.352108976719628	0.14058293451188	RNASE6	6039	ribonuclease, RNase A family, k6 [Source:HGNC Symbol;Acc:HGNC:10048]
-0.351976961697372	0.161078688226253	MS4A6A	64231	membrane-spanning 4-domains, subfamily A, member 6A [Source:HGNC Symbol;Acc:HGNC:13375]
-0.351756418039665	0.139281893774095	MCC	4163	mutated in colorectal cancers [Source:HGNC Symbol;Acc:HGNC:6935]
-0.351737862505611	0.0024910607455856	DPYSL2	1808	dihydropyrimidinase-like 2 [Source:HGNC Symbol;Acc:HGNC:3014]
-0.35144171329324	0.0116150429939906	GNPAT	8443	glyceronephosphate O-acyltransferase [Source:HGNC Symbol;Acc:HGNC:4416]
-0.351410483423646	0.187495256340956	FITM2	128486	fat storage-inducing transmembrane protein 2 [Source:HGNC Symbol;Acc:HGNC:16135]
-0.351355256919246	0.0143066284612485	NOP10	55505	NOP10 ribonucleoprotein [Source:HGNC Symbol;Acc:HGNC:14378]
-0.351000967733511	0.0762621234503116	RBM23	55147	RNA binding motif protein 23 [Source:HGNC Symbol;Acc:HGNC:20155]
-0.350318290098507	0.107933560242644	NR5A2	2494	nuclear receptor subfamily 5, group A, member 2 [Source:HGNC Symbol;Acc:HGNC:7984]
-0.350291167425672	0.121178272086533	C20orf96	140680	chromosome 20 open reading frame 96 [Source:HGNC Symbol;Acc:HGNC:16227]
-0.350181809606339	0.0291855398454027	SLC11A2	4891	solute carrier family 11 (proton-coupled divalent metal ion transporter), member 2 [Source:HGNC Symbol;Acc:HGNC:10908]
-0.34980284148938	0.193155530889151	LINC00526	147525	long intergenic non-protein coding RNA 526 [Source:HGNC Symbol;Acc:HGNC:28278]
-0.349723303391889	0.0067820660661759	CNOT6	57472	CCR4-NOT transcription complex, subunit 6 [Source:HGNC Symbol;Acc:HGNC:14099]
-0.349575470138957	0.00796716652659054	TAOK1	57551	TAO kinase 1 [Source:HGNC Symbol;Acc:HGNC:29259]
-0.348934258684695	0.0105249324421452	TEX2	55852	testis expressed 2 [Source:HGNC Symbol;Acc:HGNC:30884]
-0.348932163254345	0.143510527130778	C11orf24	53838	chromosome 11 open reading frame 24 [Source:HGNC Symbol;Acc:HGNC:1174]
-0.348824519808431	0.0658239270918836	SPATS2	65244	spermatogenesis associated, serine-rich 2 [Source:HGNC Symbol;Acc:HGNC:18650]
-0.348702237737275	0.0136147352205154	APOA1BP	128240	apolipoprotein A-I binding protein [Source:HGNC Symbol;Acc:HGNC:18453]
-0.34840876019788	0.0138478747487099	CFD	1675	complement factor D (adipsin) [Source:HGNC Symbol;Acc:HGNC:2771]
-0.347929956717565	0.18465599738459	FRRS1	391059	ferric-chelate reductase 1 [Source:HGNC Symbol;Acc:HGNC:27622]
-0.347882369268714	0.114422312602309	MAOB	4129	monoamine oxidase B [Source:HGNC Symbol;Acc:HGNC:6834]
-0.34724070432023	0.106382391278229	C2orf42	54980	chromosome 2 open reading frame 42 [Source:HGNC Symbol;Acc:HGNC:26056]
-0.347129747437752	0.0719280235490795	LY6E	4061	lymphocyte antigen 6 complex, locus E [Source:HGNC Symbol;Acc:HGNC:6727]
-0.346899371647659	0.0303201817614004	TACO1	51204	translational activator of mitochondrially encoded cytochrome c oxidase I [Source:HGNC Symbol;Acc:HGNC:24316]
-0.346659311102457	0.123696968316974	ZCCHC2	54877	zinc finger, CCHC domain containing 2 [Source:HGNC Symbol;Acc:HGNC:22916]
-0.34615752520966	0.154287109448884	GPR63	81491	G protein-coupled receptor 63 [Source:HGNC Symbol;Acc:HGNC:13302]
-0.346104890538299	0.188261255878159	XYLT1	64131	xylosyltransferase I [Source:HGNC Symbol;Acc:HGNC:15516]
-0.345919545107148	0.0439959958336887	SLC8B1	80024	solute carrier family 8 (sodium/lithium/calcium exchanger), member B1 [Source:HGNC Symbol;Acc:HGNC:26175]
-0.345568372598244	0.0273741654650365	REXO2	25996	RNA exonuclease 2 [Source:HGNC Symbol;Acc:HGNC:17851]
-0.345494171102991	0.165424063305717	CPTP	80772	ceramide-1-phosphate transfer protein [Source:HGNC Symbol;Acc:HGNC:28116]
-0.345369618155778	0.174483900614282	FAM84A	151354	family with sequence similarity 84, member A [Source:HGNC Symbol;Acc:HGNC:20743]
-0.345151160798462	0.0723862938175864	MAP3K2	10746	mitogen-activated protein kinase kinase kinase 2 [Source:HGNC Symbol;Acc:HGNC:6854]
-0.34492304241985	0.132983442406369	HNRNPA0	10949	heterogeneous nuclear ribonucleoprotein A0 [Source:HGNC Symbol;Acc:HGNC:5030]
-0.344714087818147	0.0667560649442289	BCAP31	10134	B-cell receptor-associated protein 31 [Source:HGNC Symbol;Acc:HGNC:16695]
-0.344544172402782	0.19889876167755	HIST1H2BG	8339	histone cluster 1, H2bg [Source:HGNC Symbol;Acc:HGNC:4746]
-0.344525142871759	0.151454257727543	KIAA0825	285600	KIAA0825 [Source:HGNC Symbol;Acc:HGNC:28532]
-0.344517457777435	0.176518193969652	IFI27	3429	interferon, alpha-inducible protein 27 [Source:HGNC Symbol;Acc:HGNC:5397]
-0.344497308282553	0.153164772618941	ELF3	1999	E74-like factor 3 (ets domain transcription factor, epithelial-specific) [Source:HGNC Symbol;Acc:HGNC:3318]
-0.344320115956379	0.164231358247074	YWHAZ	7534	tyrosine 3-monooxygenase/tryptophan 5-monooxygenase activation protein, zeta [Source:HGNC Symbol;Acc:HGNC:12855]
-0.343477130372735	0.0509918932491566	HPS4	89781	Hermansky-Pudlak syndrome 4 [Source:HGNC Symbol;Acc:HGNC:15844]
-0.342726711027269	0.198144858887379	FAM151B	167555	family with sequence similarity 151, member B [Source:HGNC Symbol;Acc:HGNC:33716]
-0.342709560850455	0.00523032227284678	SDHB	6390	succinate dehydrogenase complex, subunit B, iron sulfur (Ip) [Source:HGNC Symbol;Acc:HGNC:10681]
-0.342619256215188	0.124555764395487	PRDM7	11105	PR domain containing 7 [Source:HGNC Symbol;Acc:HGNC:9351]
-0.342591065709335	0.0988996503612054	AVPR1A	552	arginine vasopressin receptor 1A [Source:HGNC Symbol;Acc:HGNC:895]
-0.342288726086418	0.0862653557161268	SEPN1	57190	selenoprotein N, 1 [Source:HGNC Symbol;Acc:HGNC:15999]
-0.341971612346686	0.155989002795853	SLC35D1	23169	solute carrier family 35 (UDP-GlcA/UDP-GalNAc transporter), member D1 [Source:HGNC Symbol;Acc:HGNC:20800]
-0.341917124940594	0.142597431508551	XPR1	9213	xenotropic and polytropic retrovirus receptor 1 [Source:HGNC Symbol;Acc:HGNC:12827]
-0.341853871688175	0.0286404598016941	UBE2R2	54926	ubiquitin-conjugating enzyme E2R 2 [Source:HGNC Symbol;Acc:HGNC:19907]
-0.341689065371919	0.122347701761756	MAP1LC3B	81631	microtubule-associated protein 1 light chain 3 beta [Source:HGNC Symbol;Acc:HGNC:13352]
-0.341495935057863	0.0585374092545427	GINM1	116254	glycoprotein integral membrane 1 [Source:HGNC Symbol;Acc:HGNC:21074]
-0.341441796138705	0.171362130159599	SMPDL3B	27293	sphingomyelin phosphodiesterase, acid-like 3B [Source:HGNC Symbol;Acc:HGNC:21416]
-0.341393886661507	0.143938886389259	LIPA	3988	lipase A, lysosomal acid, cholesterol esterase [Source:HGNC Symbol;Acc:HGNC:6617]
-0.341272538174413	0.0402570591463854	FAM8A1	51439	family with sequence similarity 8, member A1 [Source:HGNC Symbol;Acc:HGNC:16372]
-0.34126361862995	0.0787620204343312	CLCN7	1186	chloride channel, voltage-sensitive 7 [Source:HGNC Symbol;Acc:HGNC:2025]
-0.340850330684774	0.0874222300503502	PARP9	83666	poly (ADP-ribose) polymerase family, member 9 [Source:HGNC Symbol;Acc:HGNC:24118]
-0.340661538378272	0.159330261729246	IGJ	3512	immunoglobulin J polypeptide, linker protein for immunoglobulin alpha and mu polypeptides [Source:HGNC Symbol;Acc:HGNC:5713]
-0.34062735574086	0.121348059526027	CGRRF1	10668	cell growth regulator with ring finger domain 1 [Source:HGNC Symbol;Acc:HGNC:15528]
-0.340549578193156	0.00779063870037449	SSH1	54434	slingshot protein phosphatase 1 [Source:HGNC Symbol;Acc:HGNC:30579]
-0.340529013453385	0.0790201760487561	WDR45	11152	WD repeat domain 45 [Source:HGNC Symbol;Acc:HGNC:28912]
-0.340457684943735	0.0546486143249302	CTSZ	1522	cathepsin Z [Source:HGNC Symbol;Acc:HGNC:2547]
-0.340376898393198	0.0854534360380301	CTSD	1509	cathepsin D [Source:HGNC Symbol;Acc:HGNC:2529]
-0.340265787843651	0.160484107469952	UGP2	7360	UDP-glucose pyrophosphorylase 2 [Source:HGNC Symbol;Acc:HGNC:12527]

-0.340176438112548	0.132785877468139	SCAP	22937	SREBF chaperone [Source:HGNC Symbol;Acc:HGNC:30634]
-0.340114310990399	0.00926138929766976	NFIA	4774	nuclear factor I/A [Source:HGNC Symbol;Acc:HGNC:7784]
-0.339890207101279	0.160295799569959	FLCN	201163	folliculin [Source:HGNC Symbol;Acc:HGNC:27310]
-0.339849913139752	0.183722004374609	TTR	7276	transthyretin [Source:HGNC Symbol;Acc:HGNC:12405]
-0.33982802880844	0.0850375635736042	TMEM8A	58986	transmembrane protein 8A [Source:HGNC Symbol;Acc:HGNC:17205]
-0.339559027151792	0.132678212407254	AKT3	10000	v-akt murine thymoma viral oncogene homolog 3 [Source:HGNC Symbol;Acc:HGNC:393]
-0.339373801510231	0.0527863030477534	C6orf120	387263	chromosome 6 open reading frame 120 [Source:HGNC Symbol;Acc:HGNC:21247]
-0.339083568885765	0.146106121180199	POFUT1	23509	protein O-fucosyltransferase 1 [Source:HGNC Symbol;Acc:HGNC:14988]
-0.338314021613643	0.122665657149019		391722	
-0.33815858101951	0.14986296315364	ZG16	653808	zymogen granule protein 16 [Source:HGNC Symbol;Acc:HGNC:30961]
-0.338154964566444	0.115250552542527	PTGFRN	5738	prostaglandin F2 receptor inhibitor [Source:HGNC Symbol;Acc:HGNC:9601]
-0.33795984656439	0.00569557206478127	UNC50	25972	unc-50 homolog (C. elegans) [Source:HGNC Symbol;Acc:HGNC:16046]
-0.337953020221944	0.0749142985920615	NCEH1	57552	neutral cholesterol ester hydrolase 1 [Source:HGNC Symbol;Acc:HGNC:29260]
-0.337755122356733	0.131392193468974	ATP2A3	489	ATPase, Ca++ transporting, ubiquitous [Source:HGNC Symbol;Acc:HGNC:813]
-0.337342527506385	0.0537975719695142	MGA	23269	MGA, MAX dimerization protein [Source:HGNC Symbol;Acc:HGNC:14010]
-0.337233725659868	0.0503778462551082	MAN2A1	4124	mannosidase, alpha, class 2A, member 1 [Source:HGNC Symbol;Acc:HGNC:6824]
-0.336733655258132	0.0846782507771254	CLEC4A	50856	C-type lectin domain family 4, member A [Source:HGNC Symbol;Acc:HGNC:13257]
-0.336665096051659	0.0329326872717592	GATB	5188	glutamyl-tRNA(Gln) amidotransferase, subunit B [Source:HGNC Symbol;Acc:HGNC:8849]
-0.336542797369805	0.194666524194775	SHOC2	8036	soc-2 suppressor of clear homolog (C. elegans) [Source:HGNC Symbol;Acc:HGNC:15454]
-0.336498989051296	0.0750573010355538	RAB28	9364	RAB28, member RAS oncogene family [Source:HGNC Symbol;Acc:HGNC:9768]
-0.336105618468711	0.0448841874997839	C9orf78	51759	chromosome 9 open reading frame 78 [Source:HGNC Symbol;Acc:HGNC:24932]
-0.335971375049723	0.0980442655016102	EMC3	55831	ER membrane protein complex subunit 3 [Source:HGNC Symbol;Acc:HGNC:23999]
-0.335809712210489	0.0615522326276142	PEAK1	79834	pseudopodium-enriched atypical kinase 1 [Source:HGNC Symbol;Acc:HGNC:29431]
-0.335768801279578	0.0407857186729881	ZNF3	7551	zinc finger protein 3 [Source:HGNC Symbol;Acc:HGNC:13089]
-0.33516825372157	0.118937404380322	GFM1	85476	G elongation factor, mitochondrial 1 [Source:HGNC Symbol;Acc:HGNC:13780]
-0.335027762275978	0.0326622977626904	CD2AP	23607	CD2-associated protein [Source:HGNC Symbol;Acc:HGNC:14258]
-0.334668782499355	0.146612009044807	HAUS1	115106	HAUS augmin-like complex, subunit 1 [Source:HGNC Symbol;Acc:HGNC:25174]
-0.334534233530266	0.197443966603106	HIST1H3D	8353	histone cluster 1, H3d [Source:HGNC Symbol;Acc:HGNC:4767]
-0.334177734669295	0.0717889157938983	DNAJC14	85406	DnaJ (Hsp40) homolog, subfamily C, member 14 [Source:HGNC Symbol;Acc:HGNC:24581]
-0.334094899393521	0.132173720187668	ARRB1	408	arrestin, beta 1 [Source:HGNC Symbol;Acc:HGNC:711]
-0.33383159365242	0.0651985201561513	CALR	811	calreticulin [Source:HGNC Symbol;Acc:HGNC:1455]
-0.333772473195652	0.0175247680008248	GBA	2629	glucosidase, beta, acid [Source:HGNC Symbol;Acc:HGNC:4177]
-0.33376969408041	0.147672412690212	ZNF518A	9849	zinc finger protein 518A [Source:HGNC Symbol;Acc:HGNC:29009]
-0.333658067066906	0.170971613398384	GOLM1	51280	golgi membrane protein 1 [Source:HGNC Symbol;Acc:HGNC:15451]
-0.333259823295544	0.0999088223115764	PEX13	5194	peroxisomal biogenesis factor 13 [Source:HGNC Symbol;Acc:HGNC:8855]
-0.333191603191433	0.0914279606437742	TSTD2	158427	thiosulfate sulfurtransferase (rhodanese)-like domain containing 2 [Source:HGNC Symbol;Acc:HGNC:30087]
-0.33293084724038	0.190581723709277	HLA-DMA	3108	major histocompatibility complex, class II, DM alpha [Source:HGNC Symbol;Acc:HGNC:4934]
-0.332903938313309	0.185686732702301	ZNF608	57507	zinc finger protein 608 [Source:HGNC Symbol;Acc:HGNC:29238]
-0.332526295270507	0.153097371136247	KPNA4	3840	karyopherin alpha 4 (importin alpha 3) [Source:HGNC Symbol;Acc:HGNC:6397]
-0.332485723299572	0.0958521245339832	TOP2B	7155	topoisomerase (DNA) II beta 180kDa [Source:HGNC Symbol;Acc:HGNC:11990]
-0.332400310647714	0.0675254648489257	CAT	847	catalase [Source:HGNC Symbol;Acc:HGNC:1516]
-0.332354525477505	0.00177281987205128	ENAH	55740	enabled homolog (Drosophila) [Source:HGNC Symbol;Acc:HGNC:18271]
-0.331888445614376	0.0485407691852063	USP46	64854	ubiquitin specific peptidase 46 [Source:HGNC Symbol;Acc:HGNC:20075]
-0.331787180703489	0.120797203535212	CCDC176	80127	coiled-coil domain containing 176 [Source:HGNC Symbol;Acc:HGNC:19855]
-0.331694813637659	0.186225083771686	C5orf63	401207	chromosome 5 open reading frame 63 [Source:HGNC Symbol;Acc:HGNC:40051]
-0.331657898390948	0.113045370497548	SLC18A2	6571	solute carrier family 18 (vesicular monoamine transporter), member 2 [Source:HGNC Symbol;Acc:HGNC:10935]
-0.330948643696224	0.0204690865367599	GUCD1	83606	guanylyl cyclase domain containing 1 [Source:HGNC Symbol;Acc:HGNC:14237]
-0.330866330216632	0.0997286529356569	ATXN1	6310	ataxin 1 [Source:HGNC Symbol;Acc:HGNC:10548]
-0.33056600150289	0.0044207090232699	EXT1	2131	exostosin glycosyltransferase 1 [Source:HGNC Symbol;Acc:HGNC:3512]
-0.330157481646784	0.127030105131118	CTSS	1520	cathepsin S [Source:HGNC Symbol;Acc:HGNC:2545]
-0.329966205603194	0.0253793144618135	PPP1CB	5500	protein phosphatase 1, catalytic subunit, beta isozyme [Source:HGNC Symbol;Acc:HGNC:9282]
-0.329948507476924	0.175878855697475	MAGT1	84061	magnesium transporter 1 [Source:HGNC Symbol;Acc:HGNC:28880]
-0.329571658173171	0.171832674042062	ZBTB7C	201501	zinc finger and BTB domain containing 7C [Source:HGNC Symbol;Acc:HGNC:31700]
-0.329492060522584	0.0847810045448699	HCST	10870	hematopoietic cell signal transducer [Source:HGNC Symbol;Acc:HGNC:16977]
-0.329359715838593	0.199361586969164	RNF144A	9781	ring finger protein 144A [Source:HGNC Symbol;Acc:HGNC:20457]
-0.329104529623201	0.0859922120144867	AFTPH	54812	aftiphilin [Source:HGNC Symbol;Acc:HGNC:25951]
-0.32899438923183	0.0975760115093383	ARHGAP44	9912	Rho GTPase activating protein 44 [Source:HGNC Symbol;Acc:HGNC:29096]
-0.328932947087864	0.0275739564751897	SLC27A3	11000	solute carrier family 27 (fatty acid transporter), member 3 [Source:HGNC Symbol;Acc:HGNC:10997]
-0.328747789137263	0.159430938982804	ARHGAP28	79822	Rho GTPase activating protein 28 [Source:HGNC Symbol;Acc:HGNC:25509]
-0.328177772299038	0.000245268345691325	MRPS5	64969	mitochondrial ribosomal protein S5 [Source:HGNC Symbol;Acc:HGNC:14498]
-0.328120043294733	0.18203858461143	FLRT2	23768	fibronectin leucine rich transmembrane protein 2 [Source:HGNC Symbol;Acc:HGNC:3761]
-0.327927881394056	0.182146591642805	NYNRIN	57523	NYN domain and retroviral integrase containing [Source:HGNC Symbol;Acc:HGNC:20165]
-0.32731682748142	0.103287432567685	COMMD9	29099	COMM domain containing 9 [Source:HGNC Symbol;Acc:HGNC:25014]
-0.326057002745207	0.0669558783406567	TMCO1	54499	transmembrane and coiled-coil domains 1 [Source:HGNC Symbol;Acc:HGNC:18188]
-0.325907917607547	0.0959392035015231	TAB2	23118	TGF-beta activated kinase 1/MAP3K7 binding protein 2 [Source:HGNC Symbol;Acc:HGNC:17075]
-0.325879179681299	0.135046513845156	MYCT1	80177	myc target 1 [Source:HGNC Symbol;Acc:HGNC:23172]
-0.325847038581978	0.0669274073812116	KIAA0930	23313	KIAA0930 [Source:HGNC Symbol;Acc:HGNC:1314]
-0.325628093205803	0.111516573402836	AMOTL1	154810	angiomotin like 1 [Source:HGNC Symbol;Acc:HGNC:17811]
-0.325040640257173	0.0911279445564213	CXCL12	6387	chemokine (C-X-C motif) ligand 12 [Source:HGNC Symbol;Acc:HGNC:10672]
-0.324586507328474	0.182798454638774	LRRC8C	84230	leucine rich repeat containing 8 family, member C [Source:HGNC Symbol;Acc:HGNC:25075]
-0.324507455711032	0.138055799311409	MYADM	91663	myeloid-associated differentiation marker [Source:HGNC Symbol;Acc:HGNC:7544]
-0.324403000145112	0.000735436097830859	MAML2	84441	mastermind-like 2 (Drosophila) [Source:HGNC Symbol;Acc:HGNC:16259]
-0.324351968511962	0.142980825332049	ATF7IP	55729	activating transcription factor 7 interacting protein [Source:HGNC Symbol;Acc:HGNC:20092]
-0.323998567557273	0.0913877880708276	FAM46A	55603	family with sequence similarity 46, member A [Source:HGNC Symbol;Acc:HGNC:18345]
-0.323674594000028	0.145076279840575	MPZL3	196264	myelin protein zero-like 3 [Source:HGNC Symbol;Acc:HGNC:27279]
-0.323542159615929	0.0705508738729483	CA1	759	carbonic anhydrase I [Source:HGNC Symbol;Acc:HGNC:1368]
-0.323443646559403	0.100640143056966	ZNF143	7702	zinc finger protein 143 [Source:HGNC Symbol;Acc:HGNC:12928]
-0.323396904714348	0.0451200865370588	VMP1	81671	vacuole membrane protein 1 [Source:HGNC Symbol;Acc:HGNC:29559]
-0.32339505089464	0.0945611393440802	SLC4A4	8671	solute carrier family 4 (sodium bicarbonate cotransporter), member 4 [Source:HGNC Symbol;Acc:HGNC:11030]
-0.323377765560456	0.0703808436863688	ESYT1	23344	extended synaptotagmin-like protein 1 [Source:HGNC Symbol;Acc:HGNC:29534]
-0.323266679473507	0.00706956412763892	TAB3	257397	TGF-beta activated kinase 1/MAP3K7 binding protein 3 [Source:HGNC Symbol;Acc:HGNC:30681]
-0.323159746992324	0.133876034091894	PPM1A	5494	protein phosphatase, Mg2+/Mn2+ dependent, 1A [Source:HGNC Symbol;Acc:HGNC:9275]
-0.323136935989148	0.0724664880559208	CRIP2	1397	cysteine-rich protein 2 [Source:HGNC Symbol;Acc:HGNC:2361]
-0.322941111657736	0.158728141651059	SERPING1	710	serpin peptidase inhibitor, clade G (C1 inhibitor), member 1 [Source:HGNC Symbol;Acc:HGNC:1228]
-0.322299574076771	0.126344114665935	GSTA2	2939	glutathione S-transferase alpha 2 [Source:HGNC Symbol;Acc:HGNC:4627]
-0.322067156906041	0.145407466277126	TOR1AIP2	163590	torsin A interacting protein 2 [Source:HGNC Symbol;Acc:HGNC:24055]
-0.321830643356888	0.0572762042244403	C10orf12	26148	chromosome 10 open reading frame 12 [Source:HGNC Symbol;Acc:HGNC:23420]
-0.321754451545414	0.0596603250939725	ZNF431	170959	zinc finger protein 431 [Source:HGNC Symbol;Acc:HGNC:20809]
-0.321672708497448	0.0689998650143721	RPA2	6118	replication protein A2, 32kDa [Source:HGNC Symbol;Acc:HGNC:10290]
-0.321604757044658	0.117887961963858	FAM89B	23625	family with sequence similarity 89, member B [Source:HGNC Symbol;Acc:HGNC:16708]
-0.321543169346369	0.166588927104771	TBX10	347853	T-box 10 [Source:HGNC Symbol;Acc:HGNC:11593]
-0.32148132044946	0.0764980304252427	NBR1	4077	neighbor of BRCA1 gene 1 [Source:HGNC Symbol;Acc:HGNC:6746]
-0.321338486090338	0.0220056961246116		84717	Hepatoma-derived growth factor-related protein 2 [Source:UniProtKB/Swiss-Prot;Acc:Q7Z4V5]
-0.321329398945402	0.0447285467520527	MGAT4A	11320	mannosyl (alpha-1,3-)-glycoprotein beta-1,4-N-acetylglucosaminyltransferase, isozyme A [Source:HGNC Symbol;Acc:HGNC:7047]
-0.321205099653207	0.0400308813711287	ENG	2022	endoglin [Source:HGNC Symbol;Acc:HGNC:3349]
-0.320829077203829	0.0634312755723665	C7orf31	136895	chromosome 7 open reading frame 31 [Source:HGNC Symbol;Acc:HGNC:21722]
-0.32058916152429	0.153699408679599	HSPA4	3308	heat shock 70kDa protein 4 [Source:HGNC Symbol;Acc:HGNC:5237]
-0.320481954842948	0.0371194775424433	FUCA1	2517	fucosidase, alpha-L- 1, tissue [Source:HGNC Symbol;Acc:HGNC:4006]
-0.320472708965785	0.143860856934245	CREBRF	153222	CREB3 regulatory factor [Source:HGNC Symbol;Acc:HGNC:24050]
-0.320415753531178	0.157232746962612	GPD1L	23171	glycerol-3-phosphate dehydrogenase 1-like [Source:HGNC Symbol;Acc:HGNC:28956]
-0.320207832453455	0.179503573862894	GK5	256356	glycerol kinase 5 (putative) [Source:HGNC Symbol;Acc:HGNC:28635]
-0.32016920880062	0.0256882372467711	PRKACB	5567	protein kinase, cAMP-dependent, catalytic, beta [Source:HGNC Symbol;Acc:HGNC:9381]
-0.320149005395561	0.0362529883227952	MRPS6	64968	mitochondrial ribosomal protein S6 [Source:HGNC Symbol;Acc:HGNC:14051]
-0.319843781963647	0.0429157202260584	MSRA	4482	methionine sulfoxide reductase A [Source:HGNC Symbol;Acc:HGNC:7377]
-0.319793911689809	0.149549122875432	KANK2	25959	KN motif and ankyrin repeat domains 2 [Source:HGNC Symbol;Acc:HGNC:29300]
-0.319734405372932	0.136474642211448	LMBRD1	55788	LMBR1 domain containing 1 [Source:HGNC Symbol;Acc:HGNC:23038]
-0.319699944346776	0.06273462877486	SEC24A	10802	SEC24 family member A [Source:HGNC Symbol;Acc:HGNC:10703]
-0.319367901944829	0.0033525042193796	RAPGEF4	11069	Rap guanine nucleotide exchange factor (GEF) 4 [Source:HGNC Symbol;Acc:HGNC:16626]
-0.3191257969421	0.115152763449619	IFT22	64792	intraflagellar transport 22 [Source:HGNC Symbol;Acc:HGNC:21895]
-0.318670318075083	0.18341053112585		79154	Dehydrogenase/reductase SDR family member 11 [Source:UniProtKB/Swiss-Prot;Acc:Q6UWP2]
-0.31861435037134	0.122510615100074	TMEM184C	55751	transmembrane protein 184C [Source:HGNC Symbol;Acc:HGNC:25587]
-0.318554470896118	0.0330059475124683	TTC9C	283237	tetratricopeptide repeat domain 9C [Source:HGNC Symbol;Acc:HGNC:28432]
-0.318489307748266	0.17892469197301	CCT6A	908	chaperonin containing TCP1, subunit 6A (zeta 1) [Source:HGNC Symbol;Acc:HGNC:1620]
-0.318439108776023	0.107743103207206	PLGRKT	55848	plasminogen receptor, C-terminal lysine transmembrane protein [Source:HGNC Symbol;Acc:HGNC:23633]
-0.318159248495221	0.0535366317518422	FRMD6	122786	FERM domain containing 6 [Source:HGNC Symbol;Acc:HGNC:19839]
-0.317821409664032	0.0888713863469321	CD81	975	CD81 molecule [Source:HGNC Symbol;Acc:HGNC:1701]
-0.317771992303046	0.0399748739656223	PARM1	25849	prostate androgen-regulated mucin-like protein 1 [Source:HGNC Symbol;Acc:HGNC:24536]
-0.317695204955374	0.199781768631722	KLHDC2	23588	kelch domain containing 2 [Source:HGNC Symbol;Acc:HGNC:20231]
-0.317401410449975	0.0571896223991301	FAM134B	54463	family with sequence similarity 134, member B [Source:HGNC Symbol;Acc:HGNC:25964]
-0.317355997692635	0.0894710454782229	SGCB	6443	sarcoglycan, beta (43kDa dystrophin-associated glycoprotein) [Source:HGNC Symbol;Acc:HGNC:10806]
-0.317311505786849	0.162701162484677	PDGFRB	5159	platelet-derived growth factor receptor, beta polypeptide [Source:HGNC Symbol;Acc:HGNC:8804]
-0.316956023104398	0.0845409917008755	ZNF678	339500	zinc finger protein 678 [Source:HGNC Symbol;Acc:HGNC:28652]
-0.316755242362225	0.0908345616167326	BTBD7	55727	BTB (POZ) domain containing 7 [Source:HGNC Symbol;Acc:HGNC:18269]
-0.316678891295525	0.0995985002462719	USP39	10713	ubiquitin specific peptidase 39 [Source:HGNC Symbol;Acc:HGNC:20071]
-0.316508007728857	0.0857460551268379	TSEN34	79042	TSEN34 tRNA splicing endonuclease subunit [Source:HGNC Symbol;Acc:HGNC:15506]
-0.316324088619872	0.174469298495466	MUC3A	4584	mucin 3A, cell surface associated [Source:HGNC Symbol;Acc:HGNC:7513]
-0.316002971830187	0.182455493524838	TMEM138	51524	transmembrane protein 138 [Source:HGNC Symbol;Acc:HGNC:26944]
-0.31584937411497	0.169253911605661	FAM117B	150864	family with sequence similarity 117, member B [Source:HGNC Symbol;Acc:HGNC:14440]
-0.3156438864858	0.0653887428237257	TOLLIP	54472	toll interacting protein [Source:HGNC Symbol;Acc:HGNC:16476]
-0.315633909737941	0.0707497328996531	DNAAF2	55172	dynein, axonemal, assembly factor 2 [Source:HGNC Symbol;Acc:HGNC:20188]
-0.315540653306396	0.0238659902926104	EIF4EBP2	1979	eukaryotic translation initiation factor 4E binding protein 2 [Source:HGNC Symbol;Acc:HGNC:3289]
-0.314752041294621	0.0692164619895903	ZNF227	7770	zinc finger protein 227 [Source:HGNC Symbol;Acc:HGNC:13020]
-0.31468182373148	0.0594885535314587	CASP6	839	caspase 6, apoptosis-related cysteine peptidase [Source:HGNC Symbol;Acc:HGNC:1507]
-0.314556286829461	0.163564556999903	CLIP2	7461	CAP-GLY domain containing linker protein 2 [Source:HGNC Symbol;Acc:HGNC:2586]
-0.314279449285684	0.130458905612497	TMEM218	219854	transmembrane protein 218 [Source:HGNC Symbol;Acc:HGNC:27344]
-0.314204354213376	0.0894239466757697	LDOC1L	84247	leucine zipper, down-regulated in cancer 1-like [Source:HGNC Symbol;Acc:HGNC:13343]
-0.313963556702407	0.117884929343204	SPA17	53340	sperm autoantigenic protein 17 [Source:HGNC Symbol;Acc:HGNC:11210]
-0.31379448645436	0.13361810209077	SYTL2	54843	synaptotagmin-like 2 [Source:HGNC Symbol;Acc:HGNC:15585]
-0.313581773123409	0.149198269164159	DUSP16	80824	dual specificity phosphatase 16 [Source:HGNC Symbol;Acc:HGNC:17909]
-0.313495254843819	0.0709365956135361	RPP14	11102	ribonuclease P/MRP 14kDa subunit [Source:HGNC Symbol;Acc:HGNC:30327]
-0.313275864532175	0.0703015737400586	FAS	355	Fas cell surface death receptor [Source:HGNC Symbol;Acc:HGNC:11920]

-0.313172661715138	0.04594677399059	ARHGEF12	23365	Rho guanine nucleotide exchange factor (GEF) 12 [Source:HGNC Symbol;Acc:HGNC:14193]
-0.312870839306247	0.190706457538593	RAB4A	5867	RAB4A, member RAS oncogene family [Source:HGNC Symbol;Acc:HGNC:9781]
-0.312832361075117	0.102685523035771	RITA1	84934	RBPJ interacting and tubulin associated 1 [Source:HGNC Symbol;Acc:HGNC:25925]
-0.312745552322545	0.124184568865159	FAM168B	130074	family with sequence similarity 168, member B [Source:HGNC Symbol;Acc:HGNC:27016]
-0.312474787758009	0.0195974411580732	ZNF148	7707	zinc finger protein 148 [Source:HGNC Symbol;Acc:HGNC:12933]
-0.312245612828767	0.172735147412914	GATSL2	729438	GATS protein-like 2 [Source:HGNC Symbol;Acc:HGNC:37073]
-0.312158970496008	0.197360801921158	DAB2	1601	Dab, mitogen-responsive phosphoprotein, homolog 2 (Drosophila) [Source:HGNC Symbol;Acc:HGNC:2662]
-0.3120313352396	0.00217579117080912	KIAA1191	57179	KIAA1191 [Source:HGNC Symbol;Acc:HGNC:29209]
-0.311405283114018	0.0431730549611791	MRPS34	65993	mitochondrial ribosomal protein S34 [Source:HGNC Symbol;Acc:HGNC:16618]
-0.311334621349429	0.0472035473300753	CYB5A	1528	cytochrome b5 type A (microsomal) [Source:HGNC Symbol;Acc:HGNC:2570]
-0.311270728856389	0.180893273838066	SLIT3	6586	slit homolog 3 (Drosophila) [Source:HGNC Symbol;Acc:HGNC:11087]
-0.31117690278076	0.0921395897406881	LPCAT3	10162	lysophosphatidylcholine acyltransferase 3 [Source:HGNC Symbol;Acc:HGNC:30244]
-0.311025591620264	0.0322489444804547	TBC1D9	23158	TBC1 domain family, member 9 (with GRAM domain) [Source:HGNC Symbol;Acc:HGNC:21710]
-0.311021374329735	0.0276162767312498	RYBP	23429	RING1 and YY1 binding protein [Source:HGNC Symbol;Acc:HGNC:10480]
-0.3108333550838	0.0608999341220522	TOMM40L	84134	translocase of outer mitochondrial membrane 40 homolog (yeast)-like [Source:HGNC Symbol;Acc:HGNC:25756]
-0.310593192458799	0.00692980883456656	SCARB2	950	scavenger receptor class B, member 2 [Source:HGNC Symbol;Acc:HGNC:1665]
-0.310539618881124	0.0585623355929705	TMEM60	85025	transmembrane protein 60 [Source:HGNC Symbol;Acc:HGNC:21754]
-0.310522059705789	0.191299522452846	RAB15	376267	RAB15, member RAS oncogene family [Source:HGNC Symbol;Acc:HGNC:20150]
-0.309651989792304	0.0531767195626979	CD9	928	CD9 molecule [Source:HGNC Symbol;Acc:HGNC:1709]
-0.309094799059344	0.127101799626965	PTGES3	10728	prostaglandin E synthase 3 (cytosolic) [Source:HGNC Symbol;Acc:HGNC:16049]
-0.30909001711673	0.0293589304554043	MPZL1	9019	myelin protein zero-like 1 [Source:HGNC Symbol;Acc:HGNC:7226]
-0.308858356578304	0.0227927088813606	MLLT4	4301	myeloid/lymphoid or mixed-lineage leukemia (trithorax homolog, Drosophila); translocated to, 4 [Source:HGNC Symbol;Acc:HGNC:7137]
-0.308631953981352	0.00779476129231589	R3HCC1	203069	R3H domain and coiled-coil containing 1 [Source:HGNC Symbol;Acc:HGNC:27329]
-0.307616911858755	0.0328056822483744	NPC2	10577	Niemann-Pick disease, type C2 [Source:HGNC Symbol;Acc:HGNC:14537]
-0.307574515617736	0.142879809916505	FAM134C	162427	family with sequence similarity 134, member C [Source:HGNC Symbol;Acc:HGNC:27258]
-0.307458090112993	0.0469769525481774	GPBP1L1	60313	GC-rich promoter binding protein 1-like 1 [Source:HGNC Symbol;Acc:HGNC:28843]
-0.307214661223884	0.188819866190471	RAB27A	5873	RAB27A, member RAS oncogene family [Source:HGNC Symbol;Acc:HGNC:9766]
-0.307200527010668	0.155700770634266	ZER1	10444	zyg-11 related, cell cycle regulator [Source:HGNC Symbol;Acc:HGNC:30960]
-0.307082631001944	0.141805850287845	AOAH	313	acyloxyacyl hydrolase (neutrophil) [Source:HGNC Symbol;Acc:HGNC:548]
-0.307019576771645	0.0295575514077609	DDR2	4921	discoidin domain receptor tyrosine kinase 2 [Source:HGNC Symbol;Acc:HGNC:2731]
-0.306986552859795	0.0948209599155455	CPM	1368	carboxypeptidase M [Source:HGNC Symbol;Acc:HGNC:2311]
-0.306967107574472	0.138912548858056	KDSR	2531	3-ketodihydrosphingosine reductase [Source:HGNC Symbol;Acc:HGNC:4021]
-0.306898757865401	0.16098818151472	TMED9	54732	transmembrane emp24 protein transport domain containing 9 [Source:HGNC Symbol;Acc:HGNC:24878]
-0.30670177040055	0.178979993469998	PRKCDBP	112464	protein kinase C, delta binding protein [Source:HGNC Symbol;Acc:HGNC:9400]
-0.306634990270259	0.169375946261759	FABP1	2168	fatty acid binding protein 1, liver [Source:HGNC Symbol;Acc:HGNC:3555]
-0.306547928204366	0.188143080300046	MSN	4478	moesin [Source:HGNC Symbol;Acc:HGNC:7373]
-0.306058186438849	0.0584473881569491	TRIM13	10206	tripartite motif containing 13 [Source:HGNC Symbol;Acc:HGNC:9976]
-0.30604951689336	0.147015843208059	FNTA	2339	farnesyltransferase, CAAX box, alpha [Source:HGNC Symbol;Acc:HGNC:3782]
-0.306045450089142	0.113318117086375	FAM134A	79137	family with sequence similarity 134, member A [Source:HGNC Symbol;Acc:HGNC:28450]
-0.305848522679948	0.183078073157783	KDM4A	9682	lysine (K)-specific demethylase 4A [Source:HGNC Symbol;Acc:HGNC:22978]
-0.305749795811315	0.0286823453224654	MAPK9	5601	mitogen-activated protein kinase 9 [Source:HGNC Symbol;Acc:HGNC:6886]
-0.305528676418204	0.150981973814594	RLF	6018	rearranged L-myc fusion [Source:HGNC Symbol;Acc:HGNC:10025]
-0.305218527029233	0.163145661711153	PIGN	23556	phosphatidylinositol glycan anchor biosynthesis, class N [Source:HGNC Symbol;Acc:HGNC:8967]
-0.304831111737214	0.145294659883494	SPICE1	152185	spindle and centriole associated protein 1 [Source:HGNC Symbol;Acc:HGNC:25083]
-0.30469613766682	0.123660754664476	LGMN	5641	legumain [Source:HGNC Symbol;Acc:HGNC:9472]
-0.304056096772185	0.13435767073967	GNPTAB	79158	N-acetylglucosamine-1-phosphate transferase, alpha and beta subunits [Source:HGNC Symbol;Acc:HGNC:29670]
-0.303458880296545	0.136300888206872		51765	Serine/threonine-protein kinase MST4 [Source:UniProtKB/Swiss-Prot;Acc:Q9P289]
-0.303365702254827	0.115278042331782	TMEM246	84302	transmembrane protein 246 [Source:HGNC Symbol;Acc:HGNC:28180]
-0.303321226952424	0.0361438761778693	STX6	10228	syntaxin 6 [Source:HGNC Symbol;Acc:HGNC:11441]
-0.303278219165328	0.19703387475002	FUT11	170384	fucosyltransferase 11 (alpha (1,3) fucosyltransferase) [Source:HGNC Symbol;Acc:HGNC:19233]
-0.303138424465705	0.0432646534961756	CTDSPL	10217	CTD (carboxy-terminal domain, RNA polymerase II, polypeptide A) small phosphatase-like [Source:HGNC Symbol;Acc:HGNC:16890]
-0.302908586132869	0.0474536129495539	SNX4	8723	sorting nexin 4 [Source:HGNC Symbol;Acc:HGNC:11175]
-0.30279010610496	0.0442880302053667	MRPL41	64975	mitochondrial ribosomal protein L41 [Source:HGNC Symbol;Acc:HGNC:14492]
-0.302685999306525	0.143816819192331	TMEM168	64418	transmembrane protein 168 [Source:HGNC Symbol;Acc:HGNC:25826]
-0.302651797343615	0.0297110771623269	DKK3	27122	dickkopf WNT signaling pathway inhibitor 3 [Source:HGNC Symbol;Acc:HGNC:2893]
-0.302622018392953	0.101031126955823	PTTG1IP	754	pituitary tumor-transforming 1 interacting protein [Source:HGNC Symbol;Acc:HGNC:13524]
-0.302469775553012	0.135670217232641	CAMK2G	818	calcium/calmodulin-dependent protein kinase II gamma [Source:HGNC Symbol;Acc:HGNC:1463]
-0.302422458126201	0.139952993679985	GPX2	2877	glutathione peroxidase 2 (gastrointestinal) [Source:HGNC Symbol;Acc:HGNC:4554]
-0.30237702552064	0.0834439806053544	GOLPH3L	55204	golgi phosphoprotein 3-like [Source:HGNC Symbol;Acc:HGNC:24882]
-0.302041021999365	0.119930081160293	AP5M1	55745	adaptor-related protein complex 5, mu 1 subunit [Source:HGNC Symbol;Acc:HGNC:20192]
-0.301907565083603	0.021373447949077	ZNF483	158399	zinc finger protein 483 [Source:HGNC Symbol;Acc:HGNC:23384]
-0.301708793505409	0.163638413060219	WDR12	55759	WD repeat domain 12 [Source:HGNC Symbol;Acc:HGNC:14098]
-0.301683259192905	0.0483808907881213	SLCO2B1	11309	solute carrier organic anion transporter family, member 2B1 [Source:HGNC Symbol;Acc:HGNC:10962]
-0.301469977366301	0.0613676041610118	SH3BGRL2	83699	SH3 domain binding glutamate-rich protein like 2 [Source:HGNC Symbol;Acc:HGNC:15567]
-0.301373357422115	0.0608614489520079	GNG2	54331	guanine nucleotide binding protein (G protein), gamma 2 [Source:HGNC Symbol;Acc:HGNC:4404]
-0.301343850894032	0.144447386471808	ISLR	3671	immunoglobulin superfamily containing leucine-rich repeat [Source:HGNC Symbol;Acc:HGNC:6133]
-0.301264112705996	0.0835638267837122	MRPL35	51318	mitochondrial ribosomal protein L35 [Source:HGNC Symbol;Acc:HGNC:14489]
-0.301219709350149	0.0157224604248832	SUCLG1	8802	succinate-CoA ligase, alpha subunit [Source:HGNC Symbol;Acc:HGNC:11449]
-0.300449899213398	0.0994351898087654	ALDH9A1	223	aldehyde dehydrogenase 9 family, member A1 [Source:HGNC Symbol;Acc:HGNC:412]
-0.300445217957067	0.164246208076469	INPP5A	3632	inositol polyphosphate-5-phosphatase, 40kDa [Source:HGNC Symbol;Acc:HGNC:6076]
-0.300429544337869	0.142309605165351	CLTB	1212	clathrin, light chain B [Source:HGNC Symbol;Acc:HGNC:2091]
-0.29996730728029	0.0359951540025505	CTSB	1508	cathepsin B [Source:HGNC Symbol;Acc:HGNC:2527]
-0.299868429895443	0.165366993077923	ZNF703	80139	zinc finger protein 703 [Source:HGNC Symbol;Acc:HGNC:25883]
-0.29973812873964	0.0789018986518216	TMEM45B	120224	transmembrane protein 45B [Source:HGNC Symbol;Acc:HGNC:25194]
-0.299649941034484	0.0945965816519388	NMT2	9397	N-myristoyltransferase 2 [Source:HGNC Symbol;Acc:HGNC:7858]
-0.299549797381179	0.0958740534082428	C12orf75	387882	chromosome 12 open reading frame 75 [Source:HGNC Symbol;Acc:HGNC:35164]
-0.299427206658588	0.117253822756406	CAMLG	819	calcium modulating ligand [Source:HGNC Symbol;Acc:HGNC:1471]
-0.299386472770898	0.153558570599914	LAMC1	3915	laminin, gamma 1 (formerly LAMB2) [Source:HGNC Symbol;Acc:HGNC:6492]
-0.299229228089381	0.0375977695052265	ERGIC3	51614	ERGIC and golgi 3 [Source:HGNC Symbol;Acc:HGNC:15927]
-0.298806347395492	0.12217957607997	SPATA2	9825	spermatogenesis associated 2 [Source:HGNC Symbol;Acc:HGNC:14681]
-0.298605417156719	0.0937597943251703	FAM217B	63939	family with sequence similarity 217, member B [Source:HGNC Symbol;Acc:HGNC:16170]
-0.29819387727215	0.171475796139145	PRKX	5613	protein kinase, X-linked [Source:HGNC Symbol;Acc:HGNC:9441]
-0.298107280322448	0.146986828165783	STARD3	10948	StAR-related lipid transfer (START) domain containing 3 [Source:HGNC Symbol;Acc:HGNC:17579]
-0.298085673012005	0.17883129188599	RPN1	6184	ribophorin I [Source:HGNC Symbol;Acc:HGNC:10381]
-0.297957201318597	0.130898485779958	WDTC1	23038	WD and tetratricopeptide repeats 1 [Source:HGNC Symbol;Acc:HGNC:29175]
-0.297704695023919	0.0942100661732432	GSK3B	2932	glycogen synthase kinase 3 beta [Source:HGNC Symbol;Acc:HGNC:4617]
-0.297665993509169	0.171162121926831	B4GALT4	8702	UDP-Gal:betaGlcNAc beta 1,4- galactosyltransferase, polypeptide 4 [Source:HGNC Symbol;Acc:HGNC:927]
-0.297540507533092	0.157087517858208	AP5B1	91056	adaptor-related protein complex 5, beta 1 subunit [Source:HGNC Symbol;Acc:HGNC:25104]
-0.29750196318167	0.192026845351926	P4HA2	8974	prolyl 4-hydroxylase, alpha polypeptide II [Source:HGNC Symbol;Acc:HGNC:8547]
-0.297487244301577	0.0277675423175676	ACTR8	93973	ARP8 actin-related protein 8 homolog (yeast) [Source:HGNC Symbol;Acc:HGNC:14672]
-0.297408728850829	0.14822018470885	TPP1	1200	tripeptidyl peptidase I [Source:HGNC Symbol;Acc:HGNC:2073]
-0.297207100992541	0.0862200880964571	FYCO1	79443	FYVE and coiled-coil domain containing 1 [Source:HGNC Symbol;Acc:HGNC:14673]
-0.297198027251483	0.170735932936536	ABCA8	10351	ATP-binding cassette, sub-family A (ABC1), member 8 [Source:HGNC Symbol;Acc:HGNC:38]
-0.297130917995433	0.146303310437188	COL14A1	7373	collagen, type XIV, alpha 1 [Source:HGNC Symbol;Acc:HGNC:2191]
-0.297076185766126	0.0203594714433236	GSTM3	2947	glutathione S-transferase mu 3 (brain) [Source:HGNC Symbol;Acc:HGNC:4635]
-0.296443602478195	0.0308831680561739	KCTD2	23510	potassium channel tetramerization domain containing 2 [Source:HGNC Symbol;Acc:HGNC:21294]
-0.296151923645635	0.127991048159867	SPPL3	121665	signal peptide peptidase like 3 [Source:HGNC Symbol;Acc:HGNC:30424]
-0.295929668832604	0.166184206575163	MMP14	4323	matrix metallopeptidase 14 (membrane-inserted) [Source:HGNC Symbol;Acc:HGNC:7160]
-0.295807421068624	0.187404744214283	ADAMDEC1	27299	ADAM-like, decysin 1 [Source:HGNC Symbol;Acc:HGNC:16299]
-0.295800166814819	0.00245772350155302	FAM162A	26355	family with sequence similarity 162, member A [Source:HGNC Symbol;Acc:HGNC:17865]
-0.295712320681966	0.132912972717636	ATG9A	79065	autophagy related 9A [Source:HGNC Symbol;Acc:HGNC:22408]
-0.295636622306576	0.167673550390787	ENPP2	5168	ectonucleotide pyrophosphatase/phosphodiesterase 2 [Source:HGNC Symbol;Acc:HGNC:3357]
-0.29554941019171	0.108691007244107	PCTP	58488	phosphatidylcholine transfer protein [Source:HGNC Symbol;Acc:HGNC:8752]
-0.295168966853395	0.169724343194728	BTBD3	22903	BTB (POZ) domain containing 3 [Source:HGNC Symbol;Acc:HGNC:15854]
-0.295160478870081	0.141199710171757	ZNF274	10782	zinc finger protein 274 [Source:HGNC Symbol;Acc:HGNC:13068]
-0.295156902952484	0.184533570214036	RILPL2	196383	Rab interacting lysosomal protein-like 2 [Source:HGNC Symbol;Acc:HGNC:28787]
-0.294865786575882	0.156788845874813	GALM	130589	galactose mutarotase (aldose 1-epimerase) [Source:HGNC Symbol;Acc:HGNC:24063]
-0.294768124211886	0.0586703473211387	ABCF3	55324	ATP-binding cassette, sub-family F (GCN20), member 3 [Source:HGNC Symbol;Acc:HGNC:72]
-0.294764333216034	0.183678562607323	SPINT2	10653	serine peptidase inhibitor, Kunitz type, 2 [Source:HGNC Symbol;Acc:HGNC:11247]
-0.294357210880216	0.146711799300955	USP20	10868	ubiquitin specific peptidase 20 [Source:HGNC Symbol;Acc:HGNC:12619]
-0.294326541301756	0.180207539128194	TMEM242	729515	transmembrane protein 242 [Source:HGNC Symbol;Acc:HGNC:17206]
-0.293937884599116	0.143531201608515	ADNP	23394	activity-dependent neuroprotector homeobox [Source:HGNC Symbol;Acc:HGNC:15766]
-0.293693481188645	0.0991377878604763	ADCY2	108	adenylate cyclase 2 (brain) [Source:HGNC Symbol;Acc:HGNC:233]
-0.293326685395575	0.0803147289696425	TATDN2	9797	TatD DNase domain containing 2 [Source:HGNC Symbol;Acc:HGNC:28988]
-0.292970223161809	0.0934624745932898	ITGB5	3693	integrin, beta 5 [Source:HGNC Symbol;Acc:HGNC:6160]
-0.292850124565536	0.0895754676677485	FAM227B	196951	family with sequence similarity 227, member B [Source:HGNC Symbol;Acc:HGNC:26543]
-0.292800891916903	0.173786661596809	SDC2	6383	syndecan 2 [Source:HGNC Symbol;Acc:HGNC:10659]
-0.29257603070674	0.193999964607504	LAMA2	3908	laminin, alpha 2 [Source:HGNC Symbol;Acc:HGNC:6482]
-0.292390037664229	0.161452839074639	HEG1	57493	heart development protein with EGF-like domains 1 [Source:HGNC Symbol;Acc:HGNC:29227]
-0.292070162290804	0.172234512023686	ATP6AP2	10159	ATPase, H+ transporting, lysosomal accessory protein 2 [Source:HGNC Symbol;Acc:HGNC:18305]
-0.291901973803493	0.171427587498093		26137	
-0.291313631716383	0.0922575890265927	NEU1	4758	sialidase 1 (lysosomal sialidase) [Source:HGNC Symbol;Acc:HGNC:7758]
-0.291253703968566	0.0673467866639543	LAMTOR1	55004	late endosomal/lysosomal adaptor, MAPK and MTOR activator 1 [Source:HGNC Symbol;Acc:HGNC:26068]
-0.290808807933535	0.0811759679611283	COPS5	10987	COP9 signalosome subunit 5 [Source:HGNC Symbol;Acc:HGNC:2240]
-0.290715223358332	0.168945368210928	PIP5K1B	8395	phosphatidylinositol-4-phosphate 5-kinase, type I, beta [Source:HGNC Symbol;Acc:HGNC:8995]
-0.290705481219434	0.0575442579427832	SPR	6697	sepiapterin reductase (7,8-dihydrobiopterin:NADP+ oxidoreductase) [Source:HGNC Symbol;Acc:HGNC:11257]
-0.290661420123482	0.0428009467857858	C7orf49	78996	chromosome 7 open reading frame 49 [Source:HGNC Symbol;Acc:HGNC:22432]
-0.290647848089648	0.0946862654774967	MFSD6	54842	major facilitator superfamily domain containing 6 [Source:HGNC Symbol;Acc:HGNC:24711]
-0.290143731085303	0.00593116619174848	COMMD5	101928879	COMM domain containing 5 [Source:HGNC Symbol;Acc:HGNC:17902]
-0.289877001131288	0.115008956945401	ZHX1	11244	zinc fingers and homeoboxes 1 [Source:HGNC Symbol;Acc:HGNC:12871]
-0.28986520375893	0.0576300875201528	SBF2	81846	SET binding factor 2 [Source:HGNC Symbol;Acc:HGNC:2135]
-0.289746628825166	0.166658105508554	EDEM3	80267	ER degradation enhancer, mannosidase alpha-like 3 [Source:HGNC Symbol;Acc:HGNC:16787]
-0.289550575150259	0.110934833489303	UCP2	7351	uncoupling protein 2 (mitochondrial, proton carrier) [Source:HGNC Symbol;Acc:HGNC:12518]
-0.289472206877975	0.12136107255549	NRIP1	8204	nuclear receptor interacting protein 1 [Source:HGNC Symbol;Acc:HGNC:8001]
-0.289214363127797	0.108269361737213	SDC3	9672	syndecan 3 [Source:HGNC Symbol;Acc:HGNC:10660]
-0.289150513875004	0.106953647230268	PMS2	5395	PMS2 postmeiotic segregation increased 2 (S. cerevisiae) [Source:HGNC Symbol;Acc:HGNC:9122]
-0.289119393435303	0.117672803306427	SORT1	6272	sortilin 1 [Source:HGNC Symbol;Acc:HGNC:11186]
-0.288953011596123	0.12655412705594	ANK3	288	ankyrin 3, node of Ranvier (ankyrin G) [Source:HGNC Symbol;Acc:HGNC:494]
-0.288950070146581	0.17573236041737	mars-08	220972	membrane-associated ring finger (C3HC4) 8, E3 ubiquitin protein ligase [Source:HGNC Symbol;Acc:HGNC:23356]
-0.288785044440133	0.108953454243018	IL34	146433	interleukin 34 [Source:HGNC Symbol;Acc:HGNC:28529]
-0.288542665188474	0.0313158332492511	SGPL1	8879	sphingosine-1-phosphate lyase 1 [Source:HGNC Symbol;Acc:HGNC:10817]

-0.28852558848541	0.0532007195145386	NCOA1	8648	nuclear receptor coactivator 1 [Source:HGNC Symbol;Acc:HGNC:7668]
-0.288468913750966	0.119865221411895	IL2RG	3561	interleukin 2 receptor, gamma [Source:HGNC Symbol;Acc:HGNC:6010]
-0.288037797994558	0.156294672532298	MICALCL	84953	MICAL C-terminal like [Source:HGNC Symbol;Acc:HGNC:25933]
-0.287874779974419	0.0792560931707534	CASC4	113201	cancer susceptibility candidate 4 [Source:HGNC Symbol;Acc:HGNC:24892]
-0.287729164721918	0.0339291334905517	LGR4	55366	leucine-rich repeat containing G protein-coupled receptor 4 [Source:HGNC Symbol;Acc:HGNC:13299]
-0.287662041081448	0.08298364044927	SEC24B	10427	SEC24 family member B [Source:HGNC Symbol;Acc:HGNC:10704]
-0.287385853482437	0.0409667100281804	TST	7263	thiosulfate sulfurtransferase (rhodanese) [Source:HGNC Symbol;Acc:HGNC:12388]
-0.287229441713255	0.0212531556087331	ASAH1	427	N-acylsphingosine amidohydrolase (acid ceramidase) 1 [Source:HGNC Symbol;Acc:HGNC:735]
-0.287052682277009	0.118120457427367	NAA35	60560	N(alpha)-acetyltransferase 35, NatC auxiliary subunit [Source:HGNC Symbol;Acc:HGNC:24340]
-0.286450700973139	0.12859928913045	PGM1	5236	phosphoglucomutase 1 [Source:HGNC Symbol;Acc:HGNC:8905]
-0.286343743406896	0.0886534788400263	PERP	64065	PERP, TP53 apoptosis effector [Source:HGNC Symbol;Acc:HGNC:17637]
-0.286033885049498	0.172578522630794		285150	uncharacterized LOC285150 [Source:EntrezGene;Acc:285150]
-0.285960880621409	0.183251139506879	UBL3	5412	ubiquitin-like 3 [Source:HGNC Symbol;Acc:HGNC:12504]
-0.285780850523702	0.054949605490219	C16orf72	29035	chromosome 16 open reading frame 72 [Source:HGNC Symbol;Acc:HGNC:30103]
-0.285362321001882	0.0954534502947237	IFT81	28981	intraflagellar transport 81 [Source:HGNC Symbol;Acc:HGNC:14313]
-0.285234924542485	0.0873374128498606	FAM105A	54491	family with sequence similarity 105, member A [Source:HGNC Symbol;Acc:HGNC:25629]
-0.28520295173808	0.0322877766546039	SH3D19	152503	SH3 domain containing 19 [Source:HGNC Symbol;Acc:HGNC:30418]
-0.285117778355729	0.0724613964729867	MARCKS	4082	myristoylated alanine-rich protein kinase C substrate [Source:HGNC Symbol;Acc:HGNC:6759]
-0.284535769578311	0.0569181214036861	TGM3	7053	transglutaminase 3 [Source:HGNC Symbol;Acc:HGNC:11779]
-0.284229235897146	0.106896882587643	CCM2	83605	cerebral cavernous malformation 2 [Source:HGNC Symbol;Acc:HGNC:21708]
-0.284100367076343	0.0212122367632806	SOD1	6647	superoxide dismutase 1, soluble [Source:HGNC Symbol;Acc:HGNC:11179]
-0.284075027215256	0.0825068038124332	C6orf89	221477	chromosome 6 open reading frame 89 [Source:HGNC Symbol;Acc:HGNC:21114]
-0.284069925760719	0.119667945889736	BOD1	91272	biorientation of chromosomes in cell division 1 [Source:HGNC Symbol;Acc:HGNC:25114]
-0.283623260253385	0.198873131579514	SCG5	6447	secretogranin V (7B2 protein) [Source:HGNC Symbol;Acc:HGNC:10816]
-0.283438544728564	0.157456318785883	SHROOM3	57619	shroom family member 3 [Source:HGNC Symbol;Acc:HGNC:30422]
-0.28328535920637	0.15411693988952	MEGF9	1955	multiple EGF-like-domains 9 [Source:HGNC Symbol;Acc:HGNC:3234]
-0.283145444817938	0.181284204177898	SLC9A9	285195	solute carrier family 9, subfamily A (NHE9, cation proton antiporter 9), member 9 [Source:HGNC Symbol;Acc:HGNC:20653]
-0.282560602909183	0.0235729906238529	ITM2B	9445	integral membrane protein 2B [Source:HGNC Symbol;Acc:HGNC:6174]
-0.282519816590564	0.0720734174608023	C17orf75	64149	chromosome 17 open reading frame 75 [Source:HGNC Symbol;Acc:HGNC:30173]
-0.282263164449797	0.186377270900081	C2orf69	205327	chromosome 2 open reading frame 69 [Source:HGNC Symbol;Acc:HGNC:26799]
-0.2820884117839	0.172385642562035	PTRH2	51651	peptidyl-tRNA hydrolase 2 [Source:HGNC Symbol;Acc:HGNC:24265]
-0.280856894791915	0.170842811221678	SH2B1	25970	SH2B adaptor protein 1 [Source:HGNC Symbol;Acc:HGNC:30417]
-0.280651298840982	0.00867847932365384	AHCYL2	23382	adenosylhomocysteinase-like 2 [Source:HGNC Symbol;Acc:HGNC:22204]
-0.280602389911817	0.103021753541945	ECHS1	1892	enoyl CoA hydratase, short chain, 1, mitochondrial [Source:HGNC Symbol;Acc:HGNC:3151]
-0.28035008062033	0.181317504079594	CCNC	892	cyclin C [Source:HGNC Symbol;Acc:HGNC:1581]
-0.280333288502354	0.0406343434499116	GPC4	2239	glypican 4 [Source:HGNC Symbol;Acc:HGNC:4452]
-0.280269349368108	0.197496359657743	PDZRN3	23024	PDZ domain containing ring finger 3 [Source:HGNC Symbol;Acc:HGNC:17704]
-0.280128515447333	0.124429638753298	KRAS	3845	Kirsten rat sarcoma viral oncogene homolog [Source:HGNC Symbol;Acc:HGNC:6407]
-0.279971652060896	0.0828976512115683	IL1RAP	3556	interleukin 1 receptor accessory protein [Source:HGNC Symbol;Acc:HGNC:5995]
-0.279788059857854	0.191836012976523	ZNF555	148254	zinc finger protein 555 [Source:HGNC Symbol;Acc:HGNC:28382]
-0.279782980238125	0.142662047468609	GPX5	2880	glutathione peroxidase 5 (epididymal androgen-related protein) [Source:HGNC Symbol;Acc:HGNC:4557]
-0.279498304938635	0.164127937043847	SOS1	6654	son of sevenless homolog 1 (Drosophila) [Source:HGNC Symbol;Acc:HGNC:11187]
-0.279495739081996	0.154392004181121	ATXN7L3B	552889	ataxin 7-like 3B [Source:HGNC Symbol;Acc:HGNC:37931]
-0.279470665604341	0.165106182338964	MAPKAPK2	9261	mitogen-activated protein kinase-activated protein kinase 2 [Source:HGNC Symbol;Acc:HGNC:6887]
-0.279439295761889	0.0721278154414687	SNX6	58533	sorting nexin 6 [Source:HGNC Symbol;Acc:HGNC:14970]
-0.279407258566648	0.0888693837588945	GOT2	2806	glutamic-oxaloacetic transaminase 2, mitochondrial [Source:HGNC Symbol;Acc:HGNC:4433]
-0.279344893066142	0.169416708668937	MORC3	23515	MORC family CW-type zinc finger 3 [Source:HGNC Symbol;Acc:HGNC:23572]
-0.278993378013424	0.0389073027518804	PPP1R10	5514	protein phosphatase 1, regulatory subunit 10 [Source:HGNC Symbol;Acc:HGNC:9284]
-0.278749599654258	0.0781533885368146	GLI3	2737	GLI family zinc finger 3 [Source:HGNC Symbol;Acc:HGNC:4319]
-0.278478483290548	0.103430905926865	ADH5	128	alcohol dehydrogenase 5 (class III), chi polypeptide [Source:HGNC Symbol;Acc:HGNC:253]
-0.278244044265335	0.105301227316365	CYB5R1	51706	cytochrome b5 reductase 1 [Source:HGNC Symbol;Acc:HGNC:13397]
-0.278071060984405	0.136332575586641	CPNE8	144402	copine VIII [Source:HGNC Symbol;Acc:HGNC:23498]
-0.278052674867901	0.0354299166369539	STX8	9482	syntaxin 8 [Source:HGNC Symbol;Acc:HGNC:11443]
-0.278016028347046	0.149820366159454	ILKAP	80895	integrin-linked kinase-associated serine/threonine phosphatase [Source:HGNC Symbol;Acc:HGNC:15566]
-0.277563260385948	0.185836349364405	MAGI1	9223	membrane associated guanylate kinase, WW and PDZ domain containing 1 [Source:HGNC Symbol;Acc:HGNC:946]
-0.276926892809697	0.0789225316656534	ATP7A	538	ATPase, Cu++ transporting, alpha polypeptide [Source:HGNC Symbol;Acc:HGNC:869]
-0.276497282264818	0.185896364845362	HEXB	3074	hexosaminidase B (beta polypeptide) [Source:HGNC Symbol;Acc:HGNC:4879]
-0.276284109424589	0.140627894369887	PIP5K1C	23396	phosphatidylinositol-4-phosphate 5-kinase, type I, gamma [Source:HGNC Symbol;Acc:HGNC:8996]
-0.276169094192826	0.16449174350063	PTAR1	375743	protein prenyltransferase alpha subunit repeat containing 1 [Source:HGNC Symbol;Acc:HGNC:30449]
-0.276139594972451	0.181449566324011	COG2	22796	component of oligomeric golgi complex 2 [Source:HGNC Symbol;Acc:HGNC:6546]
-0.276107648548465	0.106606049168669	IREB2	3658	iron-responsive element binding protein 2 [Source:HGNC Symbol;Acc:HGNC:6115]
-0.276044172003847	0.112399442210539	PRCP	5547	prolylcarboxypeptidase (angiotensinase C) [Source:HGNC Symbol;Acc:HGNC:9344]
-0.275624660304141	0.0657230175653289	SLAIN2	57606	SLAIN motif family, member 2 [Source:HGNC Symbol;Acc:HGNC:29282]
-0.275487241629152	0.0867171549452054	C14orf2	9556	chromosome 14 open reading frame 2 [Source:HGNC Symbol;Acc:HGNC:1188]
-0.275438999419833	0.180699867963588	CDC23	8697	cell division cycle 23 [Source:HGNC Symbol;Acc:HGNC:1724]
-0.275260930220326	0.116567909530836	ISCU	23479	iron-sulfur cluster assembly enzyme [Source:HGNC Symbol;Acc:HGNC:29882]
-0.275195607116091	0.173249831396612	SIRT2	22933	sirtuin 2 [Source:HGNC Symbol;Acc:HGNC:10886]
-0.275145484651471	0.0893260882862571	PQLC3	130814	PQ loop repeat containing 3 [Source:HGNC Symbol;Acc:HGNC:28503]
-0.275012386649194	0.0385395221941575	DLG3	1741	discs, large homolog 3 (Drosophila) [Source:HGNC Symbol;Acc:HGNC:2902]
-0.274963637349096	0.106153970700294	CASP9	842	caspase 9, apoptosis-related cysteine peptidase [Source:HGNC Symbol;Acc:HGNC:1511]
-0.274913719738556	0.123965986024535	TOR1A	1861	torsin family 1, member A (torsin A) [Source:HGNC Symbol;Acc:HGNC:3098]
-0.274779320793498	0.0431557004828099	H3F3A	3021	H3 histone, family 3A [Source:HGNC Symbol;Acc:HGNC:4764]
-0.274744507323091	0.186860854483853	BEX1	55859	brain expressed, X-linked 1 [Source:HGNC Symbol;Acc:HGNC:1036]
-0.274298450515728	0.179242701124266	ZNF451	26036	zinc finger protein 451 [Source:HGNC Symbol;Acc:HGNC:21091]
-0.27403197709326	0.19963751213301	SLC9A3R1	9368	solute carrier family 9, subfamily A (NHE3, cation proton antiporter 3), member 3 regulator 1 [Source:HGNC Symbol;Acc:HGNC:11075]
-0.273750476069462	0.118883620183295	PUM2	23369	pumilio RNA-binding family member 2 [Source:HGNC Symbol;Acc:HGNC:14958]
-0.273687408408744	0.136566288274093		100529855	
-0.273588996074263	0.0761516418705434	TMEM86A	144110	transmembrane protein 86A [Source:HGNC Symbol;Acc:HGNC:26890]
-0.273524913642196	0.0352692043007473	VPS8	23355	vacuolar protein sorting 8 homolog (S. cerevisiae) [Source:HGNC Symbol;Acc:HGNC:29122]
-0.27350348309856	0.000461935080772479	EXOC2	55770	exocyst complex component 2 [Source:HGNC Symbol;Acc:HGNC:24968]
-0.273460901222271	0.112558417224054	TSTD1	100131187	thiosulfate sulfurtransferase (rhodanese)-like domain containing 1 [Source:HGNC Symbol;Acc:HGNC:35410]
-0.273068566880492	0.0790028749016308	KPNA6	23633	karyopherin alpha 6 (importin alpha 7) [Source:HGNC Symbol;Acc:HGNC:6399]
-0.272972243402634	0.0863336108471742	ZC3H12B	340554	zinc finger CCCH-type containing 12B [Source:HGNC Symbol;Acc:HGNC:17407]
-0.272753397712613	0.171117806784978	MTMR4	9110	myotubularin related protein 4 [Source:HGNC Symbol;Acc:HGNC:7452]
-0.272700526294074	0.045899807165401	SEC22C	9117	SEC22 vesicle trafficking protein homolog C (S. cerevisiae) [Source:HGNC Symbol;Acc:HGNC:16828]
-0.271945316618518	0.117937471182107	POU2F1	5451	POU class 2 homeobox 1 [Source:HGNC Symbol;Acc:HGNC:9212]
-0.271757066725191	0.130035804201388	USP13	8975	ubiquitin specific peptidase 13 (isopeptidase T-3) [Source:HGNC Symbol;Acc:HGNC:12611]
-0.27175058713089	0.176135629308092	CHCHD1	118487	coiled-coil-helix-coiled-coil-helix domain containing 1 [Source:HGNC Symbol;Acc:HGNC:23518]
-0.271574184742074	0.174888529203659	MRPS30	10884	mitochondrial ribosomal protein S30 [Source:HGNC Symbol;Acc:HGNC:8769]
-0.271452751584564	0.172270628751381	IARS2	55699	isoleucyl-tRNA synthetase 2, mitochondrial [Source:HGNC Symbol;Acc:HGNC:29685]
-0.271292643922665	0.126265668900799	FBXO34	55030	F-box protein 34 [Source:HGNC Symbol;Acc:HGNC:20201]
-0.270973420170298	0.051912753442221	ANO10	55129	anoctamin 10 [Source:HGNC Symbol;Acc:HGNC:25519]
-0.270883666396528	0.168605579633883	VSIG10L	147645	V-set and immunoglobulin domain containing 10 like [Source:HGNC Symbol;Acc:HGNC:27111]
-0.270855831277822	0.149114790224418	ZNF607	84775	zinc finger protein 607 [Source:HGNC Symbol;Acc:HGNC:28192]
-0.270741249626278	0.156239991381333	TMEM30A	55754	transmembrane protein 30A [Source:HGNC Symbol;Acc:HGNC:16667]
-0.27050115955944	0.111259969955632	CASK	8573	calcium/calmodulin-dependent serine protein kinase (MAGUK family) [Source:HGNC Symbol;Acc:HGNC:1497]
-0.270406466881243	0.180312591256996	COMT	1312	catechol-O-methyltransferase [Source:HGNC Symbol;Acc:HGNC:2228]
-0.270203811918253	0.0536276449749275	PFDN6	10471	prefoldin subunit 6 [Source:HGNC Symbol;Acc:HGNC:4926]
-0.270045988871911	0.137203412040597	GALNT6	11226	polypeptide N-acetylgalactosaminyltransferase 6 [Source:HGNC Symbol;Acc:HGNC:4128]
-0.269976842269385	0.183359392890286	DSCR3	10311	Down syndrome critical region gene 3 [Source:HGNC Symbol;Acc:HGNC:3044]
-0.269790727298798	0.148445530068252	RAB11FIP1	80223	RAB11 family interacting protein 1 (class I) [Source:HGNC Symbol;Acc:HGNC:30265]
-0.269757223131437	0.00914649505841513	sept-11	55752	septin 11 [Source:HGNC Symbol;Acc:HGNC:25589]
-0.269558941594232	0.15692704031468	SNX19	399979	sorting nexin 19 [Source:HGNC Symbol;Acc:HGNC:21532]
-0.269480731620399	0.107666015972071	PDZD11	51248	PDZ domain containing 11 [Source:HGNC Symbol;Acc:HGNC:28034]
-0.269389170077959	0.143149862491582	ZNF81	347344	zinc finger protein 81 [Source:HGNC Symbol;Acc:HGNC:13156]
-0.269364002401521	0.162274476246786	NOL7	51406	nucleolar protein 7, 27kDa [Source:HGNC Symbol;Acc:HGNC:21040]
-0.2692222769697	0.133231722657374	GRN	2896	granulin [Source:HGNC Symbol;Acc:HGNC:4601]
-0.269114703599799	0.115266814702846	DDC	1644	dopa decarboxylase (aromatic L-amino acid decarboxylase) [Source:HGNC Symbol;Acc:HGNC:2719]
-0.268771052990261	0.176367442129833	SLC39A1	27173	solute carrier family 39 (zinc transporter), member 1 [Source:HGNC Symbol;Acc:HGNC:12876]
-0.268649211935319	0.181084172403035	ARPC4	10093	actin related protein 2/3 complex, subunit 4, 20kDa [Source:HGNC Symbol;Acc:HGNC:707]
-0.268473068183961	0.179873437343269	USP28	57646	ubiquitin specific peptidase 28 [Source:HGNC Symbol;Acc:HGNC:12625]
-0.268129091826713	0.0030171664781651	SUDS3	64426	suppressor of defective silencing 3 homolog (S. cerevisiae) [Source:HGNC Symbol;Acc:HGNC:29545]
-0.268089397059863	0.0924463069557589	CERK	64781	ceramide kinase [Source:HGNC Symbol;Acc:HGNC:19256]
-0.267308332853966	0.189575452043489	FARS2	10667	phenylalanyl-tRNA synthetase 2, mitochondrial [Source:HGNC Symbol;Acc:HGNC:21062]
-0.267175182104829	0.0192025762520445	RHOA	387	ras homolog family member A [Source:HGNC Symbol;Acc:HGNC:667]
-0.267063653686913	0.0674856709198096	NIT1	4817	nitrilase 1 [Source:HGNC Symbol;Acc:HGNC:7828]
-0.266889491448045	0.146949473915885	ARFIP1	27236	ADP-ribosylation factor interacting protein 1 [Source:HGNC Symbol;Acc:HGNC:21496]
-0.266809476861172	1.56334315104582e-05	NDUFA2	4695	NADH dehydrogenase (ubiquinone) 1 alpha subcomplex, 2, 8kDa [Source:HGNC Symbol;Acc:HGNC:7685]
-0.266055280361036	0.18685588338911	SUGP1	57794	SURP and G patch domain containing 1 [Source:HGNC Symbol;Acc:HGNC:18643]
-0.265790146946229	0.183086490293981	ATP1A1	476	ATPase, Na+/K+ transporting, alpha 1 polypeptide [Source:HGNC Symbol;Acc:HGNC:799]
-0.265647723695595	0.0905207832562065	XPO6	23214	exportin 6 [Source:HGNC Symbol;Acc:HGNC:19733]
-0.265442393211112	0.175468514400202	CCNDBP1	23582	cyclin D-type binding-protein 1 [Source:HGNC Symbol;Acc:HGNC:1587]
-0.265432302109994	0.0991042452267123	EIF2AK4	440275	eukaryotic translation initiation factor 2 alpha kinase 4 [Source:HGNC Symbol;Acc:HGNC:19687]
-0.265402298298773	0.154462444529396	TANK	10010	TRAF family member-associated NFKB activator [Source:HGNC Symbol;Acc:HGNC:11562]
-0.265383880122015	0.178673097754208	SOAT1	6646	sterol O-acyltransferase 1 [Source:HGNC Symbol;Acc:HGNC:11177]
-0.265382169658281	0.182588763848499	TMEM127	55654	transmembrane protein 127 [Source:HGNC Symbol;Acc:HGNC:26038]
-0.265354989403036	0.0579866863594089	AMFR	267	autocrine motility factor receptor, E3 ubiquitin protein ligase [Source:HGNC Symbol;Acc:HGNC:463]
-0.265316860246002	0.118639579379999	FKBP8	23770	FK506 binding protein 8, 38kDa [Source:HGNC Symbol;Acc:HGNC:3724]
-0.265168853784419	0.120854814494462	MUC13	56667	mucin 13, cell surface associated [Source:HGNC Symbol;Acc:HGNC:7511]
-0.264952714965482	0.0788842044977119	TMED8	283578	transmembrane emp24 protein transport domain containing 8 [Source:HGNC Symbol;Acc:HGNC:18633]
-0.264778804680675	0.026218362182754	ATAD1	84896	ATPase family, AAA domain containing 1 [Source:HGNC Symbol;Acc:HGNC:25903]
-0.264159427281262	0.0863759305534396	RAB22A	57403	RAB22A, member RAS oncogene family [Source:HGNC Symbol;Acc:HGNC:9764]
-0.263349857653869	0.161434329461345	MIEF1	54471	mitochondrial elongation factor 1 [Source:HGNC Symbol;Acc:HGNC:25979]
-0.262335718352789	0.199451523824967	C1orf56	54964	chromosome 1 open reading frame 56 [Source:HGNC Symbol;Acc:HGNC:26045]
-0.26218880345171	0.132378589881598	FCHO2	115548	FCH domain only 2 [Source:HGNC Symbol;Acc:HGNC:25180]
-0.261781776201754	0.0377420171929809	DIO2	1734	deiodinase, iodothyronine, type II [Source:HGNC Symbol;Acc:HGNC:2884]
-0.261652396298552	0.192026203899381	SRSF9	283459	serine/arginine-rich splicing factor 9 [Source:HGNC Symbol;Acc:HGNC:10791]
-0.261513845306549	0.151630456702062	ANXA5	308	annexin A5 [Source:HGNC Symbol;Acc:HGNC:543]
-0.261496694641245	0.077643451402064	ADD3	120	adducin 3 (gamma) [Source:HGNC Symbol;Acc:HGNC:245]
-0.261394889985638	0.173411154001711	CLN8	2055	ceroid-lipofuscinosis, neuronal 8 (epilepsy, progressive with mental retardation) [Source:HGNC Symbol;Acc:HGNC:2079]
-0.261166837075794	0.160998072197572	C12orf57	113246	chromosome 12 open reading frame 57 [Source:HGNC Symbol;Acc:HGNC:29521]

-0.261138512886885	0.133064086249078	GGPS1	9453	geranylgeranyl diphosphate synthase 1 [Source:HGNC Symbol;Acc:HGNC:4249]
-0.260947892555707	0.155670405169267	ZNF564	163050	zinc finger protein 564 [Source:HGNC Symbol;Acc:HGNC:31106]
-0.260946939118526	0.197399305080039	DPP8	54878	dipeptidyl-peptidase 8 [Source:HGNC Symbol;Acc:HGNC:16490]
-0.260858826016733	0.195891610018759	NR3C1	2908	nuclear receptor subfamily 3, group C, member 1 (glucocorticoid receptor) [Source:HGNC Symbol;Acc:HGNC:7978]
-0.260829812576961	0.120503281460748	TBX19	9095	T-box 19 [Source:HGNC Symbol;Acc:HGNC:11596]
-0.260742393741654	0.0953781128739385	ENTPD5	957	ectonucleoside triphosphate diphosphohydrolase 5 [Source:HGNC Symbol;Acc:HGNC:3367]
-0.260723227170099	0.156832196509641	RPUSD4	84881	RNA pseudouridylate synthase domain containing 4 [Source:HGNC Symbol;Acc:HGNC:25898]
-0.260322008982557	0.0482083941975446	TBC1D1	23216	TBC1 (tre-2/USP6, BUB2, cdc16) domain family, member 1 [Source:HGNC Symbol;Acc:HGNC:11578]
-0.260255848751862	0.181419953039641	SMIM15	643155	small integral membrane protein 15 [Source:HGNC Symbol;Acc:HGNC:33861]
-0.260241832477904	0.179599333929821	SPINT1	6692	serine peptidase inhibitor, Kunitz type 1 [Source:HGNC Symbol;Acc:HGNC:11246]
-0.259909393695503	0.0114458557927152	TBC1D14	57533	TBC1 domain family, member 14 [Source:HGNC Symbol;Acc:HGNC:29246]
-0.259885936608058	0.177515267285038	CIPC	85457	CLOCK-interacting pacemaker [Source:HGNC Symbol;Acc:HGNC:20365]
-0.2593500972743	0.165247555905749	SCP2	6342	sterol carrier protein 2 [Source:HGNC Symbol;Acc:HGNC:10606]
-0.259346679275245	0.184999398387823	SEL1L	6400	sel-1 suppressor of lin-12-like (C. elegans) [Source:HGNC Symbol;Acc:HGNC:10717]
-0.259267337503139	0.179223829554516	UBR3	130507	ubiquitin protein ligase E3 component n-recognin 3 (putative) [Source:HGNC Symbol;Acc:HGNC:30467]
-0.259181199331618	0.172993218501763	AAGAB	79719	alpha- and gamma-adaptin binding protein [Source:HGNC Symbol;Acc:HGNC:25662]
-0.25853309950331	0.0916700363284474	COPS6	10980	COP9 signalosome subunit 6 [Source:HGNC Symbol;Acc:HGNC:21749]
-0.258234044627042	0.0988180210578447	mars-05	54708	membrane-associated ring finger (C3HC4) 5 [Source:HGNC Symbol;Acc:HGNC:26025]
-0.2581579392113	0.0774420550960759	STRN3	29966	striatin, calmodulin binding protein 3 [Source:HGNC Symbol;Acc:HGNC:15720]
-0.25799333492546	0.0615318846775528	ECM2	1842	extracellular matrix protein 2, female organ and adipocyte specific [Source:HGNC Symbol;Acc:HGNC:3154]
-0.257907067961963	0.142411316002853	ICE1	23379	interactor of little elongator complex ELL subunit 1 [Source:HGNC Symbol;Acc:HGNC:29154]
-0.257694350039385	0.0246456927439524	FAF2	23197	Fas associated factor family member 2 [Source:HGNC Symbol;Acc:HGNC:24666]
-0.257499466289981	0.145737933791125	PPAPDC2	403313	phosphatidic acid phosphatase type 2 domain containing 2 [Source:HGNC Symbol;Acc:HGNC:23682]
-0.257485732953143	0.195726019750854	BRMS1	25855	breast cancer metastasis suppressor 1 [Source:HGNC Symbol;Acc:HGNC:17262]
-0.257456314645094	0.135300146763188	PTMS	5763	parathymosin [Source:HGNC Symbol;Acc:HGNC:9629]
-0.257374146726836	0.152657715536638	TBRG4	9238	transforming growth factor beta regulator 4 [Source:HGNC Symbol;Acc:HGNC:17443]
-0.257026699412664	0.124491106610288	CPEB4	80315	cytoplasmic polyadenylation element binding protein 4 [Source:HGNC Symbol;Acc:HGNC:21747]
-0.25652916186162	0.11393265154884	NDFIP2	54602	Nedd4 family interacting protein 2 [Source:HGNC Symbol;Acc:HGNC:18537]
-0.255848008361952	0.123639349797493	FBXO25	26260	F-box protein 25 [Source:HGNC Symbol;Acc:HGNC:13596]
-0.255804080568891	0.156275619883464	VAPA	9218	VAMP (vesicle-associated membrane protein)-associated protein A, 33kDa [Source:HGNC Symbol;Acc:HGNC:12648]
-0.255344039979752	0.171596758819403	FBXO8	26269	F-box protein 8 [Source:HGNC Symbol;Acc:HGNC:13587]
-0.255010065197167	0.126684321163398	ZC3H14	79882	zinc finger CCCH-type containing 14 [Source:HGNC Symbol;Acc:HGNC:20509]
-0.254983064237789	0.183805798945215	EXD2	55218	exonuclease 3'-5' domain containing 2 [Source:HGNC Symbol;Acc:HGNC:20217]
-0.254892986292724	0.110908992145298	PKN1	5585	protein kinase N1 [Source:HGNC Symbol;Acc:HGNC:9405]
-0.254106963532927	0.119821788711758	DBN1	1627	drebrin 1 [Source:HGNC Symbol;Acc:HGNC:2695]
-0.254077317938778	0.103214848924392	USP22	23326	ubiquitin specific peptidase 22 [Source:HGNC Symbol;Acc:HGNC:12621]
-0.254061728022999	0.196373632072551	SUMF1	285362	sulfatase modifying factor 1 [Source:HGNC Symbol;Acc:HGNC:20376]
-0.253935705064009	0.185284802552505	NDUFA6	4700	NADH dehydrogenase (ubiquinone) 1 alpha subcomplex, 6, 14kDa [Source:HGNC Symbol;Acc:HGNC:7690]
-0.253830534996111	0.047985051731682	TSPAN1	10103	tetraspanin 1 [Source:HGNC Symbol;Acc:HGNC:20657]
-0.253636627140054	0.17091161366106	GOT1	2805	glutamic-oxaloacetic transaminase 1, soluble [Source:HGNC Symbol;Acc:HGNC:4432]
-0.253531613157512	0.192505487185552	MYLK	4638	myosin light chain kinase [Source:HGNC Symbol;Acc:HGNC:7590]
-0.253341345830192	0.111477510077461	KIAA2026	158358	KIAA2026 [Source:HGNC Symbol;Acc:HGNC:23378]
-0.252412252352531	0.0377120326411364	PAK1	5058	p21 protein (Cdc42/Rac)-activated kinase 1 [Source:HGNC Symbol;Acc:HGNC:8590]
-0.252093397684567	0.0492279214536018	PGRMC1	10857	progesterone receptor membrane component 1 [Source:HGNC Symbol;Acc:HGNC:16090]
-0.252072193151937	0.191756103287649	ZNF124	7678	zinc finger protein 124 [Source:HGNC Symbol;Acc:HGNC:12907]
-0.25184878016734	0.118686345020236	RIPK1	8737	receptor (TNFRSF)-interacting serine-threonine kinase 1 [Source:HGNC Symbol;Acc:HGNC:10019]
-0.251666500099786	0.167084029332074	CEACAM5	1048	carcinoembryonic antigen-related cell adhesion molecule 5 [Source:HGNC Symbol;Acc:HGNC:1817]
-0.251358211470645	0.147961544161517	BMPR2	659	bone morphogenetic protein receptor, type II (serine/threonine kinase) [Source:HGNC Symbol;Acc:HGNC:1078]
-0.251101107408852	0.054266875023741	SLC26A3	1811	solute carrier family 26 (anion exchanger), member 3 [Source:HGNC Symbol;Acc:HGNC:3018]
-0.251074404315507	0.15717532338637	THRB	7068	thyroid hormone receptor, beta [Source:HGNC Symbol;Acc:HGNC:11799]
-0.25097009434468	0.153560461186352	SMAD4	4089	SMAD family member 4 [Source:HGNC Symbol;Acc:HGNC:6770]
-0.250739144054991	0.142817607513863	H2AFJ	55766	H2A histone family, member J [Source:HGNC Symbol;Acc:HGNC:14456]
-0.250693796858517	0.192852760753691	CDH1	999	cadherin 1, type 1, E-cadherin (epithelial) [Source:HGNC Symbol;Acc:HGNC:1748]
-0.250646976994565	0.131523565794788	CPSF4L	642843	cleavage and polyadenylation specific factor 4-like [Source:HGNC Symbol;Acc:HGNC:33632]
-0.250551312160612	0.119610755992963	NACA	4666	nascent polypeptide-associated complex alpha subunit [Source:HGNC Symbol;Acc:HGNC:7629]
-0.250305276781993	0.126754759426029	F11R	50848	F11 receptor [Source:HGNC Symbol;Acc:HGNC:14685]
-0.250168887700957	0.093312287723396	PAFAH1B1	5048	platelet-activating factor acetylhydrolase 1b, regulatory subunit 1 (45kDa) [Source:HGNC Symbol;Acc:HGNC:8574]
-0.250098241766463	0.0354108865283374	SLC9A2	6549	solute carrier family 9, subfamily A (NHE2, cation proton antiporter 2), member 2 [Source:HGNC Symbol;Acc:HGNC:11072]
-0.250071822305189	0.195985980500788	GPR107	57720	G protein-coupled receptor 107 [Source:HGNC Symbol;Acc:HGNC:17830]
-0.249967905017582	0.0946994797416358	PGRMC2	10424	progesterone receptor membrane component 2 [Source:HGNC Symbol;Acc:HGNC:16089]
-0.249587488827298	0.161998923717359	SALL2	6297	spalt-like transcription factor 2 [Source:HGNC Symbol;Acc:HGNC:10526]
-0.249427684393318	0.130574506284129	CYTH3	9265	cytohesin 3 [Source:HGNC Symbol;Acc:HGNC:9504]
-0.249081480714529	0.0376017317754525	ANKIB1	54467	ankyrin repeat and IBR domain containing 1 [Source:HGNC Symbol;Acc:HGNC:22215]
-0.248975473778648	0.127229779527583	CYSTM1	84418	cysteine-rich transmembrane module containing 1 [Source:HGNC Symbol;Acc:HGNC:30239]
-0.248959503736359	0.132172198858197	CSNK1G1	53944	casein kinase 1, gamma 1 [Source:HGNC Symbol;Acc:HGNC:2454]
-0.248804541535371	0.178353394566382	PIK3R4	30849	phosphoinositide-3-kinase, regulatory subunit 4 [Source:HGNC Symbol;Acc:HGNC:8982]
-0.248548799935998	0.168602198241335	ATG4D	84971	autophagy related 4D, cysteine peptidase [Source:HGNC Symbol;Acc:HGNC:20789]
-0.248402307817001	0.0290266865097132	SSR4	6748	signal sequence receptor, delta [Source:HGNC Symbol;Acc:HGNC:11326]
-0.248197891411642	0.126423636257906	EFCAB14	9813	EF-hand calcium binding domain 14 [Source:HGNC Symbol;Acc:HGNC:29051]
-0.247984010079438	0.0817331030317724	USP7	7874	ubiquitin specific peptidase 7 (herpes virus-associated) [Source:HGNC Symbol;Acc:HGNC:12630]
-0.247473564085528	0.0701228381526327	GNS	2799	glucosamine (N-acetyl)-6-sulfatase [Source:HGNC Symbol;Acc:HGNC:4422]
-0.247448095498908	0.0799520318500332	HS2ST1	9653	heparan sulfate 2-O-sulfotransferase 1 [Source:HGNC Symbol;Acc:HGNC:5193]
-0.247162563609198	0.0594624540743425	AREL1	9870	apoptosis resistant E3 ubiquitin protein ligase 1 [Source:HGNC Symbol;Acc:HGNC:20363]
-0.246309179377644	0.152952340343971	AGPAT1	10554	1-acylglycerol-3-phosphate O-acyltransferase 1 [Source:HGNC Symbol;Acc:HGNC:324]
-0.246250036133398	0.182864049862838	DPH5	51611	diphthamide biosynthesis 5 [Source:HGNC Symbol;Acc:HGNC:24270]
-0.246219627555655	0.163976172339771	CA12	771	carbonic anhydrase XII [Source:HGNC Symbol;Acc:HGNC:1371]
-0.24595764109206	0.192038615329976	PTGR1	22949	prostaglandin reductase 1 [Source:HGNC Symbol;Acc:HGNC:18429]
-0.245825895996776	0.164655496701597	TOM1L1	10040	target of myb1 (chicken)-like 1 [Source:HGNC Symbol;Acc:HGNC:11983]
-0.245733572447409	0.17926182047747	SLC44A4	80736	solute carrier family 44, member 4 [Source:HGNC Symbol;Acc:HGNC:13941]
-0.245292343592203	0.0568638602341489	MRPL34	64981	mitochondrial ribosomal protein L34 [Source:HGNC Symbol;Acc:HGNC:14488]
-0.245021230874741	0.150433047400738	HMG20A	10363	high mobility group 20A [Source:HGNC Symbol;Acc:HGNC:5001]
-0.244586440688559	0.101779019203326	FECH	2235	ferrochelatase [Source:HGNC Symbol;Acc:HGNC:3647]
-0.244481186379454	0.0516758692017779	ARFGEF2	10564	ADP-ribosylation factor guanine nucleotide-exchange factor 2 (brefeldin A-inhibited) [Source:HGNC Symbol;Acc:HGNC:15853]
-0.244477272984198	0.0834766490886719	POLR2E	5434	polymerase (RNA) II (DNA directed) polypeptide E, 25kDa [Source:HGNC Symbol;Acc:HGNC:9192]
-0.244439413907705	0.185504289062597	GPS1	2873	G protein pathway suppressor 1 [Source:HGNC Symbol;Acc:HGNC:4549]
-0.244301284825893	0.0132788089841976	NLK	51701	nemo-like kinase [Source:HGNC Symbol;Acc:HGNC:29858]
-0.244099604215707	0.116219087501346	PRKAR2A	5576	protein kinase, cAMP-dependent, regulatory, type II, alpha [Source:HGNC Symbol;Acc:HGNC:9391]
-0.244015759851324	0.0309991948672272	SSU72	29101	SSU72 RNA polymerase II CTD phosphatase homolog (S. cerevisiae) [Source:HGNC Symbol;Acc:HGNC:25016]
-0.243318046387896	0.199378947815192	AXIN2	8313	axin 2 [Source:HGNC Symbol;Acc:HGNC:904]
-0.24307126247904	0.199317519625377	FAM129A	116496	family with sequence similarity 129, member A [Source:HGNC Symbol;Acc:HGNC:16784]
-0.242969357600897	0.117140708134234	STARD10	10809	StAR-related lipid transfer (START) domain containing 10 [Source:HGNC Symbol;Acc:HGNC:10666]
-0.2428586996479	0.0575837679702026	UROD	7389	uroporphyrinogen decarboxylase [Source:HGNC Symbol;Acc:HGNC:12591]
-0.242236769873811	0.0807067628376732	WASL	8976	Wiskott-Aldrich syndrome-like [Source:HGNC Symbol;Acc:HGNC:12735]
-0.241990091353582	0.079639909839021	ASNA1	439	arsA arsenite transporter, ATP-binding, homolog 1 (bacterial) [Source:HGNC Symbol;Acc:HGNC:752]
-0.241829560865186	0.156379025032799	PDCD2	5134	programmed cell death 2 [Source:HGNC Symbol;Acc:HGNC:8762]
-0.241173216192317	0.182555060435871	TGFBR1	7046	transforming growth factor, beta receptor 1 [Source:HGNC Symbol;Acc:HGNC:11772]
-0.240944080233439	0.0497748118269983	MPDU1	9526	mannose-P-dolichol utilization defect 1 [Source:HGNC Symbol;Acc:HGNC:7207]
-0.240669000067879	0.0943462762707529	GPR32	2854	G protein-coupled receptor 32 [Source:HGNC Symbol;Acc:HGNC:4487]
-0.240650654741231	0.0652796227171388	CHP1	11261	calcineurin-like EF-hand protein 1 [Source:HGNC Symbol;Acc:HGNC:17433]
-0.240596443203172	0.101017028444168	SLC38A9	153129	solute carrier family 38, member 9 [Source:HGNC Symbol;Acc:HGNC:26907]
-0.240435789465673	0.190825329579751	TMEM69	51249	transmembrane protein 69 [Source:HGNC Symbol;Acc:HGNC:28035]
-0.240331827258304	0.0889186591056947	PSD	5662	pleckstrin and Sec7 domain containing [Source:HGNC Symbol;Acc:HGNC:9507]
-0.239585871048677	0.143004550037443	TSPAN31	6302	tetraspanin 31 [Source:HGNC Symbol;Acc:HGNC:10539]
-0.239500571739314	0.180913982943362	MAD2L1BP	9587	MAD2L1 binding protein [Source:HGNC Symbol;Acc:HGNC:21059]
-0.239473353416937	0.190435898374997	IGF2BP3	10643	insulin-like growth factor 2 mRNA binding protein 3 [Source:HGNC Symbol;Acc:HGNC:28868]
-0.239358069176561	0.172532781097983	PDE3A	5139	phosphodiesterase 3A, cGMP-inhibited [Source:HGNC Symbol;Acc:HGNC:8778]
-0.239258210374827	0.141842919996866	CDIPT	10423	CDP-diacylglycerol--inositol 3-phosphatidyltransferase [Source:HGNC Symbol;Acc:HGNC:1769]
-0.239213590159613	0.0321617356937294	CDK13	8621	cyclin-dependent kinase 13 [Source:HGNC Symbol;Acc:HGNC:1733]
-0.239195792299998	0.111148059562054	CASQ2	845	calsequestrin 2 (cardiac muscle) [Source:HGNC Symbol;Acc:HGNC:1513]
-0.239170385959818	0.150976891609573	FBXO7	25793	F-box protein 7 [Source:HGNC Symbol;Acc:HGNC:13586]
-0.239043189555509	0.0837101066125655	OTUD7B	56957	OTU deubiquitinase 7B [Source:HGNC Symbol;Acc:HGNC:16683]
-0.238528890270516	0.184045993891982	CNEP1R1	255919	CTD nuclear envelope phosphatase 1 regulatory subunit 1 [Source:HGNC Symbol;Acc:HGNC:26759]
-0.238181200370072	0.110774931511472	MTMR3	8897	myotubularin related protein 3 [Source:HGNC Symbol;Acc:HGNC:7451]
-0.23798484222309	0.115200508100517	YIPF3	25844	Yip1 domain family, member 3 [Source:HGNC Symbol;Acc:HGNC:21023]
-0.236952209521207	0.0764740066662724	GABARAP	11337	GABA(A) receptor-associated protein [Source:HGNC Symbol;Acc:HGNC:4067]
-0.23676716163539	0.182579779693951	sept-07	989	septin 7 [Source:HGNC Symbol;Acc:HGNC:1717]
-0.236520756188417	0.148068007958232	ARCN1	372	archain 1 [Source:HGNC Symbol;Acc:HGNC:649]
-0.236404496044717	0.195063383061527	AGFG2	3268	ArfGAP with FG repeats 2 [Source:HGNC Symbol;Acc:HGNC:5177]
-0.236308029903431	0.162296542481716	CEACAM7	1087	carcinoembryonic antigen-related cell adhesion molecule 7 [Source:HGNC Symbol;Acc:HGNC:1819]
-0.236184070171772	0.159818576621633	LIMA1	51474	LIM domain and actin binding 1 [Source:HGNC Symbol;Acc:HGNC:24636]
-0.236138946298263	0.0630971472623031	MTIF3	219402	mitochondrial translational initiation factor 3 [Source:HGNC Symbol;Acc:HGNC:29788]
-0.235952835303199	0.194654829007159	SAMD3	154075	sterile alpha motif domain containing 3 [Source:HGNC Symbol;Acc:HGNC:21574]
-0.235938466988518	0.0920763613614144	MGST2	4258	microsomal glutathione S-transferase 2 [Source:HGNC Symbol;Acc:HGNC:7063]
-0.235563904284163	0.181449560185621	SRSF9	8683	serine/arginine-rich splicing factor 9 [Source:HGNC Symbol;Acc:HGNC:10791]
-0.235348587454807	0.114800910132516	SSR3	6747	signal sequence receptor, gamma (translocon-associated protein gamma) [Source:HGNC Symbol;Acc:HGNC:11325]
-0.235230378333451	0.129808920219804	MAPKBP1	23005	mitogen-activated protein kinase binding protein 1 [Source:HGNC Symbol;Acc:HGNC:29536]
-0.234931184194661	0.147864143686358	RBPMS	11030	RNA binding protein with multiple splicing [Source:HGNC Symbol;Acc:HGNC:19097]
-0.234856929287513	0.113540052917839	KLHL36	79786	kelch-like family member 36 [Source:HGNC Symbol;Acc:HGNC:17844]
-0.234834375083653	0.137021687932812	GNG12	55970	guanine nucleotide binding protein (G protein), gamma 12 [Source:HGNC Symbol;Acc:HGNC:19663]
-0.234593767831199	0.161897076315212	ATP6V0D1	9114	ATPase, H+ transporting, lysosomal 38kDa, V0 subunit d1 [Source:HGNC Symbol;Acc:HGNC:13724]
-0.234541963978383	0.192581494390711	MED17	9440	mediator complex subunit 17 [Source:HGNC Symbol;Acc:HGNC:2375]
-0.234226180871386	0.155902097585224	SLC5A3	6526	solute carrier family 5 (sodium/myo-inositol cotransporter), member 3 [Source:HGNC Symbol;Acc:HGNC:11038]
-0.234089549184284	0.180938215358344	ATF6B	1388	activating transcription factor 6 beta [Source:HGNC Symbol;Acc:HGNC:2349]
-0.233875927997028	0.156902769342684	EP300	2033	E1A binding protein p300 [Source:HGNC Symbol;Acc:HGNC:3373]
-0.23376980179356	0.0652931178283587	MKLN1	4289	muskelin 1, intracellular mediator containing kelch motifs [Source:HGNC Symbol;Acc:HGNC:7109]
-0.233265153716383	0.0727306016392939	RTN4	57142	reticulon 4 [Source:HGNC Symbol;Acc:HGNC:14085]
-0.232883623063668	0.0709790856409344	MYO6	4646	myosin VI [Source:HGNC Symbol;Acc:HGNC:7605]
-0.23273687832076	0.113535476211534	DEFB121	245934	defensin, beta 121 [Source:HGNC Symbol;Acc:HGNC:18101]
-0.232320669826019	0.116811933787521	ZNF786	136051	zinc finger protein 786 [Source:HGNC Symbol;Acc:HGNC:21806]
-0.232254239348175	0.155408502341509	COX4I1	1327	cytochrome c oxidase subunit IV isoform 1 [Source:HGNC Symbol;Acc:HGNC:2265]
-0.232132952521427	0.0129300106000392	UBP1	7342	upstream binding protein 1 (LBP-1a) [Source:HGNC Symbol;Acc:HGNC:12507]

-0.232121404375338	0.142534509996405	CHD9	80205	chromodomain helicase DNA binding protein 9 [Source:HGNC Symbol;Acc:HGNC:25701]
-0.232076562903068	0.0346480123172105	UNC45A	55898	unc-45 homolog A (C. elegans) [Source:HGNC Symbol;Acc:HGNC:30594]
-0.231706477850497	0.155041315262395	FNIP1	96459	folliculin interacting protein 1 [Source:HGNC Symbol;Acc:HGNC:29418]
-0.231469932118255	0.0955688614059661	SCYL1	57410	SCY1-like 1 (S. cerevisiae) [Source:HGNC Symbol;Acc:HGNC:14372]
-0.231430534902222	0.0692613227652204	WDR83OS	51398	WD repeat domain 83 opposite strand [Source:HGNC Symbol;Acc:HGNC:30203]
-0.231250640213934	0.188591448884074	CD151	977	CD151 molecule (Raph blood group) [Source:HGNC Symbol;Acc:HGNC:1630]
-0.231166986840975	0.0709371817933001	SLMAP	7871	sarcolemma associated protein [Source:HGNC Symbol;Acc:HGNC:16643]
-0.230883223883401	0.180245126611748	LMAN2L	81562	lectin, mannose-binding 2-like [Source:HGNC Symbol;Acc:HGNC:19263]
-0.230725616690317	0.1680784001138	EHF	26298	ets homologous factor [Source:HGNC Symbol;Acc:HGNC:3246]
-0.230498465780143	0.0170997311998756	ERGIC1	57222	endoplasmic reticulum-golgi intermediate compartment (ERGIC) 1 [Source:HGNC Symbol;Acc:HGNC:29205]
-0.230469485679253	0.0921913151102534	SATB2	23314	SATB homeobox 2 [Source:HGNC Symbol;Acc:HGNC:21637]
-0.229934978468943	0.0723262027832169	ECH1	1891	enoyl CoA hydratase 1, peroxisomal [Source:HGNC Symbol;Acc:HGNC:3149]
-0.229665141360242	0.169310186936983	NR3C2	4306	nuclear receptor subfamily 3, group C, member 2 [Source:HGNC Symbol;Acc:HGNC:7979]
-0.229579890749234	0.158606075600393	COPB2	9276	coatomer protein complex, subunit beta 2 (beta prime) [Source:HGNC Symbol;Acc:HGNC:2232]
-0.229177709109834	0.195495628041729	ICAM3	3385	intercellular adhesion molecule 3 [Source:HGNC Symbol;Acc:HGNC:5346]
-0.229030205659649	0.0213810613553506	KLHDC10	23008	kelch domain containing 10 [Source:HGNC Symbol;Acc:HGNC:22194]
-0.228446460829669	0.107636112366193	BACE1	23621	beta-site APP-cleaving enzyme 1 [Source:HGNC Symbol;Acc:HGNC:933]
-0.228385882288942	0.199597161353172	TMEM199	147007	transmembrane protein 199 [Source:HGNC Symbol;Acc:HGNC:18085]
-0.228304377562131	0.179674951199072	CDC16	8881	cell division cycle 16 [Source:HGNC Symbol;Acc:HGNC:1720]
-0.227592709342943	0.0503413184214439	STAU1	6780	staufen double-stranded RNA binding protein 1 [Source:HGNC Symbol;Acc:HGNC:11370]
-0.226967243318789	0.0197341381633338	GNB1	2782	guanine nucleotide binding protein (G protein), beta polypeptide 1 [Source:HGNC Symbol;Acc:HGNC:4396]
-0.226917771219184	0.188471838227347	ATPAF1	64756	ATP synthase mitochondrial F1 complex assembly factor 1 [Source:HGNC Symbol;Acc:HGNC:18803]
-0.226830472403622	0.179848857453804	CD68	968	CD68 molecule [Source:HGNC Symbol;Acc:HGNC:1693]
-0.2266730184375	0.118475333447158	UBQLN1	29979	ubiquilin 1 [Source:HGNC Symbol;Acc:HGNC:12508]
-0.226619240887454	0.114998221339077	PCMT1	5110	protein-L-isoaspartate (D-aspartate) O-methyltransferase [Source:HGNC Symbol;Acc:HGNC:8728]
-0.22613221535994	0.127410828923618	DENND4C	55667	DENN/MADD domain containing 4C [Source:HGNC Symbol;Acc:HGNC:26079]
-0.226060193868281	0.0740218234734458	ZBTB34	403341	zinc finger and BTB domain containing 34 [Source:HGNC Symbol;Acc:HGNC:31446]
-0.225520671144037	0.193490723352598	SERINC1	57515	serine incorporator 1 [Source:HGNC Symbol;Acc:HGNC:13464]
-0.225339973009353	0.149417315343117	SLC15A4	121260	solute carrier family 15 (oligopeptide transporter), member 4 [Source:HGNC Symbol;Acc:HGNC:23090]
-0.225209797689086	0.154142097519092	ZNF200	7752	zinc finger protein 200 [Source:HGNC Symbol;Acc:HGNC:12993]
-0.224693220209892	0.180378046560648	RNF214	257160	ring finger protein 214 [Source:HGNC Symbol;Acc:HGNC:25335]
-0.224462072707266	0.134940124910119	RAB5A	5868	RAB5A, member RAS oncogene family [Source:HGNC Symbol;Acc:HGNC:9783]
-0.224380304385143	0.179166531843418	RAB7A	7879	RAB7A, member RAS oncogene family [Source:HGNC Symbol;Acc:HGNC:9788]
-0.223845757479417	0.117008325904309	NUDT11	55190	nudix (nucleoside diphosphate linked moiety X)-type motif 11 [Source:HGNC Symbol;Acc:HGNC:18011]
-0.223720155223974	0.000117541412531421	MAPKAP1	79109	mitogen-activated protein kinase associated protein 1 [Source:HGNC Symbol;Acc:HGNC:18752]
-0.223456892595168	0.154888032192384	LZTS2	84445	leucine zipper, putative tumor suppressor 2 [Source:HGNC Symbol;Acc:HGNC:29381]
-0.223451035665497	0.108101244198189	TMEM131	23505	transmembrane protein 131 [Source:HGNC Symbol;Acc:HGNC:30366]
-0.223255301285641	0.113816133438979	ITFG1	81533	integrin alpha FG-GAP repeat containing 1 [Source:HGNC Symbol;Acc:HGNC:30697]
-0.222792561121899	0.0947219313374489	STARD8	9754	StAR-related lipid transfer (START) domain containing 8 [Source:HGNC Symbol;Acc:HGNC:19161]
-0.222511949887875	0.106873009634258	SNAP29	9342	synaptosomal-associated protein, 29kDa [Source:HGNC Symbol;Acc:HGNC:11133]
-0.222356281724292	0.00170686780879071	TOR1B	27348	torsin family 1, member B (torsin B) [Source:HGNC Symbol;Acc:HGNC:11995]
-0.222029689336745	0.0219997580334186	LNX2	222484	ligand of numb-protein X 2 [Source:HGNC Symbol;Acc:HGNC:20421]
-0.221985508744914	0.198288647311316	DSG2	1829	desmoglein 2 [Source:HGNC Symbol;Acc:HGNC:3049]
-0.221970864141153	0.138066110763588	MCM9	254394	minichromosome maintenance complex component 9 [Source:HGNC Symbol;Acc:HGNC:21484]
-0.221863161853148	0.173236793492439	ACO2	50	aconitase 2, mitochondrial [Source:HGNC Symbol;Acc:HGNC:118]
-0.22131664452476	0.176502184437662	SULT1B1	27284	sulfotransferase family, cytosolic, 1B, member 1 [Source:HGNC Symbol;Acc:HGNC:17845]
-0.220568154951774	0.146925767277017	ERH	2079	enhancer of rudimentary homolog (Drosophila) [Source:HGNC Symbol;Acc:HGNC:3447]
-0.220332942450963	0.0312581194401562	RASAL2	9462	RAS protein activator like 2 [Source:HGNC Symbol;Acc:HGNC:9874]
-0.220018187394037	0.110668632434446	HSD17B11	51170	hydroxysteroid (17-beta) dehydrogenase 11 [Source:HGNC Symbol;Acc:HGNC:22960]
-0.219988177313576	0.165067974822109	FRMD8	83786	FERM domain containing 8 [Source:HGNC Symbol;Acc:HGNC:25462]
-0.219047166244753	0.0968267627405882	WDFY3	23001	WD repeat and FYVE domain containing 3 [Source:HGNC Symbol;Acc:HGNC:20751]
-0.218295827036843	0.0927253117493315	RPLP2	6181	ribosomal protein, large, P2 [Source:HGNC Symbol;Acc:HGNC:10377]
-0.21826071912249	0.115152230464717	TALDO1	6888	transaldolase 1 [Source:HGNC Symbol;Acc:HGNC:11559]
-0.218191557366067	0.0961641753437232	UBL5	59286	ubiquitin-like 5 [Source:HGNC Symbol;Acc:HGNC:13736]
-0.218067884065472	0.0561619300088666	INPP5F	22876	inositol polyphosphate-5-phosphatase F [Source:HGNC Symbol;Acc:HGNC:17054]
-0.217407243703454	0.150294457472426	SNX13	23161	sorting nexin 13 [Source:HGNC Symbol;Acc:HGNC:21335]
-0.217264240570849	0.127978689337105	MORC2	22880	MORC family CW-type zinc finger 2 [Source:HGNC Symbol;Acc:HGNC:23573]
-0.216673179756391	0.164321468135907	SNUPN	10073	snurportin 1 [Source:HGNC Symbol;Acc:HGNC:14245]
-0.216261690913703	0.0978765542682873	TBC1D13	54662	TBC1 domain family, member 13 [Source:HGNC Symbol;Acc:HGNC:25571]
-0.21624411779977	0.155205054705719	SMPD2	6610	sphingomyelin phosphodiesterase 2, neutral membrane (neutral sphingomyelinase) [Source:HGNC Symbol;Acc:HGNC:11121]
-0.215608416446067	0.198742772924705	MBD1	4152	methyl-CpG binding domain protein 1 [Source:HGNC Symbol;Acc:HGNC:6916]
-0.215464650876842	0.155251625952816	CALCOCO2	10241	calcium binding and coiled-coil domain 2 [Source:HGNC Symbol;Acc:HGNC:29912]
-0.215432084478361	0.035007173045499	GABARAPL2	11345	GABA(A) receptor-associated protein-like 2 [Source:HGNC Symbol;Acc:HGNC:13291]
-0.215401178391764	0.132441079868346	CASP12	100506742	caspase 12 (gene/pseudogene) [Source:HGNC Symbol;Acc:HGNC:19004]
-0.215301440694607	0.0704732833415567	PI4KB	5298	phosphatidylinositol 4-kinase, catalytic, beta [Source:HGNC Symbol;Acc:HGNC:8984]
-0.215272210417991	0.106789056176619	KLF7	8609	Kruppel-like factor 7 (ubiquitous) [Source:HGNC Symbol;Acc:HGNC:6350]
-0.214278732598634	0.048361718805892	RNF220	55182	ring finger protein 220 [Source:HGNC Symbol;Acc:HGNC:25552]
-0.214271530853834	0.177291289104893	CNPPD1	27013	cyclin Pas1/PHO80 domain containing 1 [Source:HGNC Symbol;Acc:HGNC:25220]
-0.212976238393097	0.135889752099065	CCNYL2	414194	cyclin Y-like 2 [Source:HGNC Symbol;Acc:HGNC:23495]
-0.212714344990273	0.121957918929501	MIB1	57534	mindbomb E3 ubiquitin protein ligase 1 [Source:HGNC Symbol;Acc:HGNC:21086]
-0.211211149239311	0.171082649799162	TMUB1	83590	transmembrane and ubiquitin-like domain containing 1 [Source:HGNC Symbol;Acc:HGNC:21709]
-0.211177353298063	0.122318696709604	ASH1L	55870	ash1 (absent, small, or homeotic)-like (Drosophila) [Source:HGNC Symbol;Acc:HGNC:19088]
-0.2110092618919	0.135700626209587	TSPAN13	27075	tetraspanin 13 [Source:HGNC Symbol;Acc:HGNC:21643]
-0.210938669717453	0.167638539786387	ARIH1	25820	ariadne RBR E3 ubiquitin protein ligase 1 [Source:HGNC Symbol;Acc:HGNC:689]
-0.210848590890505	0.126929973564634	QARS	5859	glutaminyl-tRNA synthetase [Source:HGNC Symbol;Acc:HGNC:9751]
-0.210661493049232	0.104938973730378	RGL1	23179	ral guanine nucleotide dissociation stimulator-like 1 [Source:HGNC Symbol;Acc:HGNC:30281]
-0.210355928216628	0.191045823302098	CNOT8	9337	CCR4-NOT transcription complex, subunit 8 [Source:HGNC Symbol;Acc:HGNC:9207]
-0.209826026050922	0.138070220145522	RRAS2	22800	related RAS viral (r-ras) oncogene homolog 2 [Source:HGNC Symbol;Acc:HGNC:17271]
-0.209645954389444	0.0875374814539549	FBXO38	81545	F-box protein 38 [Source:HGNC Symbol;Acc:HGNC:28844]
-0.209451989939507	0.138768670610027	MPC2	25874	mitochondrial pyruvate carrier 2 [Source:HGNC Symbol;Acc:HGNC:24515]
-0.209407609700428	0.169952885701424	B4GALT3	8703	UDP-Gal:betaGlcNAc beta 1,4- galactosyltransferase, polypeptide 3 [Source:HGNC Symbol;Acc:HGNC:926]
-0.209390470834769	0.199838227868728	TUBB6	84617	tubulin, beta 6 class V [Source:HGNC Symbol;Acc:HGNC:20776]
-0.209263447354832	0.085024544772532	SLC2A8	29988	solute carrier family 2 (facilitated glucose transporter), member 8 [Source:HGNC Symbol;Acc:HGNC:13812]
-0.20863003767793	0.10997712553171	RAB1B	81876	RAB1B, member RAS oncogene family [Source:HGNC Symbol;Acc:HGNC:18370]
-0.20821648515345	0.12226951920471	VKORC1	79001	vitamin K epoxide reductase complex, subunit 1 [Source:HGNC Symbol;Acc:HGNC:23663]
-0.208188836283884	0.124208746260826	TMEM33	55161	transmembrane protein 33 [Source:HGNC Symbol;Acc:HGNC:25541]
-0.207146119113793	0.163749600369327	NECAP2	55707	NECAP endocytosis associated 2 [Source:HGNC Symbol;Acc:HGNC:25528]
-0.205913552162121	0.181165601636888	CSF2RA	1438	colony stimulating factor 2 receptor, alpha, low-affinity (granulocyte-macrophage) [Source:HGNC Symbol;Acc:HGNC:2435]
-0.205563318407293	0.0513712046879213	MEAF6	64769	MYST/Esa1-associated factor 6 [Source:HGNC Symbol;Acc:HGNC:25674]
-0.205520403339878	0.18084582123695	RRAGC	64121	Ras-related GTP binding C [Source:HGNC Symbol;Acc:HGNC:19902]
-0.20457162310435	0.10277749206153	YPEL5	51646	yippee-like 5 (Drosophila) [Source:HGNC Symbol;Acc:HGNC:18329]
-0.204355950671506	0.177028411825947	TM9SF4	9777	transmembrane 9 superfamily protein member 4 [Source:HGNC Symbol;Acc:HGNC:30797]
-0.204305779755149	0.105561763970074	SLC16A4	9122	solute carrier family 16, member 4 [Source:HGNC Symbol;Acc:HGNC:10925]
-0.203609897437143	0.0366583028110416	FBXW11	23291	F-box and WD repeat domain containing 11 [Source:HGNC Symbol;Acc:HGNC:13607]
-0.202570182990177	0.151851585232452	APP	351	amyloid beta (A4) precursor protein [Source:HGNC Symbol;Acc:HGNC:620]
-0.202259549896886	0.109208396058443	WNK1	65125	WNK lysine deficient protein kinase 1 [Source:HGNC Symbol;Acc:HGNC:14540]
-0.202022779212537	0.152957613437688	GJC1	10052	gap junction protein, gamma 1, 45kDa [Source:HGNC Symbol;Acc:HGNC:4280]
-0.201807423486556	0.102403692432066	TMEM59	9528	transmembrane protein 59 [Source:HGNC Symbol;Acc:HGNC:1239]
-0.201676274040012	0.138521392925703	WDR1	9948	WD repeat domain 1 [Source:HGNC Symbol;Acc:HGNC:12754]
-0.200900408662676	0.0548330589063002	PLEKHA1	59338	pleckstrin homology domain containing, family A (phosphoinositide binding specific) member 1 [Source:HGNC Symbol;Acc:HGNC:14335]
-0.200132515540405	0.0950716797483266	ATRAID	51374	all-trans retinoic acid-induced differentiation factor [Source:HGNC Symbol;Acc:HGNC:24090]
-0.199968974100909	0.0739367922738689	CUL4B	8450	cullin 4B [Source:HGNC Symbol;Acc:HGNC:2555]
-0.199358612701785	0.0895889891217916	CYB5B	80777	cytochrome b5 type B (outer mitochondrial membrane) [Source:HGNC Symbol;Acc:HGNC:24374]
-0.198562463512155	0.0863373694425972	UBE2I	7329	ubiquitin-conjugating enzyme E2I [Source:HGNC Symbol;Acc:HGNC:12485]
-0.198146886805491	0.191263663320918	PPDPF	79144	pancreatic progenitor cell differentiation and proliferation factor [Source:HGNC Symbol;Acc:HGNC:16142]
-0.197637863358632	0.182105853640226	ANKRD13C	81573	ankyrin repeat domain 13C [Source:HGNC Symbol;Acc:HGNC:25374]
-0.19754737421962	0.132843200927759	ZNF428	126299	zinc finger protein 428 [Source:HGNC Symbol;Acc:HGNC:20804]
-0.197520111166858	0.198317941327822	CAMK2D	817	calcium/calmodulin-dependent protein kinase II delta [Source:HGNC Symbol;Acc:HGNC:1462]
-0.197465973075859	0.183650892880911	MED29	55588	mediator complex subunit 29 [Source:HGNC Symbol;Acc:HGNC:23074]
-0.196879047059536	0.0895025372489849	TSPYL1	7259	TSPY-like 1 [Source:HGNC Symbol;Acc:HGNC:12382]
-0.196109071211812	0.0687441803316794	ZDHHC3	51304	zinc finger, DHHC-type containing 3 [Source:HGNC Symbol;Acc:HGNC:18470]
-0.195459646651243	0.153513124426988	DEFA4	1669	defensin, alpha 4, corticostatin [Source:HGNC Symbol;Acc:HGNC:2763]
-0.1951750523593	0.130203506252905	MAP4K3	8491	mitogen-activated protein kinase kinase kinase kinase 3 [Source:HGNC Symbol;Acc:HGNC:6865]
-0.194207766526593	0.125643778233584	BUB3	9184	BUB3 mitotic checkpoint protein [Source:HGNC Symbol;Acc:HGNC:1151]
-0.193672219527253	0.153714387616994	CANX	821	calnexin [Source:HGNC Symbol;Acc:HGNC:1473]
-0.193665711441814	0.180286719815748	SLC8A3	6547	solute carrier family 8 (sodium/calcium exchanger), member 3 [Source:HGNC Symbol;Acc:HGNC:11070]
-0.193546737174234	0.137813472785505	ERC1	23085	ELKS/RAB6-interacting/CAST family member 1 [Source:HGNC Symbol;Acc:HGNC:17072]
-0.191839303502093	0.174123637832204	MRPS18A	55168	mitochondrial ribosomal protein S18A [Source:HGNC Symbol;Acc:HGNC:14515]
-0.191341535899661	0.188735199930978	SERPINA12	145264	serpin peptidase inhibitor, clade A (alpha-1 antiproteinase, antitrypsin), member 12 [Source:HGNC Symbol;Acc:HGNC:18359]
-0.191109565916895	0.171537454522749	GLIPR1L2	144321	GLI pathogenesis-related 1 like 2 [Source:HGNC Symbol;Acc:HGNC:28592]
-0.191043593136585	0.0465690546052581	KRTCAP2	200185	keratinocyte associated protein 2 [Source:HGNC Symbol;Acc:HGNC:28942]
-0.190272723560732	0.188147748860471	R3HCC1L	27291	R3H domain and coiled-coil containing 1-like [Source:HGNC Symbol;Acc:HGNC:23512]
-0.190186930450981	0.189889778228378	GOLIM4	27333	golgi integral membrane protein 4 [Source:HGNC Symbol;Acc:HGNC:15448]
-0.189100563390522	0.165203597472445	TACC1	6867	transforming, acidic coiled-coil containing protein 1 [Source:HGNC Symbol;Acc:HGNC:11522]
-0.188233105427748	0.0897433581947394	CASP10	843	caspase 10, apoptosis-related cysteine peptidase [Source:HGNC Symbol;Acc:HGNC:1500]
-0.188226632463723	0.108853331549054	TMEM248	55069	transmembrane protein 248 [Source:HGNC Symbol;Acc:HGNC:25476]
-0.188128774847149	0.148103851181153	SPATA6	54558	spermatogenesis associated 6 [Source:HGNC Symbol;Acc:HGNC:18309]
-0.187882546118938	0.161697250413132	ILF2	3608	interleukin enhancer binding factor 2 [Source:HGNC Symbol;Acc:HGNC:6037]
-0.187880841859511	0.167505626317579		432369	
-0.186937786307285	0.151472784437346	DAZAP2	9802	DAZ associated protein 2 [Source:HGNC Symbol;Acc:HGNC:2684]
-0.186424349627133	0.168279563348867	CLMN	79789	calmin (calponin-like, transmembrane) [Source:HGNC Symbol;Acc:HGNC:19972]
-0.186056269316839	0.120339744841769	UBE2K	3093	ubiquitin-conjugating enzyme E2K [Source:HGNC Symbol;Acc:HGNC:4914]
-0.185698191138209	0.136580338064878	FOXJ3	22887	forkhead box J3 [Source:HGNC Symbol;Acc:HGNC:29178]
-0.185068434252829	0.12855537615184	CNOT11	55571	CCR4-NOT transcription complex, subunit 11 [Source:HGNC Symbol;Acc:HGNC:25217]
-0.184159884650885	0.164256547806657	LRRFIP2	9209	leucine rich repeat (in FLII) interacting protein 2 [Source:HGNC Symbol;Acc:HGNC:6703]
-0.18274072352526	0.120671899459406	NDN	4692	necdin, melanoma antigen (MAGE) family member [Source:HGNC Symbol;Acc:HGNC:7675]
-0.182554137519452	0.189492775390773		22902	
-0.182045760924944	0.180786729480755	FBXW2	26190	F-box and WD repeat domain containing 2 [Source:HGNC Symbol;Acc:HGNC:13608]
-0.18162757082541	0.0482847765714527	ZXDC	79364	ZXD family zinc finger C [Source:HGNC Symbol;Acc:HGNC:28160]
-0.181495381852186	0.041059194060387	DHRS7	51635	dehydrogenase/reductase (SDR family) member 7 [Source:HGNC Symbol;Acc:HGNC:21524]
-0.181405003813212	0.178131877043007	FNIP2	57600	folliculin interacting protein 2 [Source:HGNC Symbol;Acc:HGNC:29280]

-0.18123908383652	0.187188339524649	KLF5	688	Kruppel-like factor 5 (intestinal) [Source:HGNC Symbol;Acc:HGNC:6349]
-0.181077257956306	0.173206063795828		1197	
-0.180669748176287	0.142876065229825	LRBA	987	LPS-responsive vesicle trafficking, beach and anchor containing [Source:HGNC Symbol;Acc:HGNC:1742]
-0.180504874477146	0.18922401003944	SPAG1	6674	sperm associated antigen 1 [Source:HGNC Symbol;Acc:HGNC:11212]
-0.179609681583819	0.134287263488954	WAC	51322	WW domain containing adaptor with coiled-coil [Source:HGNC Symbol;Acc:HGNC:17327]
-0.179328208533876	0.151121838300268	SRI	6717	sorcin [Source:HGNC Symbol;Acc:HGNC:11292]
-0.179018594775468	0.11540466563004	STIM2	57620	stromal interaction molecule 2 [Source:HGNC Symbol;Acc:HGNC:19205]
-0.178958681573632	0.173610409340054	VKORC1L1	154807	vitamin K epoxide reductase complex, subunit 1-like 1 [Source:HGNC Symbol;Acc:HGNC:21492]
-0.178058669337427	0.0704236088648211	C19orf43	79002	chromosome 19 open reading frame 43 [Source:HGNC Symbol;Acc:HGNC:28424]
-0.177145053827369	0.141750721064799	TRAPPC5	126003	trafficking protein particle complex 5 [Source:HGNC Symbol;Acc:HGNC:23067]
-0.176868675426915	0.13854110297457	DOCK9	23348	dedicator of cytokinesis 9 [Source:HGNC Symbol;Acc:HGNC:14132]
-0.176494072490252	0.167281090159391	DENND1B	163486	DENN/MADD domain containing 1B [Source:HGNC Symbol;Acc:HGNC:28404]
-0.175053616949502	0.144935584407044	MIR323A	442897	microRNA 323a [Source:HGNC Symbol;Acc:HGNC:31766]
-0.174783623604211	0.169283874295072	EEF1D	1936	eukaryotic translation elongation factor 1 delta (guanine nucleotide exchange protein) [Source:HGNC Symbol;Acc:HGNC:3211]
-0.174610361041199	0.17261168122242	BLMH	642	bleomycin hydrolase [Source:HGNC Symbol;Acc:HGNC:1059]
-0.174253033579721	0.124434080267083	PTRF	284119	polymerase I and transcript release factor [Source:HGNC Symbol;Acc:HGNC:9688]
-0.173259145682416	0.139748485149888	CHST1	8534	carbohydrate (keratan sulfate Gal-6) sulfotransferase 1 [Source:HGNC Symbol;Acc:HGNC:1969]
-0.172371193142201	0.0622100825964957	EDF1	8721	endothelial differentiation-related factor 1 [Source:HGNC Symbol;Acc:HGNC:3164]
-0.171065163296905	0.176126696364329	TRAM1	23471	translocation associated membrane protein 1 [Source:HGNC Symbol;Acc:HGNC:20568]
-0.170874317704485	0.129243382521859	TMEM205	374882	transmembrane protein 205 [Source:HGNC Symbol;Acc:HGNC:29631]
-0.17000627340964	0.175187511740901	MFN2	9927	mitofusin 2 [Source:HGNC Symbol;Acc:HGNC:16877]
-0.169958755691311	0.141187255648882	HLA-B	3106	major histocompatibility complex, class I, B [Source:HGNC Symbol;Acc:HGNC:4932]
-0.169882495906517	0.0782317239364241	UQCR10	29796	ubiquinol-cytochrome c reductase, complex III subunit X [Source:HGNC Symbol;Acc:HGNC:30863]
-0.168722721814873	0.124752261909163	UBE3C	9690	ubiquitin protein ligase E3C [Source:HGNC Symbol;Acc:HGNC:16803]
-0.168210804626024	0.161332926471249	ATP5B	506	ATP synthase, H+ transporting, mitochondrial F1 complex, beta polypeptide [Source:HGNC Symbol;Acc:HGNC:830]
-0.167200108051746	0.140242211493336	CHKA	1119	choline kinase alpha [Source:HGNC Symbol;Acc:HGNC:1937]
-0.166945165331513	0.176868929422457	NDUFB4	4710	NADH dehydrogenase (ubiquinone) 1 beta subcomplex, 4, 15kDa [Source:HGNC Symbol;Acc:HGNC:7699]
-0.166891092698289	0.143880059853764	FGFR1OP	11116	FGFR1 oncogene partner [Source:HGNC Symbol;Acc:HGNC:17012]
-0.166287122566652	0.185281995725602	GRSF1	2926	G-rich RNA sequence binding factor 1 [Source:HGNC Symbol;Acc:HGNC:4610]
-0.166060106776032	0.163738680677554	FAM160A1	729830	family with sequence similarity 160, member A1 [Source:HGNC Symbol;Acc:HGNC:34237]
-0.165901938992056	0.182384804169479	SMURF1	57154	SMAD specific E3 ubiquitin protein ligase 1 [Source:HGNC Symbol;Acc:HGNC:16807]
-0.165514855467068	0.0989816416528218	MS4A13	503497	membrane-spanning 4-domains, subfamily A, member 13 [Source:HGNC Symbol;Acc:HGNC:16674]
-0.164951480496725	0.0658362775450917	NF1	4763	neurofibromin 1 [Source:HGNC Symbol;Acc:HGNC:7765]
-0.164705286073599	0.124907082250738	PHACTR4	65979	phosphatase and actin regulator 4 [Source:HGNC Symbol;Acc:HGNC:25793]
-0.164620188875205	0.143518428275215	NPTN	27020	neuroplastin [Source:HGNC Symbol;Acc:HGNC:17867]
-0.163880583555698	0.175030210506501	THYN1	29087	thymocyte nuclear protein 1 [Source:HGNC Symbol;Acc:HGNC:29560]
-0.163728487422726	0.177664383686475	HNRNPUL1	11100	heterogeneous nuclear ribonucleoprotein U-like 1 [Source:HGNC Symbol;Acc:HGNC:17011]
-0.163595801896545	0.0762985277299496	ZMAT5	55954	zinc finger, matrin-type 5 [Source:HGNC Symbol;Acc:HGNC:28046]
-0.163247817859957	0.0369542890964822	LPAR1	1902	lysophosphatidic acid receptor 1 [Source:HGNC Symbol;Acc:HGNC:3166]
-0.161909579224155	0.13491197690239	CSTB	1476	cystatin B (stefin B) [Source:HGNC Symbol;Acc:HGNC:2482]
-0.1616252599483	0.192089258407531	SLC26A4	5172	solute carrier family 26 (anion exchanger), member 4 [Source:HGNC Symbol;Acc:HGNC:8818]
-0.160854674664497	0.112471500883544	DTX3L	151636	deltex 3 like, E3 ubiquitin ligase [Source:HGNC Symbol;Acc:HGNC:30323]
-0.160343992123002	0.0992035406909443	ATP5O	539	ATP synthase, H+ transporting, mitochondrial F1 complex, O subunit [Source:HGNC Symbol;Acc:HGNC:850]
-0.159925275715573	0.116874204889711	ARF1	375	ADP-ribosylation factor 1 [Source:HGNC Symbol;Acc:HGNC:652]
-0.15986853207931	0.195058721745809	TRIP12	9320	thyroid hormone receptor interactor 12 [Source:HGNC Symbol;Acc:HGNC:12306]
-0.159256873127406	0.142993562079463	GLYR1	84656	glyoxylate reductase 1 homolog (Arabidopsis) [Source:HGNC Symbol;Acc:HGNC:24434]
-0.157807388082621	0.0578775587781468	SAP18	10284	Sin3A-associated protein, 18kDa [Source:HGNC Symbol;Acc:HGNC:10530]
-0.15529229742852 3	0.0636373417980589	CAPZB	832	capping protein (actin filament) muscle Z-line, beta [Source:HGNC Symbol;Acc:HGNC:1491]
-0.152835634616528	0.199629570844859	ELAVL3	1995	ELAV like neuron-specific RNA binding protein 3 [Source:HGNC Symbol;Acc:HGNC:3314]
-0.152469754989903	0.125655666366266	MBNL1	4154	muscleblind-like splicing regulator 1 [Source:HGNC Symbol;Acc:HGNC:6923]
-0.150940734130392	0.128324130683389	CRTAP	10491	cartilage associated protein [Source:HGNC Symbol;Acc:HGNC:2379]
-0.149540215457467	0.161134373990219	ATP6V0C	527	ATPase, H+ transporting, lysosomal 16kDa, V0 subunit c [Source:HGNC Symbol;Acc:HGNC:855]
-0.148956379545105	0.162907295096534	BRAF	673	B-Raf proto-oncogene, serine/threonine kinase [Source:HGNC Symbol;Acc:HGNC:1097]
-0.142842025985182	0.157825494865287	PKD2	5311	polycystic kidney disease 2 (autosomal dominant) [Source:HGNC Symbol;Acc:HGNC:9009]
-0.142771407537712	0.170307260047506	SSR2	6746	signal sequence receptor, beta (translocon-associated protein beta) [Source:HGNC Symbol;Acc:HGNC:11324]
-0.142162745668041	0.139810366644512	SUMO3	6612	small ubiquitin-like modifier 3 [Source:HGNC Symbol;Acc:HGNC:11124]
-0.141386382503546	0.157630104254417	DYNC1LI2	1783	dynein, cytoplasmic 1, light intermediate chain 2 [Source:HGNC Symbol;Acc:HGNC:2966]
-0.140254262199142	0.198769181607147	RAD23B	5887	RAD23 homolog B (S. cerevisiae) [Source:HGNC Symbol;Acc:HGNC:9813]
-0.139305136894997	0.185213238450524	ZDHHC5	25921	zinc finger, DHHC-type containing 5 [Source:HGNC Symbol;Acc:HGNC:18472]
-0.136770835281887	0.191950239235552	SPIN1	10927	spindlin 1 [Source:HGNC Symbol;Acc:HGNC:11243]
-0.135483498660988	0.159627937109468	ANKRD17	26057	ankyrin repeat domain 17 [Source:HGNC Symbol;Acc:HGNC:23575]
-0.130723645137968	0.167216404250756	CDC42SE2	56990	CDC42 small effector 2 [Source:HGNC Symbol;Acc:HGNC:18547]
-0.124431897549514	0.109961968604218	ESRP1	54845	epithelial splicing regulatory protein 1 [Source:HGNC Symbol;Acc:HGNC:25966]
-0.118681900187337	0.140224075585938	RAB5B	5869	RAB5B, member RAS oncogene family [Source:HGNC Symbol;Acc:HGNC:9784]
-0.112781444903457	0.162784450756365	UCK1	83549	uridine-cytidine kinase 1 [Source:HGNC Symbol;Acc:HGNC:14859]
-0.103277788133514	0.136775018953343	MAP3K4	4216	mitogen-activated protein kinase kinase kinase 4 [Source:HGNC Symbol;Acc:HGNC:6856]
-0.0999016767274116	0.141596275219037	ZMYND11	10771	zinc finger, MYND-type containing 11 [Source:HGNC Symbol;Acc:HGNC:16966]
-0.0972678791999481	0.196952404971495	RALGAPB	57148	Ral GTPase activating protein, beta subunit (non-catalytic) [Source:HGNC Symbol;Acc:HGNC:29221]
-0.0958738515793023	0.196612351796802	IFNA1	3447	interferon, alpha 1 [Source:HGNC Symbol;Acc:HGNC:5417]
